# Quantitative Trait Locus Mapping of Melanization in the Plant Pathogenic Fungus *Zymoseptoria tritici*

**DOI:** 10.1534/g3.114.015289

**Published:** 2014-10-29

**Authors:** Mark H. Lendenmann, Daniel Croll, Ethan L. Stewart, Bruce A. McDonald

**Affiliations:** Institute of Integrative Biology, Plant Pathology, ETH Zürich, 8092 Zürich, Switzerland

**Keywords:** *Mycosphaerella graminicola*, genetic linkage map, QTL, restriction site–associated DNA sequencing (RADseq), automated digital image analysis

## Abstract

Melanin plays an important role in virulence and antimicrobial resistance in several fungal pathogens. The wheat pathogen *Zymoseptoria tritici* is important worldwide, but little is known about the genetic architecture of pathogenicity, including the production of melanin. Because melanin production can exhibit complex inheritance, we used quantitative trait locus (QTL) mapping in two crosses to identify the underlying genes. Restriction site−associated DNA sequencing was used to genotype 263 (cross 1) and 261 (cross 2) progeny at ~8500 single-nucleotide polymorphisms and construct two dense linkage maps. We measured gray values, representing degrees of melanization, for single-spore colonies growing on Petri dishes by using a novel image-processing approach that enabled high-throughput phenotyping. Because melanin production can be affected by stress, each offspring was grown in two stressful environments and one control environment. We detected six significant QTL in cross 1 and nine in cross 2, with three QTL shared between the crosses. Different QTL were identified in different environments and at different colony ages. By obtaining complete genome sequences for the four parents and analyzing sequence variation in the QTL confidence intervals, we identified 16 candidate genes likely to affect melanization. One of these candidates was *PKS1*, a polyketide synthase gene known to play a role in the synthesis of dihydroxynaphthalene melanin. Three candidate quantitative trait nucleotides were identified in *PKS1*. Many of the other candidate genes were not previously associated with melanization.

Most fungi produce melanin pigments that are located mainly in their cell walls. Melanins are dark, often black, biological macromolecules composed of various types of phenolic or indolic monomers that often form complexes with proteins and carbohydrates. Proposed functions of fungal melanins include protection against irradiation, enzymatic lysis, and extreme temperatures (Butler and Day 1998). Melanin also plays an important role in virulence (Nosanchuk *et al.* 2000; Morris-Jones *et al.* 2003; Youngchim *et al.* 2004; Mednick *et al.* 2005; Ngamskulrungroj and Meyer 2009) and resistance to antimicrobial compounds (Larsson and Tjalve 1979; Ikeda *et al.* 2003; Nosanchuk and Casadevall 2006; Taborda *et al.* 2008; Liaw *et al.* 2010). Melanin is needed in appressoria to contain the high turgor pressure formed during penetration of the plant epidermis by fungal pathogens (Howard *et al.* 1991). Thus, melanization plays an important role in fungal biology and in host−parasite interactions.

At least four melanins have been identified in fungi, and two of these, dihydroxynaphthalene (DHN) and dihydroxyphenylalanine melanin, have been subjected to intensive study. DHN melanin is considered to be the main fungal melanin. It is produced by a wide range of plant pathogenic fungi and is the best characterized fungal melanin, with a known biosynthetic pathway. The genetic basis of melanization can differ among fungi and the complexity in known melanin production pathways suggested that a quantitative trait locus (QTL) mapping approach would be useful to identify candidate genes (Butler and Day 1998).

*Zymoseptoria tritici* (syn *Mycosphaerella graminicola*) is a heterothallic, hemibiotrophic filamentous ascomycete that causes the foliar disease Septoria tritici blotch on wheat worldwide. Under favorable conditions, yield losses can reach 30–50% (Eyal *et al.* 1987), especially in regions with humid and temperate climates such as Northwestern Europe. *Z. tritici* is currently one of the most important wheat pathogens in Europe (Hardwick *et al.* 2001; O’Driscoll *et al.* 2014); however, only eight genes involved in melanization have been investigated in *Z. tritici*. These eight genes encoded G proteins (Mehrabi *et al.* 2009), mitogen-activated protein kinases (Cousin *et al.* 2006; Mehrabi *et al.* 2006a; Mehrabi *et al.* 2006b), a velvet protein (Choi and Goodwin 2011b), and a c-type cyclin (Choi and Goodwin 2011a). The G proteins and c-type cyclin had been associated with melanization in other filamentous fungi, but none of the eight genes were associated with a specific melanin biosynthetic pathway (Butler and Day 1998). The effect of each gene on melanin production was validated by the use of knockout mutants. Seven of the knockout mutants showed reduced virulence in addition to reduced melanization (Cousin *et al.* 2006; Mehrabi *et al.* 2006a,b; 2009; Choi and Goodwin 2011b). The eighth knockout mutant exhibited increased melanization and reduced virulence (Choi and Goodwin 2011a). Lower melanization was correlated with greater sensitivity to azole fungicides in one of the mutants (Mehrabi *et al.* 2006a), but in another study a knockout of a different gene (Mehrabi *et al.* 2006b) showed lower melanization as well as lower sensitivity to phenylpyrrole and dicarboximide fungicides. Hence, the typical pattern of greater melanization correlating with greater virulence and lesser fungicide sensitivity is not clearly established in *Z. tritici*.

Melanization is likely a quantitative trait in *Z. tritici* because several genes were associated with the trait in other pathogenic fungi (Takano *et al.* 1995; Eliahu *et al.* 2007; Karkowska-Kuleta *et al.* 2009; Fujihara *et al.* 2010; Ipcho *et al.* 2012; Lin *et al.* 2012; Upadhyay *et al.* 2013) and some gene knockouts caused only reduced melanin production (Kimura *et al.* 2001; Parisot *et al.* 2002; Solomon *et al.* 2004; Lin *et al.* 2006; Mehrabi *et al.* 2009). We tested this hypothesis by using QTL mapping to determine the genetic architecture of melanization in *Z. tritici*. QTL mapping enables the identification and characterization of the chromosomal segments and corresponding genes that encode quantitative traits (Mackay 2001). QTL mapping has been used extensively in animals (Goddard and Hayes 2009), including humans (Flint and Mackay 2009) and also plants (Holland 2007), but very few QTL mapping studies have been reported for filamentous fungi (Foulongne-Oriol 2012). Although fungal melanin biosynthetic pathways were characterized in earlier studies, additional melanin genes were identified in recent studies (Fujihara *et al.* 2010; Choi and Goodwin 2011a,b), indicating that melanin biosynthetic pathways may be more complex than previously thought. QTL mapping was applied to fungal melanization in an earlier study with *Cryptococcus neoformans* (Lin *et al.* 2006), but this is the first QTL analysis of melanization in a fungal plant pathogen.

We used two mapping populations derived from four unique wild-type strains to identify QTL involved in melanization. Because melanin production can be affected by stress (Butler and Day 1998; Henson *et al.* 1999; Jacobson 2000), we exposed each offspring to two stressful environments, cold temperature and sublethal fungicide exposure, that could be compared with a control environment to determine whether temperature or fungicide stress would result in environment-dependent QTL. We chose a next-generation sequencing genotyping method called restriction site−associated DNA sequencing (RADseq) to construct two highly dense genetic maps using single-nucleotide polymorphism (SNP) markers covering most of the reference genome. Phenotyping was based on high-throughput digital image processing to score the melanization of single-spore colonies grown on solid media.

## Material and Methods

### Generation of mapping populations

Two crosses were made between four *Z. tritici* isolates. Isolate ST99CH3D1 (3D1: SRS383146) was crossed to ST99CH3D7 (3D7: SRS383147), and ST99CH1A5 (1A5: SRS383142) was crossed to ST99CH1E4 (1E4: SRS383143). All four parents were collected from naturally infected wheat fields in 1999 in Switzerland. The isolates were previously characterized phenotypically (Zhan *et al.* 2002) and genetically (Zhan *et al.* 2002; Croll *et al.* 2013) and found to differ for virulence and several life history traits, including melanization. Crosses were performed by coinfecting wheat leaves via an established protocol (Kema *et al.* 1996). Ascospores were collected, grown *in vitro*, and stored on anhydrous silica gel and glycerol at −80°. Cross 3D1 × 3D7 produced 359 progeny and cross 1A5 × 1E4 produced 341 progeny.

### Genotyping

We used RADseq (Baird *et al.* 2008) to identify SNP markers segregating in the progeny populations. RADseq SNPs in the parental strains were confirmed using complete parental genome sequences obtained previously (Croll *et al.* 2013). National Center for Biotechnology Information (NCBI) BioSample accession numbers of parental strains were as follows: SRS383146 (ST99CH3D1), SRS383147 (ST99CH3D7), SRS383142 (ST99CH1A5), and SRS383143 (ST99CH1E4). To construct libraries, spores were lyophilized, and DNA was extracted using the DNeasy plant mini kit (QIAGEN Inc., Basel, Switzerland). DNA was then quantified and standardized for all samples. A total of 1.5 μg of genomic DNA per progeny was digested with the restriction enzyme *Pst*1. Libraries and pools were constructed following a modified RADseq protocol (Etter *et al.* 2011). The main modification was to use Illumina TrueSeq compatible P2 adapters. Pools containing an average of 132 progeny were generated, with each pool consisting of six different P2 adapters. 22 P1 adapters with distinct inline barcodes were used to distinguish progeny DNA with an identical P2 adapter. Pools were sequenced on an Illumina HiSeq2000 in 100 bp paired-end mode.

Raw sequence reads were quality checked using the tool FastQC (Babraham Bioinformatics; Cambridge, UK). Reads were then quality trimmed with Trimmomatic v. 0.30 (Lohse *et al.* 2012) by using the following settings upon phred + 33 quality scores: trailing = 3, slidingwindow = 20:5 and minlen = 50. Progeny reads were separated according to the P1 adapter using the FASTX-Toolkit v. 0.13 (Hannon Lab: http://hannonlab.cshl.edu/fastx_toolkit/links.html). Progeny reads were aligned individually against the IPO323 reference genome (assembly version MG2, Sept 2008) (Goodwin *et al.* 2011) using the short-read aligner Bowtie 2 version 2.0.2 (Langmead and Salzberg 2012) to create progeny sequence alignment/map (sam) files. Default settings for a sensitive end-to-end alignment were used (-D 15; -R 2; -L 22; -I S, 1,1.15). All four parental genome sequences (Croll *et al.* 2013) also were aligned to the reference genome using identical trimming and assembly parameters. Parental and progeny sam files were converted into binary alignment/map files (*i.e.*, bam) using SAMtools version 0.1.18 (Li *et al.* 2009). RADseq progeny-aligned read data are available from the NCBI Short Read Archive under the BioProject accession numbers PRJNA256988 (cross: ST99CH3D1 × ST99CH3D7) and PRJNA256991 (cross: ST99CH1A5 × ST99CH1E4).

SNPs were identified using the Genome Analysis Toolkit (GATK), version 2.6-4-g3e5ff60 (Depristo *et al.* 2011) and VCFtools version 0.1.10 (http://vcftools.sourceforge.net). Initial SNP calling was performed in comparison with the reference genome using the GATK UnifiedGenotyper with a maximum alternative allele setting of 1. The sample-level was set to ploidy of 1 (haploid) using the sequence alignment/map files of progeny of one of the crosses combined with their respective parents. The genotype likelihood model was set to the SNP general ploidy model. Marker genotype SNP filtering was performed using GATK VariantFiltration, by setting the following filters for SNPs to pass: quality by depth (QD ≥ 5), Fisher’s exact test for strand bias (FS ≤ 60), haplotype score (HaplotypeScore ≤ 10), overall quality score (QUAL ≥ 1000), lower and upper allele frequency (AFlower ≥ 0.2 and AFupper ≤ 0.8), and total number of alleles within each marker genotype (AN ≥ 60). This filter excluded SNPs only called among the parental genomes. Progeny and parent genotypes were then filtered further using a phred-scaled genotype quality setting of at least 30.

### Genetic map construction and quality assessment

The construction of the linkage map was performed using R/qtl version 1.27-10 (Arends *et al.* 2010), a package of the open-source software R (R_Core_Team 2012). A genotype matrix was constructed containing only progeny with a minimum of 45% of all SNPs genotyped. We further omitted any markers if less than 70% of the progeny were genotyped. Potential clones in the progeny populations were excluded by randomly selecting one progeny from a group of potential clones. Clones were defined as having at least 90% of SNP alleles in common. Adjacent nonrecombining markers were reduced to retain only a single marker for each cluster of nonrecombining markers. We excluded any markers showing significant evidence of non-Mendelian segregation (χ^2^ test: *P* < 0.1), but no progeny genotypes matched the omission criteria for segregation distortion. Progeny genotypes were further investigated for evidence of genotyping errors (error.prob < 0.01). Markers with significant evidence for genotyping error in at least one progeny were excluded from all progeny. NCBI Short Read Archive accession numbers for the retained progeny within each cross can be found in Supporting Information, Table S1.

The genetic map was constructed by estimating the genetic distance among pairs of neighboring markers and translating recombination frequency into map distance (centiMorgans, cM). We checked for appropriate linkage group assignment, order and binary status among the retained markers by inspecting each genetic map for unusual inflation patterns. We constructed a plot of pairwise marker recombination fractions and logarithm of odds (LOD) scores for tests of r = 1/2 (heat map). A plot with a diagonal red line indicates a good assignment with consistent marker order and an absence of switched alleles. We compared the chromosome coverage of our linkage groups relative to the reference genome IPO323.

### Phenotyping

Melanization was measured using a Petri dish assay combined with digital image analysis. Progeny of each cross were retrieved from long-term storage and grown on yeast malt sucrose agar (4 g/L yeast extract, 4 g/L malt extract, 4 g/L sucrose, and 50 mg/L kanamycin). Petri dishes were stored for approximately 4 d at 18°. A total of 600 μL of sterile water was added to each plate, and blastospores were gently scraped into the solution using a sterile glass slide. A total of 450 μL of the highly concentrated spore suspension were then transferred into sterile 500-μL Eppendorf tubes and stored for no longer then 3 mo at −20°. Spores of each offspring were taken from these tubes and diluted in sterile water to a concentration of 200 spores per milliliter using a hemocytometer. A total of 500 μL of the spore suspension was spread across Petri plates containing potato dextrose agar (PDA; 4 g/L potato starch, 20 g/L dextrose, 15 g/L agar) using a sterile glass rod. Only single-spore colonies were scored. An average of 20 colonies formed on each plate, but only nine individual colonies were scored on average because ~50% of all colonies had fused with a neighboring colony. Each treatment produced the same average number of colonies.

Each progeny was exposed to two stress environments (cold temperature and sublethal fungicide concentration) and one control environment, with five technical repeats for each environment. The data point from a technical repeat was the average gray value from an average of nine colonies scored on one Petri dish. The control environment consisted of PDA plates growing at 22°. The cold-stress environment was PDA plates growing at 15°, whereas the fungicide-stress environment was PDA plates containing 0.75 ppm propiconazole (Syngenta, Basel, Switzerland) growing at 22°.

After inoculation, Petri plates were dried for 30 min in a laminar flow cabinet and sealed using Parafilm. Plates were then randomized in a growth chamber set to a constant temperature with 70% humidity and no light. Plates were photographed at 8, 11, and 14 days postinoculation (dpi) for image analysis. These time points were chosen because they represented different colony ages and provided the largest phenotypic variance in preliminary experiments. This experimental design resulted in nine unique Environment-Colony-Age-Melanization (ECAM) phenotypes for each isolate, which we will refer to as ECAM phenotypes.

**Figure 1:**
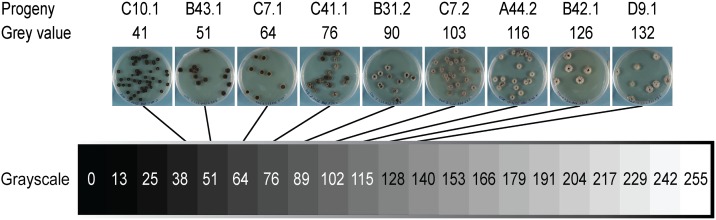
Colony melanization was measured by digital image analyses of grayscale values ranging from 0 (black) to 255 (white). Images from the control environment at 14 dpi for representative progeny from the cross 1A5 × 1E4 are positioned onto the scale to illustrate the different degrees of melanization observed in the crosses. Each image is labeled with the corresponding progeny name (top) and the corresponding gray value (bottom) as measured in the images. The gray values shown represent the full phenotypic variance found under the control environment at 14 dpi in the progeny from the cross 1A5 × 1E4.

Digital images were captured through the Petri dish lid using standardized camera settings and lighting environments (see Table S2, Table S3, and Figure S1). After images were acquired, Petri dishes were rerandomized and returned to the growth chamber for further colony growth and later image acquisition. Images were processed using a batch macro developed in the open-source software ImageJ (Schneider *et al.* 2012), enabling automated image analysis. The macro identified and scored individual colonies in the images (see File S1). Melanization was measured using average gray values of individual colonies. Gray values range from 0 to 255, with 0 representing black and 255 representing white (Figure 1). We used untransformed gray values as phenotypic values representing different degrees of colony melanization. We calculated broad-sense heritability (H^2^) values (Burton and Devane 1953) over means of the five technical repeats for each environment and colony age using a one-way analysis of variance model in R (R_Core_Team 2012). The melanization phenotypes for the retained progeny within each cross can be found in Table S4.

### QTL mapping

QTL mapping was performed in R/qtl version 1.27-10 (Arends *et al.* 2010) using single marker analysis combined with interval mapping, resulting in simple interval mapping (SIM) analysis. The analysis was based on progeny mean values, calculated over the five technical repeats for each environment and colony age, so that nine different ECAM phenotypes were generated for each cross (three environments × three colony ages). Interval mapping improves on the marker regression method by estimating markers (pseudomarkers) in between true markers. For SIM analysis we used the EM algorithm. Significance thresholds were determined by permutating the marker data. We applied genome-wide permutations, with 1000 permutations for each ECAM phenotype. We considered only QTL peaks with LOD scores that provided *P*-values lower then 0.05. We used the reference IPO323 genome to convert cM positions of markers into base pair (bp) positions on the reference genome. For 95% confidence interval calculations, we used Bayesian credible intervals from SIM combined with the EM algorithm (Manichaikul *et al.* 2006). Marker mean differences and the amount of variance explained by significant markers were calculated using true markers and not pseudomarkers. We assumed that our marker alleles were additive because we did not detect any significant epistasis.

### Identification of candidate genes within QTL confidence intervals

To identify candidate genes within QTL confidence intervals, we first identified sequence variants among the parents in each cross by comparing their genome sequences, which had been previously aligned against the IPO323 reference genome. SNPs associated with synonymous and nonsynonymous mutations were called using the GATK UnifiedGenotyper with a maximum alternative allele setting of 2. Filter settings in the GATK VariantFiltration were as follows: QD ≥ 5, FS ≤ 60, HaplotypeScore ≤ 10.0, QUAL ≥ 100, AFlower ≥ 0.2, AFupper ≤ 0.8, and AN ≥ 2. For all other sequence variants, we used default settings of the GATK tools (Depristo *et al.* 2011). SNP and other sequence variation annotation results among the parental strains within each cross were obtained using the open-source tools SnpEff and SnpSift version 3.3h (Cingolani *et al.* 2012). Synonymous SNPs were not considered in further analyses. Genes without Gene Ontology annotations were described as having unknown functions. Genes were considered as candidate genes within a confidence interval if they contained at least one sequence variant within the boundaries of the confidence interval, excluding genes with no sequence variation or with only synonymous SNPs.

A total of 29 genes involved in fungal melanin biosynthesis have been described in the literature. Orthologs (Table S5) for each of these genes were identified in *Z. tritici* by performing a BlastP search in the National Center for Biotechnology Information nonredundant protein database (http://www.ncbi.nlm.nih.gov) and conducting phylogenetic analyses of the amino acid sequences using the maximum likelihood algorithm with default parameters in MEGA5 (Tamura *et al.* 2011). All QTL confidence intervals were searched for presence of the identified orthologs, which were considered as candidate melanization genes.

RNA-Seq data were obtained for parent 3D7 as described previously (Brunner *et al.* 2013). In summary, RNA-Seq data were obtained during an in planta infection time course that included all major phases of the pathogen life cycle (*i.e.*, biotrophic, necrotrophic, and saprotrophic phases), including three biological repeats at 7, 13, and 56 dpi. Reads per kilobase per million per gene values were calculated by normalizing the RNA reads and mapping them onto the reference genome. Significant differences in transcript abundances were calculated as described previously (Brunner *et al.* 2013). Transcription profiles were obtained for the orthologs to genes involved in melanin biosynthesis as well as candidate genes identified within QTL confidence intervals.

### Amino acid differences in *PKS1* among parents 3D1 and 3D7

We identified synonymous and nonsynonymous substitutions in the gene *PKS1* (ProteinID 96592) encoding a polyketide synthase that catalyzes the first step of the DHN melanin biosynthetic pathway. Sequence polymorphism was scored in a Swiss field population of 25 *Z. tritici* isolates. Nine of the 25 isolates, including the four parental strains, had their genomes sequenced in a previous study (Croll *et al.* 2013), whereas complete genome sequences of 16 additional isolates (3A2, 3A4, 3A5, 3A6, 3A8, 3A10, 3B2, 3B4, 3C7, 3D3, 3D5, 3F1, 3F3, 3F4, 3G3, and 3H1) were obtained using the same methods for this study. SNPs among the isolates were called and filtered using the GATK tools (Depristo *et al.* 2011) with similar settings as applied for the RADseq dataset. Introns as well as synonymous and nonsynonymous SNPs were manually curated.

A BlastP search in the National Center for Biotechnology Information non-redundant protein database (http://www.ncbi.nlm.nih.gov) was performed to investigate sequence conservation among ascomycetes. The *PKS1* amino acid sequence of the *Z. tritici* reference genome isolate IPO323 was aligned against 10 ascomycetes, including *Pseudocercospora fijiensis*, *Cladosporium phlei*, *Elsinoe fawcettii*, *Leptosphaeria maculans*, *Alternaria alternata*, *Phaeosphaeria nodorum*, *Pyrenophora tritici-repentis*, *Magnaporthe oryzae*, *Neurospora crassa*, and *Aspergillus nidulans*. The Pfam database was used to identify functional *PKS1* protein domains (Finn *et al.* 2014).

## Results

### Genetic maps

We retained 263 unique progeny and 9745 SNP markers in the 3D1 × 3D7 cross. A total of 96 progeny were excluded because of missing genotypes (18) or clonality (78). The quality-filtered dataset in the 1A5 × 1E4 cross included 261 progeny and 7333 SNP markers; 80 progeny were excluded because of missing genotypes (14) or clonality (66). Table 1 and Table 2 summarize the results for each genetic map.

**Table 1:** Genetic map summary for the cross between *Zymoseptoria tritici* isolates 3D1 and 3D7

Chromosome	No. Markers	Average Marker Spacing, cM	Genetic Length Covered by Markers, cM	Physical Length Covered by Markers, kb	Ratio of Physical Distance to Genetic Distance, kb/cM	Percentage of Reference Genome Covered by Markers, %
1	1906	0.299	569.3	5885	10.34	97
2	1129	0.338	381.7	3755	9.84	97
3	987	0.364	358.6	3409	9.51	97
4	811	0.355	287.9	2816	9.78	98
5	553	0.775	427.8	2730	6.38	95
6	775	0.357	276.1	2533	9.17	95
7	429	0.555	237.6	2574	10.83	97
8	836	0.317	264.5	2347	8.87	96
9	543	0.465	252.1	2075	8.23	97
10	149	1.386	205.2	1487	7.24	88
11	252	0.759	190.4	1522	7.99	94
12	365	0.55	200.2	1361	6.8	93
13	400	0.723	288.5	1044	3.62	88
16	48	0.792	37.2	455	12.24	75
17	192	0.501	95.8	439	4.59	75
19	173	0.48	82.5	470	5.69	85
20	197	0.51	99.9	429	4.29	91
Total	9745	0.437	4255.4	35331	Average 7.97	Average 92

**Table 2:** Genetic map summary for the cross between *Zymoseptoria tritici* isolates 1A5 and 1E4

Chromosome	No. Markers	Average Marker Spacing, cM	Genetic Length Covered by Markers, cM	Physical Length Covered by Markers, kb	Ratio of Physical Distance to Genetic Distance, kb/cM	Percentage of Reference Genome Covered by Markers, %
1	1336	0.612	817.5	5813	7.11	95
2	743	0.59	437.8	3712	8.48	96
3	695	0.738	512.5	3419	6.67	98
4	494	0.796	392.5	2806	7.15	97
5	504	0.753	378.7	2657	7.02	93
6	468	0.769	359.2	2495	6.95	93
7	522	0.725	377.7	2574	6.82	97
8	528	0.591	311.6	2329	7.47	95
9	421	0.699	293.7	2056	7.00	96
10	366	0.711	259.4	1634	6.30	97
11	212	0.975	205.7	1521	7.40	94
12	95	0.743	69.8	515	7.38	35
13	263	1.038	272	1069	3.93	90
14	55	1.314	71	620	8.74	80
15	160	0.797	126.7	509	4.01	80
16	29	1.297	36.3	434	11.96	71
18	43	1.348	56.6	492	8.70	86
19	113	0.877	98.2	494	5.03	90
20	106	0.76	79.8	398	4.99	84
21	180	0.192	34.4	279	8.10	68
Total	7333	0.708	5191.3	35827	Average 7.06	Average 87

**Figure 2:**
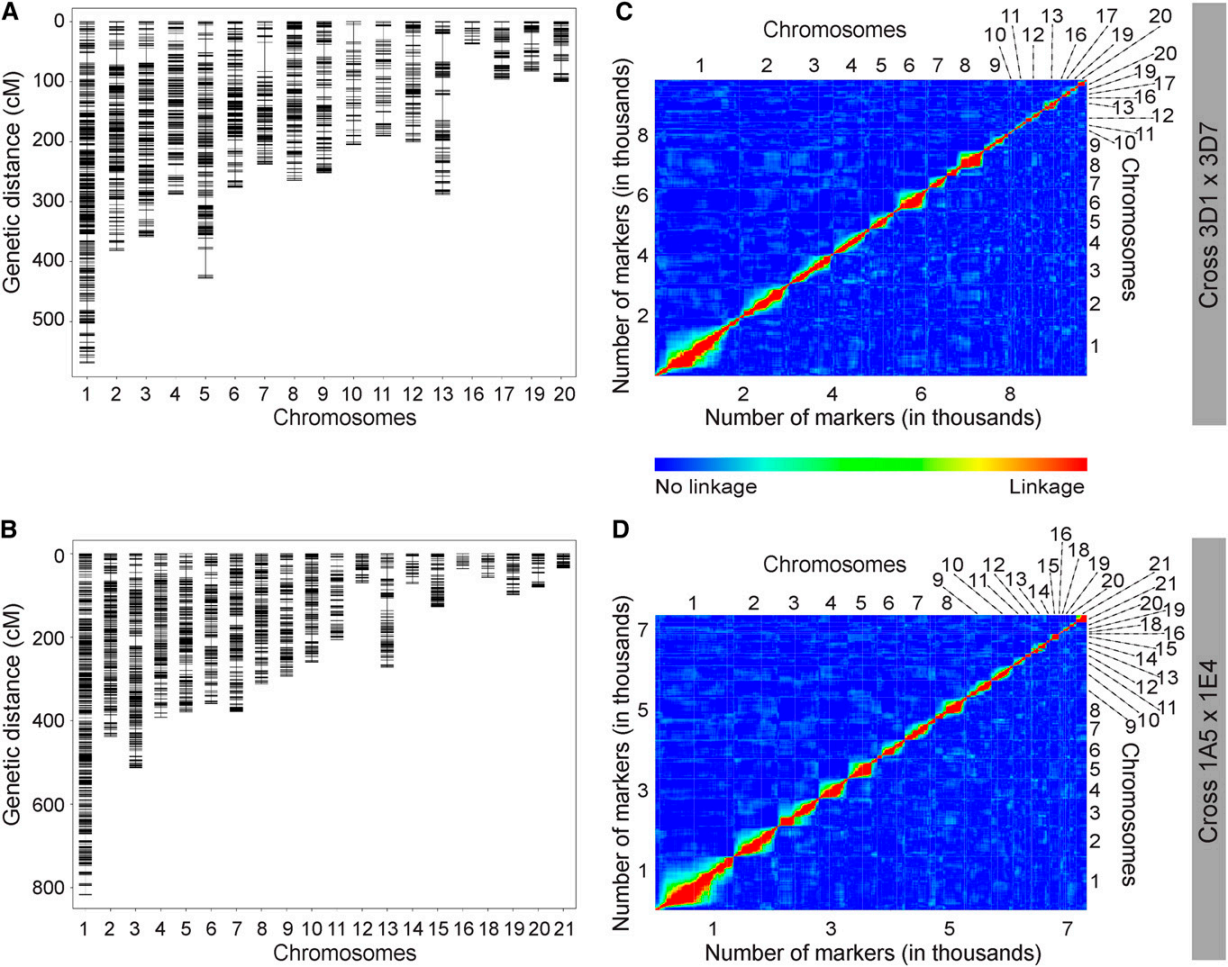
(A, B) Genetic maps and (C, D) pairwise linkage comparisons of markers for each cross (Top: 3D1 × 3D7; Bottom: 1A5 × 1E4). (C, D) Within the pairwise linkage comparison plot marker-pairwise recombination fractions are shown in the upper left triangle and LOD scores for tests of r = 1/2 are shown in the lower right triangle. Red corresponds to a large LOD or a small recombination fraction, while blue is the reverse. Thus red indicates linkage, while blue indicates no linkage.

Marker density was high with an average genetic spacing of 0.44 cM, representing an average physical spacing of ~3600 bp between SNP markers in cross 3D1 × 3D7 and average spacing of 0.71 cM (~4700 bp) in cross 1A5 × 1E4. On average, our genetic maps covered around 90% of the reference genome over both crosses. Figure 2 shows the genetic maps as well as plots of pairwise marker recombination fractions and LOD scores for tests of r = 0.5, which indicate a distorted diagonal red line for chromosome 13 in both crosses, indicating marker order inconsistencies. Chromosome 13 likely exhibits map inflation in both crosses, indicated by an unusual genetic length (Table 1 and Table 2) and larger gaps on the genetic map (Figure 2).

The eight smallest chromosomes (14 to 21) in the *Z. tritici* reference genome are accessory chromosomes (ACs) (Goodwin *et al.* 2011) that often exhibit presence/absence polymorphisms (Croll *et al.* 2013). In the 3D1 × 3D7 cross, the parental genome sequences revealed that ACs 14, 15, 18, and 21 were missing in one of the parents, hence only ACs 16, 17, 19, and 20 were mapped. In the 1A5 × 1E4 cross, AC 17 was missing in one of the parents and therefore only ACs 14, 15, 16, 18, 19, 20, and 21 were mapped.

### Melanization is a quantitative trait that shows transgressive segregation

For both crosses over all three environments and all three colony ages, we found that melanization showed a continuous distribution consistent with a quantitative character (Figure S2 and Figure S3). Many progeny had more extreme phenotypes than the parents, indicating transgressive segregation. The degree of melanization increased over time for most isolates in most environments, indicating that melanin concentration increases as colonies age.

Reaction norms over the three colony ages differed among the progeny in each cross, providing evidence for differential interactions between genotypes and environments over time. To illustrate this, we compared the two progeny showing the most extreme phenotypes at 14 dpi in one environment with their corresponding phenotypes in the other two environments (Figure S4 and Figure S5). In both crosses we found strong evidence for phenotype-by-environment interaction over all colony ages. Hence, we analyzed each colony age separately in the QTL analyses.

### Comparison of mapped QTL reveals unique and shared QTL between the two crosses

**Figure 3:**
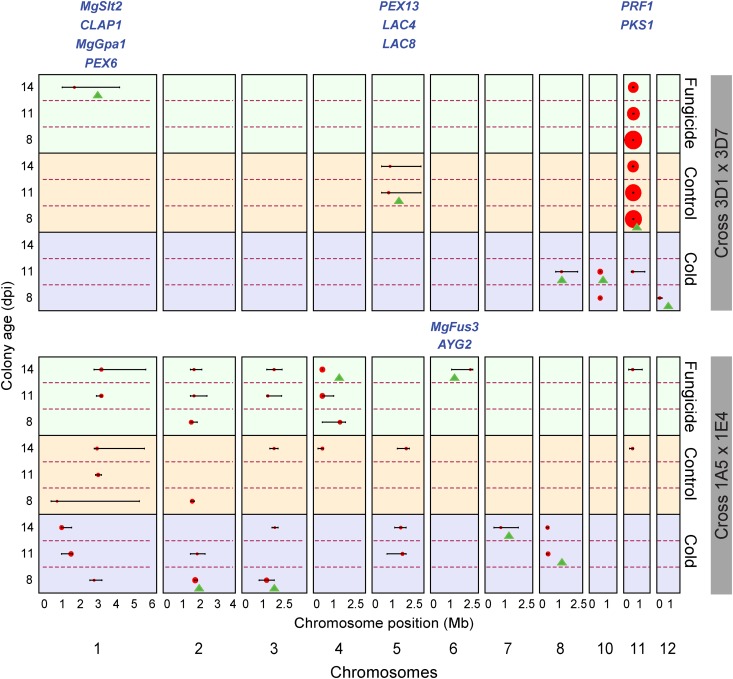
QTL peaks (red dots) and associated confidence intervals for all significant QTL detected in the experiment. Cross 3D1 × 3D7 is shown in the upper half and cross 1A5 × 1E4 is shown in the lower half of the figure. Environments are color-coded: green = fungicide stress environment; orange = control environment; purple = cold stress environment. Colony age increases within each environment from the bottom up. The different sizes of red dots represent the relative size of the associated LOD scores. The 12 cross-specific QTL are marked with a green triangle. Orthologs involved in melanin biosynthesis found within 12 cross-specific QTL confidence intervals as candidate genes are indicated with names in blue positioned above the corresponding chromosome. Chromosome sizes are presented in mega base pairs (Mb). QTL, quantitative trait loci; LOD, logarithm of odds.

We found six significant QTL in cross 3D1 × 3D7 and nine significant QTL in cross 1A5 × 1E4 (Figure 3, Table 3, and Table 4). QTL were considered to be different if the confidence intervals did not overlap. This approach was applied across environments and colony ages as well as within crosses and among crosses. Three QTL were shared between the two crosses. Shared QTL were further characterized based on the 3D1 × 3D7 cross. A detailed description of all significant QTL found in both crosses is given in Table S6, Table S7, Table S8, and Table S9. No QTL were found on ACs.

**Table 3:** Positions and effects of the six cross-specific QTL identified in cross 3D1 × 3D7

Environment	Colony Age, dpi	QTL Peak Marker*^a^*	Chromosome	Estimated Position of Peaking Marker, cM	Estimated Position of Peaking Marker, kb	LOD Score at Peak	*P* Value	Mean 3D1 Allele (Gray Value)	Mean 3D7 Allele (Gray Value)	Mean Difference	Allele Effect*^b^*	Percentage of Variance Explained by QTL, %	Estimated Position of Proximal Marker, kb*^c^*	Estimated Position of Distal Marker, kb*^c^*	Bayes Confidence Interval Length, kb
Cold	8	12_143193	12	5.74	143	4.85	0.002	134.56	129.28	5.27	3D1	9.2	29	305	276
Cold	11	8_1250811	8	140.32	1251	4.07	0.01	101.72	112.39	10.66	3D7	7.1	897	2228	1332
Cold	11	c10.loc108 (p)	10	108.13	649	9.55	< 0.001	116.28	100.17	16.11	3D1	15.6	634	673	39
Control	8	c11.loc82 (p)	11	82.06	581	32.2	< 0.001	118	93.73	24.27	3D1	39.2	560	603	43
Control	11	5_871061	5	157.26	871	4.38	0.003	74.99	85.47	10.48	3D7	7.5	449	2799	2350
Fungicide	14	1_1740191	1	193.5	1740	3.64	0.014	64.87	73.03	8.16	3D7	6.3	1063	4267	3203

LOD, logarithm of odds.

a A (p) indicates that a pseudomarker provided the greatest LOD score. In all other cases a true marker provided the highest LOD score.

b 3D7 indicates that the parental 3D7 allele provided the greater phenotypic mean then the parental 3D1 allele, whereas 3D1 indicates that the parental 3D1 allele provided the higher phenotypic mean then the parental 3D7 allele.

c Markers flanking Bayes confidence interval.

**Table 4:** Positions and effects of the six cross-specific QTL identified in cross 1A5 × 1E4

Environment	Colony Age, dpi	QTL Peak Marker*^a^*	Chromosome	Estimated Position of Peaking Marker, cM	Estimated Position of Peaking Marker, kb	LOD Score at Peak	*P* Value	Mean 1A5 Allele (Gray Values)	Mean 1E4 Allele (Gray Values)	Mean Difference	Allele Effect*^b^*	Percentage of Variance Explained by QTL, %	Estimated Position of Proximal Marker, kb*^c^*	Estimated Position of Distal Marker, kb*^c^*	Bayes Confidence Interval Length, kb
Cold	8	c3.loc208 (p)	3	208.04	1301	8.52	<0.001	133.71	138.79	5.08	1E4	12.1	869	1749	880
Cold	11	8_429575	8	58.42	430	7.65	<0.001	117.28	103.6	13.68	1A5	12.8	348	446	99
Cold	14	7_841106	7	181.47	841	4.01	0.014	103.42	93.3	10.13	1A5	7	428	1893	1464
Cold	8	c2.loc226 (p)	2	226.07	1699	10	<0.001	139.11	133.43	5.69	1A5	17	1646	1819	173
Fungicide	14	c4.loc54 (p)	4	54.04	420	9.47	<0.001	92.37	111.73	19.36	1E4	16.2	417	426	9
Fungicide	14	6_2299166	6	289.62	2299	3.56	0.027	108.33	96	12.33	1A5	6.2	1166	2438	1272

LOD, logarithm of odds.

a A (p) indicates that a pseudomarker provided the highest LOD score. In all other cases a true marker provided the highest LOD score.

b 1E4 indicates that the parental 1E4 allele provided the greater phenotypic mean then the parental 1A5 allele, whereas 1A5 indicates that the parental 1A5 allele provided the greater phenotypic mean then the parental 1E4 allele.

c Markers flanking Bayes confidence interval.

### Environment and colony age affect QTL mapping results

In both crosses we found environment-specific QTL as well as QTL that were shared across all environments. In cross 3D1 × 3D7, five QTL were environment-specific and a QTL on chromosome 11 was found in all environments. In cross 1A5 × 1E4, six QTL were environment-specific and three QTL were found in all environments (Figure 3).

Colony age also affects QTL detection for some environments in both crosses. For example, in cross 3D1 × 3D7 the chromosome 5 QTL was found in colonies that were 11 and 14 d old, whereas the chromosome 8 QTL was found only in colonies that were 11 d old. In the 1A5 × 1E4 cross, the QTL on chromosomes 5 and 8 were significant in colonies that were 11 and 14 d old, but the QTL on chromosomes 6, 7 and 11 were found only in colonies that were 14 d old (Figure 3).

### Genetic architecture of melanization in the two crosses

We found differences in the genetic architecture of melanization among the two crosses. On average, cross 3D1 × 3D7 had fewer (1.8) melanization QTL for each colony age and environment than cross 1A5 × 1E4 (3.6); however, the average total variance explained by the QTL for each ECAM phenotype was similar (~35%) in both crosses. In cross 3D1 × 3D7 nearly half of the total phenotypic variance was explained by one QTL on chromosome 11, whereas in cross 1A5 × 1E4 a similar amount of variance was explained by six QTL distributed across several chromosomes, suggesting a more complex genetic basis of melanin production in cross 1A5 × 1E4 compared with cross 3D1 × 3D7 (Table 5).

**Table 5:** Phenotypic contributions and distribution of all significant QTL

Cross*^a^*	Environment	Colony Age, dpi	Chromosomes With QTL	Allele Effect*^b^*	Total Percentage of Variance Explained by QTL(s), %	Number of Significant QTL
3D1 × 3D7 °	Cold	8	10, 12	3D1(2)	22.8	2
3D1 × 3D7 °	Cold	11	8, 10, 11	3D1(2), 3D7(1)	30.1	3
3D1 × 3D7	Control	8	11	3D1(1)	39.2	1
3D1 × 3D7 °	Control	11	5, 11	3D1(1), 3D7(1)	45.7	2
3D1 × 3D7 °	Control	14	5, 11	3D1(1), 3D7(1)	36.5	2
3D1 × 3D7	Fungicide	8	11	3D1(1)	45.8	1
3D1 × 3D7	Fungicide	11	11	3D1(1)	30.4	1
3D1 × 3D7 °	Fungicide	14	1, 2	3D1(1), 3D7(1)	33.5	2
Average					35.5	1.8
1A5 × 1E4 °	Cold	8	1, 2, 3	1A5(1), 1E4(2)	35.4	3
1A5 × 1E4 °	Cold	11	1, 2, 5, 8	1A5(3), 1E4(1)	43.6	4
1A5 × 1E4 °	Cold	14	1, 3, 5, 7, 8	1A5(4), 1E4(1)	45.9	5
1A5 × 1E4 °	Control	8	1, 2	1A5(2)	18.1	2
1A5 × 1E4	Control	11	1	1E4(1)	10.9	1
1A5 × 1E4 °	Control	14	1, 3, 4, 5, 11	1A5(1), 1E4(4)	42.4	5
1A5 × 1E4 °	Fungicide	8	2, 4	1A5(1), 1E4(1)	25.2	2
1A5 × 1E4 °	Fungicide	11	1, 2, 3, 4	1A5(1), 1E4(3)	40.6	4
1A5 × 1E4 °	Fungicide	14	1, 2, 3, 4, 6, 11	1A5(3), 1E4(3)	55.5	6
Average					35.3	3.6

QTL, quantitative trait loci; dpi, days postinoculation; ECAM, Environment-Colony-Age-Melanization.

a A ° refers to ECAM phenotypes with at least two significant QTL, whereas no ° refers to ECAM phenotypes with only one significant QTL.

b 3D7/1E4 indicates that the parental 3D7/1E4 allele provided the greater phenotypic mean contribution then the parental 3D1/1A5 allele, whereas 3D1/1A5 indicates that the parental 3D1/1A5 allele provided the greater phenotypic mean contribution then the parental 3D7/1E4 allele. The number within brackets following the parental allele indicates the number of significant QTL.

### Identification of candidate genes and orthologous genes related to melanization in QTL regions

A summary of the candidate genes found within each confidence interval for cross 3D1 × 3D7 and 1A5 × 1E4 is given in Table S8 and Table S9, respectively. On average we found ~190 candidate genes per confidence interval, with a minimum of one candidate gene (14 dpi, fungicide environment, 1A5 × 1E4) and a maximum of 1245 genes (8 dpi, control environment, 1A5 × 1E4). Approximately 32% of the candidate genes found in a confidence interval had no known function and ~42% showed significant changes in transcript abundance across biotrophic, necrotrophic, and saprotrophic stages in the life cycle. Eleven orthologs of the 29 genes described to be involved in melanin biosynthesis (Table S5) were identified as candidate genes in four of the 12 cross-specific QTL confidence intervals (Figure 3). Nine of the 29 orthologs were found in three of the QTL in cross 3D1 × 3D7, and two were found in one of the QTL in cross 1A5 × 1E4 (Table 6). These 11 orthologs encode two (*MgSlt2* and *MgFus3*) mitogen-activated protein kinases, one (*CLAP1*) copper-transporting ATPase, two (*LAC4* and *LAC8*) laccases, one (*PRF1*) prefolding chaperone, one (*PKS1*) polyketide synthase, one (*MgGpa1*) G alpha protein, as well as two (*PEX6* and *PEX13*) genes involved in peroxisome biosynthesis and one (*AYG2*) of an unknown function (Table S5). Three (*MgSlt2*, *MgFus3* and *MgGpa1*) of these 11 genes were associated with melanization in *Z. tritici* in earlier studies (Cousin *et al.* 2006; Mehrabi *et al.* 2006a, 2009), but the other eight genes have not previously been associated with melanization in *Z. tritici*.

**Table 6:** Genes associated with melanization that were identified as candidate genes within the six cross-specific QTL in each cross

Cross	Environment	Colony Age, dpi	LOD Score at Peak	Chromosome	Estimated Position of Peaking Marker, kb	Estimated Position of Proximal Marker, kb*^a^*	Estimated Position of Distal Marker, kb*^a^*	Orthologs to Genes Involved in Melanin Biosynthesis*^b^*
3D1 × 3D7	Cold	8	4.85	12	143	29	305	/
3D1 × 3D7	Cold	11	4.07	8	1251	897	2228	/
3D1 × 3D7	Cold	11	9.55	10	649	634	673	/
3D1 × 3D7	Control	8	32.2	11	581	560	603	*PRF1*, *PKS1*
3D1 × 3D7	Control	11	4.38	5	871	449	2799	*PEX13*, *LAC4*, *LAC8*
3D1 × 3D7	Fungicide	14	3.64	1	1740	1063	4267	*MgSlt2*, *CLAP1*, *MgGpa1*, *PEX6*
1A5 × 1E4	Cold	8	8.52	3	1301	869	1749	/
1A5 × 1E4	Cold	11	7.65	8	430	348	446	/
1A5 × 1E4	Cold	14	4.01	7	841	428	1893	/
1A5 × 1E4	Cold	8	10	2	1699	1646	1819	/
1A5 × 1E4	Fungicide	14	9.47	4	420	417	426	/
1A5 × 1E4	Fungicide	14	3.56	6	2299	1166	2438	*MgFus3*, *AYG2*

QTL, quantitative trait loci; dpi, days postinoculation; LOD, logarithm of odds.

a Markers flanking Bayes confidence interval.

b Orthologs to genes involved in melanin biosynthesis found as candidate genes in Bayes confidence interval.

**Figure 4:**
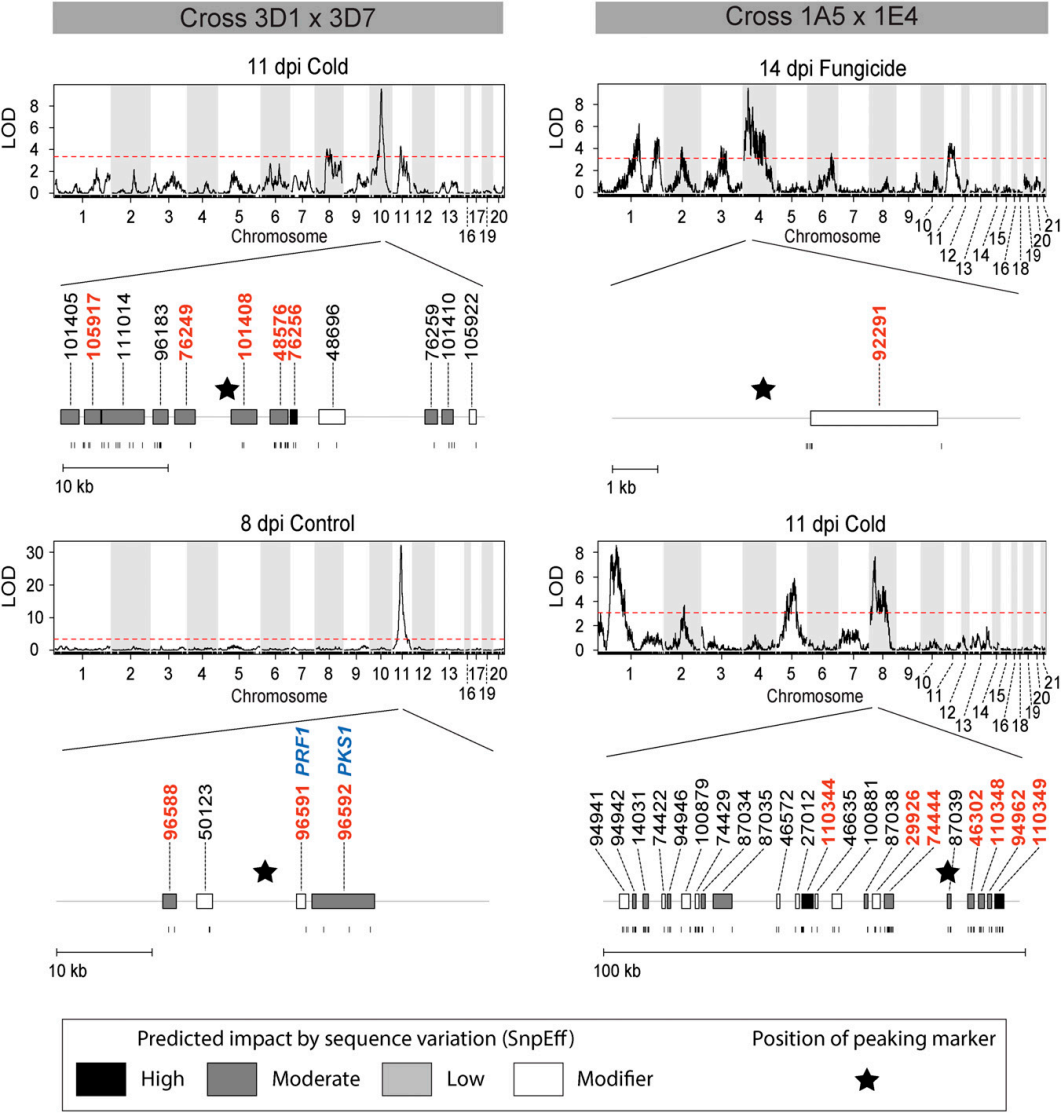
LOD plots from the single marker interval mapping analysis over all chromosomes for the phenotypes that provided the peaks with confidence interval containing the smallest number (≤ 30) of candidate genes. The horizontal dashed red line in the LOD plots represents the significance threshold (*P* = 0.05) obtained by 1000 genome-wide permutations. Below each LOD plot is the confidence interval (gray line) for the peak with the fewest candidate genes (gene bars), which are positioned within the confidence interval. The genes are color coded according to the predicted impact by SnpEff of the observed sequence variation [white = modifier, light gray = low (no case within the confidence interval shown), gray = moderate, black = high] and labeled with their protein ID just above the gene bars. Below the gene bars are vertical lines, representing the position of each sequence variant within a gene. The asterisk symbol represents the position of the peaking marker within the confidence interval. Orthologs to genes involved in melanin biosynthesis found as candidate genes within the confidence interval are represented by their name in blue, just above the corresponding protein ID. High-priority candidate genes are colored in red.

To narrow the search for candidate genes affecting melanization, we focused on major QTL (LOD > 7.5) containing ≤30 candidate genes in their confidence intervals. We identified two confidence intervals meeting these criteria in each cross (Table 7). For cross 3D1 × 3D7, the QTL on chromosome 10 for the 11 dpi cold environment had 12 candidate genes and the QTL on chromosome 11 for the 8 dpi control environment had four candidate genes (Table S10 and Figure 4). For cross 1A5 × 1E4, the QTL on chromosome 4 for the 14 dpi fungicide environment had one candidate gene and the QTL on chromosome 8 for the 11 dpi cold environment had 22 candidate genes (Table S11 and Figure 4). Taking into account all of the available information (gene ontology, the predicted impact of observed sequence variation, the position of the QTL peak, gene expression) as shown in Table S10 and Table S11, we further narrowed the list down to eight high-priority candidate genes in the 3D1 × 3D7 cross and eight high-priority candidate genes in the 1A5 × 1E4 cross, resulting in a total of 16 high-priority candidate genes over both crosses.

**Table 7:** Large-effect QTL intervals containing ≤30 candidate genes

Cross	Environment	Colony Age, dpi	Chromosome	Estimated Position of Peaking Marker, kb	LOD Score at Peak	*P* Value	Estimated Position of Proximal Marker, kb^a^	Estimated Position of Distal Marker Flanking, kb*^a^*	Bayes Confidence Interval Length, kb	Number of Sequence Variations*^b^*	Number of Genes *^b^*	Number of Genes Affected by Sequence Variations*^b^*	Percentage of Total Genes Affected by Sequence Variations, %^b^	Number of Sequence Variation Affected Genes With Unknown Function*^b^*	Percentage of Total Sequence Variation Affected Genes With Unknown Function, %*^b^*
3D1 × 3D7	Cold	11	10	649	9.55	<0.001	634	673	39	59	15	12	80	2	17
3D1 × 3D7	Control	8	11	581	32.2	<0.001	560	603	43	8	14	4	29	0	0
1A5 × 1E4	Fungicide	14	4	420	9.47	<0.001	417	426	9	7	2	1	50	0	0
1A5 × 1E4	Cold	11	8	430	7.65	<0.001	348	446	99	132	27	22	81	9	41

QTL, quantitative trait loci; LOD, logarithm of odds.

a Markers flanking Bayes confidence interval.

b Numbers refer to within Bayes confidence interval.

## Discussion

### Genetic maps

The two genetic maps have very high marker density compared with other reported genetic maps in filamentous fungi (Foulongne-Oriol 2012). The high marker density reflects the high degree of recombination in the mapping populations as well as the large number of markers provided by our genotyping method. Earlier genetic maps based on the genome reference isolate IPO323 (Kema *et al.* 2002; Wittenberg *et al.* 2009) were ~72% smaller than our genetic maps. We hypothesize that our maps are larger due to greater recombination resulting from larger numbers of offspring and more extensive chromosome coverage resulting from larger numbers of genetic markers. Our maps covered ~90% of the reference genome on average. Incomplete coverage can be explained by missing restriction sites or low quality mapping reads toward one or both telomeres of each chromosome. Other explanations include a lack of recombining polymorphic markers or lower polymorphism toward the telomeres. The ratio of physical distance to genetic distance was ~7.5 kb/cM for both crosses, comparable with the ratio of 8.1 kb/cM described in *Aspergillus nidulans* (Christians *et al.* 2011).

The unusual genetic length (Table 1 and Table 2) and larger gaps on the genetic map of chromosome 13 (Figure 2) are consistent with map inflation resulting from incorrect marker order, most likely due to inappropriate alignment of our illumina reads onto the reference genome. We hypothesize that this could be due to the presence of the mating type idiomorphs (Waalwijk *et al.* 2002) on this chromosome, the occurrence of transposable elements or because of incorrect reference genome assembly. As chromosome 13 coverage was good (88% in cross 3D1 × 3D7 and 90% for cross 1A5 × 1E4) and no QTL were found on this chromosome for either cross, this issue was not investigated further.

### Melanization is a quantitative trait that is affected by environment and colony age

The finding that all ECAM phenotypes showed a continuous distribution (Figure S2 and Figure S3) and high broad-sense heritability (74–95%; Figure S4 and Figure S5) in both crosses indicates that several genes are likely to contribute to the melanization phenotype in *Z. tritici*. We found 15 significant QTL in total, with more than one significant QTL identified for 13 out of 18 ECAM phenotypes (Table 5). On average, a QTL contributed ~15% of the phenotypic variance observed in each cross. The pattern of transgressive segregation is also consistent with the hypothesis that melanization is a quantitative trait. Our findings stand in contrast to an earlier melanization mapping study (Lin *et al.* 2006) that identified only one significant QTL associated with several melanin phenotypes in a mapping population.

Our experimental design allowed us to investigate the effects of both colony age and environment on the melanization phenotype. We were able to demonstrate both colony age and environmental effects, as reflected by unique QTL, within both mapping populations. These findings are consistent with the hypothesis that genes affecting melanization are differentially regulated in different environments and at different stages of colony development.

### Unique QTL and shared QTL were identified by comparing QTL of the two crosses

We identified three shared QTL and nine QTL that were unique to one of the crosses. For the three shared QTL (found on chromosomes 1, 5, and 11), we compared the candidate genes found in the corresponding confidence intervals and found that more than 40% of the candidate genes in these confidence intervals were shared among the two crosses, suggesting that the shared QTL are likely to be due to same source of genetic variance in each cross. Among the 15 identified QTL, we postulate that the six QTL in Table 8 are the most likely to be replicated in other QTL mapping studies, based on having a combination of high LOD scores (≥ 7) and being found at least twice over different colony ages and/or environments.

**Table 8:** Most reproducible QTL in the two crosses

Cross	Environment	Colony Age, dpi	Chromosome	LOD Score at Peak	*P* Value	Number of Peaks Confirming the QTL of a Possible 9
3D1 × 3D7	Cold	11	10	9.55	<0.001	2
3D1 × 3D7	Control	8	11	32.2	<0.001	7
1A5 × 1E4	Cold	8	3	8.52	<0.001	5
1A5 × 1E4	Cold	11	8	7.65	<0.001	2
1A5 × 1E4	Cold	8	2	10	<0.001	6
1A5 × 1E4	Fungicide	14	4	9.47	<0.001	4

QTL, quantitative trait loci; dpi, days postinoculation; LOD, logarithm of odds.

### Identification of orthologs to genes involved in melanin biosynthesis within QTL intervals

All the previously identified orthologs of genes involved in melanin biosynthesis (Table S5) were expressed (≥ 2 reads per kilobase per million), hence all of the orthologs could affect melanization in *Z. tritici*. Eleven of the 29 orthologs were found within four of the 12 cross-specific QTL confidence intervals (Table 6 and Figure 3) identified in our analyses. We postulate that several of these known genes contributed to the melanization phenotypes in our crosses. Among the 11 orthologous candidates, three [*MgSlt2* (Mehrabi *et al.* 2006a), *MgGpa1* (Solomon *et al.* 2004; Mehrabi *et al.* 2009) and *MgFus3* (Cousin *et al.* 2006)] were functionally validated in *Z. tritici*, but none of these have been investigated as contributing to a quantitative character. The other eight candidates have not yet been analyzed in *Z. tritici*, but our QTL analyses indicate that these genes may also affect melanization in *Z. tritici*. After gene disruption, eight of these 11 orthologous candidates exhibited a measurable change in mycelial melanization *in vitro*, and three (*PEX6*, *LAC4*, and *LAC8*) affected melanization of appressoria but not mycelia in Colletotrichum spp (Kimura *et al.* 2001; Lin *et al.* 2012). None of the 18 remaining orthologs was found as candidate genes in the 12 cross-specific QTL confidence intervals.

### QTL confidence intervals contain both novel and known candidate genes affecting melanization

Three of the 16 high-priority candidate genes carried no Gene Ontology annotation and thus have no known function. These candidates could represent novel transcription factors, enzymes, or structural proteins that affect the melanization phenotype. The remaining 13 genes had Gene Ontology annotations. Seven of the 16 high-priority candidate genes showed significant changes in transcript abundance across biotrophic, necrotrophic, and saprotrophic stages of the life cycle, consistent with expected changes in melanin production across the pathogen life cycle. Melanin production is expected to increase during the development of the black pycnidia that form early in the saprotrophic stage of the life cycle (Eyal *et al.* 1987).

Within three of the four major-effect QTL confidence intervals, none of the candidate genes is an ortholog of genes known to be involved in melanin biosynthesis; thus, these represent novel candidates for genes affecting fungal melanization. Gene 92291 encodes a transcription factor and was the only candidate gene within the QTL confidence interval on chromosome 4. We hypothesize that this transcription factor regulates genes under fungicide stress because this QTL was found only in the fungicide environment.

The major-effect QTL on chromosome 11 contains three high-priority candidate genes and had the greatest LOD score (32.2) among the 12 cross specific QTL over both crosses (Table 3 and Table 4). Two of the three genes were orthologous to genes known to be involved in melanin biosynthesis (Table S5), namely *PRF1* (Protein ID: 96591) and *PKS1* (Protein ID: 96592). The third gene (96588) is involved in zinc binding and also was considered a high-priority candidate because the melanin biosynthesis ortholog *CMR1* (Eliahu *et al.* 2007) (Table S5) encodes a transcription factor with a zinc finger (Tsuji *et al.* 2000) and is located 28 kb upstream from 96588. We hypothesize that gene 96588 is involved in zinc homeostasis of *CMR1* and ultimately affects melanin biosynthesis; however, our analyses of sequence diversity led us to conclude that *PKS1* is the major contributor to the phenotypic variance explained by the large effect QTL on chromosome 11.

### Identification of a candidate quantitative trait nucleotide in *PKS1*

*PKS1* is the polyketide synthase enzyme catalyzing the first step of DHN melanin synthesis through head-to-tail joining and cyclization of acetate molecules (Takano *et al.* 1995; Butler and Day 1998) in the DHN melanin biosynthetic pathway. *PKS1* carries three nonsynonymous mutations among parents 3D1 and 3D7 while *PRF1* and 96588 contain 1 and 0 nonsynonymous mutations, respectively (Table S10). The region on chromosome 11 containing *PKS1* comprises the main DHN melanin biosynthetic gene cluster. We interpret this finding as a validation of our QTL mapping approach to identify genes affecting melanization. DHN melanin, which is the best characterized melanin in fungi (Butler and Day 1998), has so far not been studied in *Z. tritici* (Table S5). The identification of *PKS1* as a highly probable candidate gene within the QTL having the largest LOD score among the 12 cross-specific QTL suggests that DHN melanin plays a major role in melanization in *Z. tritici*.

**Figure 5:**
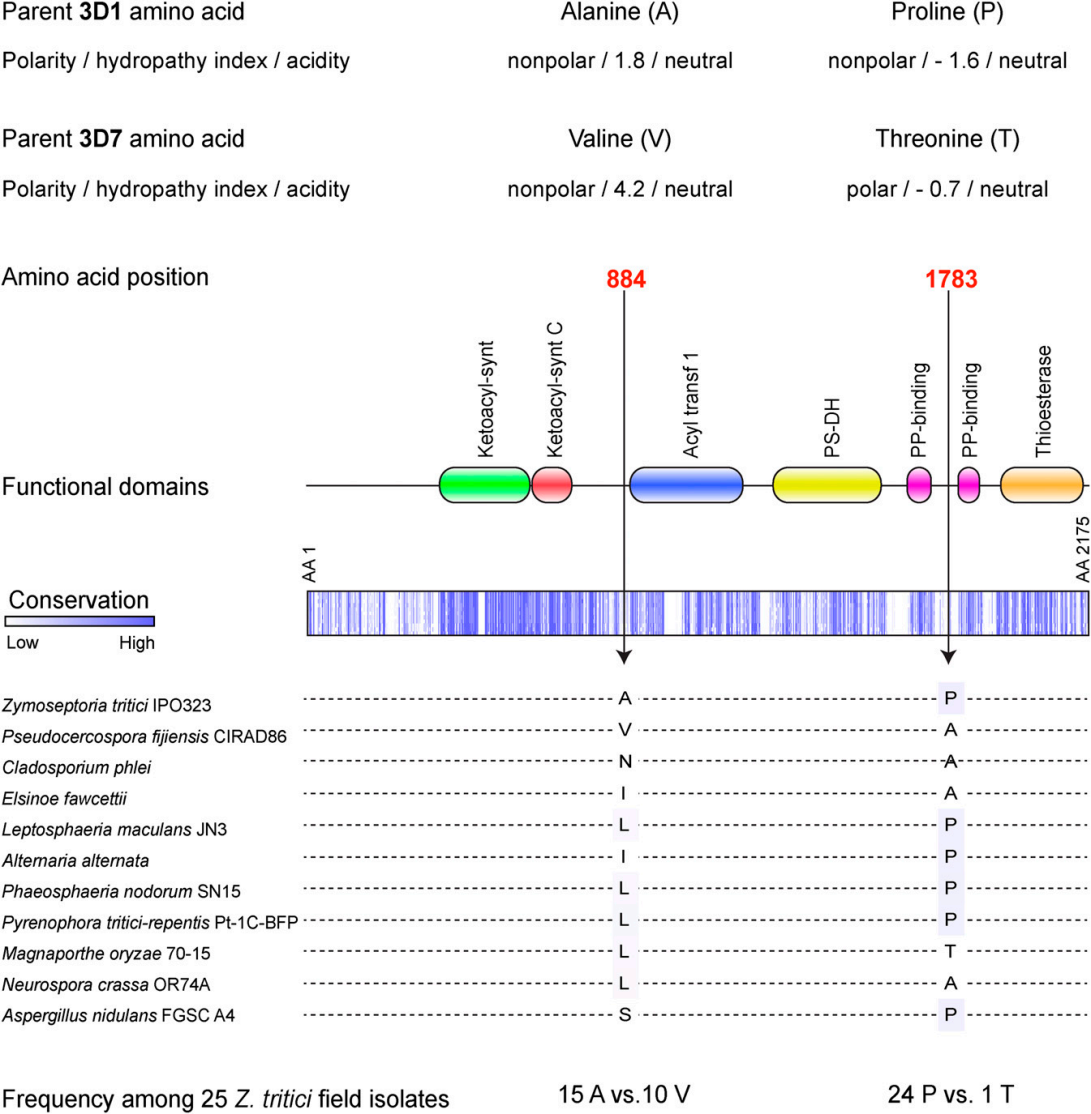
*PKS1* amino acid alterations between parent 3D1 and 3D7 and a schematic diagram of the entire *PKS1* gene (2175 amino acids). The two investigated amino acid alterations at position 884 and 1783 are indicated on the gene, showing their positions relative to the predicted functional domain sites. The lower panel shows polymorphisms present among 11 ascomycetes as well as 25 genetically distinct *Z. tritici* field isolates from Switzerland.

Three nonsynonymous substitutions at amino acid positions 155, 884, and 1783 were found in *PKS1* among parents 3D1 and 3D7, but positions 884 and 1783 (Figure 5) were considered as more likely candidates to explain differences in melanization because position 155 was not in a functional domain and the alternative amino acids did not differ for polarity, acidity (Taylor 1986) or hydropathy index (Kyte and Doolittle 1982). Fifteen of the 25 field isolates had an alanine at position 884 while 10 isolates carried a valine at that position. At position 1783, only the 3D7 parent had a threonine residue while the other 24 isolates had a proline (Figure 5). Position 1783 was more conserved among ascoymcetes than site 884, but neither site was located within an annotated functional domain of *PKS1* (Figure 5). The amino acid property changes were most striking for site 1783, because proline is nonpolar while threonine is polar. In addition, proline is unique among the 20 proteinogenic amino acids because its side group links to the amino group, often resulting in strong effects on protein secondary structure.

Based on these analyses of *PKS1* sequence polymorphism, we postulate that the nonsynonymous mutation found at amino acid 1783 explains most of the phenotypic variance associated with the large-effect QTL on chromosome 11. If this is confirmed, then the associated SNP represents a quantitative trait nucleotide (Fridman *et al.* 2004) that explains the majority of the phenotypic variance in this cross. Functional validation will be needed to confirm this hypothesis.

## Supplementary Material

Supporting Information
