# Supplementary material for: Quantitative Trait Locus Mapping of Melanization in the Plant Pathogenic Fungus Zymoseptoria tritici
Source: G3 (Bethesda). 2014 Oct 29;4(12):2519–33. doi: 10.1534/g3.114.015289 (PMC4267946; doi:10.1534/g3.114.015289)
Supplement: Supporting Information [file supp_4_12_2519__index.html]

Quantitative Trait Locus Mapping of Melanization in the Plant Pathogenic Fungus Zymoseptoria tritici — Supporting Information 

# Quantitative Trait Locus Mapping of Melanization in the Plant Pathogenic Fungus *Zymoseptoria tritici*

## Supporting Information for Lendenmann *et al.*, 2014

**Files in this Data Supplement:**

- Supporting Information - Tables S1-S11, Figures S1-S5, and File S1 (PDF, 657 KB)
- Table S1 - BioProject and sample accession numbers for the quality filter retained progeny used in the QTL analysis for each of the two crosses. (PDF, 155 KB)
- Table S2 - Camera and light setup overview. (PDF, 141 KB)
- Table S3 - Detailed information on the camera and lens used during image taking, as well as detailed information regarding the camera settings. (PDF, 142 KB)
- Table S4 - Melanization phenotypes (grey values) for the quality filter retained progeny used in the QTL analysis for each of the two crosses. (PDF, 226 KB)
- Table S5 - Orthologs in *Z. tritici* to genes involved in melanin biosynthesis. (PDF, 206 KB)
- Table S6 - Summary of QTL positions and effects for cross 3D1 x 3D7. (PDF, 143 KB)
- Table S7 - Summary of QTL positions and effects for cross 1A5 x 1E4. (PDF, 146 KB)
- Table S8 - Summary of genes affected by sequence variation within each Bayes confidence interval for cross 3D1 x 3D7, excluding all genes containing no sequence variation or with synonymous SNPs only. (PDF, 143 KB)
- Table S9 - Summary of genes affected by sequence variation within each Bayes confidence interval for cross 1A5 x 1E4 excluding all genes containing no sequence variation or with synonymous SNPs only. (PDF, 145 KB)
- Table S10 - Genes within large-effect QTL regions (confidence intervals containing ≤ 30 candidate genes) for cross 3D1 x 3D7, excluding genes with no sequence variation or with only synonymous SNPs. (PDF, 155 KB)
- Table S11 - Genes within large-effect QTL regions (confidence intervals containing ≤ 30 candidate genes) for cross 1A5 x 1E4, excluding genes with no sequence variation or with only synonymous SNPs. (PDF, 159 KB)
- Figure S1 - Camera and light setup overview. (PDF, 184 KB)
- Figure S2 - Melanization measured in the cross 3D1 x 3D7 over the three different environments. (PDF, 169 KB)
- Figure S3 - Melanization measured in the cross 1A5 x 1E4 over the three different environments. (PDF, 167 KB)
- Figure S4 - Norms of reaction for the cross 3D1 x 3D7 across the three colony ages and environments. (PDF, 225 KB)
- Figure S5 - Norms of reaction for the cross 1A5 x1E4 across the three colony ages and environments. (PDF, 243 KB)
- File S1 - Fiji Batch Macro (PDF, 155 KB)
