# Supplementary material for: Quantitative Trait Locus Mapping of Melanization in the Plant Pathogenic Fungus Zymoseptoria tritici
Source: G3 (Bethesda). 2014 Oct 29;4(12):2519–33. doi: 10.1534/g3.114.015289 (PMC4267946; doi:10.1534/g3.114.015289)
Supplement: Supporting Information [file supp_g3.114.015289_015289SI.pdf]

G3·Genes | Genomes | Genetics

Quantitative Trait Locus Mapping of Melanization in the Plant Pathogenic Fungus *Zymoseptoria tritici*

Mark H. Lendenmann*, Daniel Croll*, Ethan L. Stewart* and Bruce A. McDonald*

*Institute of Integrative Biology, Plant Pathology, ETH Zürich, 8092 Zürich, Switzerland

DOI: 10.1534/g3.114.015289

Table S1 BioProject and sample accession numbers for the quality filter retained progeny used in the QTL analysis for each of the two crosses.

Cross	Retained progeny for QTL analysis	NCBI BioProject accession	NCBI SRA sample accession
3D1 x 3D7	1.1	PRJNA256988	SRS670337
3D1 x 3D7	1.2	PRJNA256988	SRS670339
3D1 x 3D7	10.1	PRJNA256988	SRS670338
3D1 x 3D7	100.1	PRJNA256988	SRS670340
3D1 x 3D7	100.2	PRJNA256988	SRS670342
3D1 x 3D7	101.2	PRJNA256988	SRS670344
3D1 x 3D7	102.1	PRJNA256988	SRS670345
3D1 x 3D7	102.2	PRJNA256988	SRS670346
3D1 x 3D7	103.1	PRJNA256988	SRS670347
3D1 x 3D7	104.1	PRJNA256988	SRS670349
3D1 x 3D7	104.2	PRJNA256988	SRS670350
3D1 x 3D7	105.1	PRJNA256988	SRS670351
3D1 x 3D7	105.2	PRJNA256988	SRS670352
3D1 x 3D7	106.1	PRJNA256988	SRS670353
3D1 x 3D7	106.2	PRJNA256988	SRS670355
3D1 x 3D7	107.1	PRJNA256988	SRS670354
3D1 x 3D7	108.1	PRJNA256988	SRS670356
3D1 x 3D7	108.2	PRJNA256988	SRS670358
3D1 x 3D7	109.1	PRJNA256988	SRS670359
3D1 x 3D7	109.2	PRJNA256988	SRS670360
3D1 x 3D7	11.1	PRJNA256988	SRS670361
3D1 x 3D7	11.2	PRJNA256988	SRS670362
3D1 x 3D7	110.1	PRJNA256988	SRS670363
3D1 x 3D7	110.2	PRJNA256988	SRS670365
3D1 x 3D7	111.2	PRJNA256988	SRS670367
3D1 x 3D7	112.1	PRJNA256988	SRS670366
3D1 x 3D7	113.1	PRJNA256988	SRS670369
3D1 x 3D7	113.2	PRJNA256988	SRS670370
3D1 x 3D7	114.1	PRJNA256988	SRS670371
3D1 x 3D7	114.2	PRJNA256988	SRS670372
3D1 x 3D7	115.1	PRJNA256988	SRS670373
3D1 x 3D7	116.1	PRJNA256988	SRS670375
3D1 x 3D7	116.2	PRJNA256988	SRS670376
3D1 x 3D7	117.1	PRJNA256988	SRS670378
3D1 x 3D7	117.2	PRJNA256988	SRS670377
3D1 x 3D7	118.1	PRJNA256988	SRS670380
3D1 x 3D7	118.2	PRJNA256988	SRS670379
3D1 x 3D7	119.1	PRJNA256988	SRS670381
3D1 x 3D7	119.2	PRJNA256988	SRS670382

3D1 x 3D7	12.1	PRJNA256988	SRS670383
3D1 x 3D7	120.1	PRJNA256988	SRS670385
3D1 x 3D7	120.2	PRJNA256988	SRS670386
3D1 x 3D7	121.2	PRJNA256988	SRS670388
3D1 x 3D7	122.1	PRJNA256988	SRS670389
3D1 x 3D7	123.1	PRJNA256988	SRS670391
3D1 x 3D7	125.1	PRJNA256988	SRS670395
3D1 x 3D7	126.1	PRJNA256988	SRS670397
3D1 x 3D7	127.1	PRJNA256988	SRS670398
3D1 x 3D7	127.2	PRJNA256988	SRS670399
3D1 x 3D7	128.1	PRJNA256988	SRS670400
3D1 x 3D7	128.2	PRJNA256988	SRS670401
3D1 x 3D7	129.2	PRJNA256988	SRS670403
3D1 x 3D7	13.1	PRJNA256988	SRS670404
3D1 x 3D7	130.1	PRJNA256988	SRS670406
3D1 x 3D7	131.1	PRJNA256988	SRS670408
3D1 x 3D7	133.1	PRJNA256988	SRS670413
3D1 x 3D7	134.1	PRJNA256988	SRS670414
3D1 x 3D7	135.2	PRJNA256988	SRS670418
3D1 x 3D7	136.1	PRJNA256988	SRS670417
3D1 x 3D7	137.1	PRJNA256988	SRS670419
3D1 x 3D7	137.2	PRJNA256988	SRS670420
3D1 x 3D7	138.1	PRJNA256988	SRS670421
3D1 x 3D7	139.2	PRJNA256988	SRS670423
3D1 x 3D7	14.1	PRJNA256988	SRS670425
3D1 x 3D7	141.1	PRJNA256988	SRS670429
3D1 x 3D7	142.1	PRJNA256988	SRS670430
3D1 x 3D7	142.2	PRJNA256988	SRS670431
3D1 x 3D7	143.1	PRJNA256988	SRS670432
3D1 x 3D7	144.1	PRJNA256988	SRS670434
3D1 x 3D7	145.1	PRJNA256988	SRS670437
3D1 x 3D7	145.2	PRJNA256988	SRS670436
3D1 x 3D7	146.1	PRJNA256988	SRS670438
3D1 x 3D7	146.2	PRJNA256988	SRS670440
3D1 x 3D7	147.1	PRJNA256988	SRS670439
3D1 x 3D7	148.1	PRJNA256988	SRS670443
3D1 x 3D7	149.1	PRJNA256988	SRS670444
3D1 x 3D7	149.2	PRJNA256988	SRS670445
3D1 x 3D7	15.1	PRJNA256988	SRS670446
3D1 x 3D7	15.2	PRJNA256988	SRS670447
3D1 x 3D7	150.1	PRJNA256988	SRS670448
3D1 x 3D7	150.2	PRJNA256988	SRS670449
3D1 x 3D7	151.1	PRJNA256988	SRS670452
3D1 x 3D7	152.1	PRJNA256988	SRS670451

3D1 x 3D7	152.2	PRJNA256988	SRS670453
3D1 x 3D7	154.1	PRJNA256988	SRS670456
3D1 x 3D7	154.2	PRJNA256988	SRS670458
3D1 x 3D7	155.1	PRJNA256988	SRS670457
3D1 x 3D7	156.1	PRJNA256988	SRS670461
3D1 x 3D7	157.1	PRJNA256988	SRS670462
3D1 x 3D7	158.1	PRJNA256988	SRS670464
3D1 x 3D7	158.2	PRJNA256988	SRS670465
3D1 x 3D7	159.1	PRJNA256988	SRS670466
3D1 x 3D7	16.1	PRJNA256988	SRS670468
3D1 x 3D7	160.1	PRJNA256988	SRS670470
3D1 x 3D7	160.2	PRJNA256988	SRS670471
3D1 x 3D7	161.1	PRJNA256988	SRS670472
3D1 x 3D7	161.2	PRJNA256988	SRS670473
3D1 x 3D7	162.1	PRJNA256988	SRS670474
3D1 x 3D7	162.2	PRJNA256988	SRS670475
3D1 x 3D7	163.1	PRJNA256988	SRS670476
3D1 x 3D7	164.1	PRJNA256988	SRS670478
3D1 x 3D7	164.2	PRJNA256988	SRS670479
3D1 x 3D7	165.1	PRJNA256988	SRS670480
3D1 x 3D7	165.2	PRJNA256988	SRS670481
3D1 x 3D7	166.1	PRJNA256988	SRS670482
3D1 x 3D7	167.1	PRJNA256988	SRS670484
3D1 x 3D7	167.2	PRJNA256988	SRS670485
3D1 x 3D7	168.1	PRJNA256988	SRS670486
3D1 x 3D7	169.1	PRJNA256988	SRS670487
3D1 x 3D7	169.2	PRJNA256988	SRS670488
3D1 x 3D7	17.2	PRJNA256988	SRS670492
3D1 x 3D7	170.1	PRJNA256988	SRS670491
3D1 x 3D7	170.2	PRJNA256988	SRS670493
3D1 x 3D7	171.1	PRJNA256988	SRS670494
3D1 x 3D7	172.1	PRJNA256988	SRS670496
3D1 x 3D7	173.1	PRJNA256988	SRS670498
3D1 x 3D7	174.1	PRJNA256988	SRS670499
3D1 x 3D7	174.2	PRJNA256988	SRS670501
3D1 x 3D7	175.1	PRJNA256988	SRS670502
3D1 x 3D7	176.2	PRJNA256988	SRS670505
3D1 x 3D7	177.2	PRJNA256988	SRS670507
3D1 x 3D7	178.1	PRJNA256988	SRS670508
3D1 x 3D7	179.1	PRJNA256988	SRS670510
3D1 x 3D7	179.2	PRJNA256988	SRS670511
3D1 x 3D7	18.1	PRJNA256988	SRS670512
3D1 x 3D7	18.2	PRJNA256988	SRS670513

3D1 x 3D7	180.1	PRJNA256988	SRS670514
3D1 x 3D7	180.2	PRJNA256988	SRS670515
3D1 x 3D7	181.1	PRJNA256988	SRS670516
3D1 x 3D7	181.2	PRJNA256988	SRS670518
3D1 x 3D7	182.1	PRJNA256988	SRS670517
3D1 x 3D7	183.1	PRJNA256988	SRS670520
3D1 x 3D7	184.1	PRJNA256988	SRS670521
3D1 x 3D7	185.1	PRJNA256988	SRS670522
3D1 x 3D7	19.1	PRJNA256988	SRS670524
3D1 x 3D7	19.2	PRJNA256988	SRS670525
3D1 x 3D7	2.1	PRJNA256988	SRS670526
3D1 x 3D7	20.1	PRJNA256988	SRS670528
3D1 x 3D7	20.2	PRJNA256988	SRS670529
3D1 x 3D7	21.1	PRJNA256988	SRS670530
3D1 x 3D7	21.2	PRJNA256988	SRS670531
3D1 x 3D7	22.1	PRJNA256988	SRS670532
3D1 x 3D7	22.2	PRJNA256988	SRS670533
3D1 x 3D7	23.1	PRJNA256988	SRS670534
3D1 x 3D7	23.2	PRJNA256988	SRS670536
3D1 x 3D7	24.1	PRJNA256988	SRS670535
3D1 x 3D7	24.2	PRJNA256988	SRS670537
3D1 x 3D7	25.1	PRJNA256988	SRS670538
3D1 x 3D7	26.1	PRJNA256988	SRS670540
3D1 x 3D7	26.2	PRJNA256988	SRS670541
3D1 x 3D7	27.1	PRJNA256988	SRS670542
3D1 x 3D7	28.1	PRJNA256988	SRS670544
3D1 x 3D7	29.1	PRJNA256988	SRS670545
3D1 x 3D7	3.1	PRJNA256988	SRS670547
3D1 x 3D7	3.2	PRJNA256988	SRS670548
3D1 x 3D7	30.1	PRJNA256988	SRS670550
3D1 x 3D7	31.1	PRJNA256988	SRS670551
3D1 x 3D7	32.1	PRJNA256988	SRS670553
3D1 x 3D7	33.1	PRJNA256988	SRS670555
3D1 x 3D7	34.1	PRJNA256988	SRS670557
3D1 x 3D7	34.2	PRJNA256988	SRS670559
3D1 x 3D7	35.1	PRJNA256988	SRS670558
3D1 x 3D7	35.2	PRJNA256988	SRS670560
3D1 x 3D7	36.1	PRJNA256988	SRS670561
3D1 x 3D7	37.1	PRJNA256988	SRS670564
3D1 x 3D7	37.2	PRJNA256988	SRS670563
3D1 x 3D7	38.1	PRJNA256988	SRS670565
3D1 x 3D7	39.1	PRJNA256988	SRS670567
3D1 x 3D7	4.1	PRJNA256988	SRS670569
3D1 x 3D7	4.2	PRJNA256988	SRS670570

3D1 x 3D7	40.1	PRJNA256988	SRS670572
3D1 x 3D7	40.2	PRJNA256988	SRS670571
3D1 x 3D7	41.1	PRJNA256988	SRS670573
3D1 x 3D7	41.2	PRJNA256988	SRS670575
3D1 x 3D7	42.1	PRJNA256988	SRS670574
3D1 x 3D7	42.2	PRJNA256988	SRS670576
3D1 x 3D7	43.1	PRJNA256988	SRS670577
3D1 x 3D7	44.1	PRJNA256988	SRS670580
3D1 x 3D7	45.1	PRJNA256988	SRS670581
3D1 x 3D7	46.1	PRJNA256988	SRS670583
3D1 x 3D7	46.2	PRJNA256988	SRS670584
3D1 x 3D7	47.1	PRJNA256988	SRS670585
3D1 x 3D7	48.1	PRJNA256988	SRS670587
3D1 x 3D7	48.2	PRJNA256988	SRS670588
3D1 x 3D7	49.1	PRJNA256988	SRS670589
3D1 x 3D7	49.2	PRJNA256988	SRS670590
3D1 x 3D7	5.1	PRJNA256988	SRS670591
3D1 x 3D7	5.2	PRJNA256988	SRS670592
3D1 x 3D7	50.1	PRJNA256988	SRS670593
3D1 x 3D7	51.1	PRJNA256988	SRS670596
3D1 x 3D7	51.2	PRJNA256988	SRS670595
3D1 x 3D7	52.1	PRJNA256988	SRS670597
3D1 x 3D7	52.2	PRJNA256988	SRS670598
3D1 x 3D7	53.2	PRJNA256988	SRS670600
3D1 x 3D7	54.1	PRJNA256988	SRS670601
3D1 x 3D7	55.1	PRJNA256988	SRS670604
3D1 x 3D7	55.2	PRJNA256988	SRS670603
3D1 x 3D7	56.1	PRJNA256988	SRS670605
3D1 x 3D7	56.2	PRJNA256988	SRS670606
3D1 x 3D7	57.1	PRJNA256988	SRS670607
3D1 x 3D7	57.2	PRJNA256988	SRS670609
3D1 x 3D7	58.1	PRJNA256988	SRS670608
3D1 x 3D7	59.1	PRJNA256988	SRS670611
3D1 x 3D7	59.2	PRJNA256988	SRS670612
3D1 x 3D7	6.1	PRJNA256988	SRS670614
3D1 x 3D7	60.2	PRJNA256988	SRS670613
3D1 x 3D7	61.1	PRJNA256988	SRS670615
3D1 x 3D7	62.1	PRJNA256988	SRS670617
3D1 x 3D7	62.2	PRJNA256988	SRS670618
3D1 x 3D7	63.1	PRJNA256988	SRS670619
3D1 x 3D7	64.1	PRJNA256988	SRS670621
3D1 x 3D7	65.1	PRJNA256988	SRS670623
3D1 x 3D7	66.1	PRJNA256988	SRS670624

3D1 x 3D7	67.1	PRJNA256988	SRS670626
3D1 x 3D7	68.1	PRJNA256988	SRS670629
3D1 x 3D7	68.2	PRJNA256988	SRS670630
3D1 x 3D7	69.1	PRJNA256988	SRS670631
3D1 x 3D7	69.2	PRJNA256988	SRS670633
3D1 x 3D7	7.1	PRJNA256988	SRS670632
3D1 x 3D7	70.1	PRJNA256988	SRS670635
3D1 x 3D7	71.2	PRJNA256988	SRS670636
3D1 x 3D7	72.1	PRJNA256988	SRS670637
3D1 x 3D7	72.2	PRJNA256988	SRS670638
3D1 x 3D7	73.1	PRJNA256988	SRS670639
3D1 x 3D7	74.1	PRJNA256988	SRS670641
3D1 x 3D7	75.1	PRJNA256988	SRS670644
3D1 x 3D7	75.2	PRJNA256988	SRS670643
3D1 x 3D7	76.1	PRJNA256988	SRS670646
3D1 x 3D7	76.2	PRJNA256988	SRS670645
3D1 x 3D7	77.1	PRJNA256988	SRS670647
3D1 x 3D7	78.1	PRJNA256988	SRS670650
3D1 x 3D7	79.1	PRJNA256988	SRS670651
3D1 x 3D7	8.1	PRJNA256988	SRS670653
3D1 x 3D7	8.2	PRJNA256988	SRS670654
3D1 x 3D7	80.1	PRJNA256988	SRS670655
3D1 x 3D7	80.2	PRJNA256988	SRS670656
3D1 x 3D7	82.1	PRJNA256988	SRS670659
3D1 x 3D7	82.2	PRJNA256988	SRS670660
3D1 x 3D7	83.1	PRJNA256988	SRS670661
3D1 x 3D7	83.2	PRJNA256988	SRS670662
3D1 x 3D7	84.1	PRJNA256988	SRS670663
3D1 x 3D7	84.2	PRJNA256988	SRS670664
3D1 x 3D7	86.2	PRJNA256988	SRS670668
3D1 x 3D7	87.1	PRJNA256988	SRS670669
3D1 x 3D7	88.1	PRJNA256988	SRS670672
3D1 x 3D7	89.1	PRJNA256988	SRS670674
3D1 x 3D7	89.2	PRJNA256988	SRS670673
3D1 x 3D7	9.1	PRJNA256988	SRS670675
3D1 x 3D7	90.1	PRJNA256988	SRS670677
3D1 x 3D7	91.2	PRJNA256988	SRS670679
3D1 x 3D7	92.1	PRJNA256988	SRS670680
3D1 x 3D7	93.1	PRJNA256988	SRS670682
3D1 x 3D7	93.2	PRJNA256988	SRS670683
3D1 x 3D7	94.1	PRJNA256988	SRS670685
3D1 x 3D7	94.2	PRJNA256988	SRS670684
3D1 x 3D7	95.1	PRJNA256988	SRS670686
3D1 x 3D7	95.2	PRJNA256988	SRS670687

3D1 x 3D7	96.1	PRJNA256988	SRS670688
3D1 x 3D7	96.2	PRJNA256988	SRS670689
3D1 x 3D7	97.1	PRJNA256988	SRS670690
3D1 x 3D7	97.2	PRJNA256988	SRS670691
3D1 x 3D7	98.1	PRJNA256988	SRS670693
3D1 x 3D7	99.1	PRJNA256988	SRS670694
1A5 x 1E4	A1.1	PRJNA256991	SRS670696
1A5 x 1E4	A10.1	PRJNA256991	SRS670697
1A5 x 1E4	A11.1	PRJNA256991	SRS670699
1A5 x 1E4	A11.2	PRJNA256991	SRS670700
1A5 x 1E4	A12.2	PRJNA256991	SRS670701
1A5 x 1E4	A13.1	PRJNA256991	SRS670702
1A5 x 1E4	A13.2	PRJNA256991	SRS670704
1A5 x 1E4	A14.1	PRJNA256991	SRS670705
1A5 x 1E4	A14.2	PRJNA256991	SRS670706
1A5 x 1E4	A16.1	PRJNA256991	SRS670707
1A5 x 1E4	A16.2	PRJNA256991	SRS670708
1A5 x 1E4	A17.1	PRJNA256991	SRS670709
1A5 x 1E4	A18.1	PRJNA256991	SRS670711
1A5 x 1E4	A18.2	PRJNA256991	SRS670712
1A5 x 1E4	A19.1	PRJNA256991	SRS670713
1A5 x 1E4	A2.2	PRJNA256991	SRS670716
1A5 x 1E4	A21.1	PRJNA256991	SRS670717
1A5 x 1E4	A21.2	PRJNA256991	SRS670718
1A5 x 1E4	A22.1	PRJNA256991	SRS670720
1A5 x 1E4	A22.2	PRJNA256991	SRS670719
1A5 x 1E4	A23.1	PRJNA256991	SRS670722
1A5 x 1E4	A23.2	PRJNA256991	SRS670721
1A5 x 1E4	A24.1	PRJNA256991	SRS670723
1A5 x 1E4	A24.2	PRJNA256991	SRS670724
1A5 x 1E4	A25.1	PRJNA256991	SRS670725
1A5 x 1E4	A25.2	PRJNA256991	SRS670726
1A5 x 1E4	A26.1	PRJNA256991	SRS670727
1A5 x 1E4	A26.2	PRJNA256991	SRS670728
1A5 x 1E4	A28.1	PRJNA256991	SRS670729
1A5 x 1E4	A29.1	PRJNA256991	SRS670731
1A5 x 1E4	A3.1	PRJNA256991	SRS670733
1A5 x 1E4	A3.2	PRJNA256991	SRS670734
1A5 x 1E4	A30.1	PRJNA256991	SRS670735
1A5 x 1E4	A30.2	PRJNA256991	SRS670736
1A5 x 1E4	A31.1	PRJNA256991	SRS670738
1A5 x 1E4	A32.1	PRJNA256991	SRS670739
1A5 x 1E4	A33.1	PRJNA256991	SRS670741

1A5 x 1E4	A33.2	PRJNA256991	SRS670742
1A5 x 1E4	A35.2	PRJNA256991	SRS670744
1A5 x 1E4	A36.1	PRJNA256991	SRS670745
1A5 x 1E4	A37.1	PRJNA256991	SRS670747
1A5 x 1E4	A38.1	PRJNA256991	SRS670749
1A5 x 1E4	A39.1	PRJNA256991	SRS670751
1A5 x 1E4	A39.2	PRJNA256991	SRS670752
1A5 x 1E4	A4.1	PRJNA256991	SRS670753
1A5 x 1E4	A40.1	PRJNA256991	SRS670755
1A5 x 1E4	A40.2	PRJNA256991	SRS670756
1A5 x 1E4	A41.1	PRJNA256991	SRS670757
1A5 x 1E4	A42.1	PRJNA256991	SRS670760
1A5 x 1E4	A42.2	PRJNA256991	SRS670759
1A5 x 1E4	A43.1	PRJNA256991	SRS670762
1A5 x 1E4	A43.2	PRJNA256991	SRS670761
1A5 x 1E4	A44.1	PRJNA256991	SRS670763
1A5 x 1E4	A44.2	PRJNA256991	SRS670765
1A5 x 1E4	A45.1	PRJNA256991	SRS670766
1A5 x 1E4	A45.2	PRJNA256991	SRS670764
1A5 x 1E4	A46.1	PRJNA256991	SRS670767
1A5 x 1E4	A46.2	PRJNA256991	SRS670768
1A5 x 1E4	A47.1	PRJNA256991	SRS670769
1A5 x 1E4	A47.2	PRJNA256991	SRS670770
1A5 x 1E4	A48.1	PRJNA256991	SRS670771
1A5 x 1E4	A49.1	PRJNA256991	SRS670773
1A5 x 1E4	A5.1	PRJNA256991	SRS670775
1A5 x 1E4	A50.2	PRJNA256991	SRS670778
1A5 x 1E4	A51.2	PRJNA256991	SRS670779
1A5 x 1E4	A53.2	PRJNA256991	SRS670781
1A5 x 1E4	A54.1	PRJNA256991	SRS670780
1A5 x 1E4	A54.2	PRJNA256991	SRS670782
1A5 x 1E4	A55.1	PRJNA256991	SRS670783
1A5 x 1E4	A55.2	PRJNA256991	SRS670784
1A5 x 1E4	A57.1	PRJNA256991	SRS670785
1A5 x 1E4	A59.1	PRJNA256991	SRS670787
1A5 x 1E4	A59.2	PRJNA256991	SRS670788
1A5 x 1E4	A6.1	PRJNA256991	SRS670789
1A5 x 1E4	A60.1	PRJNA256991	SRS670791
1A5 x 1E4	A60.2	PRJNA256991	SRS670792
1A5 x 1E4	A62.1	PRJNA256991	SRS670793
1A5 x 1E4	A62.2	PRJNA256991	SRS670794
1A5 x 1E4	A63.1	PRJNA256991	SRS670795
1A5 x 1E4	A63.2	PRJNA256991	SRS670796
1A5 x 1E4	A64.1	PRJNA256991	SRS670797

1A5 x 1E4	A66.1	PRJNA256991	SRS670799
1A5 x 1E4	A66.2	PRJNA256991	SRS670800
1A5 x 1E4	A8.1	PRJNA256991	SRS670801
1A5 x 1E4	A9.1	PRJNA256991	SRS670803
1A5 x 1E4	A9.2	PRJNA256991	SRS670804
1A5 x 1E4	B1.1	PRJNA256991	SRS670805
1A5 x 1E4	B1.3	PRJNA256991	SRS670807
1A5 x 1E4	B10.1	PRJNA256991	SRS670808
1A5 x 1E4	B10.2	PRJNA256991	SRS670809
1A5 x 1E4	B11.1	PRJNA256991	SRS670811
1A5 x 1E4	B12.1	PRJNA256991	SRS670812
1A5 x 1E4	B13.2	PRJNA256991	SRS670815
1A5 x 1E4	B14.1	PRJNA256991	SRS670816
1A5 x 1E4	B15.1	PRJNA256991	SRS670818
1A5 x 1E4	B16.1	PRJNA256991	SRS670821
1A5 x 1E4	B16.2	PRJNA256991	SRS670820
1A5 x 1E4	B17.1	PRJNA256991	SRS670823
1A5 x 1E4	B17.2	PRJNA256991	SRS670824
1A5 x 1E4	B18.1	PRJNA256991	SRS670822
1A5 x 1E4	B18.2	PRJNA256991	SRS670826
1A5 x 1E4	B19.1	PRJNA256991	SRS670827
1A5 x 1E4	B19.2	PRJNA256991	SRS670825
1A5 x 1E4	B20.1	PRJNA256991	SRS670828
1A5 x 1E4	B20.2	PRJNA256991	SRS670829
1A5 x 1E4	B21.1	PRJNA256991	SRS670830
1A5 x 1E4	B22.1	PRJNA256991	SRS670831
1A5 x 1E4	B22.2	PRJNA256991	SRS670832
1A5 x 1E4	B23.1	PRJNA256991	SRS670833
1A5 x 1E4	B24.1	PRJNA256991	SRS670835
1A5 x 1E4	B24.2	PRJNA256991	SRS670836
1A5 x 1E4	B25.1	PRJNA256991	SRS670837
1A5 x 1E4	B26.1	PRJNA256991	SRS670839
1A5 x 1E4	B27.1	PRJNA256991	SRS670840
1A5 x 1E4	B28.1	PRJNA256991	SRS670842
1A5 x 1E4	B28.2	PRJNA256991	SRS670843
1A5 x 1E4	B29.1	PRJNA256991	SRS670844
1A5 x 1E4	B3.1	PRJNA256991	SRS670846
1A5 x 1E4	B30.1	PRJNA256991	SRS670847
1A5 x 1E4	B31.1	PRJNA256991	SRS670849
1A5 x 1E4	B31.2	PRJNA256991	SRS670850
1A5 x 1E4	B32.1	PRJNA256991	SRS670852
1A5 x 1E4	B32.2	PRJNA256991	SRS670851
1A5 x 1E4	B33.1	PRJNA256991	SRS670853

1A5 x 1E4	B33.2	PRJNA256991	SRS670854
1A5 x 1E4	B34.2	PRJNA256991	SRS670857
1A5 x 1E4	B35.1	PRJNA256991	SRS670856
1A5 x 1E4	B35.2	PRJNA256991	SRS670858
1A5 x 1E4	B37.1	PRJNA256991	SRS670859
1A5 x 1E4	B37.3	PRJNA256991	SRS670861
1A5 x 1E4	B38.1	PRJNA256991	SRS670862
1A5 x 1E4	B39.1	PRJNA256991	SRS670864
1A5 x 1E4	B39.2	PRJNA256991	SRS670865
1A5 x 1E4	B4.1	PRJNA256991	SRS670867
1A5 x 1E4	B4.2	PRJNA256991	SRS670868
1A5 x 1E4	B40.2	PRJNA256991	SRS670869
1A5 x 1E4	B41.1	PRJNA256991	SRS670870
1A5 x 1E4	B42.1	PRJNA256991	SRS670871
1A5 x 1E4	B42.2	PRJNA256991	SRS670872
1A5 x 1E4	B42.3	PRJNA256991	SRS670873
1A5 x 1E4	B43.1	PRJNA256991	SRS670874
1A5 x 1E4	B44.1	PRJNA256991	SRS670876
1A5 x 1E4	B45.1	PRJNA256991	SRS670878
1A5 x 1E4	B45.2	PRJNA256991	SRS670879
1A5 x 1E4	B46.1	PRJNA256991	SRS670880
1A5 x 1E4	B46.2	PRJNA256991	SRS670881
1A5 x 1E4	B48.1	PRJNA256991	SRS670883
1A5 x 1E4	B48.2	PRJNA256991	SRS670884
1A5 x 1E4	B49.1	PRJNA256991	SRS670885
1A5 x 1E4	B50.1	PRJNA256991	SRS670886
1A5 x 1E4	B50.2	PRJNA256991	SRS670888
1A5 x 1E4	B51.1	PRJNA256991	SRS670890
1A5 x 1E4	B7.1	PRJNA256991	SRS670891
1A5 x 1E4	B9.1	PRJNA256991	SRS670895
1A5 x 1E4	B9.2	PRJNA256991	SRS670896
1A5 x 1E4	C1.1	PRJNA256991	SRS670897
1A5 x 1E4	C1.2	PRJNA256991	SRS670898
1A5 x 1E4	C10.1	PRJNA256991	SRS670899
1A5 x 1E4	C11.1	PRJNA256991	SRS670901
1A5 x 1E4	C12.1	PRJNA256991	SRS670903
1A5 x 1E4	C12.2	PRJNA256991	SRS670904
1A5 x 1E4	C13.1	PRJNA256991	SRS670905
1A5 x 1E4	C14.1	PRJNA256991	SRS670907
1A5 x 1E4	C16.1	PRJNA256991	SRS670908
1A5 x 1E4	C16.2	PRJNA256991	SRS670910
1A5 x 1E4	C16.3	PRJNA256991	SRS670911
1A5 x 1E4	C17.1	PRJNA256991	SRS670912
1A5 x 1E4	C19.1	PRJNA256991	SRS670914

1A5 x 1E4	C2.1	PRJNA256991	SRS670917
1A5 x 1E4	C2.2	PRJNA256991	SRS670919
1A5 x 1E4	C20.1	PRJNA256991	SRS670918
1A5 x 1E4	C20.2	PRJNA256991	SRS670920
1A5 x 1E4	C21.1	PRJNA256991	SRS670921
1A5 x 1E4	C21.2	PRJNA256991	SRS670922
1A5 x 1E4	C22.1	PRJNA256991	SRS670923
1A5 x 1E4	C22.2	PRJNA256991	SRS670924
1A5 x 1E4	C23.1	PRJNA256991	SRS670925
1A5 x 1E4	C23.2	PRJNA256991	SRS670926
1A5 x 1E4	C24.1	PRJNA256991	SRS670927
1A5 x 1E4	C24.2	PRJNA256991	SRS670929
1A5 x 1E4	C25.1	PRJNA256991	SRS670928
1A5 x 1E4	C26.1	PRJNA256991	SRS670931
1A5 x 1E4	C27.1	PRJNA256991	SRS670933
1A5 x 1E4	C27.2	PRJNA256991	SRS670934
1A5 x 1E4	C28.1	PRJNA256991	SRS670935
1A5 x 1E4	C28.2	PRJNA256991	SRS670936
1A5 x 1E4	C29.2	PRJNA256991	SRS670938
1A5 x 1E4	C3.1	PRJNA256991	SRS670939
1A5 x 1E4	C3.2	PRJNA256991	SRS670940
1A5 x 1E4	C30.1	PRJNA256991	SRS670941
1A5 x 1E4	C30.2	PRJNA256991	SRS670942
1A5 x 1E4	C31.1	PRJNA256991	SRS670943
1A5 x 1E4	C32.1	PRJNA256991	SRS670946
1A5 x 1E4	C32.2	PRJNA256991	SRS670947
1A5 x 1E4	C33.1	PRJNA256991	SRS670948
1A5 x 1E4	C33.2	PRJNA256991	SRS670949
1A5 x 1E4	C34.1	PRJNA256991	SRS670950
1A5 x 1E4	C34.2	PRJNA256991	SRS670951
1A5 x 1E4	C35.1	PRJNA256991	SRS670952
1A5 x 1E4	C36.1	PRJNA256991	SRS670954
1A5 x 1E4	C38.1	PRJNA256991	SRS670956
1A5 x 1E4	C38.2	PRJNA256991	SRS670957
1A5 x 1E4	C4.1	PRJNA256991	SRS670958
1A5 x 1E4	C4.2	PRJNA256991	SRS670959
1A5 x 1E4	C41.1	PRJNA256991	SRS670960
1A5 x 1E4	C41.2	PRJNA256991	SRS670961
1A5 x 1E4	C42.1	PRJNA256991	SRS670962
1A5 x 1E4	C42.2	PRJNA256991	SRS670963
1A5 x 1E4	C44.1	PRJNA256991	SRS670964
1A5 x 1E4	C44.2	PRJNA256991	SRS670965
1A5 x 1E4	C45.1	PRJNA256991	SRS670966

1A5 x 1E4	C45.2	PRJNA256991	SRS670967
1A5 x 1E4	C48.1	PRJNA256991	SRS670968
1A5 x 1E4	C48.2	PRJNA256991	SRS670969
1A5 x 1E4	C49.1	PRJNA256991	SRS670970
1A5 x 1E4	C5.1	PRJNA256991	SRS670972
1A5 x 1E4	C5.2	PRJNA256991	SRS670973
1A5 x 1E4	C51.1	PRJNA256991	SRS670974
1A5 x 1E4	C51.2	PRJNA256991	SRS670975
1A5 x 1E4	C52.1	PRJNA256991	SRS670976
1A5 x 1E4	C53.1	PRJNA256991	SRS670977
1A5 x 1E4	C54.1	PRJNA256991	SRS670979
1A5 x 1E4	C55.1	PRJNA256991	SRS670981
1A5 x 1E4	C55.2	PRJNA256991	SRS670982
1A5 x 1E4	C56.1	PRJNA256991	SRS670983
1A5 x 1E4	C56.2	PRJNA256991	SRS670985
1A5 x 1E4	C57.1	PRJNA256991	SRS670986
1A5 x 1E4	C57.2	PRJNA256991	SRS670987
1A5 x 1E4	C59.2	PRJNA256991	SRS670989
1A5 x 1E4	C6.1	PRJNA256991	SRS670990
1A5 x 1E4	C60.1	PRJNA256991	SRS670992
1A5 x 1E4	C60.2	PRJNA256991	SRS670993
1A5 x 1E4	C61.1	PRJNA256991	SRS670994
1A5 x 1E4	C61.2	PRJNA256991	SRS670995
1A5 x 1E4	C62.1	PRJNA256991	SRS670996
1A5 x 1E4	C62.2	PRJNA256991	SRS670997
1A5 x 1E4	C63.1	PRJNA256991	SRS670998
1A5 x 1E4	C7.1	PRJNA256991	SRS671000
1A5 x 1E4	C7.2	PRJNA256991	SRS671001
1A5 x 1E4	C8.1	PRJNA256991	SRS671002
1A5 x 1E4	CR4_A1.1	PRJNA256991	SRS671004
1A5 x 1E4	CR4_A2.1	PRJNA256991	SRS671006
1A5 x 1E4	CR4_A2.2	PRJNA256991	SRS671007
1A5 x 1E4	CR4_A3.1	PRJNA256991	SRS671010
1A5 x 1E4	CR4_A3.2	PRJNA256991	SRS671012
1A5 x 1E4	CR4_A4.1	PRJNA256991	SRS671015
1A5 x 1E4	D1.1	PRJNA256991	SRS671014
1A5 x 1E4	D1.2	PRJNA256991	SRS671016
1A5 x 1E4	D1.3	PRJNA256991	SRS671018
1A5 x 1E4	D10.1	PRJNA256991	SRS671019
1A5 x 1E4	D11.1	PRJNA256991	SRS671021
1A5 x 1E4	D2.1	PRJNA256991	SRS671023
1A5 x 1E4	D3.1	PRJNA256991	SRS671026
1A5 x 1E4	D3.2	PRJNA256991	SRS671025
1A5 x 1E4	D4.1	PRJNA256991	SRS671027

1A5 x 1E4	D5.1	PRJNA256991	SRS671029
1A5 x 1E4	D7.1	PRJNA256991	SRS671031
1A5 x 1E4	D7.2	PRJNA256991	SRS671032
1A5 x 1E4	D7.3	PRJNA256991	SRS671033
1A5 x 1E4	D9.1	PRJNA256991	SRS671035
1A5 x 1E4	D9.2	PRJNA256991	SRS671038

Table S2   Camera and light setup overview.

Parameter	Specification
Camera height	45 cm
Lights	Two boxes with each two light bulbs (set up as a X). Boxes are setup to the left and right!
Bulbs	PHILIPHS TL 20W/55 DE LUXE
Background	Blue paper (single layered)
Calibration reference	Millimeter grid paper
Focus reference	Petri dish lid with a labeling 'Focus Reference'

Table S3 Detailed information on the camera and lens used during image taking, as well as detailed information regarding the camera settings.

Camera type / Parameter [a]	Specification / Settings
Body	CANON EOS 60D Body
Lens	CANON EF 50mm F/2.5 Compact Macro
ISO-Sensitivity	200
Aperture (Depth of field)	14
Shutter speed 1)	5
Focus point 2)	Set to the most right square
Light exposure measurement	full
Quality	RAW (5184x3456) and JPEG (5184x3456)
Perpheral illumin. Correct.	Enable
Red-eye reduc.	Disable
Flash control (Flash firing)	Disable
Expo.comp./AEB	Set to Zero (in the center)
Auto Lighting Optimizer	Standard
Picture Style	Standard
White Balance	AWB (=automatic)
WB Shift/BKT	0,0/±0
Color space	sRGB
ISO Auto	Max: 3200
Live View shoot.	Enable
AF mode	Live mode
Grid display	OFF
Aspect ratio	3:2
Expo.simulation	Enable
Silent shooting	Mode 1
Metering timer	4 sec.
File numbering	Continuous

[a] 1) The shutter speed was set to 5, because under the specific light settings this value provided optimal illumination conditions. 2) The focus point was not set onto the fungal colonies, but to a Petri dish lid, just beside the plate containing the colonies. This additional lid was labeled with 'Focus Reference'. This labeling was at the same height as the fungal colonies, thus resulting for all colonies in sharp images, even if the height of the medium varied a bit.

Table S4 **Melanization phenotypes (grey values) for the quality filter retained progeny used in the QTL analysis for each of the two crosses.**

		Environment / Colony age (dpi)								
Cross	Retained progeny for QTL analysis	Cold / 8	Cold / 11	Cold / 14	Control / 8	Control / 11	Control / 14	Fungicide / 8	Fungicide / 11	Fungicide / 14
3D1 x 3D7	1.1	NA	93.04607873	63.78769444	NA	71.23581861	67.52683429	NA	67.02107424	69.7407959
3D1 x 3D7	1.2	138.9211667	122.9319667	97.11144524	96.55215	58.83029167	51.17904167	90.96024888	65.84066857	52.35253429
3D1 x 3D7	10.1	NA	64.8691709	49.19910134	NA	57.37409722	52.89087429	NA	58.08289303	61.88949621
3D1 x 3D7	100.1	126.0365062	96.51860995	84.74405776	84.4686653	54.38001237	41.18947667	97.21626125	61.62162222	43.78903833
3D1 x 3D7	100.2	NA	NA	NA	130.362	111.038	89.081	122.192	103.41	80.703
3D1 x 3D7	101.2	105.2208167	110.885	82.07116667	115.7047548	87.58248333	70.77283333	113.8538611	92.12066667	77.19388889
3D1 x 3D7	102.1	105.6240078	58.91512232	62.00044471	70.04411476	54.14168105	44.92877808	75.14588822	52.81095889	45.30180626
3D1 x 3D7	102.2	130.57	107.3673333	89.673	125.4268	123.7577667	98.14745	128.3555556	110.1217778	84.55083333
3D1 x 3D7	103.1	123.2223212	86.10474875	58.1981572	79.39884322	50.95954548	39.97316381	85.77439636	53.72039736	41.51781945
3D1 x 3D7	104.1	129.5802667	106.52175	100.1136917	84.41975333	68.6814	53.95695	79.29458333	70.58371667	56.54508334
3D1 x 3D7	104.2	128.1313236	97.07834538	77.17254615	120.69925	113.3807896	94.37626944	124.7285833	104.5802222	80.3585
3D1 x 3D7	105.1	136.8757474	120.7077669	105.5548362	125.8309911	70.91158182	59.40977424	114.7506416	68.71309821	58.40494
3D1 x 3D7	105.2	143.5365783	141.1571521	119.8104176	131.9361982	106.5099222	81.25990333	137.0743612	102.7411322	83.67342
3D1 x 3D7	106.1	139.6565128	114.8448463	77.57321724	128.167917	63.6189048	56.07898959	99.45640081	74.7566568	61.05086472
3D1 x 3D7	106.2	115.9161838	79.5166859	62.98536364	72.47211933	60.85494351	53.55747143	95.35758965	67.89552051	58.22254167
3D1 x 3D7	107.1	139.9931532	134.0789152	104.7542548	99.64869838	69.55389269	59.78968333	100.5925153	71.2904198	56.71587476
3D1 x 3D7	108.1	126.0041042	100.9846077	88.60353125	84.49662993	62.95990197	50.02795222	87.86632974	61.15597824	47.16040321
3D1 x 3D7	108.2	129.5529	82.22087905	56.18452	117.9318333	117.24645	97.407375	119.83958	110.3138833	85.44816667
3D1 x 3D7	109.1	135.3257329	120.0563643	97.52829116	82.16497106	65.55824286	61.74073047	82.79800085	67.83865263	58.78931581
3D1 x 3D7	109.2	137.3307708	123.0388448	93.01046042	96.44440355	67.56110829	52.35975952	100.2994008	72.21352232	56.7993125

3D1 x 3D7	11.1	137.6486214	131.1213452	108.9592876	132.0365407	90.66232009	66.06892381	133.1571107	109.75148	78.56545167
3D1 x 3D7	11.2	NA	121.8148423	111.8334222	NA	120.8221206	117.90772	NA	126.1377512	117.5103294
3D1 x 3D7	110.1	126.5874751	95.12559761	78.28639186	100.1105441	65.51498795	47.47087143	115.2536224	67.42445379	43.80073636
3D1 x 3D7	110.2	NA	NA	NA	125.4243611	106.8474572	85.39096667	122.3512603	110.7835679	90.86082619
3D1 x 3D7	111.2	137.30737	136.2373752	97.88299143	117.6417819	80.28050952	76.18885953	118.6178548	90.40874	77.8929
3D1 x 3D7	112.1	135.478825	115.7907415	97.84553665	105.8666501	68.53853043	48.48154286	118.8581524	74.87240171	53.75149107
3D1 x 3D7	113.1	125.3779453	95.21866615	76.047	85.39857165	62.78296942	53.28581778	97.93049	66.821415	54.44839
3D1 x 3D7	113.2	128.519147	133.066299	124.0499274	142.3264405	139.8238529	119.5206	148.5043167	147.0384889	125.2621607
3D1 x 3D7	114.1	138.3819095	124.2893086	105.4281481	96.22480025	66.47385357	54.10065476	99.12644524	68.84467762	54.68510667
3D1 x 3D7	114.2	133.3009271	121.1906575	80.90807242	125.2174758	78.20477222	55.09530286	116.1495555	75.72660556	60.96945833
3D1 x 3D7	115.1	136.0427333	98.37231537	71.32154925	89.0400375	63.88561667	48.7861197	108.78675	76.25120833	53.87179167
3D1 x 3D7	116.1	135.0430083	115.5107914	84.17046389	118.9417083	91.14092897	71.23753333	128.22167	96.84129	66.53856333
3D1 x 3D7	116.2	140.8755833	139.82436	78.72343333	93.04799778	73.22596111	58.11796667	98.49983333	72.07444444	58.56844444
3D1 x 3D7	117.1	132.4399439	122.5927791	106.0175239	119.3944039	81.66589767	67.64874889	112.8362233	76.85737111	57.38383143
3D1 x 3D7	117.2	128.52064	105.45836	79.7792	75.26450762	68.66133143	56.7680875	94.56266667	75.62916667	58.88944444
3D1 x 3D7	118.1	133.3702659	112.158625	83.27358667	119.619208	91.38438881	64.85122679	122.2293167	96.69215	64.49689143
3D1 x 3D7	118.2	126.11014	84.838625	55.55818333	138.3078333	123.9671667	117.181125	142.0044792	127.4886042	108.3779167
3D1 x 3D7	119.1	145.1185318	135.580901	117.24819	134.9084222	119.741765	101.1712333	139.3677143	123.6001867	110.5751833
3D1 x 3D7	119.2	139.7671636	133.0280805	99.67200237	127.5515459	99.94991549	75.25956643	126.7247514	89.38076064	73.23461905
3D1 x 3D7	12.1	139.7715	110.0047283	94.17649128	102.651645	66.20026864	56.78980476	108.3259117	72.55310973	61.16291869
3D1 x 3D7	120.1	134.7883886	105.9334518	79.61856238	133.1658166	95.6161536	67.51032576	135.1733952	93.09351571	65.90646667
3D1 x 3D7	120.2	124.2219805	110.072525	100.2247767	95.65480329	85.03769111	86.65640714	90.14887841	83.99976778	81.37065333
3D1 x 3D7	121.2	123.9392351	98.98418546	79.66360635	92.39195763	65.91036984	54.76798907	89.79158722	70.27571382	58.98644444
3D1 x 3D7	122.1	126.5892667	109.40377	80.8714	101.6230529	57.814995	48.83373333	98.04725	59.8783	46.02056667
3D1 x 3D7	123.1	NA	69.50102659	49.82799148	NA	58.8571529	46.36049889	NA	68.11835801	54.66151539

3D1 x 3D7	125.1	126.4556433	100.3504289	77.63635833	85.64143535	59.08528523	53.43250051	90.51655429	69.37335	61.18938809
3D1 x 3D7	126.1	139.5133892	118.4039468	94.91439377	76.11003998	66.28649052	64.25514187	75.358708	65.68804542	67.48054593
3D1 x 3D7	127.1	131.725375	117.4720167	104.2255	101.6777198	81.4786	68.09599333	124.9721167	90.83951667	75.69251667
3D1 x 3D7	127.2	143.4491733	122.447816	81.54710834	131.3147957	111.5763038	92.14050667	123.9024349	91.32499	63.9832
3D1 x 3D7	128.1	NA	105.0129791	76.99662611	NA	82.70266069	74.0846381	NA	113.1437882	99.79397253
3D1 x 3D7	128.2	127.0527224	78.39297549	59.571295	78.78503135	58.73308359	52.63618222	90.26637411	61.753475	50.01614445
3D1 x 3D7	129.2	140.1191589	114.5787879	93.51255278	134.9651768	119.3261459	93.968775	126.6757781	106.5582031	82.00331905
3D1 x 3D7	13.1	134.0823333	121.9055201	104.598197	116.7126463	79.55738214	61.99740833	117.3200201	87.98913532	73.77595577
3D1 x 3D7	130.1	136.1447291	122.906545	103.4695933	78.05342548	54.76757206	49.95019	97.02448333	63.06407	50.06703333
3D1 x 3D7	131.1	141.5348463	124.7765354	84.07406762	109.4931242	105.9982066	91.02191667	125.7177935	121.0286218	99.46412
3D1 x 3D7	133.1	131.9478242	106.4417907	67.59122038	97.33066485	73.30647811	58.20252214	113.4148019	95.20031684	69.50066061
3D1 x 3D7	134.1	143.5851279	134.3886446	102.1055175	124.3813	111.7503562	92.38336143	136.938979	126.3972179	110.295
3D1 x 3D7	135.2	130.4660128	114.7943829	97.77874911	127.2511994	99.90496112	77.859375	123.9033679	93.88124672	72.15154167
3D1 x 3D7	136.1	140.7493921	129.8525634	82.80071678	127.8523795	94.4159059	70.69312222	134.8109516	108.0835791	81.27556254
3D1 x 3D7	137.1	119.4746667	110.7685583	85.52414583	102.61695	73.00778333	49.91662	91.11275333	66.73592667	56.01036667
3D1 x 3D7	137.2	129.5483633	112.82077	92.908915	128.7930011	121.4635848	109.73014	131.7700625	119.1038375	107.63625
3D1 x 3D7	138.1	127.0638667	83.92144286	53.52492238	84.11145338	58.71760143	49.02142889	85.82568	63.03203	48.49969
3D1 x 3D7	139.2	125.9147446	108.3824429	107.074289	87.53323333	58.30098714	49.5000881	112.3985143	78.85935714	59.55893571
3D1 x 3D7	14.1	NA	83.32912603	53.33956	NA	49.97735758	41.21580381	NA	64.96018294	50.04632222
3D1 x 3D7	141.1	115.4414806	73.28415486	59.03199092	89.82217616	79.61292417	60.25002833	109.025493	86.46800278	70.04466888
3D1 x 3D7	142.1	125.3967	93.19292	61.25091	80.61012	64.16727	58.29271	83.57514333	82.07171	63.61969667
3D1 x 3D7	142.2	142.21608	123.4780691	103.7644203	98.01887413	62.77557698	50.43148333	92.194025	63.94450625	50.59447917
3D1 x 3D7	143.1	130.7703195	103.581716	73.65817778	119.7673708	92.76618333	69.39238032	137.1490394	114.8420785	83.41273809
3D1 x 3D7	144.1	138.1035984	133.8091562	70.73748056	106.6117286	80.84769157	65.92154254	82.92068762	65.62776071	59.47129333
3D1 x 3D7	145.1	135.3504	133.8312	86.7972	113.604875	69.32104167	51.56841667	95.634	69.729625	49.977875

3D1 x 3D7	145.2	129.2534468	73.03755654	56.01757783	79.4303338	59.82851984	54.35139405	88.94466165	62.80936379	53.76453056
3D1 x 3D7	146.1	118.8807422	81.40470303	62.60609658	74.08911486	58.54237652	47.38810556	92.77377802	69.4047919	60.45700377
3D1 x 3D7	146.2	132.6046987	111.6485549	74.14845667	122.1992651	101.986996	81.48734048	135.4102204	106.7135515	78.93662286
3D1 x 3D7	147.1	136.4732993	128.67055	108.254875	134.6993644	120.7140088	99.95066	139.6552089	124.4744369	106.8345488
3D1 x 3D7	148.1	136.286	114.7665762	82.628	125.8477333	117.1805	98.0468	137.6229	117.5911333	103.2995333
3D1 x 3D7	149.1	131.1865095	128.3453467	116.5734	112.1270984	73.51390333	49.46273333	94.54061667	63.88535	51.77802
3D1 x 3D7	149.2	120.8365625	95.95891667	75.98576667	98.12266667	69.509	54.48325	120.652875	104.4980625	83.12341667
3D1 x 3D7	15.1	NA	111.2883258	83.38954571	NA	79.41166167	60.071305	NA	72.57174698	56.74346091
3D1 x 3D7	15.2	NA	85.192125	53.33523667	NA	67.56582	44.65534583	NA	61.62569524	53.80952
3D1 x 3D7	150.1	125.2006901	96.7623936	62.65496821	114.4581669	91.90862167	70.32053929	126.4477333	102.5120111	80.92061
3D1 x 3D7	150.2	127.8079833	94.45016667	71.11658333	117.9136214	75.18476429	59.65614048	107.148	78.792	70.349
3D1 x 3D7	151.1	122.6230759	98.57915474	78.37785353	130.1197491	97.49294555	69.00558333	128.9237327	104.1192422	80.02399091
3D1 x 3D7	152.1	141.7708667	128.1424171	94.66597238	124.0634471	104.0821583	79.34974306	121.41195	96.74219	78.05893333
3D1 x 3D7	152.2	139.6088605	127.4561053	86.96286372	93.76765471	70.264686	60.49020409	90.42330261	75.80675778	64.27734476
3D1 x 3D7	154.1	138.0291878	120.4358417	100.9598698	115.1169251	86.2850923	66.31999048	124.7431726	94.19792538	68.14131399
3D1 x 3D7	154.2	133.12278	132.6397492	119.433145	116.4124	101.28245	72.76950333	113.42433	87.48600333	58.78476667
3D1 x 3D7	155.1	128.35642	91.59913333	62.19364444	91.89161623	74.82485119	54.38807778	104.7476958	75.24792262	58.17046072
3D1 x 3D7	156.1	120.3050869	73.37593952	63.11625915	90.66820417	71.42360812	56.7617875	88.0975903	70.99817429	56.71396571
3D1 x 3D7	157.1	145.3160951	108.641799	94.49760608	111.9562691	73.80657301	50.79755	106.2519157	75.28544077	55.66672061
3D1 x 3D7	158.1	144.9867352	99.01515128	63.72135032	85.61962574	60.51236923	52.34413	89.64194496	63.89058974	52.76340741
3D1 x 3D7	158.2	140.6205324	134.9761876	118.6019507	107.4968587	72.74571847	57.73827746	112.6076579	73.71987929	59.36658241
3D1 x 3D7	159.1	129.3485369	93.50156855	85.11393056	82.90811275	66.96638244	58.19407802	85.6230771	63.60724387	52.54960675
3D1 x 3D7	16.1	136.2823619	106.9365986	70.32432833	105.1598383	92.32704722	86.28127083	129.1850959	106.6898065	85.71427762
3D1 x 3D7	160.1	124.8917081	91.80438838	87.26488643	131.9349647	114.8857203	89.0324873	130.1053634	110.7333865	87.97914643
3D1 x 3D7	160.2	132.7578886	107.5048927	82.40952286	97.73144359	76.27823433	61.50911905	124.2041796	102.113184	76.92891429

3D1 x 3D7	161.1	114.9409848	82.71843261	79.32695781	83.57319937	67.72782061	58.15893	99.9556764	75.35892381	60.68038889
3D1 x 3D7	161.2	126.16632	88.48092736	60.78921611	115.965403	100.6948312	86.29	113.9808894	97.87364215	81.15535357
3D1 x 3D7	162.1	112.5389223	61.18032419	66.87168131	85.64414134	66.32368606	54.04496299	101.0176697	81.77553891	58.71008232
3D1 x 3D7	162.2	142.0265508	101.1079996	83.996875	120.0816425	87.86108921	76.23789286	115.9747656	78.46690183	66.89146578
3D1 x 3D7	163.1	116.2694698	79.69892045	62.5408549	89.94441995	62.28876657	46.71306806	85.43330952	69.03779977	52.04257833
3D1 x 3D7	164.1	141.3336511	139.7041262	133.1069132	138.5022579	122.944054	86.19689039	134.1091958	128.660429	96.45068538
3D1 x 3D7	164.2	130.4233825	77.79271731	63.12385139	85.29031857	66.18034675	56.76234921	111.9178215	74.39504266	59.81449167
3D1 x 3D7	165.1	141.7799278	120.2202652	85.1389498	130.8062332	86.82499984	64.84592292	119.3387083	85.5438497	68.66865833
3D1 x 3D7	165.2	140.5679498	126.6398297	94.29226089	103.5160775	77.99954265	65.83169047	95.78463277	74.20973939	58.44691587
3D1 x 3D7	166.1	140.6971626	122.0042207	114.9363918	88.64881645	64.79402064	56.74132484	106.7851129	84.66343045	63.83962937
3D1 x 3D7	167.1	139.3141667	135.0375986	124.2252	136.7217143	142.5445778	139.22575	146.5065333	147.9730857	135.04325
3D1 x 3D7	167.2	140.1945519	135.0509203	100.6235077	103.7432064	61.63875268	56.9507875	104.0934101	61.93783445	52.77663632
3D1 x 3D7	168.1	137.4919675	131.4617516	122.27475	126.7435308	112.2218591	72.28571191	109.4642303	100.3596607	70.47102143
3D1 x 3D7	169.1	121.9518131	91.7737569	69.72953667	106.9001767	91.21401799	80.64860278	135.9567168	109.0450082	86.70945873
3D1 x 3D7	169.2	133.4081591	104.632679	89.26455794	92.91455357	86.19270667	72.29893333	95.70178745	86.33760273	62.88875682
3D1 x 3D7	17.2	NA	78.02109722	54.53772727	NA	85.84417613	72.71295175	NA	96.9145714	81.59784473
3D1 x 3D7	170.1	133.4990222	112.38848	94.14356667	104.4950397	75.96337042	57.4673	91.141675	62.02766806	51.631275
3D1 x 3D7	170.2	130.4647887	122.1167192	99.50609158	96.46677897	73.62106374	53.98268816	81.29295325	64.64216982	51.46869637
3D1 x 3D7	171.1	136.9493321	101.8828103	77.79513857	113.2749764	80.69472718	63.04177083	113.3054646	83.92688542	67.251925
3D1 x 3D7	172.1	131.3999605	97.87915555	73.07184722	94.15807478	79.93741238	61.98920635	103.2158988	87.81111458	68.5316625
3D1 x 3D7	173.1	128.2914354	101.0742199	69.81236667	120.2945938	92.97012777	69.64448611	132.6136389	93.72208333	73.5067619
3D1 x 3D7	174.1	NA	133.973075	103.3649967	81.41447786	57.23125476	49.63572308	92.17682792	63.07896575	50.92446667
3D1 x 3D7	174.2	134.6040208	121.4943916	99.81489299	129.7922309	114.1100626	101.15172	137.0385239	112.3471531	98.18325556
3D1 x 3D7	175.1	136.9860548	127.7213121	98.38204366	98.94851281	71.53172436	56.63314048	101.5353816	76.76785069	61.03854524
3D1 x 3D7	176.2	135.6852333	134.4379333	94.76	88.8825381	64.45169047	56.53269667	89.58376111	61.75283492	54.26904762

3D1 x 3D7	177.2	133.1597735	103.0041777	71.98844051	85.96196895	80.39028286	78.73309167	94.20947329	91.685755	76.73945833
3D1 x 3D7	178.1	NA	NA	NA	NA	NA	NA	126.9862143	92.74939286	82.50085715
3D1 x 3D7	179.1	136.8075833	128.5307389	91.42731667	122.78785	88.36983306	77.86247222	128.5165	100.612375	84.09683333
3D1 x 3D7	179.2	132.0871729	102.2969964	84.54231337	89.65980145	64.50878737	55.3489295	93.37581436	66.74216143	59.45812667
3D1 x 3D7	18.1	NA	74.11730128	63.12752571	NA	70.05034295	73.37750333	NA	71.63110883	61.39760389
3D1 x 3D7	18.2	NA	79.52240393	51.92528031	NA	64.17108769	47.83613551	NA	96.81743163	78.69287397
3D1 x 3D7	180.1	130.928	125.6056111	110.2960417	102.5074846	61.14320089	48.77499762	101.8163208	67.18779167	59.23447917
3D1 x 3D7	180.2	123.4830833	91.92473651	82.19987143	93.62233333	69.86728571	53.272	122.2260441	96.3794127	70.87319444
3D1 x 3D7	181.1	138.5844167	118.37634	94.73722921	127.1647764	117.1652574	104.1304444	129.1146393	121.4279271	110.6032292
3D1 x 3D7	181.2	133.9570464	100.5583562	73.34261857	92.76504167	89.20709333	76.90155	90.9479006	77.88104091	61.97859281
3D1 x 3D7	182.1	138.8383854	132.047874	101.2305104	106.9959371	64.53352258	57.63130667	90.86887583	64.45460702	57.52971167
3D1 x 3D7	183.1	NA	NA	NA	NA	NA	NA	NA	NA	NA
3D1 x 3D7	184.1	NA	NA	NA	NA	NA	NA	NA	NA	NA
3D1 x 3D7	185.1	NA	NA	NA	NA	NA	NA	NA	NA	NA
3D1 x 3D7	19.1	138.7258714	107.6435993	82.88130148	103.3610352	78.20949838	61.11105111	117.9348998	94.24286286	73.7936619
3D1 x 3D7	19.2	NA	107.6364694	72.39309874	NA	85.75249216	56.85602167	NA	76.3162016	62.8778537
3D1 x 3D7	2.1	120.7387152	73.80284558	53.2984424	71.45976675	59.86445624	53.14225512	100.9353505	73.39745619	59.47159333
3D1 x 3D7	20.1	147.066	115.4841667	69.31626667	95.77294643	70.54279464	61.3963125	122.2411944	97.62733334	83.31825
3D1 x 3D7	20.2	123.4709953	97.40425237	63.45829606	NA	NA	NA	113.3631644	90.21282463	67.59146515
3D1 x 3D7	21.1	119.0331243	79.74781381	50.06561714	101.3145369	70.13502747	51.53922047	107.9206684	72.78840476	53.30451872
3D1 x 3D7	21.2	138.2668083	133.4046289	90.352745	100.6563	63.72740222	53.8289927	103.1368583	69.40558399	60.48088413
3D1 x 3D7	22.1	127.368559	72.98749167	51.847525	93.68857333	59.97400333	49.10106667	102.3140991	69.31290667	50.18473333
3D1 x 3D7	22.2	132.1485744	122.2830277	96.73257455	90.00063577	88.60251508	77.730375	114.7322311	93.48751581	81.75186905
3D1 x 3D7	23.1	140.7580371	112.4338281	87.59477607	96.36800265	63.78457273	56.1898875	110.0397162	71.49468462	56.49143778
3D1 x 3D7	23.2	111.2859571	72.89072999	69.66789171	72.92616186	60.61715912	51.36600897	80.51897992	64.78053619	57.30784974

3D1 x 3D7	24.1	125.5408957	91.2279461	73.49852576	97.42646881	85.0940594	82.83992333	135.9855603	98.26310206	92.04419333
3D1 x 3D7	24.2	122.6635302	74.30309643	57.48431317	92.277975	59.74193222	50.36335667	95.16678889	66.39898667	53.14889667
3D1 x 3D7	25.1	123.7440512	76.32463467	61.97325604	81.67686039	64.00770968	65.80936667	91.84036791	72.28121398	66.28256528
3D1 x 3D7	26.1	140.2089124	134.5376982	113.3056728	113.6351578	76.04276089	61.45908294	112.2144822	76.8064081	68.85675286
3D1 x 3D7	26.2	122.0893114	90.12150899	66.15026381	74.09280354	54.49137167	43.91788333	85.62801631	67.28101238	52.85703506
3D1 x 3D7	27.1	137.9265	97.268225	68.1778125	121.5021	100.93985	85.25397917	131.6998	96.7159	82.45925
3D1 x 3D7	28.1	140.1991462	135.1912308	119.8964695	115.6988642	79.92737254	79.81264948	113.6345348	83.9633832	72.56067293
3D1 x 3D7	29.1	136.9227365	94.89935517	66.83721091	110.1512011	73.20999159	67.25682222	117.3635191	76.75948	67.31321191
3D1 x 3D7	3.1	135.5114162	132.5341077	124.030381	130.4991552	99.91538214	85.47613524	144.4285324	113.2363191	94.00156667
3D1 x 3D7	3.2	NA	107.2703559	64.23136177	NA	67.22586355	52.98679189	NA	64.34867429	51.22074476
3D1 x 3D7	30.1	136.137124	100.6566617	80.20256547	83.55256528	61.18095386	53.02263854	88.22787638	59.62700433	46.67800013
3D1 x 3D7	31.1	140.299	131.90167	115.1181386	130.8839768	104.23676	81.53833	132.2737457	95.49844333	74.93745
3D1 x 3D7	32.1	139.6322857	133.0101176	92.56205882	105.6008301	84.40129808	60.34463636	122.6354031	103.2017243	80.88757446
3D1 x 3D7	33.1	137.7362917	85.02304286	50.20698809	129.2478433	84.59907667	53.65629	131.2358743	77.19615333	54.93131667
3D1 x 3D7	34.1	128.8797411	80.97609762	66.22621944	105.5443994	74.50585111	61.6895031	111.4458092	83.48721312	67.43619141
3D1 x 3D7	34.2	135.6100941	133.8769663	97.29378749	130.6120754	109.3446677	81.57846212	124.8864203	101.5228742	78.99150222
3D1 x 3D7	35.1	151.3156667	135.808625	107.8965833	118.443	80.05411333	64.34767778	103.7530875	90.82901667	80.89579167
3D1 x 3D7	35.2	129.0207497	106.7253499	78.1549258	105.7457097	77.39811111	66.00772917	136.0357192	99.6603437	78.43361119
3D1 x 3D7	36.1	129.9945	105.3896333	69.2704	99.90005	71.04260833	57.93026667	108.9648571	82.60601429	69.52265714
3D1 x 3D7	37.1	138.7162725	126.4002913	93.65840051	71.75059888	62.1588506	60.5575518	82.24823684	68.06613503	61.01281925
3D1 x 3D7	37.2	128.931	99.48766667	79.28011111	110.0875	76.6515	57.3565	97.5155	66.30625	48.82704167
3D1 x 3D7	38.1	NA	133.1666807	127.3755176	NA	116.1471719	70.44852164	NA	104.3004748	88.90872074
3D1 x 3D7	39.1	NA	131.3461301	93.78400151	NA	94.60087308	74.96788155	NA	89.10517679	66.84571429
3D1 x 3D7	4.1	NA	90.05499416	73.03173041	NA	52.72491286	46.65054381	NA	64.51305291	56.62683689
3D1 x 3D7	4.2	NA	124.1556813	73.6604533	NA	64.56066044	48.38919464	NA	69.79101837	56.30538159

3D1 x 3D7	40.1	141.70531	107.8079846	82.61208714	100.6079834	84.92258603	75.18412286	122.9637647	99.98711556	85.53970786
3D1 x 3D7	40.2	121.3964834	78.51984117	61.78659825	78.12761823	61.414025	49.45535	101.8484005	75.96920917	55.26850249
3D1 x 3D7	41.1	130.7753503	97.19234806	73.98085974	80.533316	57.7114879	53.42984048	97.68310368	78.7261676	62.48762976
3D1 x 3D7	41.2	117.689935	95.46783108	72.33974667	120.7719914	100.0805483	79.40846226	131.0665609	105.8263777	78.498605
3D1 x 3D7	42.1	142.536875	113.4659381	81.20591	131.0386572	125.636	109.2982334	131.7406417	129.0642292	121.8810209
3D1 x 3D7	42.2	139.9364979	128.3161324	115.6285059	137.440968	114.3296267	93.23377083	139.1647589	122.5143214	89.06352571
3D1 x 3D7	43.1	125.9104712	76.26142996	59.77047929	76.55469454	60.85553348	50.81685893	85.7241218	60.48627	50.08355026
3D1 x 3D7	44.1	135.1108	110.750666	76.83469026	104.6690036	73.41648379	57.1443697	122.8837675	98.8061257	75.23764611
3D1 x 3D7	45.1	133.2493889	109.9353779	87.20926458	113.0734917	84.49419515	76.42185	122.0764024	88.34963524	73.34068333
3D1 x 3D7	46.1	142.40395	131.890406	115.3515167	121.0491152	79.19920595	64.28520524	120.4966191	86.00835635	77.68723691
3D1 x 3D7	46.2	103.9169082	71.74250997	59.35801967	84.64489352	70.5434067	56.17210909	106.3485062	78.6240503	62.95410857
3D1 x 3D7	47.1	NA	148.15	137.5893667	98.138	78.86825	64.451	100.2993333	91.31076667	73.99536667
3D1 x 3D7	48.1	142.6662994	140.8874869	112.6236155	123.0763619	76.02267111	60.51474286	113.9076738	74.77128333	56.8428
3D1 x 3D7	48.2	132.7349751	89.82751422	72.52160694	101.9810254	72.28780165	58.44153997	119.3008538	74.49031914	56.38209286
3D1 x 3D7	49.1	104.866192	78.25491788	62.8686925	81.2022176	58.65756659	43.28791212	120.2614794	92.3313479	64.8911899
3D1 x 3D7	49.2	140.9522	118.044747	83.390575	124.3443333	103.61325	91.84045833	127.3345452	107.1449267	81.60776809
3D1 x 3D7	5.1	NA	117.4724124	81.57235068	NA	88.41194371	68.96772462	NA	110.6734782	89.4195342
3D1 x 3D7	5.2	136.4810257	95.6565312	70.62825176	118.5702153	96.29915757	73.24583905	116.0068305	82.71073195	67.58075296
3D1 x 3D7	50.1	138.1254355	118.2649729	96.53738788	121.2600262	98.56399498	81.08327	130.294369	98.5071941	83.51318857
3D1 x 3D7	51.1	130.0670719	88.1732157	50.34719958	116.4704617	100.4002129	86.17416667	138.0303553	118.9777818	97.89570001
3D1 x 3D7	51.2	145.73354	126.3671257	102.5568233	90.03249889	71.14110791	54.30990857	76.13278778	63.09383397	47.22846278
3D1 x 3D7	52.1	132.1225505	111.8326229	83.38598294	98.35742868	77.84063683	62.95922949	130.1691795	101.9171043	82.38034524
3D1 x 3D7	52.2	145.1371458	138.333125	105.03245	102.8240625	83.50347917	64.64826042	112.9603583	75.57325	59.4755
3D1 x 3D7	53.2	144.7885	134.3973867	121.74444	136.7690417	104.0823125	79.99897917	134.1033438	112.0042042	97.91391667
3D1 x 3D7	54.1	125.7245038	107.8008033	96.61177667	95.91757393	77.67038	62.11016667	115.3815275	90.43367347	65.33060674

3D1 x 3D7	55.1	141.002075	133.5922761	116.6712278	91.99541047	70.9634	60.9066373	100.0572597	76.32626667	64.16302286
3D1 x 3D7	55.2	148.8433191	118.5140814	93.24240555	122.0431391	87.61767833	61.74316667	128.2704688	92.63234524	64.5694
3D1 x 3D7	56.1	126.2931533	99.61779999	90.84006667	72.43056786	59.49861429	51.64427119	107.7752924	80.46331472	62.45433939
3D1 x 3D7	56.2	143.7479312	117.6152838	84.11179417	112.3781365	89.13699818	67.38544667	103.633403	83.66261667	65.72513333
3D1 x 3D7	57.1	131.310289	108.6622561	79.3535651	132.1836299	106.4823254	70.67171929	145.8548178	108.3602286	80.40545333
3D1 x 3D7	57.2	136.3089227	101.7280267	68.54899584	120.1614797	102.9056305	86.48826857	132.9383929	108.1800977	86.24935
3D1 x 3D7	58.1	141.2475465	122.043441	103.3169964	121.9814364	77.82913603	61.93803531	115.7518433	78.52296357	62.99389238
3D1 x 3D7	59.1	137.9244676	120.4946433	93.36255651	109.0218792	66.16379343	56.00400833	100.3726743	67.16583788	53.87725555
3D1 x 3D7	59.2	132.60078	81.15803048	50.39966	82.54733766	56.68342857	46.95882857	89.71904167	60.40077976	49.0646625
3D1 x 3D7	6.1	NA	93.41434254	68.84771718	NA	57.95341286	45.03879365	NA	69.62722917	66.09039881
3D1 x 3D7	60.2	145.262375	130.9526875	98.77077778	114.6155667	61.89401428	50.57148667	102.9869	60.81056667	50.03586667
3D1 x 3D7	61.1	124.452478	87.75384171	65.20402111	75.40455953	63.22999915	55.97930339	91.75474965	64.07253333	55.5072739
3D1 x 3D7	62.1	125.9933989	79.39557802	59.69427473	106.5580009	74.18817016	51.14752198	128.5982939	100.96777	63.98341
3D1 x 3D7	62.2	124.4408159	76.57061762	65.62779167	78.01873371	58.81243131	53.98816381	93.67488833	66.53611309	54.87406667
3D1 x 3D7	63.1	133.1737375	118.0319444	88.19184653	112.8804551	88.40741286	60.84107429	126.1854829	82.33631333	61.41224286
3D1 x 3D7	64.1	NA	106.5487444	76.49073111	NA	95.31878207	71.59089	NA	66.42441441	56.28366806
3D1 x 3D7	65.1	129.4267262	121.809479	91.16494127	117.4588334	94.79097143	70.55424405	124.1708791	101.3354929	76.91656818
3D1 x 3D7	66.1	129.6917941	104.0463241	73.37228949	94.90311463	78.34021628	64.29825394	113.3980429	83.80335357	67.29928333
3D1 x 3D7	67.1	134.8926073	111.6200654	87.11802254	112.5281602	82.475125	59.93570571	137.7336072	85.81034226	63.99475824
3D1 x 3D7	68.1	124.1402381	92.11848571	70.13685357	82.35368809	72.62845714	63.716	118.4946875	91.217375	75.532125
3D1 x 3D7	68.2	144.06475	128.6390417	89.34975	91.018875	48.432375	39.62333333	83.05716667	56.93666667	43.477
3D1 x 3D7	69.1	NA	120.5982618	57.93401032	NA	76.29766893	59.30156048	NA	94.96980357	67.89861753
3D1 x 3D7	69.2	145.4181667	126.857975	70.13020357	91.71168841	58.1824377	46.55726159	87.02538254	60.70047619	51.30366667
3D1 x 3D7	7.1	132.5015709	91.46150114	76.02591778	96.82423341	87.68825182	75.63258667	127.2132401	106.1091593	82.56958333
3D1 x 3D7	70.1	138.9225604	116.7144049	84.30784231	113.9619414	76.95461298	55.08614556	113.8873173	78.59654936	54.82436239

3D1 x 3D7	71.2	119.4642417	74.62974097	81.52620833	79.34175128	69.86559573	63.92911905	92.60511042	72.98635417	64.59934375
3D1 x 3D7	72.1	135.1607585	114.8528589	90.19066742	118.8364471	106.132657	85.20056581	131.5317916	108.2671471	86.20108167
3D1 x 3D7	72.2	128.0205253	101.0333415	78.08121603	118.2341122	80.19439233	65.79632216	114.8081555	77.62225144	60.66963095
3D1 x 3D7	73.1	129.7474636	101.8440771	68.32869333	96.87696081	71.21999157	59.56131524	104.5816117	69.05347936	58.26187524
3D1 x 3D7	74.1	134.6715337	121.0559872	101.7737357	111.16535	76.40378692	64.89848382	114.5054034	80.10963308	75.49383833
3D1 x 3D7	75.1	130.3196065	104.7133066	81.85850476	109.3800674	94.57603175	81.46999905	112.1699701	94.66611691	77.97759524
3D1 x 3D7	75.2	129.1837541	85.17277272	77.54531758	90.40055411	77.68543802	66.83718475	98.68646601	76.41030705	65.0068375
3D1 x 3D7	76.1	133.5391	119.0110048	100.08689	108.7695333	86.52351619	73.31793333	125.5714333	84.69796	73.69570667
3D1 x 3D7	76.2	131.9574773	100.369903	79.43331976	131.2627192	97.31352948	76.97154722	138.3672406	100.5760292	79.03825615
3D1 x 3D7	77.1	112.2192307	77.31251155	83.31881957	82.85405324	79.43143167	63.31244961	92.77239034	74.17171094	62.79830923
3D1 x 3D7	78.1	116.7932006	87.36882299	61.67377469	83.61560857	68.0388633	50.16998321	104.2035277	76.88833658	60.39997778
3D1 x 3D7	79.1	133.17	112.7085	72.31582222	104.7686333	74.36303333	60.6129	111.141625	84.8306875	63.855875
3D1 x 3D7	8.1	121.8492468	77.59015334	78.16812475	85.07275989	64.00210096	53.89652172	85.06553007	68.07385397	59.06951238
3D1 x 3D7	8.2	139.2425217	129.8859946	93.23705232	104.8151985	71.12934389	59.12174048	95.33764016	75.46994696	59.01196341
3D1 x 3D7	80.1	116.1126154	65.84912121	57.30694944	96.54146415	66.83216952	58.0993	123.5218227	87.43271222	64.62689278
3D1 x 3D7	80.2	115.7311444	86.55267833	73.24845952	95.45435464	91.27937045	78.71675	94.78494762	74.23724643	75.01395
3D1 x 3D7	82.1	127.1834967	82.67500658	50.17296659	83.42189909	54.64328762	45.9118	97.6490682	61.74469299	51.14766667
3D1 x 3D7	82.2	130.8845429	78.89452788	61.55035276	117.0183546	93.47223095	83.01379028	126.496826	98.93255507	76.715925
3D1 x 3D7	83.1	116.5425241	76.03682893	69.55818864	84.18277488	63.14270772	52.25750877	121.3914298	87.54078062	67.9946998
3D1 x 3D7	83.2	NA	NA	NA	71.722	51.673	41.856	NA	NA	NA
3D1 x 3D7	84.1	122.5390833	113.2460833	114.0093125	138.799919	118.12185	98.2186	142.0643533	126.87885	108.80015
3D1 x 3D7	84.2	139.9358867	129.704392	114.2944071	91.39070024	65.82530643	57.37835773	80.60586943	63.94896263	56.81418595
3D1 x 3D7	86.2	130.90975	83.08857917	66.333125	86.9205	59.06973667	50.79038	NA	NA	NA
3D1 x 3D7	87.1	119.4662	72.2639092	53.68916104	69.94410834	55.68082692	49.76068006	82.79014444	54.46703643	49.11392333
3D1 x 3D7	88.1	128.5371605	121.11342	97.80528911	84.91981648	69.77668316	61.44299154	86.95841158	68.23619879	60.30950404

3D1 x 3D7	89.1	130.0942326	124.4101288	113.1728922	117.874396	82.51538977	70.30835556	137.4089416	109.1990627	72.33131667
3D1 x 3D7	89.2	123.2666667	132.5490833	123.0581667	117.9218691	91.89802858	69.80549524	126.73456	101.3191	83.42284333
3D1 x 3D7	9.1	127.5061178	96.33129728	74.34117143	99.29095116	74.66140208	59.44418651	107.4584133	81.72285143	60.24218333
3D1 x 3D7	90.1	124.1059409	78.02744416	59.96323818	85.30534091	60.62567111	54.19336667	103.7316753	75.44853333	63.92218
3D1 x 3D7	91.2	131.7397859	114.3827143	102.2866889	114.106	97.85499893	83.90320833	127.8512286	94.42201667	77.04988333
3D1 x 3D7	92.1	134.1069473	122.2893976	82.2742621	103.2878176	80.5254809	56.65332667	93.93645363	73.42889835	56.39866539
3D1 x 3D7	93.1	126.0612453	106.4070198	73.60460364	112.6845277	98.69777475	83.41178572	126.2278936	106.2318507	94.98958333
3D1 x 3D7	93.2	140.4458789	118.9459466	87.0450144	111.8445034	79.66245576	64.34473691	106.6398897	89.55186246	78.64313333
3D1 x 3D7	94.1	116.3552507	68.77585889	58.79182093	85.2101035	71.15724824	54.09736947	106.9119375	76.44265947	65.98349275
3D1 x 3D7	94.2	134.3618664	123.5504928	100.7884533	128.7292414	108.15678	89.42358889	138.1719361	109.5675905	91.57035
3D1 x 3D7	95.1	134.8024131	129.9026638	122.6036508	135.5167899	125.2594775	110.4964441	123.9543039	111.56085	93.16291818
3D1 x 3D7	95.2	141.1246189	124.4959078	100.0641096	94.06768127	59.70294939	53.56043918	82.77282723	58.54581293	53.7420254
3D1 x 3D7	96.1	121.5177962	63.45879472	49.96683043	68.57126972	55.61269659	49.64093087	95.12954237	64.49154222	56.6136747
3D1 x 3D7	96.2	124.296238	86.67959407	70.29780167	80.44291685	61.8958596	54.94391905	84.71772647	59.71470746	53.61638809
3D1 x 3D7	97.1	138.1796607	100.3891116	68.43869268	119.1502393	78.36920063	58.97555714	136.962099	92.29469121	68.41900897
3D1 x 3D7	97.2	128.5034806	102.2765582	64.6266859	115.3046029	91.96235268	77.66428572	104.7856744	76.8605727	65.12964667
3D1 x 3D7	98.1	141.210134	114.906873	92.998	89.02942909	57.17405951	44.84483266	83.10620476	55.00385238	47.99129667
3D1 x 3D7	99.1	NA	110.6977885	84.6508166	NA	74.19974585	54.27046907	NA	93.19490873	70.52173047
1A5 x 1E4	A1.1	132.5790795	63.51353985	56.04975215	117.6858372	99.76006338	87.54909508	106.8396329	65.5010146	73.15050697
1A5 x 1E4	A10.1	142.9830019	134.5393723	100.0858318	106.6643368	63.99398022	51.21225476	108.0828536	86.66337922	93.15221478
1A5 x 1E4	A11.1	127.6758367	90.22278341	98.99613553	100.1444955	73.21620833	59.40346667	128.8109878	86.88855994	72.76998893
1A5 x 1E4	A11.2	133.1738894	86.42442276	87.7914685	76.62979025	60.10945913	52.29096156	117.7366153	97.33199177	85.27899593
1A5 x 1E4	A12.2	144.8502917	137.824848	118.5203617	135.7655886	92.82833099	86.64696114	146.766013	143.6718542	127.8917814
1A5 x 1E4	A13.1	140.6154299	114.7669524	113.7456037	92.6217908	99.09597842	70.50643029	109.3177058	101.9236541	77.0169961
1A5 x 1E4	A13.2	134.5581563	121.4889278	116.2314639	74.44483159	65.60741548	72.87964286	122.1446957	89.13732001	75.79855

1A5 x 1E4	A14.1	138.6986332	133.9625169	124.2767459	135.2878282	91.4913371	68.21450981	66.29180588	108.4765724	101.2765516
1A5 x 1E4	A14.2	138.37409	116.2342862	109.2732059	94.84636191	64.25854329	47.00154242	144.1510614	116.6752539	98.92374286
1A5 x 1E4	A16.1	135.5075833	129.0794425	129.0404991	100.9680229	79.89036855	56.38064881	147.2335933	134.7606533	117.8988333
1A5 x 1E4	A16.2	138.1434167	91.82944448	85.69151786	125.6431238	98.42976667	74.06099714	154.02225	144.7092222	121.06525
1A5 x 1E4	A17.1	133.1909728	128.9427935	110.7885259	83.72118406	73.47524402	44.71963413	132.7753152	115.5283863	108.2860229
1A5 x 1E4	A18.1	127.3506712	115.7412573	120.2684069	74.74213354	72.7874175	46.99657182	104.4959783	73.88110567	66.34136313
1A5 x 1E4	A18.2	129.1399439	74.41897701	82.52629193	81.07931155	54.47141143	54.61966667	117.473407	68.28897142	69.31737708
1A5 x 1E4	A19.1	137.3291517	129.1016152	122.1713675	114.8750199	65.84285945	55.52193956	119.6722083	100.0062796	92.99184349
1A5 x 1E4	A2.2	133.7945333	96.60595476	64.67851905	90.66408667	65.67700333	49.69129333	129.5042	111.9315	87.016
1A5 x 1E4	A21.1	131.0761357	87.36033167	99.8336711	77.3623032	77.1727873	59.84112778	82.71865221	82.28084667	67.12995879
1A5 x 1E4	A21.2	139.89555	121.0532072	118.8668214	85.39833333	85.99943333	55.02743333	97.68191667	104.1765834	68.83288889
1A5 x 1E4	A22.1	124.4123333	104.7658333	82.51233333	111.5723611	67.17516667	53.61016667	133.24	118.28	107.0805
1A5 x 1E4	A22.2	143.0424025	133.3678388	118.4917771	113.454642	69.51728251	53.39574066	142.5509417	99.49903252	87.42445675
1A5 x 1E4	A23.1	138.7694893	120.1341026	103.3632918	103.015939	77.35675812	66.11889222	125.6242972	91.72923656	89.07978099
1A5 x 1E4	A23.2	137.7155392	90.11965742	67.08916712	104.5964524	100.1020111	100.7403542	148.0114006	151.3291591	149.3876882
1A5 x 1E4	A24.1	139.1906869	82.51778271	72.20053347	121.4889598	105.8941424	97.72690001	125.1300802	108.1280397	99.58680903
1A5 x 1E4	A24.2	145.1315	111.6883334	97.05383332	116.8624583	102.83025	73.87679167	145.5495	146.94375	133.596375
1A5 x 1E4	A25.1	145.9211531	122.0976939	95.87569646	101.2892737	89.65724907	78.92249136	130.3232446	103.489721	110.4403998
1A5 x 1E4	A25.2	133.3692643	99.25903834	105.4285083	90.33938416	76.71826667	66.0684	143.825	117.5645933	99.17278
1A5 x 1E4	A26.1	135.0252967	94.7338896	104.8950762	78.05405359	73.46566768	55.37960905	129.09845	86.65133091	107.3535159
1A5 x 1E4	A26.2	142.7656382	107.981773	98.26837192	109.4252095	79.87659524	62.435	158.6042611	142.8583504	125.19635
1A5 x 1E4	A28.1	127.4782045	109.6338012	109.1889528	66.3117875	57.32428614	45.95409984	108.8615133	53.98129444	68.94106667
1A5 x 1E4	A29.1	127.9265542	113.7188785	103.2456775	78.40115632	63.75651326	47.65048667	106.6025515	96.70824022	65.70786497
1A5 x 1E4	A3.1	132.6597167	97.88726001	80.17256667	101.0009467	64.45957333	51.65788	128.2988981	56.36165333	84.67344524
1A5 x 1E4	A3.2	150.6098832	140.2116994	118.1365329	138.9273489	101.1325535	71.10404692	138.5841527	131.8159045	93.44056161

1A5 x 1E4	A30.1	151.6194667	135.9128616	117.2946396	87.00642338	79.69179206	50.802785	135.2840417	120.7787544	77.97255556
1A5 x 1E4	A30.2	137.0505126	115.8981977	108.0665547	98.05828578	64.91121667	53.51093333	135.9968012	86.10851366	69.28510392
1A5 x 1E4	A31.1	143.6752758	106.7417029	96.71028477	89.94646667	75.48881191	59.91456667	160.9419334	134.2334167	103.78175
1A5 x 1E4	A32.1	142.0272354	131.6329133	117.8287005	90.59721154	83.59696667	67.82681	127.2505748	126.5577364	100.3617333
1A5 x 1E4	A33.1	145.7235333	113.524955	90.12016667	109.6034647	99.29941506	86.96640095	134.4067333	127.51079	102.10654
1A5 x 1E4	A33.2	138.4803667	116.583269	104.6886898	101.2511357	67.15950955	59.63854788	119.7407047	95.43281282	81.59951056
1A5 x 1E4	A35.2	130.7345987	112.9385192	112.8132933	90.26866116	86.43454351	68.38337394	146.7870439	134.38783	109.7301907
1A5 x 1E4	A36.1	143.1481689	140.9159657	126.3872468	132.3760106	79.05037815	56.64825178	107.2926933	105.2822248	88.01124857
1A5 x 1E4	A37.1	132.4328367	106.1139162	101.5665065	88.22397422	83.11985341	46.52782727	109.0477078	111.4371116	74.36529524
1A5 x 1E4	A38.1	151.6616333	143.3072286	129.0137833	116.8726844	85.70714539	47.74022143	97.70244	111.5696067	75.79465417
1A5 x 1E4	A39.1	142.3779717	100.6595933	83.25163722	126.7278746	127.627911	105.0282276	165.6963009	155.8281622	131.7053457
1A5 x 1E4	A39.2	138.3859	119.5689	93.11612667	101.123225	74.46750833	60.41398333	108.95705	116.69864	105.4121667
1A5 x 1E4	A4.1	110.5555249	69.87343665	69.66528988	96.31039826	121.9455877	119.0412329	147.9205894	133.2705481	140.1868579
1A5 x 1E4	A40.1	147.2704205	141.3243064	122.5631598	130.0601929	106.3686648	61.93760833	144.4268333	129.10238	85.54543333
1A5 x 1E4	A40.2	129.1255778	84.85927951	67.89256682	125.1018135	123.6736914	109.3732343	149.5326674	138.7090651	109.08101
1A5 x 1E4	A41.1	139.7002614	117.7233675	104.15073	74.18158386	64.11071678	51.12194667	124.5683955	109.8487445	78.1695987
1A5 x 1E4	A42.1	NA	NA	NA	NA	NA	NA	NA	NA	NA
1A5 x 1E4	A42.2	132.6881369	87.73847854	69.81695714	90.6378026	74.69791511	65.12042619	140.2226088	137.1479287	124.123999
1A5 x 1E4	A43.1	140.265125	137.0129792	124.8567917	135.7705	124.1853333	77.39696667	153.792	129.3958	101.4582
1A5 x 1E4	A43.2	143.5481167	131.0605181	122.2229895	122.0099324	99.95770666	80.58088524	122.5364829	87.29904273	91.42494152
1A5 x 1E4	A44.1	148.6290934	146.0935148	122.0924748	101.6941934	83.35170286	75.08639524	130.1571198	109.3873122	95.5128357
1A5 x 1E4	A44.2	134.4996991	87.59516346	71.93589033	114.5356663	119.348498	115.9900301	144.4155095	142.3938027	138.7617523
1A5 x 1E4	A45.1	144.9642917	113.28578	82.64723333	125.8014576	112.8108019	95.06084667	162.3703786	163.50136	148.1152
1A5 x 1E4	A45.2	141.3185667	131.8298144	117.4909179	96.25123021	68.70987293	57.23838738	146.4312421	132.4308826	108.2757133
1A5 x 1E4	A46.1	135.5093449	100.4376277	100.0153823	80.11675762	75.78519049	49.04425626	137.8661671	125.9194243	98.19198129

1A5 x 1E4	A46.2	137.7198833	109.3041105	93.9163638	86.72292121	64.60196162	58.24560952	126.7294738	105.1249029	87.81838667
1A5 x 1E4	A47.1	137.7872733	116.3291876	100.0947738	104.8185105	76.61460778	57.92884068	142.5591921	129.3636956	114.5495083
1A5 x 1E4	A47.2	142.0822783	131.5754548	114.0051174	106.2476641	88.69771389	65.16922857	137.117671	136.4285155	122.9240967
1A5 x 1E4	A48.1	131.8528889	129.8186	111.2033833	82.30006667	72.7424	47.5742	117.44325	109.1277867	70.44095
1A5 x 1E4	A49.1	144.0360333	105.3717533	98.88207936	119.5317333	92.00655	70.26708333	139.8451	128.6395667	102.08793
1A5 x 1E4	A5.1	127.6277718	73.14858054	89.52311273	101.7392523	72.24244167	60.50827857	139.5365542	100.8558421	83.23721442
1A5 x 1E4	A50.2	138.3679194	117.7755948	108.19339	105.3818867	81.13446667	69.46616667	148.6895625	136.74825	130.0129667
1A5 x 1E4	A51.2	134.9260333	132.9756857	124.3790071	128.274603	79.17764286	44.30346667	92.30891667	100.493725	60.62627333
1A5 x 1E4	A53.2	137.8274533	127.4843683	124.565385	101.324893	63.30901714	50.21866667	115.1636467	101.11245	86.37755
1A5 x 1E4	A54.1	132.415	122.4978333	107.9678333	125.1972084	88.47916665	71.74841667	124.8423333	112.5528333	93.0335
1A5 x 1E4	A54.2	122.9869589	117.3784516	111.5344056	80.88426667	75.64437619	60.18197143	129.93235	119.0513889	81.52066667
1A5 x 1E4	A55.1	142.418575	90.408695	88.52519278	89.47980556	83.7371	64.88553333	114.7867333	98.26295	76.46991667
1A5 x 1E4	A55.2	135.1555	107.0047	111.5239	84.33645833	82.42841667	44.81225	106.942625	56.35675	83.65645833
1A5 x 1E4	A57.1	131.9742191	85.32011667	58.15598333	103.9768238	80.77416429	66.37371	117.5860333	117.7001	90.777875
1A5 x 1E4	A59.1	127.96256	114.0855933	96.89270665	79.91454167	81.53592857	61.7538	139.5225917	119.3076167	79.76961667
1A5 x 1E4	A59.2	132.5115	144.2213334	125.201	121.147875	100.214375	68.043375	163.802	142.084	120.7175
1A5 x 1E4	A6.1	132.7965971	130.4159867	119.3923019	74.84744893	69.4330004	60.62271333	126.506097	141.3996621	143.7859211
1A5 x 1E4	A60.1	145.78355	119.3120317	99.76969842	107.4881528	87.20307333	72.61015	153.5468724	143.1628584	107.382445
1A5 x 1E4	A60.2	131.0936047	84.02562233	77.72023658	111.9049391	99.26510683	84.26126	135.8522742	126.2155284	110.2238298
1A5 x 1E4	A62.1	NA	NA	NA	NA	NA	NA	NA	NA	NA
1A5 x 1E4	A62.2	139.6797801	124.14506	115.8640572	80.90529689	83.80021234	59.68796889	128.8021144	128.9199584	94.39715491
1A5 x 1E4	A63.1	128.9855294	114.1023754	110.6916741	96.86473332	89.50750335	60.19971524	126.8765	101.96475	82.215625
1A5 x 1E4	A63.2	143.1964801	131.1013224	121.0491986	126.481857	95.21814883	64.3777141	120.996454	107.6633833	86.29578408
1A5 x 1E4	A64.1	129.211285	92.07845761	90.28865065	106.2831761	76.93611966	69.79676278	131.5425586	106.0642833	83.11237
1A5 x 1E4	A66.1	133.4675407	116.9485872	110.0947785	95.81794348	62.53737672	53.71243071	129.2341556	108.2874429	85.72849524

1A5 x 1E4	A66.2	134.7839333	90.15729001	66.9965	128.4970965	95.47460714	73.06184643	148.285	143.60954	130.4062
1A5 x 1E4	A8.1	125.4681583	120.930332	104.5378498	89.98524407	56.86299865	45.65538413	129.5496293	112.0432488	89.11155095
1A5 x 1E4	A9.1	119.4421308	78.12743333	78.86890385	62.04392204	57.30012535	71.60754842	125.0787143	117.6950194	89.0731049
1A5 x 1E4	A9.2	130.35401	75.25609841	67.92827957	119.3472766	94.03320782	71.80053214	157.7818671	144.7443084	123.0305747
1A5 x 1E4	B1.1	128.5080333	109.614325	100.0280667	99.58846	86.51501333	61.303	109.0359375	105.55452	62.229
1A5 x 1E4	B1.3	128.0616947	96.35146621	94.00783356	81.64581055	70.25627641	63.28457603	144.9114646	128.7444737	93.80936044
1A5 x 1E4	B10.1	129.7614788	111.3268313	100.7267792	76.29773133	60.95323164	44.08152987	117.9937711	86.2285683	70.93620545
1A5 x 1E4	B10.2	135.2359228	106.7759484	102.1226727	101.368487	80.73537521	66.79540805	125.1993446	103.3987878	98.62729286
1A5 x 1E4	B11.1	141.7384965	115.6457832	98.3688306	82.5369656	67.69005643	59.29933333	119.9751621	103.9971897	88.38618017
1A5 x 1E4	B12.1	141.5255035	132.42609	121.9116829	139.7456425	95.81670814	62.2581548	108.3949184	111.6018044	85.49447619
1A5 x 1E4	B13.2	130.0131	120.1087667	110.36835	84.93360571	67.01679333	57.68346667	118.7979	122.4579	89.53116667
1A5 x 1E4	B14.1	125.1453322	86.09613634	97.7787486	115.6244924	105.1143541	89.08824666	114.9433221	128.2443436	118.5328107
1A5 x 1E4	B15.1	NA	NA	NA	NA	NA	NA	NA	NA	NA
1A5 x 1E4	B16.1	129.7618295	134.448456	103.3734042	65.03256726	60.93242853	52.00648409	73.53937857	94.49350167	56.07600714
1A5 x 1E4	B16.2	133.5910267	74.03923035	69.45972476	122.0592316	114.44024	106.36536	151.0714667	147.8694422	143.1907333
1A5 x 1E4	B17.1	134.194713	86.2012914	78.49137286	78.77827026	66.37804532	62.76420778	134.2188009	110.7325069	92.63421056
1A5 x 1E4	B17.2	137.9435833	101.0213756	91.02327143	98.70439334	67.32458667	54.1888	144.2077778	130.3613533	108.3912667
1A5 x 1E4	B18.1	144.6246333	141.1399	97.1924	134.7702	103.47165	100.9975833	158.1511	159.7602222	143.9955
1A5 x 1E4	B18.2	134.8159594	110.8981636	108.906787	101.8989477	81.14125481	63.77520404	126.5698357	135.5055404	105.9267589
1A5 x 1E4	B19.1	136.1340571	125.9591277	114.6866234	103.1804196	80.34716845	50.12216901	130.2656447	106.6616476	88.86485843
1A5 x 1E4	B19.2	141.0155083	113.1531694	108.5638875	107.0559149	95.47190223	85.45019024	146.2291833	141.7172598	139.7296997
1A5 x 1E4	B20.1	143.55828	113.5642566	67.96673286	118.3602919	103.6283588	84.93420309	145.6275634	135.632825	123.7297079
1A5 x 1E4	B20.2	139.1774103	95.37115429	78.74335848	119.1122956	84.16932738	75.4789	148.5363373	138.4223522	126.9171271
1A5 x 1E4	B21.1	161.281	NA	NA	112.739	96.318	94.502	147.4275	152.4523333	142.8315
1A5 x 1E4	B22.1	132.7680006	83.11662486	75.07489622	112.2363388	117.160774	105.0957764	125.4875486	122.7314557	113.0768567

1A5 x 1E4	B22.2	139.1952627	123.481887	115.0993309	111.0737396	91.70052183	81.72054181	148.61088	144.0961076	138.16405
1A5 x 1E4	B23.1	137.1044352	130.9171317	117.246215	100.7991559	89.16327714	76.867305	135.9359895	137.8761842	140.965961
1A5 x 1E4	B24.1	132.4782599	130.605809	117.1873413	85.14666827	68.91424936	49.44348434	110.4923662	91.05316673	80.97807053
1A5 x 1E4	B24.2	137.2404799	134.8213389	124.7277361	95.47497071	99.256425	61.31818333	117.2651516	87.30823556	96.51404857
1A5 x 1E4	B25.1	130.7955455	90.37751187	92.60034262	82.10492569	61.00173175	54.13857283	110.164525	65.89980064	71.12189611
1A5 x 1E4	B26.1	132.1237159	122.4718224	119.1080457	98.58230511	86.70305286	64.60113429	151.1460755	141.3617091	107.3603071
1A5 x 1E4	B27.1	138.085921	102.9919718	100.6205989	108.3601161	85.31249987	77.30130892	142.9945053	116.1073948	76.95035833
1A5 x 1E4	B28.1	141.1243435	116.7684836	91.97350862	100.0655125	88.89748809	80.6754	144.090426	137.5373225	130.2682758
1A5 x 1E4	B28.2	143.23725	89.97300333	74.10408	95.73631429	65.6586	60.23148	143.35475	121.8797333	119.3861
1A5 x 1E4	B29.1	138.4255	131.5315	115.6945	109.6045	75.2175	58.4075	140.9045	133.4095	101.734
1A5 x 1E4	B3.1	132.6958875	112.90655	98.81697	89.40645167	64.63610667	50.94798667	115.2772333	125.364625	76.91194
1A5 x 1E4	B30.1	137.9884309	96.2201925	77.0611681	100.7175396	105.8821973	84.97631809	132.713852	134.526945	114.8883433
1A5 x 1E4	B31.1	138.1798667	117.766325	111.62	102.32775	91.720125	60.218	127.274	123.789	103.3935
1A5 x 1E4	B31.2	131.6718945	120.8216799	113.6070354	113.2249022	104.7102111	89.53248	157.8152	128.3234738	108.36704
1A5 x 1E4	B32.1	138.7214013	75.69165808	71.08225076	94.63540622	67.40480401	55.67591111	122.7344765	107.4379494	88.13497974
1A5 x 1E4	B32.2	135.5626167	134.5339833	117.1776733	95.25105833	70.01573333	59.57763	151.197	153.7325	136.5776667
1A5 x 1E4	B33.1	145.205288	137.2626367	122.3737256	96.48070661	72.52905247	50.95451154	137.75206	126.8075433	93.34935953
1A5 x 1E4	B33.2	129.4767367	84.21919384	81.64606525	118.055901	67.63995833	53.07055556	133.6182843	65.72365585	61.25066667
1A5 x 1E4	B34.2	129.5802086	131.3973956	116.418814	106.0849396	66.90673429	55.45728333	86.79510417	103.5976976	73.20078333
1A5 x 1E4	B35.1	138.1842385	74.32456511	69.73116667	111.6035597	75.78205455	62.16727667	141.8544567	143.3892196	122.3254108
1A5 x 1E4	B35.2	135.4471357	128.4571441	115.5542085	69.391025	64.1969	60.39947714	127.42976	112.3707067	91.62565238
1A5 x 1E4	B37.1	130.8659178	99.83010384	68.32395179	87.25136667	65.12884889	57.67602857	147.279525	119.3171667	117.7047958
1A5 x 1E4	B37.3	137.8295732	126.0171412	97.15215625	109.0527674	112.7640119	94.7834375	155.66806	154.8043276	145.4118033
1A5 x 1E4	B38.1	139.4165383	90.644215	95.60740714	113.5009139	79.06933333	67.25332667	144.1964667	77.4182	79.48383333
1A5 x 1E4	B39.1	130.5622126	112.0812618	105.8642321	111.6439901	97.40146021	95.1614724	136.5257681	141.2983541	136.4894674

1A5 x 1E4	B39.2	139.6081707	133.3632509	112.6192921	124.8674568	104.4782996	98.44952666	145.1739729	140.84618	132.1983088
1A5 x 1E4	B4.1	143.7639931	116.029286	94.53149554	103.4939681	91.75732202	73.96416714	144.9810334	132.5640737	111.5671869
1A5 x 1E4	B4.2	131.2955	117.5425	105.1417333	96.475405	91.82979166	66.51614167	129.89125	101.298625	101.735375
1A5 x 1E4	B40.2	140.842375	111.5995	77.0483	82.96198333	70.7223	57.70006667	156.3395	155.563	131.889
1A5 x 1E4	B41.1	134.516229	86.44480663	NA	112.5749542	92.64829143	80.1234005	140.2502783	127.1528203	120.3244413
1A5 x 1E4	B42.1	138.3251411	108.6528	82.67801667	122.85721	129.11522	126.6996667	164.1462029	163.6769167	135.62272
1A5 x 1E4	B42.2	124.0168373	82.61479515	78.6627503	72.72879522	68.23400212	66.48560664	128.7297522	109.9260841	97.19888542
1A5 x 1E4	B42.3	139.0953334	108.9238333	69.73733333	114.30975	93.47453	88.59615	157.1938333	155.2893333	126.9715
1A5 x 1E4	B43.1	133.9527683	109.2556533	104.7310197	82.57884483	65.07837936	50.98952084	150.2178182	122.6348206	114.1586252
1A5 x 1E4	B44.1	138.6275192	94.18028024	NA	103.9329205	94.62070348	80.38115673	140.5590853	113.6569681	102.418449
1A5 x 1E4	B45.1	141.4474554	134.1595908	123.0438228	132.423774	82.70684485	56.22325095	138.007628	122.306676	89.36502035
1A5 x 1E4	B45.2	145.98095	99.9948	73.1593	101.7535133	67.3481	58.01701667	151.1516667	118.6985	105.15125
1A5 x 1E4	B46.1	137.536422	118.4371207	95.99732728	96.34851139	67.18737949	61.8608419	134.4066159	83.1526283	82.89940476
1A5 x 1E4	B46.2	129.8602201	126.1781683	113.1956278	103.2035306	73.75895549	55.15816306	123.8968548	122.8148559	109.6998867
1A5 x 1E4	B48.1	139.5879353	106.4159279	100.5809852	84.82881424	72.57909064	51.74898064	141.7865768	138.7888194	110.5240635
1A5 x 1E4	B48.2	125.8614879	99.08073353	85.89214108	113.1137093	91.55285228	75.291	116.7033886	95.15253005	96.09366195
1A5 x 1E4	B49.1	144.1564905	128.2497613	107.9341162	102.2783771	64.61570659	55.92975	151.9002381	125.6348067	109.9867951
1A5 x 1E4	B50.1	123.5875	117.2452467	109.7721067	87.98448667	65.15776666	46.90643333	124.8872833	80.4757	63.03908667
1A5 x 1E4	B50.2	125.7868333	115.5642397	112.0851997	82.99202222	81.74185985	60.19221778	125.9008964	106.0321041	84.75310131
1A5 x 1E4	B51.1	133.1131879	105.2627535	101.976049	104.1805617	68.71855487	62.54118571	133.9954808	137.3796131	121.9296723
1A5 x 1E4	B7.1	141.9079791	137.258303	126.6419826	106.7360542	105.71044	65.07901571	133.7761673	123.5348667	90.19001334
1A5 x 1E4	B9.1	138.2268579	131.4043573	116.7382508	106.0870073	76.30081809	61.07464762	115.75994	115.2124667	67.37472
1A5 x 1E4	B9.2	138.9647485	106.3165457	76.89690721	75.47834298	57.71783262	44.56008222	134.5449572	97.43468388	79.94560434
1A5 x 1E4	C1.1	127.5245389	73.64792524	59.50207372	102.8008739	71.10956455	54.34925	142.7621974	118.399576	108.3362155
1A5 x 1E4	C1.2	137.13525	124.0585	119.613	102.7106667	88.32183333	62.7545	125.93325	117.778	97.4685

1A5 x 1E4	C10.1	135.7489835	102.8437168	104.2433061	123.0122465	74.51611475	41.02894311	155.2625376	106.4701828	83.57872073
1A5 x 1E4	C11.1	145.2241476	119.57814	96.4371875	109.1291333	104.2816667	97.54918	166.7965556	163.2025167	155.0664444
1A5 x 1E4	C12.1	141.5438286	116.2443784	111.7239715	120.0890667	90.20245	60.10815889	135.2768423	105.329946	94.72405238
1A5 x 1E4	C12.2	NA	NA	NA	NA	NA	NA	NA	NA	NA
1A5 x 1E4	C13.1	127.5553165	89.29952628	94.7013492	80.26816015	68.84060641	55.70192794	159.1511333	115.75638	107.5439222
1A5 x 1E4	C14.1	140.0680891	94.98631877	95.71696463	109.4511264	80.23738481	62.09112641	147.3266742	114.4920824	115.2885183
1A5 x 1E4	C16.1	138.7354529	84.7587192	72.29394432	95.850793	62.87359295	53.86069818	132.718665	101.5904968	86.95619487
1A5 x 1E4	C16.2	123.876625	101.60025	105.815	103.3781	70.4133	74.5273	128.853	98.4185	96.89175
1A5 x 1E4	C16.3	138.3346675	96.76493328	93.44512333	90.91069442	66.09414475	62.871395	126.9855567	74.22889206	88.79056627
1A5 x 1E4	C17.1	142.7215644	132.9026265	120.3982217	91.45934417	77.86443056	59.03718127	152.5392766	139.4103952	131.6338833
1A5 x 1E4	C19.1	146.3476967	127.4464732	109.5807119	88.94753714	75.80795821	58.79238827	144.7334518	139.387173	133.4559239
1A5 x 1E4	C2.1	131.7234601	95.27911065	101.0985851	105.8110037	71.64244635	63.26905714	153.0798486	115.353605	93.98769714
1A5 x 1E4	C2.2	130.1316827	91.51264895	73.99062026	114.6030851	89.83661606	62.46781857	121.0379009	99.52963007	81.57816282
1A5 x 1E4	C20.1	129.6544431	97.80093213	93.48593444	81.9401527	54.928691	41.28602424	NA	NA	98.216
1A5 x 1E4	C20.2	137.724285	107.2917967	99.59888166	102.029189	79.40390357	60.38620857	154.0035341	125.6119964	102.2982833
1A5 x 1E4	C21.1	119.3093315	81.55554787	80.78540189	89.29023655	49.10955738	43.15654314	137.2280196	108.9856075	73.88662927
1A5 x 1E4	C21.2	126.0507262	108.9028131	108.8525581	86.17175569	70.07317212	54.06693333	156.7422	135.6315667	114.9175625
1A5 x 1E4	C22.1	142.8353514	117.1007588	107.536309	78.11042204	69.69702157	60.36667167	156.3499396	133.8794199	142.9835
1A5 x 1E4	C22.2	144.4536667	130.71475	87.423375	135.0869333	117.45825	102.525875	148.87225	156.6655	137.7085
1A5 x 1E4	C23.1	136.2338078	117.4659515	103.4335346	91.27405506	53.80671236	52.37190333	153.576576	114.5450588	101.9189507
1A5 x 1E4	C23.2	128.4643228	95.25976012	102.7280243	126.1464875	90.05688529	80.27681131	124.903625	110.5636667	78.76466667
1A5 x 1E4	C24.1	134.0582105	69.64222597	51.00982212	100.8950475	76.25730486	66.52643405	180.047	106.9842071	100.3839756
1A5 x 1E4	C24.2	132.1045	105.756875	73.69875	137.6296458	128.115375	115.4746042	159.4175	162.1505	163.062
1A5 x 1E4	C25.1	136.0160391	70.41153283	60.80436321	79.59059306	57.87156142	48.97869889	115.7216251	85.76035522	70.39166526
1A5 x 1E4	C26.1	133.9156975	118.9206149	82.16869582	65.68936975	67.49848356	77.6047823	163.5146433	150.9274457	144.0472689

1A5 x 1E4	C27.1	132.8423309	80.06154592	72.90514286	94.55936737	72.61621597	59.69446429	168.8231659	151.2485923	131.9449827
1A5 x 1E4	C27.2	136.3252721	131.1158003	118.3307716	83.82705264	64.8126165	53.34550718	139.4995522	120.2966596	92.59595913
1A5 x 1E4	C28.1	130.479069	116.6854858	113.4424294	76.85163926	65.87534538	51.76089143	158.69825	91.24401118	84.80298818
1A5 x 1E4	C28.2	130.1669992	73.85098107	64.27348104	117.9577885	110.2962957	96.22513	130.6200046	101.4368999	89.23253304
1A5 x 1E4	C29.2	139.6823971	115.5535913	116.9444255	116.1837258	101.136391	69.34035233	138.7755714	71.40249167	94.63641666
1A5 x 1E4	C3.1	140.3301186	103.9566103	85.83152333	100.8894152	79.92450564	71.98861778	160.2839909	152.0471128	134.8799714
1A5 x 1E4	C3.2	134.5841893	83.11963697	64.99290286	134.9947094	121.7876562	119.1931122	140.8284822	132.8992663	140.1654556
1A5 x 1E4	C30.1	151.5715786	136.1510761	125.9742967	130.5114987	71.17414216	64.29031753	152.0555	104.8751432	87.51754444
1A5 x 1E4	C30.2	134.1023547	101.4425202	104.2191254	103.6012222	77.6689329	69.2841875	105.0896244	108.153301	100.4925355
1A5 x 1E4	C31.1	147.838	132.5845	127.7885	104.2463333	63.38866667	52.29133333	NA	NA	NA
1A5 x 1E4	C32.1	132.71918	123.9616664	117.9231526	111.9334998	73.90874889	58.08241587	NA	105.920547	79.74651167
1A5 x 1E4	C32.2	139.7737333	108.1751167	101.287285	90.73287333	83.4193246	69.37380476	155.6486189	146.256621	133.6010238
1A5 x 1E4	C33.1	137.1705296	108.964665	89.44336111	105.4674609	72.76378988	71.56895119	139.1918049	99.99494887	74.45681429
1A5 x 1E4	C33.2	139.369881	102.1718115	87.10281092	120.0569589	113.02144	116.1991933	140.0506445	112.9342417	125.7985167
1A5 x 1E4	C34.1	147.5775536	136.6016341	125.677171	84.93427605	74.52785824	63.50498952	149.1029315	137.8857736	133.915305
1A5 x 1E4	C34.2	130.321625	123.4813333	120.4628667	86.26383333	88.465625	56.985	144.7725556	87.6595	50.91175
1A5 x 1E4	C35.1	142.3662227	128.8896975	109.3473719	113.666039	60.78977044	47.9867019	108.5758444	84.48499945	85.3406602
1A5 x 1E4	C36.1	127.0308333	86.16389583	86.6175625	72.6855	81.30425	96.0484	162.3046667	150.5595833	154.282875
1A5 x 1E4	C38.1	147.0651333	116.3787813	83.46219444	120.3935724	101.3347627	94.37235556	151.9736667	141.5345226	144.3460167
1A5 x 1E4	C38.2	118.9603417	80.41088214	92.51217917	76.26142218	67.45820698	57.08816964	133.4716438	103.8560554	89.45447619
1A5 x 1E4	C4.1	125.6319663	77.58713649	95.82068889	105.4003852	69.62644459	59.70382333	110.3769072	79.09003818	73.12279333
1A5 x 1E4	C4.2	121.7002894	77.88447504	77.51602175	99.36923833	77.58271452	66.40835857	146.0494667	100.08159	98.63253999
1A5 x 1E4	C41.1	147.7500086	109.7750465	89.49132975	102.2181195	93.64220574	76.24140111	157.3657333	108.3418756	94.90216883
1A5 x 1E4	C41.2	139.0121882	133.2154852	115.915477	120.1366951	91.64463783	74.86228604	143.0756151	145.9642432	128.0395209
1A5 x 1E4	C42.1	144.6483969	98.84638462	69.41314795	106.0900022	93.79166952	92.66941667	139.81129	130.8678591	131.2997667

1A5 x 1E4	C42.2	142.6855625	131.8754042	122.3299678	106.7901857	77.17219881	54.20860571	141.3477667	124.2586	102.3181
1A5 x 1E4	C44.1	141.4718384	106.8179153	103.8437758	86.13929075	75.16571905	53.4784	107.8652784	83.54049013	87.41918385
1A5 x 1E4	C44.2	138.0350445	117.0016017	103.2994714	107.3702259	93.90524666	82.03859048	154.0073542	147.6823111	148.3282667
1A5 x 1E4	C45.1	137.5851776	94.36936982	90.00061948	88.56857445	87.04234333	65.62448286	125.5999722	104.5545349	83.58703481
1A5 x 1E4	C45.2	136.0938333	92.10665909	71.24163691	139.43201	128.9586983	120.74786	156.2096506	158.758505	145.57049
1A5 x 1E4	C48.1	132.4121205	127.8426862	116.1254657	85.69619206	80.82021031	56.40029	100.51829	126.6741792	75.9943
1A5 x 1E4	C48.2	137.5296833	99.63488952	91.5923883	99.78220204	78.31131814	60.73988556	141.469541	144.0531725	121.1839548
1A5 x 1E4	C49.1	147.9555156	130.9669089	114.1252422	NA	NA	NA	155.6216762	146.5110324	143.8070431
1A5 x 1E4	C5.1	134.74533	91.19825855	67.53456722	112.1167485	67.3717248	54.50375333	144.5631696	109.6402337	97.07734103
1A5 x 1E4	C5.2	122.4868448	98.56379539	94.16805925	108.9229595	64.77106176	50.61289167	128.56437	115.9043164	81.41373214
1A5 x 1E4	C51.1	126.8299393	80.95133587	62.85174085	94.018625	69.96709889	48.91384866	134.4387948	92.10839897	82.27423162
1A5 x 1E4	C51.2	NA	129.402	90.195	125.5095	103.489	91.9435	136.676	120.114	108.526
1A5 x 1E4	C52.1	149.9309667	139.4417	121.3661	102.1914667	91.1401	81.41772083	144.6253333	151.4207333	147.6338333
1A5 x 1E4	C53.1	144.8260667	105.0682333	73.24316667	132.9782122	123.8044111	108.7698917	149.735875	128.65875	114.4807833
1A5 x 1E4	C54.1	135.5046667	90.06941667	78.36625	99.638875	62.7065	45.8876	110.648125	92.74	81.227
1A5 x 1E4	C55.1	109.167344	107.2654064	100.8906894	57.26538036	61.84735752	51.71754559	106.18113	73.07108507	64.8809356
1A5 x 1E4	C55.2	135.1879165	115.0362036	107.6181829	87.86478712	80.1432212	62.87777342	125.08156	91.30203841	74.81903333
1A5 x 1E4	C56.1	145.842725	86.43894667	65.59886	115.9547068	109.7606541	98.63940602	145.4812875	114.9390147	108.0668241
1A5 x 1E4	C56.2	122.2490264	121.5312937	103.0517146	77.84124999	70.86354445	58.7452503	99.59983334	90.83019429	62.575715
1A5 x 1E4	C57.1	144.5168427	91.01843401	69.39815666	90.03664024	81.25058572	62.37663338	133.6969909	114.0097167	100.2970748
1A5 x 1E4	C57.2	139.5225179	129.0397701	118.6768624	136.1172544	82.07330555	79.93935533	147.9636867	144.962802	110.863324
1A5 x 1E4	C59.2	138.3616443	123.1176089	108.8007682	112.8022248	95.06695825	84.43780667	152.3203889	157.2451458	150.3229919
1A5 x 1E4	C6.1	140.369809	76.11906	61.99127151	85.73941124	62.67687485	44.5367	114.6468019	83.5593218	72.51994193
1A5 x 1E4	C60.1	143.2763818	133.1723465	113.3340635	116.2300829	91.46114155	71.00761305	139.8293823	130.9212085	120.1724615
1A5 x 1E4	C60.2	133.4199495	78.84219596	66.76915498	98.95501287	62.58574033	56.96450476	110.4542643	97.89470158	70.73642667

1A5 x 1E4	C61.1	140.4823907	129.3644404	123.0604814	106.8338879	116.219205	72.27605	123.9249949	114.8183606	105.1328956
1A5 x 1E4	C61.2	151.352	129.3785	119.10325	NA	NA	NA	NA	NA	NA
1A5 x 1E4	C62.1	136.1648143	78.11690191	57.21065016	112.6051667	93.17224262	85.99448833	134.4011389	92.88533651	87.48232
1A5 x 1E4	C62.2	136.1580048	125.5261103	113.8715245	100.5129568	58.69548398	46.13089015	119.7205857	105.9849997	84.67107476
1A5 x 1E4	C63.1	142.5026762	130.5333909	121.1775795	91.47786315	67.20438607	47.365845	127.3047442	122.857718	122.6557621
1A5 x 1E4	C7.1	141.5204583	131.4315708	121.2324941	107.0507563	85.77393214	64.21690625	133.6570833	127.1418	75.51886667
1A5 x 1E4	C7.2	136.0962522	92.10649795	69.3716777	120.2493719	116.8331467	103.4156257	156.0892984	141.4524407	146.0501393
1A5 x 1E4	C8.1	129.047	146.4905939	140.9315711	137.959353	86.46366084	59.71768571	119.5967432	107.5144365	74.37524199
1A5 x 1E4	CR4_A1.1	139.9556049	138.0073314	122.8030192	120.6225381	47.92726329	47.05137667	115.2707845	91.49091524	76.67543944
1A5 x 1E4	CR4_A2.1	144.2044739	128.8940845	115.4417262	122.1748049	124.0750834	95.23080757	119.2809543	78.84947969	85.91690825
1A5 x 1E4	CR4_A2.2	132.4851678	94.4412177	71.75834965	114.9885022	99.15919555	82.27587525	140.0621167	136.48	127.7393418
1A5 x 1E4	CR4_A3.1	133.4120786	92.26691548	83.46434429	92.48945	71.28244444	63.00256667	117.039125	98.18359823	76.27530556
1A5 x 1E4	CR4_A3.2	132.4076667	115.7426567	93.26704953	98.72075	67.68665	60.30465	146.1632333	155.88444	145.7870667
1A5 x 1E4	CR4_A4.1	139.0089542	130.8030266	121.9852163	95.89821771	69.33165951	54.53366667	113.5860222	101.7604929	75.25519253
1A5 x 1E4	D1.1	128.2198073	99.31057183	94.87723454	68.54464988	62.15237103	52.74219065	110.2411657	64.06267241	65.10296911
1A5 x 1E4	D1.2	130.8457906	90.43009944	72.0139829	128.3467526	115.8741778	103.55543	143.0750406	151.1918897	130.1428483
1A5 x 1E4	D1.3	136.9333446	127.3751092	115.4084881	68.09081038	64.30700227	57.02493827	127.8084872	103.2388425	85.59412857
1A5 x 1E4	D10.1	137.13191	97.16337923	98.19009126	93.13030816	55.46310278	47.96614167	125.5779811	81.95287525	81.72203053
1A5 x 1E4	D11.1	137.4227722	128.4090567	116.0124194	104.4621952	85.031795	64.62406	152.0947143	148.1162556	140.3647691
1A5 x 1E4	D2.1	147.0093667	111.6559	91.41493636	105.253095	97.73143112	73.492095	109.4359262	110.30165	93.83195279
1A5 x 1E4	D3.1	140.3704502	120.6201148	105.9397132	102.1309036	66.12438462	55.45510715	137.2680826	108.4175022	94.5778123
1A5 x 1E4	D3.2	127.2232359	123.9091457	109.0111575	106.4264574	87.55285172	76.70290848	144.8806259	126.8359511	103.25651
1A5 x 1E4	D4.1	142.9247362	99.85726419	98.16665112	86.86838947	77.3569095	51.355195	126.8347138	112.9523967	86.03669192
1A5 x 1E4	D5.1	139.6082102	123.9581736	101.9029697	129.9543736	120.8089585	91.11336095	155.3814996	158.4681185	155.3631985
1A5 x 1E4	D7.1	141.4650625	90.737	68.1256	99.07035	72.71023833	55.702675	146.61775	145.754375	131.328

1A5 x 1E4	D7.2	142.8895	139.5806627	130.2345897	132.3254059	77.77676049	49.79751746	117.9996648	110.61985	64.02539464
1A5 x 1E4	D7.3	131.353625	117.7358583	118.582375	80.81006667	79.23994167	58.67323333	131.358	145.0743333	134.449
1A5 x 1E4	D9.1	141.3127633	102.2868183	75.38129524	155.4523	146.25109	131.66258	162.43075	164.8569633	155.3209733
1A5 x 1E4	D9.2	138.49125	99.77906587	100.5998273	95.95926784	86.737175	71.68969167	139.1101325	141.2166408	112.2440933

Table S5 Orthologs in *Z. tritici* to genes involved in melanin biosynthesis

Protein ID	Name [a]	Chromo-some	Start (bp)	Stop (bp)	Melanin pathway function	Melanin pathway	Mutant effect	Tested in *Z. tritici*	Highest RPKM mean [b]	RPKM Stdv	Reference [c]
96592	*PKS1*[1)]	11	586456	593034	Polyketide synthase encoding gene	DHN	Full loss of function	No	7.8 (8wpi) ✖	0.6	(Takano *et al.* 1995; Butler and Day 1998; Langfelder *et al.* 2003[2)]; Hu *et al.* 2012[1)])
68710	*THR2*[2)]	2	2514972	2515981	1;3;6;8-tetrahydroxynahthalene reductase encoding gene	DHN	Full loss of function	No	132.5 (13dpi)	41.8	(Butler and Day 1998; Langfelder *et al.* 2003[2)]; Ipcho *et al.* 2012)
18775	*MgSCY1*[1)]	1	963695	964159	Syctalone dehydratase encoding gene	DHN	Full loss of function	No	133.1 (8wpi)	15	(Butler and Day 1998; Langfelder *et al.* 2003[2)]; Ipcho *et al.* 2012)
90337	*SCY2*[3)]	2	133146	133741	Syctalone dehydratase encoding gene	DHN	Full loss of function	No	13.6 (7dpi) ✖	9.4	(Butler and Day 1998; Langfelder *et al.* 2003[2)]; Ipcho *et al.* 2012)
87994	*THR1*[4)]	11	604612	605471	1;3;8-tetrahydroxynahthalene reductase encoding gene	DHN	Full loss of function	No	231.2 (8wpi) ✖	43.3	(Butler and Day 1998; Langfelder *et al.* 2003[2)]; Ipcho *et al.* 2012)
35451	*LAC1*[5)]	2	140750	142914	Laccase encoding gene	DHN/ DOPA ?	Reduced	No	24.3 (7dpi) ✖	12.4	(Lin *et al.* 2012; Upadhyay *et al.* 2013)
36350	*LAC2*[5)]	2	144093	146209	Laccase encoding gene	DHN/ DOPA ?	Reduced	No	40.8 (7dpi) ✖	24.2	(Lin *et al.* 2012; Upadhyay *et al.* 2013)
42222	*LAC3*[5)]	5	2279071	2281252	Laccase encoding gene	DHN/ DOPA ?	Reduced	No	38.0 (7dpi) ✖	22	(Lin *et al.* 2012; Upadhyay *et al.* 2013)

42793	*LAC4*[5)]	5	2282533	2284432	Laccase encoding gene	DHN/ DOPA ?	Reduced	No	32.6 (7dpi)	20.8	(Lin *et al.* 2012; Upadhyay *et al.* 2013)
32322	*LAC5*[5)]	1	5292579	5294444	Laccase encoding gene	DHN/ DOPA ?	Reduced	No	56.7 (7dpi) ✖	37.8	(Lin *et al.* 2012; Upadhyay *et al.* 2013)
108040	*LAC6*[5)]	2	1650426	1652574	Laccase encoding gene	DHN/ DOPA ?	Reduced	No	193.8 (7dpi) ✖	49.5	(Lin *et al.* 2012; Upadhyay *et al.* 2013)
38161	*LAC7*[5)]	3	2746686	2748706	Laccase encoding gene	DHN/ DOPA ?	Reduced	No	7.9 (8wpi)	0.6	(Lin *et al.* 2012; Upadhyay *et al.* 2013)
109666	*LAC8*[5)]	5	2492745	2495149	Laccase encoding gene	DHN/ DOPA ?	Reduced	No	451.7 (8wpi) ✖	74.3	(Lin *et al.* 2012; Upadhyay *et al.* 2013)
52873	*MgGpa1*[1)]	1	2514902	2516229	G alpha protein encoding gene	?	Hampered	Yes	132.5 (13dpi)	17.6	(Solomon *et al.* 2004; Mehrabi *et al.* 2009)
68892	*MgGpb1*[1)]	2	2862220	2863783	G beta protein encoding gene	?	Hampered	Yes	118.2 (13dpi) ✖	7.3	(Mehrabi *et al.* 2009)
76503	*MgGpa3*[1)]	10	1111808	1113544	G alpha protein encoding gene	?	Reduced	Yes	33.9 (13dpi)	4.9	(Mehrabi *et al.* 2009)
76502	*MgHog1*[1)]	10	1108132	1110166	Mitogen-activated protein kinase encoding gene	?	Full loss of function	Yes	228.6 (7dpi)	64.8	(Mehrabi *et al.* 2006b)
102121	*MgSlt2*[1)]	1	1331331	1332992	Mitogen-activated protein kinase encoding gene	?	Full loss of function	Yes	205.4 (13dpi) ✖	14.8	(Mehrabi *et al.* 2006a)
73071	*MgFus3*[1)]	6	1182095	1184167	Mitogen-activated protein kinase encoding gene	?	Full loss of function	Yes	163.1 (13dpi) ✖	3.9	(Cousin *et al.* 2006)
37276	*MVE1*[1)]	2	2995014	2995823	Velvet gene	?	Full loss of function	Yes	123.8 (7dpi)	38.1	(Choi and Goodwin 2011b)
47503	*MCC1*[1)]	9	1594202	1595191	C-type cyclin	?	Increased	Yes	35.5 (13dpi)	7	(Choi and Goodwin 2011a)

					encoding gene					
96591	*PRF1*[5)]	11	584834	585795	Prefoldin chaperone encoding gene	DHN ?	?	No	91.4 (8wpi)	11	No Reference, but this is a gene just downstream from 96592 (*PKS1*), thus in the region of the expected DHN melanin pathway cluster. It shows conservation over other fungi and thus is believed to belong to the DHN melanin pathway.
87993	*CMR1*[4)]	11	600309	603354	Transcription factor encoding gene	DHN ?	Full loss of function	No	12.7 (8wpi) ✖	1.6	(Eliahu *et al.* 2007)
99683	*AYG1*[6)]	3	2737599	2739046	Unknown	DHN	Reduced	No	34.0 (8wpi) ✖	3.4	(Tsai *et al.* 1999[3)])
100584	*AYG2*[7)]	6	2057066	2058412	Unknown	DHN	Reduced	No	15.7 (13dpi) ✖	0.4	(Tsai *et al.* 1999[3)])
72443	*PEX13*[8)]	5	1942248	1943680	Peroxisome biogenesis factor encoding gene	?	Full loss of function	No	101.9 (7dpi) ✖	40.6	(Fujihara *et al.* 2010)
98537	*PEX6*[4)]	1	3057033	3061525	Peroxisome assembly factor encoding gene	?	Reduced	No	21.1 (13dpi)	2.2	(Kimura *et al.* 2001)
65403	*CLAP1*[9)]	1	1543207	1547078	Copper-transporting ATPase encoding gene	?	Reduced	No	41.4 (7dpi)	12.5	(Parisot *et al.* 2002)
57911	*MAC1*[10)]	4	1213598	1215218	Transcription factor encoding gene	?	Reduced	No	201.6 (7dpi) ✖	48.5	(Lin *et al.* 2006)

[a] [1)] Name as found in the reference literature or on the joint genome institute (JGI) website for *Z. tritici* (http://genomeportal.jgi-psf.org/Mycgr3/Mycgr3.home.html). [2)] Name given by us. Similar name given as found in ortholog of *Colletotrichum lagenarium*. [3)] Two syctalone dehydratases (Protein ID: 18775 / 90337) were found when blasting the *SNOG_11564.3* (Ipcho *et al.* 2012) gene against *Z. tritici*. As 18775 had the name '*MgSCY1*' (JGI), we gave 90337 the name '*SCY2*'. [4)] Same name given as found in ortholog of *Colletotrichum lagenarium*. [5)] Name given by us by abbreviating the enzyme encoded by the gene. [6)] Same name

given as found in ortholog of *Aspergillus fumigatus*. [7)] Name given by us. Similar name given as found in ortholog of *Aspergillus fumigatus*. [8)] Same name given as found in ortholog of *Colletotrichum orbiculare*. [9)] Same name given as found in ortholog of *Colletotrichum lindemuthianum*. [10)] Same name given as found in ortholog of *Cryptococcus neoformans*.

[b] We had chosen a RKPM value of 2 as an expression threshold. All the genes associated with melanization showed ≥ 2 RPKM values. Hence all of the orthologs could affect melanization in *Z. tritici.* ✖ Indicates significant changes in transcript abundances over time.

[c] [1)] Hu, Y., X. Hao, J. Lou, P. Zhang, J. Pan et al., 2012 A PKS gene, pks-1, is involved in chaetoglobosin biosynthesis, pigmentation and sporulation in *Chaetomium globosum*. Science China-Life Sciences 55: 1100–1108.

[2)] Langfelder, K., M. Streibel, B. Jahn, G. Haase, and A. A. Brakhage, 2003 Biosynthesis of fungal melanins and their importance for human 11 pathogenic fungi. Fungal Genet. Biol. 38: 143–158.

[3)] Tsai, H. F., M. H. Wheeler, Y. C. Chang, and K. J. Kwon-Chung, 1999 A developmentally regulated gene cluster involved in conidial pigment biosynthesis in *Aspergillus fumigatus*. J. Bacteriol. 181: 6469–6477.

Table S6   Summary of QTL positions and effects for cross 3D1 x 3D7.

Environment	Colony age (dpi)	QTL peak marker [a]	Chromosome	Estimated position of peaking marker (cM)	Estimated position of peaking marker (kb)	LOD score at peak	P-value	Mean 1A5 allele (grey values)	Mean 1E4 allele (grey values)	Mean difference	Allele effect [b]	Percentage of variance explained by QTL (%)	Estimated position of proximal marker (kb) [c]	Estimated position of distal marker (kb) [c]	Bayes confidence interval length (kb)
Cold	8	c10.loc107 (p)	10	107.13	646	8.29	< 0.001	135.78	129.09	6.69	3D1	13.6	634	673	39
Cold	8	12_143193	12	5.74	143	4.85	0.002	134.56	129.28	5.27	3D1	9.2	29	305	276
Cold	11	8_1250811	8	140.32	1251	4.07	0.01	101.72	112.39	10.66	3D7	7.1	897	2228	1332
Cold	11	c10.loc108 (p)	10	108.13	649	9.55	< 0.001	116.28	100.17	16.11	3D1	15.6	634	673	39
Cold	11	11_535446	11	77.83	535	4.32	0.006	113.31	102.18	11.13	3D1	7.4	447	1332	885
Control	8	c11.loc82 (p)	11	82.06	581	32.2	< 0.001	118	93.73	24.27	3D1	39.2	560	603	43
Control	11	5_871061	5	157.26	871	4.38	0.003	74.99	85.47	10.48	3D7	7.5	449	2799	2350
Control	11	c11.loc81 (p)	11	81.06	571	30.79	< 0.001	93.62	69.23	24.4	3D1	38.2	549	603	54
Control	14	5_958098	5	160.33	958	3.78	0.016	61.52	69.96	8.43	3D7	6.5	449	2799	2350
Control	14	c11.loc80 (p)	11	80.06	560	21.17	< 0.001	75.79	57.28	18.51	3D1	30	512	603	91
Fungicide	8	c11.loc81 (p)	11	81.06	571	34	< 0.001	124.55	100.17	24.38	3D1	45.8	549	592	43
Fungicide	11	c11.loc82 (p)	11	82.06	581	23.8	< 0.001	96.79	75.05	21.74	3D1	30.4	549	625	76
Fungicide	14	1_1740191	1	193.5	1740	3.64	0.014	64.87	73.03	8.16	3D7	6.3	1063	4267	3203
Fungicide	14	c11.loc81 (p)	11	81.06	571	19.34	< 0.001	78.24	60.96	17.28	3D1	27.2	535	614	79

[a] A (p) indicates that a pseudomarker provided the highest LOD score. In all other cases a true marker provided the highest LOD score.

[b] 3D7 indicates that the parental 3D7 allele provided the higher phenotypic mean then the parental 3D1 allele, while 3D1 indicates that the parental 3D1 allele provided the higher phenotypic mean then the parental 3D7 allele.

[c] Markers flanking Bayes confidence interval.

Table S7 Summary of QTL positions and effects for cross 1A5 x 1E4.

Environment	Colony age (dpi)	QTL peak marker [a]	Chromosome	Estimated position of peaking marker (cM)	Estimated position of peaking marker (kb)	LOD score at peak	P-value	Mean 1A5 allele (grey values)	Mean 1E4 allele (grey values)	Mean difference	Allele effect [b]	Percentage of variance explained by QTL (%)	Estimated position of proximal marker (kb) [c]	Estimated position of distal marker (kb) [c]	Bayes confidence interval length (kb)
Cold	8	1_2840762	1	445.26	2841	3.6	0.029	134.52	138.11	3.58	1E4	6.3	2604	3275	671
Cold	8	c2.loc226 (p)	2	226.07	1699	10	< 0.001	139.11	133.43	5.69	1A5	17	1646	1819	173
Cold	8	c3.loc208 (p)	3	208.04	1301	8.52	< 0.001	133.71	138.79	5.08	1E4	12.1	869	1749	880
Cold	11	1_1553785	1	219.31	1554	8.59	< 0.001	103.25	117.72	14.47	1E4	14.3	1020	1627	607
Cold	11	2_1814469	2	243.95	1814	3.68	0.018	114.88	105.17	9.71	1A5	6.4	1428	2270	842
Cold	11	5_1699464	5	224.63	1699	5.9	< 0.001	116.26	104.11	12.15	1A5	10.1	773	1911	1138
Cold	11	8_429575	8	58.42	430	7.65	< 0.001	117.28	103.6	13.68	1A5	12.8	348	446	99
Cold	14	1_1028414	1	157.63	1028	7.65	< 0.001	91.91	105.6	13.69	1E4	13	1014	1589	575
Cold	14	c3.loc249 (p)	3	249.04	1780	3.44	0.046	103.29	94.17	9.12	1A5	5.7	1621	1968	347
Cold	14	5_1593047	5	215.71	1593	5.13	< 0.001	104.07	92.71	11.36	1A5	8.9	1230	1913	683
Cold	14	7_841106	7	181.47	841	4.01	0.014	103.42	93.3	10.13	1A5	7	428	1893	1464
Cold	14	8_397588	8	55.79	398	6.62	< 0.001	105.18	92.37	12.81	1A5	11.3	348	446	99
Control	8	c1.loc106 (p)	1	106.14	771	3.4	0.037	106.6	97.98	8.62	1A5	5.8	441	5371	4930
Control	8	2_1526778	2	205.66	1527	7.34	< 0.001	108.31	95.86	12.45	1A5	12.3	1455	1646	191
Control	11	1_3069693	1	492.19	3070	6.24	< 0.001	76.24	88.1	11.86	1E4	10.9	2919	3249	331
Control	14	1_3006378	1	480.86	3006	5.32	< 0.001	61.69	72.61	10.92	1E4	9.2	2854	5652	2798
Control	14	3_1739730	3	245.64	1740	4.29	0.005	62.41	72.25	9.84	1E4	7.5	1494	1968	474
Control	14	4_421356	4	55.21	421	5.61	< 0.001	61.71	72.91	11.2	1E4	9.5	161	476	315
Control	14	5_1919504	5	249.23	1920	4.64	0.001	62.31	72.55	10.24	1E4	8	1385	2105	721

Control	14	11_523597	11	66.72	524	4.71	< 0.001	72.97	62.64	10.33	1A5	8.2	329	581	252
Fungicide	8	2_1470426	2	201.05	1470	7.55	< 0.001	140.56	127.73	12.83	1A5	12.9	1464	1814	350
Fungicide	8	c4.loc245 (p)	4	245.04	1471	7.17	< 0.001	127.64	140.17	12.53	1E4	12.3	415	1802	1386
Fungicide	11	1_3249298	1	511.19	3249	6.55	< 0.001	108.84	124.9	16.05	1E4	11.2	2974	3275	301
Fungicide	11	2_1632971	2	211.37	1633	4.01	0.011	123.66	111.06	12.6	1A5	7	1428	2375	947
Fungicide	11	3_1371178	3	221.45	1371	4.34	0.007	111.23	124.29	13.06	1E4	7.6	1308	2180	872
Fungicide	11	c4.loc52 (p)	4	52.04	418	9.15	< 0.001	108.37	126.55	18.18	1E4	14.8	415	1094	679
Fungicide	14	1_3249289	1	511.19	3249	6.24	< 0.001	93.34	109.6	16.26	1E4	10.7	2840	5735	2894
Fungicide	14	2_1632971	2	211.37	1633	4.14	0.01	108.58	95.31	13.27	1A5	7.2	1428	2084	657
Fungicide	14	3_1739730	3	245.64	1740	4.22	0.01	95.5	108.91	13.41	1E4	7.4	1315	2206	891
Fungicide	14	c4.loc54 (p)	4	54.04	420	9.47	< 0.001	92.37	111.73	19.36	1E4	16.2	417	426	9
Fungicide	14	6_2299166	6	289.62	2299	3.56	0.027	108.33	96	12.33	1A5	6.2	1166	2438	1272
Fungicide	14	c11.loc70 (p)	11	70.07	533	4.48	0.007	109.68	96.1	13.58	1A5	7.8	271	1160	889

[a] A (p) indicates that a pseudomarker provided the highest LOD score. In all other cases a true marker provided the highest LOD score.
[b] 1E4 indicates that the parental 1E4 allele provided the higher phenotypic mean then the parental 1A5 allele, while 1A5 indicates that the parental 1A5 allele provided the higher phenotypic mean then the parental 1E4 allele.
[c] Markers flanking Bayes confidence interval.

Table S8 Summary of genes affected by sequence variation within each Bayes confidence interval for cross 3D1 x 3D7, excluding all genes containing no sequence variation or with synonymous SNPs only.

Environment	Colony age (dpi)	Chromosome	Estimated position of peaking marker (kb)	LOD score at peak	P-value	Estimated position of proximal marker (kb) [a]	Estimated position of distal marker flanking (kb) [a]	Bayes confidence interval length (kb)	Number of sequence variations [b]	Number of genes [b]	Number of genes affected by sequence variations [b]	Percentage of total genes affected by sequence variations (%) [b]	Number of sequence variation affected genes with unknown function [b]	Percentage of total sequence variation affected genes with unknown function (%) [b]	Number of sequence variation affected genes with significant changes in transcript abundances [b]	Percentage of total sequence variation affected genes with significant changes in transcript abundances (%) [b]
Fungicide	14	1	1740	3.64	0.014	1063	4267	3203	4342	1130	858	76	296	34	349	41
Control	11	5	871	4.38	0.003	449	2799	2350	2551	679	504	74	170	34	220	44
Control	14	5	958	3.78	0.016	449	2799	2350	2550	679	504	74	170	34	220	44
Cold	11	8	1251	4.07	0.01	897	2228	1332	1661	433	304	70	112	37	114	38
Cold	8	10	646	8.29	< 0.001	634	673	39	59	15	12	80	2	17	7	58
Cold	11	10	649	9.55	< 0.001	634	673	39	59	15	12	80	2	17	7	58
Control	8	11	581	32.2	< 0.001	560	603	43	8	14	4	29	0	0	1	25
Control	11	11	571	30.79	< 0.001	549	603	54	13	16	6	38	1	17	3	50
Fungicide	8	11	571	34	< 0.001	549	592	43	12	14	6	43	1	17	3	50
Fungicide	11	11	581	23.8	< 0.001	549	625	76	28	24	12	50	4	33	5	42
Fungicide	14	11	571	19.34	< 0.001	535	614	79	50	26	13	50	5	38	6	46
Control	14	11	560	21.17	< 0.001	512	603	91	67	29	16	55	5	31	6	38
Cold	11	11	535	4.32	0.006	447	1332	885	1025	277	190	69	78	41	86	45
Cold	8	12	143	4.85	0.002	29	305	276	441	78	56	72	22	39	23	41

[a] Markers flanking Bayes confidence interval.
[b] Numbers refer to within Bayes confidence interval.

Table S9 Summary of genes affected by sequence variation within each Bayes confidence interval for cross 1A5 x 1E4 excluding all genes containing no sequence variation or with synonymous SNPs only.

Environment	Colony age (dpi)	Chromosome	Estimated position of peaking marker (kb)	LOD score at peak	P-value	Estimated position of proximal marker (kb) [a]	Estimated position of distal marker flanking (kb) [a]	Bayes confidence interval length (kb)	Number of sequence variations [b]	Number of genes [b]	Number of genes affected by sequence variations [b]	Percentage of total genes affected by sequence variations (%) [b]	Number of sequence variation affected genes with unknown function [b]	Percentage of total sequence variation affected genes with unknown function (%) [b]	Number of sequence variation affected genes with significant changes in transcript abundances [b]	Percentage of total sequence variation affected genes with significant changes in transcript abundances (%) [b]
Fungicide	11	1	3249	6.55	< 0.001	2974	3275	301	677	113	84	74	25	30	30	36
Control	11	1	3070	6.24	< 0.001	2919	3249	331	725	121	90	74	27	30	33	37
Cold	14	1	1028	7.65	< 0.001	1014	1589	575	784	182	145	80	49	34	72	50
Cold	11	1	1554	8.59	< 0.001	1020	1627	607	853	195	154	79	54	35	74	48
Cold	8	1	2841	3.6	0.029	2604	3275	671	1257	243	184	76	60	33	72	39
Control	14	1	3006	5.32	< 0.001	2854	5652	2798	3855	963	704	73	237	34	278	39
Fungicide	14	1	3249	6.24	< 0.001	2840	5735	2894	3956	997	732	73	244	33	294	40
Control	8	1	771	3.4	0.037	441	5371	4930	6888	1673	1245	74	436	35	511	41
Cold	8	2	1699	10	< 0.001	1646	1819	173	225	48	38	79	18	47	28	74
Control	8	2	1527	7.34	< 0.001	1455	1646	191	240	59	44	75	15	34	15	34
Fungicide	8	2	1470	7.55	< 0.001	1464	1814	350	437	101	78	77	31	40	40	51
Fungicide	14	2	1633	4.14	0.01	1428	2084	657	803	188	139	74	59	42	60	43

Cold	11	2	1814	3.68	0.018	1428	2270	842	1075	262	195	74	88	45	85	44
Fungicide	11	2	1633	4.01	0.011	1428	2375	947	1193	301	222	74	95	43	95	43
Cold	14	3	1780	3.44	0.046	1621	1968	347	366	116	81	70	20	25	25	31
Control	14	3	1740	4.29	0.005	1494	1968	474	465	150	103	69	28	27	34	33
Cold	8	3	1301	8.52	< 0.001	869	1749	880	933	266	186	70	69	37	68	37
Fungicide	11	3	1371	4.34	0.007	1308	2180	872	949	282	197	70	62	31	71	36
Fungicide	14	3	1740	4.22	0.01	1315	2206	891	967	290	202	70	64	32	76	38
Fungicide	14	4	420	9.47	< 0.001	417	426	9	7	2	1	50	0	0	0	0
Control	14	4	421	5.61	< 0.001	161	476	315	274	80	59	74	19	32	21	36
Fungicide	11	4	418	9.15	< 0.001	415	1094	679	905	213	153	72	73	48	67	44
Fungicide	8	4	1471	7.17	< 0.001	415	1802	1386	1676	417	297	71	131	44	125	42
Control	14	5	1920	4.64	0.001	1385	2105	721	744	215	156	73	56	36	68	44
Cold	14	5	1593	5.13	< 0.001	1230	1913	683	764	219	162	74	54	33	65	40
Cold	11	5	1699	5.9	< 0.001	773	1911	1138	1272	352	259	74	86	33	102	39
Fungicide	14	6	2299	3.56	0.027	1166	2438	1272	1099	342	244	71	82	34	93	38
Cold	14	7	841	4.01	0.014	428	1893	1464	1194	440	224	51	90	40	103	46
Cold	11	8	430	7.65	< 0.001	348	446	99	132	27	22	81	9	41	7	32
Cold	14	8	398	6.62	< 0.001	348	446	99	132	27	22	81	9	41	7	32
Control	14	11	524	4.71	< 0.001	329	581	252	324	84	67	80	24	36	37	55
Fungicide	14	11	533	4.48	0.007	271	1160	889	1064	275	203	74	76	37	100	49

[a] Markers flanking Bayes confidence interval.
[b] Numbers refer to within Bayes confidence interval.

Table S10 Genes within large-effect QTL regions (confidence intervals containing ≤ 30 candidate genes) for cross 3D1 x 3D7, excluding genes with no sequence variation or with only synonymous SNPs.

Protein ID [a]	Gene ontology Name	Gene ontology biological process	Gene ontology cellular component	Gene ontology molecular function	Number of Non-Syn SNPs [b]	Number of other sequence variations [b c]	Additional information	Highest RPKM mean [d]	RPKM Stdv
Chromosome 10 (Phenotype: 11 dpi cold)									
101405	catalytic activity	acetyl-CoA metabolic process; acetate metabolic process; pyruvate metabolic process;	cytosol; mitochondrion; acetate CoA-transferase complex;	acetate CoA-transferase activity; acetyl-CoA hydrolase activity;	2 (M)	0	/	71.4 (13dpi)	17.4
105917 °	5-methyltetrahydropteroyltriglutamate-homocysteine S-methyltransferase activity	methionine biosynthetic process; methylation;	Not described	5-methyltetrahydropteroyltriglutamate-homocysteine S-methyltransferase activity;	3 (M)	2 upstream (m), 1 UTR5 Prime (m)	/	148.0 (7dpi)	24.0
111014	proteolysis	proteolysis;	Not described	serine-type peptidase activity;	7 (M)	2 intron (m), 1 UTR5 Prime (m)	/	49.8 (7dpi)	43.3
96183	Not described	Not described	Not described	Not described	5 (M)	2 intron (m)	/	0 (No	0.0

								Specific Day)	
76249 °	protein kinase activity	protein phosphorylation; serine family amino acid metabolic process;	Not described	ATP binding; protein serine/threonine kinase activity;	3 (M)	0	Name: MgPbs2 (Hypothetical MAP kinase kinase (MAPKK) involved in the osmosensing signal-transduction pathway, activated under severe osmotic)	139.2 (7dpi)[illegible]	31.8
101408 # °	integral to membrane	transmembrane transport;	integral to membrane;	Not described	2 (M)	0	Transporter - auxin efflux carrier-like with six predicted transmembrane regions	168.0 (8wpi) [illegible]	24.3
48576 °	amino acid transport	amino acid transmembrane transport;	integral to membrane;	amino acid transmembrane transporter activity;	18 (M)	1 intron (m)	/	15.5 (8wpi)[illegible]	1.4
76256 °	N-acetyltransferase activity	acyl-carrier-protein biosynthetic process;	Not described	N-acetyltransferase activity;	2 (M)	1 frame shift (H)	/	24.9 (13dpi)	8.6

48696	DNA binding	regulation of transcription, DNA-dependent;	transcription factor complex;	sequence-specific DNA binding transcription factor activity; DNA binding;	0	1 upstream (m), 1 intron (m)	Name: MgSwi6 (Hypothetical transcription factor that is related to DNA binding component of the SBF complex (Swi4p-Swi6p) in S. cerevisiae)	12.3 (13dpi)[illegible]	3.1
76259	Not described	oxidation-reduction process; tryptophan metabolic process; peroxidase reaction; response to oxidative stress; methane metabolic process;	Not described	heme binding; catalase activity;	1 (M)	0	/	70.7 (8wpi)[illegible]	12.6
101410	protein binding	Not described	Not described	Not described	1 (M)	1 upstream (m), 1 UTR5 Prime (m)	/	413.6 (7dpi)[illegible]	64.8
105922	Not described	Not described	Not described	Not described	0	1 UTR5 Prime (m)	/	48.9 (13dpi)[illegible]	6.7
Chromosome 11 (Phenotype: 8 dpi control)									
96588 °	Not described	Not described	intracellular;	zinc ion binding; nucleic acid binding;	1 (M)	1 intron (m)	/	2.0 (8wpi)	0.6

50123	ubiquitin thiolesterase activity	ubiquitin-dependent protein catabolic process; protein deubiquitination;	Not described	ubiquitin thiolesterase activity; cysteine-type peptidase activity;	0 (M)	2 intron (m)	/	31.9 (8wpi)	3.9
96591 # ° $	protein folding	protein folding;	prefoldin complex;	unfolded protein binding;	0 (M)	1 upstream (m)	/	91.4 (8wpi)	11.0
96592 ° $	catalytic activity	biosynthetic process;	Not described	hydrolase activity, acting on ester bonds; transferase activity;	3 (M)	0	Name: *PKS1* (polyketide synthase gene highly similar to those involved in fungal melanin biosynthesis; contains conserved domains typically found in PKSs that synthesize fungal pigments)	7.8 (8wpi) [illegible]	0.6

[a] A # Indicates the QTL peak is positioned closest to this gene or within the gene. A ° Indicates candidate genes with a higher priority of contributing to melanization compared to unmarked candidate genes. $ Indicates orthologs in *Z. tritici* to genes involved in melanin biosynthesis (Table S5).

[b] The letter within brackets following the number of a specific sequence polymorphism refers to the likely impact of the sequence polymorphism according to SnpEff. H=high, M=moderate, L=low, m=modifier.

[c] Other sequence variations include codon change plus insertion/deletion, codon insertion/deletions, 100 bp upstream or downstream, frame shift, intron, splice site acceptor/donor, start gained/lost, stop gained/lost, untranslated regions (UTR).

[d] We chose a RKPM value of 2 as an expression threshold. [illegible]A [illegible]Indicates significant changes in transcript abundances over time.

Table S11 Genes within large-effect QTL regions (confidence intervals containing ≤ 30 candidate genes) for cross 1A5 x 1E4, excluding genes with no sequence variation or with only synonymous SNPs.

Protein ID [a]	Gene ontology Name	Gene ontology biological process	Gene ontology cellular component	Gene ontology molecular function	Number of Non-Syn SNPs [b]	Number of other sequence variations [b c]	Additional information	Highest RPKM mean [d]	RPKM Stdv
Chromosome 4 (Phenotype: 14 dpi fungicide)									
92291 # °	transcription factor activity	regulation of transcription from RNA polymerase II promoter;	nucleus;	sequence-specific DNA binding RNA polymerase II transcription factor activity; zinc ion binding;	0 (M)	4 downstream (m), 1 upstream (m), 2 intron (m)	/	80.7 (7 dpi)	21.9
Chromosome 8 (Phenotype: 11 dpi cold)									
94941	Not described	Not described	Not described	Not described	0 (M)	4 intron (m)	/	14.7 (13dpi)	4.8
94942	Not described	Not described	Not described	Not described	16 (M)	0	/	0.2 (8wpi)	0.2
14031	Not described	Not described	Not described	Not described	9 (M)	1 upstream (m)	/	3.2 (13dpi)	1.6
74422	Not described	Not described	Not described	Not described	0 (M)	1 intron (m)	/	33.2 (13dpi)	5.9

94946	Not described	Not described	Not described	Not described	7 (M)	0	/	4.3 (13dpi) ▯	2.9
100879	glucose-6-phosphate dehydrogenase activity	pentose-phosphate shunt; response to hydrogen peroxide; glutathione metabolic process;	cytoplasm;	glucose-6-phosphate dehydrogenase activity; NADP binding;	0 (M)	1 downstream (m), 2 UTR5 Prime	Name: G6PD (EC 1.1.1.49 glucose-6-phosphate dehydrogenase; NADP-glucose-6-phosphate dehydrogenase; Zwischenferment; D-glucose 6-phosphate dehydrogenase; glucose 6-phosphate dehydrogenase (NADP); NADP-dependent glucose 6-phosphate dehydrogenase; 6-phosphoglucose dehydrogenase; Entner-Doudoroff enzyme; glucose-6-phosphate 1-dehydrogenase; G6PDH; GPD)	623.7 (8wpi)	33.8

74429	structural constituent of ribosome	translation; ribosome biogenesis;	ribosome;	structural constituent of ribosome;	0 (M)	2 downstream (m), 1 upstream (m), 2 intron (m), 4 UTR3 Prime	/	2315.0 (13dpi)	194.8
87034	Not described	ribosomal large subunit assembly;	nucleolus; preribosome, large subunit precursor;	5S rRNA binding; rRNA primary transcript binding;	3 (M)	0	/	58.2 (13dpi)	6.8
87035	DNA binding	transcription, DNA-dependent; response to DNA damage stimulus;	membrane;	DNA binding; ATP binding; helicase activity;	1 (M)	1 downstream (m)	Homolog of Saccharomyces cerevisiae INO80, a Swi2/Snf2-related ATPase that forms a large complex, containing actin and several actin-related proteins, that has chromatin remodeling activity and 3' to 5' DNA helicase activity in vitro	36.0 (13dpi)	6.0

46572	Not described	Not described	Not described	Not described	0 (M)	1 upstream (m), 1 intron (m)	/	107.2 (7dpi) ▯	18.9
27012	phospholipid biosynthetic process	phospholipid biosynthetic process;	membrane;	phosphotransferase activity, for other substituted phosphate groups;	0 (M)	2 upstream (m)	Putative CDP-alcohol phosphatidyltransferase. Shows some sequence similarity to cardiolipin synthase. Predicted signal peptide. Predicted membrane localization.	10.0 (7dpi)	5.5
110344 °	Not described	nuclear mRNA cis splicing, via spliceosome; mRNA transport;	nuclear cap binding complex;	RNA cap binding;	4 (M)	1 downstream (m), 2 frame shift (H), 1 intron (m)	/	48.6 (13dpi) ▯	5.2
46635	nucleus	nuclear mRNA splicing, via spliceosome; cellular bud site selection;	U2 snRNP;	Not described	0 (M)	2 intron (m)	/	69.1 (7dpi)	25.6

100881	protein kinase activity	protein phosphorylation; serine family amino acid metabolic process;	Not described	ATP binding; protein serine/threonine kinase activity;	0 (M)	3 intron (m), 1 UTR5 Prime (m)	Name: GSK3 (serine/threonine protein kinase, CMGC family, glycogen synthase kinase subfamily)	180.8 (13dpi) ▯	51.8
87038	Not described	Not described	Not described	nucleotide binding;	2 (M)	0	/	1.1 (7dpi)	1.8
29926 °	Not described	Not described	Not described	Not described	0 (M)	1 upstream (m), 6 intron (m)	/	31.7 (13dpi) ▯	3.2
74444 °	tRNA processing	mitochondrial tRNA wobble uridine modification;	mitochondrion;	flavin adenine dinucleotide binding;	10 (M)	2 intron (m)	/	31.5 (7dpi)	9.5
87039 #	Not described	Not described	membrane;	Not described	3 (M)	0	/	0 (No Specific Day)	0
46302 °	protein kinase activity	protein phosphorylation; serine family amino acid metabolic process;	Not described	ATP binding; protein serine/threonine kinase activity;	4 (M)	4 downstream (m), 2 intron (m)	/	21.6 (13dpi)	2.0

110348 °	nucleic acid binding	DNA catabolic process;	Not described	endonuclease activity; nucleic acid binding;	7 (M)	0	/	4.5 (7dpi)	4.5
94962 °	Not described	Not described	Not described	Not described	3 (M)	0	/	13.0 (7dpi) ▯	7.7
110349 °	Not described	Not described	Not described	Not described	9 (M)	3 frame shift (H), 3 intron (m)	putative secreted protein unknown function, probable unique to M graminicola.	11.8 (7dpi)	8.3

[a] A # Indicates the QTL peak is positioned closest to this gene or within the gene. A ° Indicates candidate genes with a higher priority of contributing to melanization compared to unmarked candidate genes. $ Indicates orthologs in *Z. tritici* to genes involved in melanin biosynthesis (Table S5).
[b] The letter within brackets following the number of a specific sequence polymorphism refers to the likely impact of the sequence polymorphism according to SnpEff. H=high, M=moderate, L=low, m=modifier.
[c] Other sequence variations include codon change plus insertion/deletion, codon insertion/deletions, 100 bp upstream or downstream, frame shift, intron, splice site acceptor/donor, start gained/lost, stop gained/lost, untranslated regions (UTR).
[d] We chose a RKPM value of 2 as an expression threshold. ▯A ▯▯Indicates significant changes in transcript abundances over time.

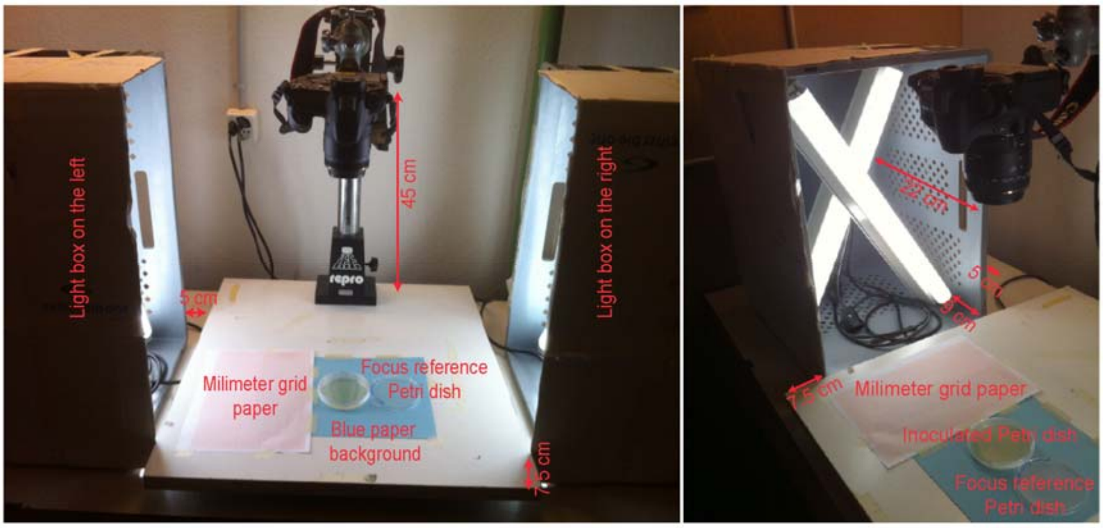

Figure S1 Camera and light setup overview.

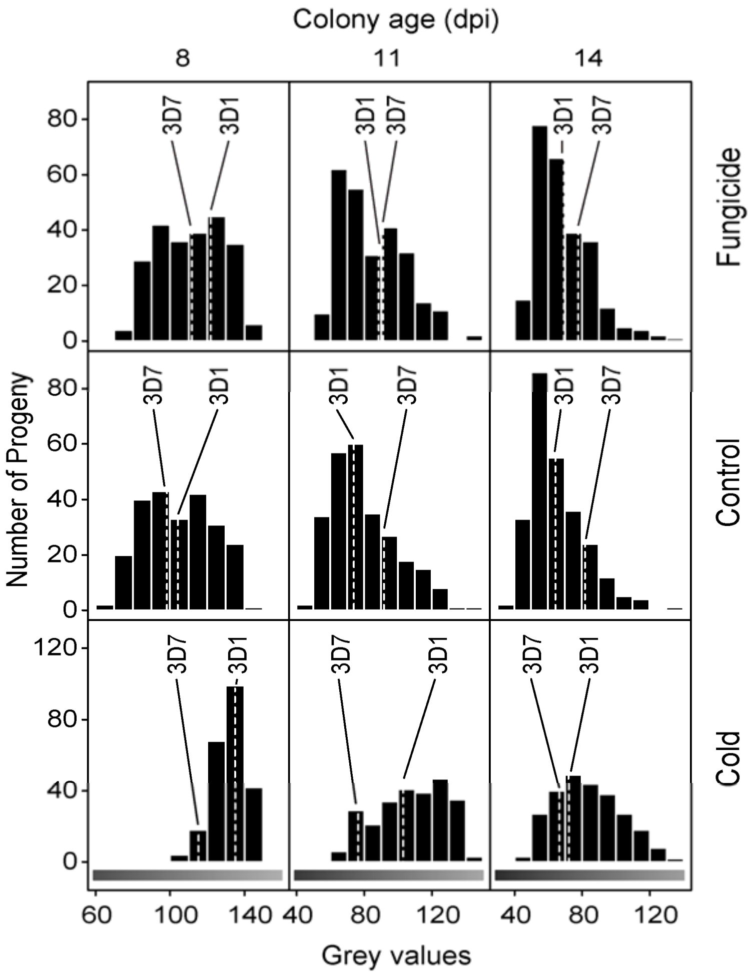

Figure S2 Melanization measured in the cross 3D1 x 3D7 over the three different environments. Each environment is represented by the three colony ages. The rug just above the grey values corresponds to the intensity of grey shading. Melanization increases as time progresses, indicated with lower mean values for older colonies.

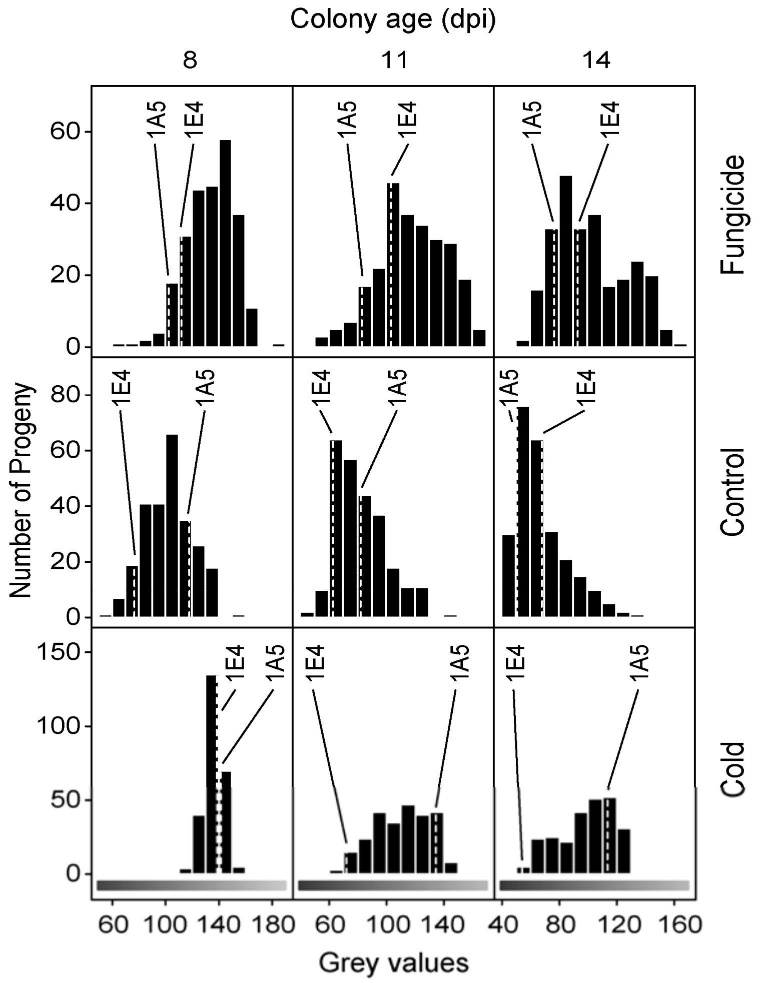

Figure S3 Melanization measured in the cross 1A5 x 1E4 over the three different environments. Each environment is represented by the three colony ages. The rug just above the grey values corresponds to the intensity of grey shading. Melanization increases as time progresses, indicated with lower mean values for older colonies.

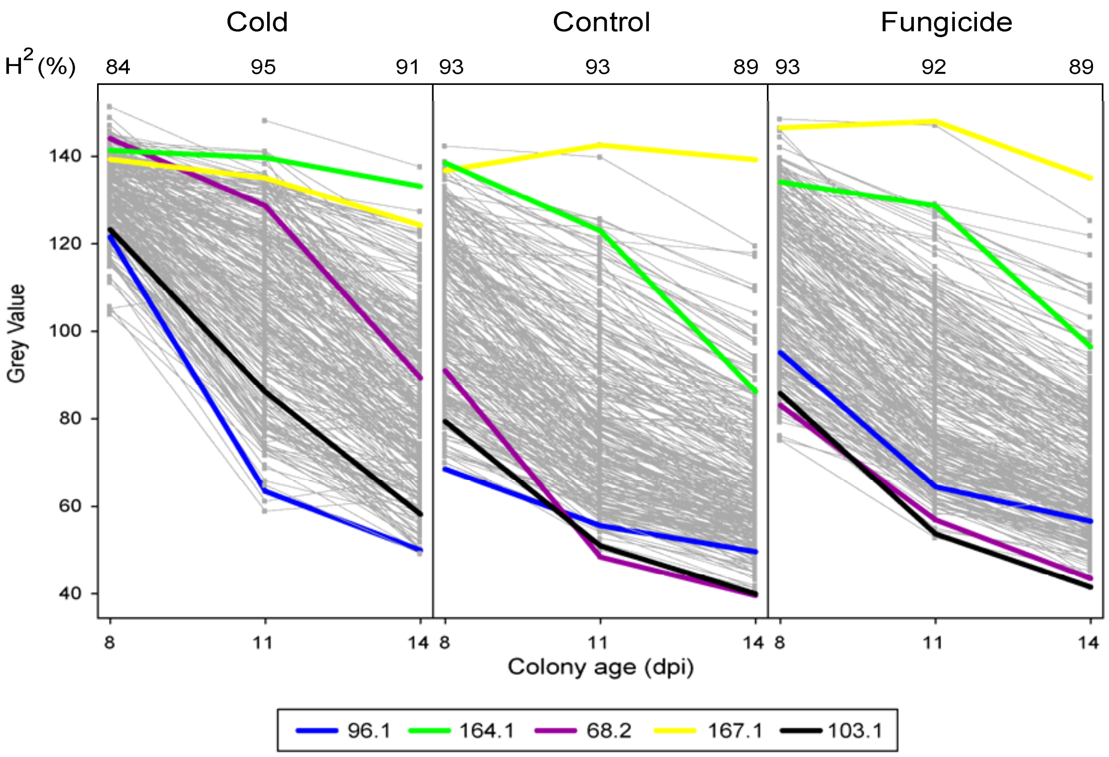

Figure S4 Norms of reaction for the cross 3D1 x 3D7 across the three colony ages and environments. The two progeny with the most extreme (highest and lowest), complete phenotypes found at 14 dpi colony age in each environment are highlighted in the two other environments. Broad-sense heritability (H^2) values are indicated for each ECAM phenotype.

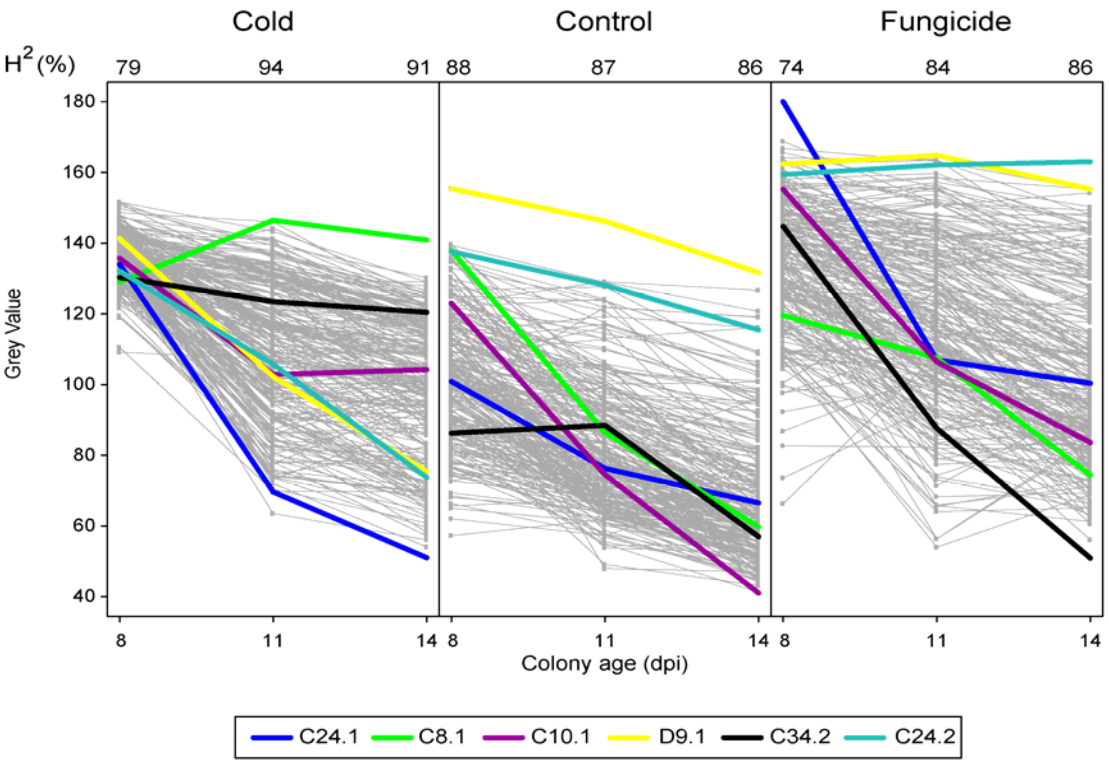

Figure S5 Norms of reaction for the cross 1A5 x1E4 across the three colony ages and environments. The two progeny with the most extreme (highest and lowest), complete phenotypes found at 14 dpi colony age in each environment are highlighted in the two orhter environments. Broad-sense heritability (H^2) values are indicated for each ECAM phenotype.

File S1

Fiji Batch Macro

Important Macro Information:

The following macro can be used in Fiji. To run the macro, do the following:

1. Create a folder (e.g. 'Main Folder').
2. Place your JPEG images to be analyzed into the folder.
3. Create the following subfolders within the folder:
    a. FinalSummary
    b. HSVtemp
    c. Labels
    d. Macro Used
    e. Masks
    f. OutlinesAndBackground
    g. Results
4. Save the following macro-text in a normal text file into the folder 'Macro Used'.
5. Open ImageJ/Fiji and select from the menu 'Plugins -> Macros -> Run'.
6. Select the txt file, where the macro-text has been saved.
7. Select the 'Main Folder' and your macro should run automatically.

Additional notes:

1. The macro requires more or less circular colony growth of the target organism.
2. This macro as shown here is suitable for Fiji version 1.47i running on a mac computer. For a windows computer, singular forward slashes (/) need to be changed into double back slashes (\\), except for the forward slash in the command: (endsWith(list[i], "/").
3. The speed of the batch macro can be optimized be deactivating commands that generate masks (e.g all commands which contain 'HSVtemp'). Deactivation can be conducted by applying two forward slashes in front of the command.
4. To avoid scoring of noise particles (including the polygon selection created during the macro) and fused colonies, we recommend to apply a subset onto the individual image results tables, by selecting for only particles providing a roundness value above 0.9. We used a loop in R to do so.

5. Settings most likely need to be adjusted if using a different organism then *Z. tritici* and different light and camera settings. Under same light and camera settings, we were able to apply the macro also for other plant pathogenic fungi, such as *Rhynchosporium commune*.

Detailed information regarding camera and light setup can be found in Table S2 and Figure S1. Detailed information regarding camera settings can be found in Table S3.

THE BATCH MACRO:

```
//*********************************************************************************************
//Batch macro: "Image Analysis; Zymoseptoria trtici; Size and Grey Value Measure of Single Spore Colony on Petri Dish"

//Author: Mark Lendenmann, Plant Pathology Group, ETH Zurich

//Measures size and grey value of single spore colonies grown on Petri dishes.
//Creates single image results tables, labeled overlay masks on binary image, labeled outline masks on original image, overall summary table

//*********************************************************************************************
// 1. SET SCALE: e.g. pixels to square millimeters / SET MEASURMEMENTS – change this to suit images

open("/Users/lmark/Desktop/Fiji Macro on this Mac/Callibration_45cm_9468.JPG");
run("In");
makeLine(1980, 1338, 2250, 1338);
run("Set Scale...", "distance=270 known=10 pixel=1 unit=mm global");
close();

//*********************************************************************************************
// 2. BATCH PROCESS: IMAGE THRESHOLDING, LABELS AND MASKS SAVING OF EACH IMAGE, RESULTS WITH PARTICLE AREA AND MEAN GREY VALUE, OUTLINE MASKS SAVED

macro "Image Analysis Colony Sizes" {
```

```
dir = getDirectory("Choose a Directory ");
print ("Chose Directory:" + dir);
list = getFileList(dir);
print("Directory contains "+list.length+" files and subfolders");
setBatchMode(true);
for (i=0; i<list.length; i++) {
if (endsWith(list[i], "/")) {
print("File is a subfolder and is ignored");
}
else {
if (endsWith(list[i], "db")) {
print("File is a Thumbs.db file and is ignored");
}
else {
path = dir+list[i];
IJ.freeMemory();
print("Free memory:", call("ij.IJ.freeMemory"));
open(path);
name = File.getName(path);
nameshort = File.nameWithoutExtension;
print("Path:", path);
print("Name:", name);
print("Directory:", dir);

// run("Threshold...");
// Color Thresholder 1.46b
// Autogenerated macro, single images only!
min=newArray(3);
max=newArray(3);
filter=newArray(3);
a=getTitle();
run("HSB Stack");
```

```
run("Convert Stack to Images");
selectWindow("Hue");
saveAs("Jpeg", dir+"/HSVtemp/"+nameshort+"Hue.jpg");
rename("0");
selectWindow("Saturation");
saveAs("Jpeg", dir+"/HSVtemp/"+nameshort+"Saturation.jpg");
rename("1");
selectWindow("Brightness");
saveAs("Jpeg", dir+"/HSVtemp/"+nameshort+"Brightness.jpg");
rename("2");
min[0]=57;
max[0]=190;
filter[0]="stop";
min[1]=0;
max[1]=255;
filter[1]="pass";
min[2]=0;
max[2]=255;
filter[2]="pass";
for (j=0;j<3;j++){
selectWindow(""+j);
setThreshold(min[j], max[j]);
saveAs("Jpeg", dir+"/HSVtemp/"+nameshort+"HSVRed"+j+".jpg");
rename(j);
run("Convert to Mask");
saveAs("Jpeg", dir+"/HSVtemp/"+nameshort+"HSVRed"+j+".jpg");
rename(j);
if (filter[j]=="stop")  run("Invert");
}
imageCalculator("AND create", "0","1");
imageCalculator("AND create", "Result of 0","2");
for (k=0;k<3;k++){
```

```
selectWindow(""+k);
close();
}
selectWindow("Result of 0");
close();
selectWindow("Result of Result of 0");
rename(a);
// Colour Thresholding-------------

//*****************************************************************************************
        // 2.1 ANALYZE PARTICLES: NOTE: The Selections are saved to the RoiManager.
run("Set Measurements...", "area mean min fit shape redirect=None decimal=3");
run("Analyze Particles...", "size=0.70-Infinity circularity=0.3-1.00 show=[Overlay Masks] display clear include summarize add");

//*****************************************************************************************
                // 2.1.1 CALL PLUGIN TO SET LABEL BACKGROUND FILL COLOR, LABEL COLOR AND FONT
// !!!!! NOTE: Here you can make changes for the background of the labels, stroke-color (=outline color), width, fill and even for the labels color and font size!!!!!

call("ij.plugin.filter.ParticleAnalyzer.setFontSize", 50);
run("Overlay Options...", "stroke=yellow width=1 fill=cyan apply");
run("Labels...", "color=black font=30 show bold");

//*****************************************************************************************
        // 2.2 SAVE MASKS AND LABELS

saveAs("Jpeg", dir+"/Labels/"+nameshort+"label.jpg");
run("Remove Overlay");
saveAs("Jpeg", dir+"/Masks/"+nameshort+"mask.jpg");

//*****************************************************************************************
```

```
// 2.3 SAVE RESULTS FORMED ON ORIGINAL IMAGE

run("Clear Results");
open(path);
rename("D");
print("Path:", path);
print("Name:", name);
print("Directory:", dir);

//Note: We add a small polygon with a very low roundness, just so the RoiManager isn’t empty!

makePolygon(4102,3206,4099,3207,4106,3206,4104,3204);
roiManager("Add");
roiManager("Measure");
selectWindow("D");
roiManager("Set Color", "yellow");
roiManager("Set Line Width", 2.5);
run("From ROI Manager");
run("Labels...", "color=yellow font=30 show");
saveAs("Jpeg", dir+"/OutlinesAndBackground/"+nameshort+"outline.jpg");
run("Close");
run("Select None");
selectWindow("Results");
saveAs("Text", dir+"/Results/"+nameshort+"results.txt");
selectWindow("Results");
run("Close");
selectWindow("Log");
run("Close");
roiManager("Delete");
} //ends else if statement
} //ends else statement
} // end for loop batch processing complete folder
```

```
//*****************************************************************************************
// 3. SAVE THE FINAL SUMMARY TO A NEW FOLDER NAMED "SUMMARY"


selectWindow("Summary");
saveAs("Text", dir+"/FinalSummary/"+"Final"+"summary.txt");
run("Close");
print("Macro is finished");
beep();


} //end macro
```
