# Supplementary material for: Quantitative Trait Locus Mapping of Melanization in the Plant Pathogenic Fungus Zymoseptoria tritici
Source: G3 (Bethesda). 2014 Oct 29;4(12):2519–33. doi: 10.1534/g3.114.015289 (PMC4267946; doi:10.1534/g3.114.015289)
Supplement: Supporting Information [file supp_g3.114.015289_TableS1.pdf]

Table S1 BioProject and sample accession numbers for the quality filter retained progeny used in the QTL analysis for each of the two crosses.

Cross	Retained progeny for QTL analysis	NCBI BioProject accession	NCBI SRA sample accession
3D1 x 3D7	1.1	PRJNA256988	SRS670337
3D1 x 3D7	1.2	PRJNA256988	SRS670339
3D1 x 3D7	10.1	PRJNA256988	SRS670338
3D1 x 3D7	100.1	PRJNA256988	SRS670340
3D1 x 3D7	100.2	PRJNA256988	SRS670342
3D1 x 3D7	101.2	PRJNA256988	SRS670344
3D1 x 3D7	102.1	PRJNA256988	SRS670345
3D1 x 3D7	102.2	PRJNA256988	SRS670346
3D1 x 3D7	103.1	PRJNA256988	SRS670347
3D1 x 3D7	104.1	PRJNA256988	SRS670349
3D1 x 3D7	104.2	PRJNA256988	SRS670350
3D1 x 3D7	105.1	PRJNA256988	SRS670351
3D1 x 3D7	105.2	PRJNA256988	SRS670352
3D1 x 3D7	106.1	PRJNA256988	SRS670353
3D1 x 3D7	106.2	PRJNA256988	SRS670355
3D1 x 3D7	107.1	PRJNA256988	SRS670354
3D1 x 3D7	108.1	PRJNA256988	SRS670356
3D1 x 3D7	108.2	PRJNA256988	SRS670358
3D1 x 3D7	109.1	PRJNA256988	SRS670359
3D1 x 3D7	109.2	PRJNA256988	SRS670360
3D1 x 3D7	11.1	PRJNA256988	SRS670361
3D1 x 3D7	11.2	PRJNA256988	SRS670362
3D1 x 3D7	110.1	PRJNA256988	SRS670363
3D1 x 3D7	110.2	PRJNA256988	SRS670365
3D1 x 3D7	111.2	PRJNA256988	SRS670367
3D1 x 3D7	112.1	PRJNA256988	SRS670366
3D1 x 3D7	113.1	PRJNA256988	SRS670369
3D1 x 3D7	113.2	PRJNA256988	SRS670370
3D1 x 3D7	114.1	PRJNA256988	SRS670371
3D1 x 3D7	114.2	PRJNA256988	SRS670372
3D1 x 3D7	115.1	PRJNA256988	SRS670373
3D1 x 3D7	116.1	PRJNA256988	SRS670375
3D1 x 3D7	116.2	PRJNA256988	SRS670376
3D1 x 3D7	117.1	PRJNA256988	SRS670378
3D1 x 3D7	117.2	PRJNA256988	SRS670377
3D1 x 3D7	118.1	PRJNA256988	SRS670380
3D1 x 3D7	118.2	PRJNA256988	SRS670379
3D1 x 3D7	119.1	PRJNA256988	SRS670381
3D1 x 3D7	119.2	PRJNA256988	SRS670382

3D1 x 3D7	12.1	PRJNA256988	SRS670383
3D1 x 3D7	120.1	PRJNA256988	SRS670385
3D1 x 3D7	120.2	PRJNA256988	SRS670386
3D1 x 3D7	121.2	PRJNA256988	SRS670388
3D1 x 3D7	122.1	PRJNA256988	SRS670389
3D1 x 3D7	123.1	PRJNA256988	SRS670391
3D1 x 3D7	125.1	PRJNA256988	SRS670395
3D1 x 3D7	126.1	PRJNA256988	SRS670397
3D1 x 3D7	127.1	PRJNA256988	SRS670398
3D1 x 3D7	127.2	PRJNA256988	SRS670399
3D1 x 3D7	128.1	PRJNA256988	SRS670400
3D1 x 3D7	128.2	PRJNA256988	SRS670401
3D1 x 3D7	129.2	PRJNA256988	SRS670403
3D1 x 3D7	13.1	PRJNA256988	SRS670404
3D1 x 3D7	130.1	PRJNA256988	SRS670406
3D1 x 3D7	131.1	PRJNA256988	SRS670408
3D1 x 3D7	133.1	PRJNA256988	SRS670413
3D1 x 3D7	134.1	PRJNA256988	SRS670414
3D1 x 3D7	135.2	PRJNA256988	SRS670418
3D1 x 3D7	136.1	PRJNA256988	SRS670417
3D1 x 3D7	137.1	PRJNA256988	SRS670419
3D1 x 3D7	137.2	PRJNA256988	SRS670420
3D1 x 3D7	138.1	PRJNA256988	SRS670421
3D1 x 3D7	139.2	PRJNA256988	SRS670423
3D1 x 3D7	14.1	PRJNA256988	SRS670425
3D1 x 3D7	141.1	PRJNA256988	SRS670429
3D1 x 3D7	142.1	PRJNA256988	SRS670430
3D1 x 3D7	142.2	PRJNA256988	SRS670431
3D1 x 3D7	143.1	PRJNA256988	SRS670432
3D1 x 3D7	144.1	PRJNA256988	SRS670434
3D1 x 3D7	145.1	PRJNA256988	SRS670437
3D1 x 3D7	145.2	PRJNA256988	SRS670436
3D1 x 3D7	146.1	PRJNA256988	SRS670438
3D1 x 3D7	146.2	PRJNA256988	SRS670440
3D1 x 3D7	147.1	PRJNA256988	SRS670439
3D1 x 3D7	148.1	PRJNA256988	SRS670443
3D1 x 3D7	149.1	PRJNA256988	SRS670444
3D1 x 3D7	149.2	PRJNA256988	SRS670445
3D1 x 3D7	15.1	PRJNA256988	SRS670446
3D1 x 3D7	15.2	PRJNA256988	SRS670447
3D1 x 3D7	150.1	PRJNA256988	SRS670448
3D1 x 3D7	150.2	PRJNA256988	SRS670449
3D1 x 3D7	151.1	PRJNA256988	SRS670452
3D1 x 3D7	152.1	PRJNA256988	SRS670451

3D1 x 3D7	152.2	PRJNA256988	SRS670453
3D1 x 3D7	154.1	PRJNA256988	SRS670456
3D1 x 3D7	154.2	PRJNA256988	SRS670458
3D1 x 3D7	155.1	PRJNA256988	SRS670457
3D1 x 3D7	156.1	PRJNA256988	SRS670461
3D1 x 3D7	157.1	PRJNA256988	SRS670462
3D1 x 3D7	158.1	PRJNA256988	SRS670464
3D1 x 3D7	158.2	PRJNA256988	SRS670465
3D1 x 3D7	159.1	PRJNA256988	SRS670466
3D1 x 3D7	16.1	PRJNA256988	SRS670468
3D1 x 3D7	160.1	PRJNA256988	SRS670470
3D1 x 3D7	160.2	PRJNA256988	SRS670471
3D1 x 3D7	161.1	PRJNA256988	SRS670472
3D1 x 3D7	161.2	PRJNA256988	SRS670473
3D1 x 3D7	162.1	PRJNA256988	SRS670474
3D1 x 3D7	162.2	PRJNA256988	SRS670475
3D1 x 3D7	163.1	PRJNA256988	SRS670476
3D1 x 3D7	164.1	PRJNA256988	SRS670478
3D1 x 3D7	164.2	PRJNA256988	SRS670479
3D1 x 3D7	165.1	PRJNA256988	SRS670480
3D1 x 3D7	165.2	PRJNA256988	SRS670481
3D1 x 3D7	166.1	PRJNA256988	SRS670482
3D1 x 3D7	167.1	PRJNA256988	SRS670484
3D1 x 3D7	167.2	PRJNA256988	SRS670485
3D1 x 3D7	168.1	PRJNA256988	SRS670486
3D1 x 3D7	169.1	PRJNA256988	SRS670487
3D1 x 3D7	169.2	PRJNA256988	SRS670488
3D1 x 3D7	17.2	PRJNA256988	SRS670492
3D1 x 3D7	170.1	PRJNA256988	SRS670491
3D1 x 3D7	170.2	PRJNA256988	SRS670493
3D1 x 3D7	171.1	PRJNA256988	SRS670494
3D1 x 3D7	172.1	PRJNA256988	SRS670496
3D1 x 3D7	173.1	PRJNA256988	SRS670498
3D1 x 3D7	174.1	PRJNA256988	SRS670499
3D1 x 3D7	174.2	PRJNA256988	SRS670501
3D1 x 3D7	175.1	PRJNA256988	SRS670502
3D1 x 3D7	176.2	PRJNA256988	SRS670505
3D1 x 3D7	177.2	PRJNA256988	SRS670507
3D1 x 3D7	178.1	PRJNA256988	SRS670508
3D1 x 3D7	179.1	PRJNA256988	SRS670510
3D1 x 3D7	179.2	PRJNA256988	SRS670511
3D1 x 3D7	18.1	PRJNA256988	SRS670512
3D1 x 3D7	18.2	PRJNA256988	SRS670513

3D1 x 3D7	180.1	PRJNA256988	SRS670514
3D1 x 3D7	180.2	PRJNA256988	SRS670515
3D1 x 3D7	181.1	PRJNA256988	SRS670516
3D1 x 3D7	181.2	PRJNA256988	SRS670518
3D1 x 3D7	182.1	PRJNA256988	SRS670517
3D1 x 3D7	183.1	PRJNA256988	SRS670520
3D1 x 3D7	184.1	PRJNA256988	SRS670521
3D1 x 3D7	185.1	PRJNA256988	SRS670522
3D1 x 3D7	19.1	PRJNA256988	SRS670524
3D1 x 3D7	19.2	PRJNA256988	SRS670525
3D1 x 3D7	2.1	PRJNA256988	SRS670526
3D1 x 3D7	20.1	PRJNA256988	SRS670528
3D1 x 3D7	20.2	PRJNA256988	SRS670529
3D1 x 3D7	21.1	PRJNA256988	SRS670530
3D1 x 3D7	21.2	PRJNA256988	SRS670531
3D1 x 3D7	22.1	PRJNA256988	SRS670532
3D1 x 3D7	22.2	PRJNA256988	SRS670533
3D1 x 3D7	23.1	PRJNA256988	SRS670534
3D1 x 3D7	23.2	PRJNA256988	SRS670536
3D1 x 3D7	24.1	PRJNA256988	SRS670535
3D1 x 3D7	24.2	PRJNA256988	SRS670537
3D1 x 3D7	25.1	PRJNA256988	SRS670538
3D1 x 3D7	26.1	PRJNA256988	SRS670540
3D1 x 3D7	26.2	PRJNA256988	SRS670541
3D1 x 3D7	27.1	PRJNA256988	SRS670542
3D1 x 3D7	28.1	PRJNA256988	SRS670544
3D1 x 3D7	29.1	PRJNA256988	SRS670545
3D1 x 3D7	3.1	PRJNA256988	SRS670547
3D1 x 3D7	3.2	PRJNA256988	SRS670548
3D1 x 3D7	30.1	PRJNA256988	SRS670550
3D1 x 3D7	31.1	PRJNA256988	SRS670551
3D1 x 3D7	32.1	PRJNA256988	SRS670553
3D1 x 3D7	33.1	PRJNA256988	SRS670555
3D1 x 3D7	34.1	PRJNA256988	SRS670557
3D1 x 3D7	34.2	PRJNA256988	SRS670559
3D1 x 3D7	35.1	PRJNA256988	SRS670558
3D1 x 3D7	35.2	PRJNA256988	SRS670560
3D1 x 3D7	36.1	PRJNA256988	SRS670561
3D1 x 3D7	37.1	PRJNA256988	SRS670564
3D1 x 3D7	37.2	PRJNA256988	SRS670563
3D1 x 3D7	38.1	PRJNA256988	SRS670565
3D1 x 3D7	39.1	PRJNA256988	SRS670567
3D1 x 3D7	4.1	PRJNA256988	SRS670569
3D1 x 3D7	4.2	PRJNA256988	SRS670570

3D1 x 3D7	40.1	PRJNA256988	SRS670572
3D1 x 3D7	40.2	PRJNA256988	SRS670571
3D1 x 3D7	41.1	PRJNA256988	SRS670573
3D1 x 3D7	41.2	PRJNA256988	SRS670575
3D1 x 3D7	42.1	PRJNA256988	SRS670574
3D1 x 3D7	42.2	PRJNA256988	SRS670576
3D1 x 3D7	43.1	PRJNA256988	SRS670577
3D1 x 3D7	44.1	PRJNA256988	SRS670580
3D1 x 3D7	45.1	PRJNA256988	SRS670581
3D1 x 3D7	46.1	PRJNA256988	SRS670583
3D1 x 3D7	46.2	PRJNA256988	SRS670584
3D1 x 3D7	47.1	PRJNA256988	SRS670585
3D1 x 3D7	48.1	PRJNA256988	SRS670587
3D1 x 3D7	48.2	PRJNA256988	SRS670588
3D1 x 3D7	49.1	PRJNA256988	SRS670589
3D1 x 3D7	49.2	PRJNA256988	SRS670590
3D1 x 3D7	5.1	PRJNA256988	SRS670591
3D1 x 3D7	5.2	PRJNA256988	SRS670592
3D1 x 3D7	50.1	PRJNA256988	SRS670593
3D1 x 3D7	51.1	PRJNA256988	SRS670596
3D1 x 3D7	51.2	PRJNA256988	SRS670595
3D1 x 3D7	52.1	PRJNA256988	SRS670597
3D1 x 3D7	52.2	PRJNA256988	SRS670598
3D1 x 3D7	53.2	PRJNA256988	SRS670600
3D1 x 3D7	54.1	PRJNA256988	SRS670601
3D1 x 3D7	55.1	PRJNA256988	SRS670604
3D1 x 3D7	55.2	PRJNA256988	SRS670603
3D1 x 3D7	56.1	PRJNA256988	SRS670605
3D1 x 3D7	56.2	PRJNA256988	SRS670606
3D1 x 3D7	57.1	PRJNA256988	SRS670607
3D1 x 3D7	57.2	PRJNA256988	SRS670609
3D1 x 3D7	58.1	PRJNA256988	SRS670608
3D1 x 3D7	59.1	PRJNA256988	SRS670611
3D1 x 3D7	59.2	PRJNA256988	SRS670612
3D1 x 3D7	6.1	PRJNA256988	SRS670614
3D1 x 3D7	60.2	PRJNA256988	SRS670613
3D1 x 3D7	61.1	PRJNA256988	SRS670615
3D1 x 3D7	62.1	PRJNA256988	SRS670617
3D1 x 3D7	62.2	PRJNA256988	SRS670618
3D1 x 3D7	63.1	PRJNA256988	SRS670619
3D1 x 3D7	64.1	PRJNA256988	SRS670621
3D1 x 3D7	65.1	PRJNA256988	SRS670623
3D1 x 3D7	66.1	PRJNA256988	SRS670624

3D1 x 3D7	67.1	PRJNA256988	SRS670626
3D1 x 3D7	68.1	PRJNA256988	SRS670629
3D1 x 3D7	68.2	PRJNA256988	SRS670630
3D1 x 3D7	69.1	PRJNA256988	SRS670631
3D1 x 3D7	69.2	PRJNA256988	SRS670633
3D1 x 3D7	7.1	PRJNA256988	SRS670632
3D1 x 3D7	70.1	PRJNA256988	SRS670635
3D1 x 3D7	71.2	PRJNA256988	SRS670636
3D1 x 3D7	72.1	PRJNA256988	SRS670637
3D1 x 3D7	72.2	PRJNA256988	SRS670638
3D1 x 3D7	73.1	PRJNA256988	SRS670639
3D1 x 3D7	74.1	PRJNA256988	SRS670641
3D1 x 3D7	75.1	PRJNA256988	SRS670644
3D1 x 3D7	75.2	PRJNA256988	SRS670643
3D1 x 3D7	76.1	PRJNA256988	SRS670646
3D1 x 3D7	76.2	PRJNA256988	SRS670645
3D1 x 3D7	77.1	PRJNA256988	SRS670647
3D1 x 3D7	78.1	PRJNA256988	SRS670650
3D1 x 3D7	79.1	PRJNA256988	SRS670651
3D1 x 3D7	8.1	PRJNA256988	SRS670653
3D1 x 3D7	8.2	PRJNA256988	SRS670654
3D1 x 3D7	80.1	PRJNA256988	SRS670655
3D1 x 3D7	80.2	PRJNA256988	SRS670656
3D1 x 3D7	82.1	PRJNA256988	SRS670659
3D1 x 3D7	82.2	PRJNA256988	SRS670660
3D1 x 3D7	83.1	PRJNA256988	SRS670661
3D1 x 3D7	83.2	PRJNA256988	SRS670662
3D1 x 3D7	84.1	PRJNA256988	SRS670663
3D1 x 3D7	84.2	PRJNA256988	SRS670664
3D1 x 3D7	86.2	PRJNA256988	SRS670668
3D1 x 3D7	87.1	PRJNA256988	SRS670669
3D1 x 3D7	88.1	PRJNA256988	SRS670672
3D1 x 3D7	89.1	PRJNA256988	SRS670674
3D1 x 3D7	89.2	PRJNA256988	SRS670673
3D1 x 3D7	9.1	PRJNA256988	SRS670675
3D1 x 3D7	90.1	PRJNA256988	SRS670677
3D1 x 3D7	91.2	PRJNA256988	SRS670679
3D1 x 3D7	92.1	PRJNA256988	SRS670680
3D1 x 3D7	93.1	PRJNA256988	SRS670682
3D1 x 3D7	93.2	PRJNA256988	SRS670683
3D1 x 3D7	94.1	PRJNA256988	SRS670685
3D1 x 3D7	94.2	PRJNA256988	SRS670684
3D1 x 3D7	95.1	PRJNA256988	SRS670686
3D1 x 3D7	95.2	PRJNA256988	SRS670687

3D1 x 3D7	96.1	PRJNA256988	SRS670688
3D1 x 3D7	96.2	PRJNA256988	SRS670689
3D1 x 3D7	97.1	PRJNA256988	SRS670690
3D1 x 3D7	97.2	PRJNA256988	SRS670691
3D1 x 3D7	98.1	PRJNA256988	SRS670693
3D1 x 3D7	99.1	PRJNA256988	SRS670694
1A5 x 1E4	A1.1	PRJNA256991	SRS670696
1A5 x 1E4	A10.1	PRJNA256991	SRS670697
1A5 x 1E4	A11.1	PRJNA256991	SRS670699
1A5 x 1E4	A11.2	PRJNA256991	SRS670700
1A5 x 1E4	A12.2	PRJNA256991	SRS670701
1A5 x 1E4	A13.1	PRJNA256991	SRS670702
1A5 x 1E4	A13.2	PRJNA256991	SRS670704
1A5 x 1E4	A14.1	PRJNA256991	SRS670705
1A5 x 1E4	A14.2	PRJNA256991	SRS670706
1A5 x 1E4	A16.1	PRJNA256991	SRS670707
1A5 x 1E4	A16.2	PRJNA256991	SRS670708
1A5 x 1E4	A17.1	PRJNA256991	SRS670709
1A5 x 1E4	A18.1	PRJNA256991	SRS670711
1A5 x 1E4	A18.2	PRJNA256991	SRS670712
1A5 x 1E4	A19.1	PRJNA256991	SRS670713
1A5 x 1E4	A2.2	PRJNA256991	SRS670716
1A5 x 1E4	A21.1	PRJNA256991	SRS670717
1A5 x 1E4	A21.2	PRJNA256991	SRS670718
1A5 x 1E4	A22.1	PRJNA256991	SRS670720
1A5 x 1E4	A22.2	PRJNA256991	SRS670719
1A5 x 1E4	A23.1	PRJNA256991	SRS670722
1A5 x 1E4	A23.2	PRJNA256991	SRS670721
1A5 x 1E4	A24.1	PRJNA256991	SRS670723
1A5 x 1E4	A24.2	PRJNA256991	SRS670724
1A5 x 1E4	A25.1	PRJNA256991	SRS670725
1A5 x 1E4	A25.2	PRJNA256991	SRS670726
1A5 x 1E4	A26.1	PRJNA256991	SRS670727
1A5 x 1E4	A26.2	PRJNA256991	SRS670728
1A5 x 1E4	A28.1	PRJNA256991	SRS670729
1A5 x 1E4	A29.1	PRJNA256991	SRS670731
1A5 x 1E4	A3.1	PRJNA256991	SRS670733
1A5 x 1E4	A3.2	PRJNA256991	SRS670734
1A5 x 1E4	A30.1	PRJNA256991	SRS670735
1A5 x 1E4	A30.2	PRJNA256991	SRS670736
1A5 x 1E4	A31.1	PRJNA256991	SRS670738
1A5 x 1E4	A32.1	PRJNA256991	SRS670739
1A5 x 1E4	A33.1	PRJNA256991	SRS670741

1A5 x 1E4	A33.2	PRJNA256991	SRS670742
1A5 x 1E4	A35.2	PRJNA256991	SRS670744
1A5 x 1E4	A36.1	PRJNA256991	SRS670745
1A5 x 1E4	A37.1	PRJNA256991	SRS670747
1A5 x 1E4	A38.1	PRJNA256991	SRS670749
1A5 x 1E4	A39.1	PRJNA256991	SRS670751
1A5 x 1E4	A39.2	PRJNA256991	SRS670752
1A5 x 1E4	A4.1	PRJNA256991	SRS670753
1A5 x 1E4	A40.1	PRJNA256991	SRS670755
1A5 x 1E4	A40.2	PRJNA256991	SRS670756
1A5 x 1E4	A41.1	PRJNA256991	SRS670757
1A5 x 1E4	A42.1	PRJNA256991	SRS670760
1A5 x 1E4	A42.2	PRJNA256991	SRS670759
1A5 x 1E4	A43.1	PRJNA256991	SRS670762
1A5 x 1E4	A43.2	PRJNA256991	SRS670761
1A5 x 1E4	A44.1	PRJNA256991	SRS670763
1A5 x 1E4	A44.2	PRJNA256991	SRS670765
1A5 x 1E4	A45.1	PRJNA256991	SRS670766
1A5 x 1E4	A45.2	PRJNA256991	SRS670764
1A5 x 1E4	A46.1	PRJNA256991	SRS670767
1A5 x 1E4	A46.2	PRJNA256991	SRS670768
1A5 x 1E4	A47.1	PRJNA256991	SRS670769
1A5 x 1E4	A47.2	PRJNA256991	SRS670770
1A5 x 1E4	A48.1	PRJNA256991	SRS670771
1A5 x 1E4	A49.1	PRJNA256991	SRS670773
1A5 x 1E4	A5.1	PRJNA256991	SRS670775
1A5 x 1E4	A50.2	PRJNA256991	SRS670778
1A5 x 1E4	A51.2	PRJNA256991	SRS670779
1A5 x 1E4	A53.2	PRJNA256991	SRS670781
1A5 x 1E4	A54.1	PRJNA256991	SRS670780
1A5 x 1E4	A54.2	PRJNA256991	SRS670782
1A5 x 1E4	A55.1	PRJNA256991	SRS670783
1A5 x 1E4	A55.2	PRJNA256991	SRS670784
1A5 x 1E4	A57.1	PRJNA256991	SRS670785
1A5 x 1E4	A59.1	PRJNA256991	SRS670787
1A5 x 1E4	A59.2	PRJNA256991	SRS670788
1A5 x 1E4	A6.1	PRJNA256991	SRS670789
1A5 x 1E4	A60.1	PRJNA256991	SRS670791
1A5 x 1E4	A60.2	PRJNA256991	SRS670792
1A5 x 1E4	A62.1	PRJNA256991	SRS670793
1A5 x 1E4	A62.2	PRJNA256991	SRS670794
1A5 x 1E4	A63.1	PRJNA256991	SRS670795
1A5 x 1E4	A63.2	PRJNA256991	SRS670796
1A5 x 1E4	A64.1	PRJNA256991	SRS670797

1A5 x 1E4	A66.1	PRJNA256991	SRS670799
1A5 x 1E4	A66.2	PRJNA256991	SRS670800
1A5 x 1E4	A8.1	PRJNA256991	SRS670801
1A5 x 1E4	A9.1	PRJNA256991	SRS670803
1A5 x 1E4	A9.2	PRJNA256991	SRS670804
1A5 x 1E4	B1.1	PRJNA256991	SRS670805
1A5 x 1E4	B1.3	PRJNA256991	SRS670807
1A5 x 1E4	B10.1	PRJNA256991	SRS670808
1A5 x 1E4	B10.2	PRJNA256991	SRS670809
1A5 x 1E4	B11.1	PRJNA256991	SRS670811
1A5 x 1E4	B12.1	PRJNA256991	SRS670812
1A5 x 1E4	B13.2	PRJNA256991	SRS670815
1A5 x 1E4	B14.1	PRJNA256991	SRS670816
1A5 x 1E4	B15.1	PRJNA256991	SRS670818
1A5 x 1E4	B16.1	PRJNA256991	SRS670821
1A5 x 1E4	B16.2	PRJNA256991	SRS670820
1A5 x 1E4	B17.1	PRJNA256991	SRS670823
1A5 x 1E4	B17.2	PRJNA256991	SRS670824
1A5 x 1E4	B18.1	PRJNA256991	SRS670822
1A5 x 1E4	B18.2	PRJNA256991	SRS670826
1A5 x 1E4	B19.1	PRJNA256991	SRS670827
1A5 x 1E4	B19.2	PRJNA256991	SRS670825
1A5 x 1E4	B20.1	PRJNA256991	SRS670828
1A5 x 1E4	B20.2	PRJNA256991	SRS670829
1A5 x 1E4	B21.1	PRJNA256991	SRS670830
1A5 x 1E4	B22.1	PRJNA256991	SRS670831
1A5 x 1E4	B22.2	PRJNA256991	SRS670832
1A5 x 1E4	B23.1	PRJNA256991	SRS670833
1A5 x 1E4	B24.1	PRJNA256991	SRS670835
1A5 x 1E4	B24.2	PRJNA256991	SRS670836
1A5 x 1E4	B25.1	PRJNA256991	SRS670837
1A5 x 1E4	B26.1	PRJNA256991	SRS670839
1A5 x 1E4	B27.1	PRJNA256991	SRS670840
1A5 x 1E4	B28.1	PRJNA256991	SRS670842
1A5 x 1E4	B28.2	PRJNA256991	SRS670843
1A5 x 1E4	B29.1	PRJNA256991	SRS670844
1A5 x 1E4	B3.1	PRJNA256991	SRS670846
1A5 x 1E4	B30.1	PRJNA256991	SRS670847
1A5 x 1E4	B31.1	PRJNA256991	SRS670849
1A5 x 1E4	B31.2	PRJNA256991	SRS670850
1A5 x 1E4	B32.1	PRJNA256991	SRS670852
1A5 x 1E4	B32.2	PRJNA256991	SRS670851
1A5 x 1E4	B33.1	PRJNA256991	SRS670853

1A5 x 1E4	B33.2	PRJNA256991	SRS670854
1A5 x 1E4	B34.2	PRJNA256991	SRS670857
1A5 x 1E4	B35.1	PRJNA256991	SRS670856
1A5 x 1E4	B35.2	PRJNA256991	SRS670858
1A5 x 1E4	B37.1	PRJNA256991	SRS670859
1A5 x 1E4	B37.3	PRJNA256991	SRS670861
1A5 x 1E4	B38.1	PRJNA256991	SRS670862
1A5 x 1E4	B39.1	PRJNA256991	SRS670864
1A5 x 1E4	B39.2	PRJNA256991	SRS670865
1A5 x 1E4	B4.1	PRJNA256991	SRS670867
1A5 x 1E4	B4.2	PRJNA256991	SRS670868
1A5 x 1E4	B40.2	PRJNA256991	SRS670869
1A5 x 1E4	B41.1	PRJNA256991	SRS670870
1A5 x 1E4	B42.1	PRJNA256991	SRS670871
1A5 x 1E4	B42.2	PRJNA256991	SRS670872
1A5 x 1E4	B42.3	PRJNA256991	SRS670873
1A5 x 1E4	B43.1	PRJNA256991	SRS670874
1A5 x 1E4	B44.1	PRJNA256991	SRS670876
1A5 x 1E4	B45.1	PRJNA256991	SRS670878
1A5 x 1E4	B45.2	PRJNA256991	SRS670879
1A5 x 1E4	B46.1	PRJNA256991	SRS670880
1A5 x 1E4	B46.2	PRJNA256991	SRS670881
1A5 x 1E4	B48.1	PRJNA256991	SRS670883
1A5 x 1E4	B48.2	PRJNA256991	SRS670884
1A5 x 1E4	B49.1	PRJNA256991	SRS670885
1A5 x 1E4	B50.1	PRJNA256991	SRS670886
1A5 x 1E4	B50.2	PRJNA256991	SRS670888
1A5 x 1E4	B51.1	PRJNA256991	SRS670890
1A5 x 1E4	B7.1	PRJNA256991	SRS670891
1A5 x 1E4	B9.1	PRJNA256991	SRS670895
1A5 x 1E4	B9.2	PRJNA256991	SRS670896
1A5 x 1E4	C1.1	PRJNA256991	SRS670897
1A5 x 1E4	C1.2	PRJNA256991	SRS670898
1A5 x 1E4	C10.1	PRJNA256991	SRS670899
1A5 x 1E4	C11.1	PRJNA256991	SRS670901
1A5 x 1E4	C12.1	PRJNA256991	SRS670903
1A5 x 1E4	C12.2	PRJNA256991	SRS670904
1A5 x 1E4	C13.1	PRJNA256991	SRS670905
1A5 x 1E4	C14.1	PRJNA256991	SRS670907
1A5 x 1E4	C16.1	PRJNA256991	SRS670908
1A5 x 1E4	C16.2	PRJNA256991	SRS670910
1A5 x 1E4	C16.3	PRJNA256991	SRS670911
1A5 x 1E4	C17.1	PRJNA256991	SRS670912
1A5 x 1E4	C19.1	PRJNA256991	SRS670914

1A5 x 1E4	C2.1	PRJNA256991	SRS670917
1A5 x 1E4	C2.2	PRJNA256991	SRS670919
1A5 x 1E4	C20.1	PRJNA256991	SRS670918
1A5 x 1E4	C20.2	PRJNA256991	SRS670920
1A5 x 1E4	C21.1	PRJNA256991	SRS670921
1A5 x 1E4	C21.2	PRJNA256991	SRS670922
1A5 x 1E4	C22.1	PRJNA256991	SRS670923
1A5 x 1E4	C22.2	PRJNA256991	SRS670924
1A5 x 1E4	C23.1	PRJNA256991	SRS670925
1A5 x 1E4	C23.2	PRJNA256991	SRS670926
1A5 x 1E4	C24.1	PRJNA256991	SRS670927
1A5 x 1E4	C24.2	PRJNA256991	SRS670929
1A5 x 1E4	C25.1	PRJNA256991	SRS670928
1A5 x 1E4	C26.1	PRJNA256991	SRS670931
1A5 x 1E4	C27.1	PRJNA256991	SRS670933
1A5 x 1E4	C27.2	PRJNA256991	SRS670934
1A5 x 1E4	C28.1	PRJNA256991	SRS670935
1A5 x 1E4	C28.2	PRJNA256991	SRS670936
1A5 x 1E4	C29.2	PRJNA256991	SRS670938
1A5 x 1E4	C3.1	PRJNA256991	SRS670939
1A5 x 1E4	C3.2	PRJNA256991	SRS670940
1A5 x 1E4	C30.1	PRJNA256991	SRS670941
1A5 x 1E4	C30.2	PRJNA256991	SRS670942
1A5 x 1E4	C31.1	PRJNA256991	SRS670943
1A5 x 1E4	C32.1	PRJNA256991	SRS670946
1A5 x 1E4	C32.2	PRJNA256991	SRS670947
1A5 x 1E4	C33.1	PRJNA256991	SRS670948
1A5 x 1E4	C33.2	PRJNA256991	SRS670949
1A5 x 1E4	C34.1	PRJNA256991	SRS670950
1A5 x 1E4	C34.2	PRJNA256991	SRS670951
1A5 x 1E4	C35.1	PRJNA256991	SRS670952
1A5 x 1E4	C36.1	PRJNA256991	SRS670954
1A5 x 1E4	C38.1	PRJNA256991	SRS670956
1A5 x 1E4	C38.2	PRJNA256991	SRS670957
1A5 x 1E4	C4.1	PRJNA256991	SRS670958
1A5 x 1E4	C4.2	PRJNA256991	SRS670959
1A5 x 1E4	C41.1	PRJNA256991	SRS670960
1A5 x 1E4	C41.2	PRJNA256991	SRS670961
1A5 x 1E4	C42.1	PRJNA256991	SRS670962
1A5 x 1E4	C42.2	PRJNA256991	SRS670963
1A5 x 1E4	C44.1	PRJNA256991	SRS670964
1A5 x 1E4	C44.2	PRJNA256991	SRS670965
1A5 x 1E4	C45.1	PRJNA256991	SRS670966

1A5 x 1E4	C45.2	PRJNA256991	SRS670967
1A5 x 1E4	C48.1	PRJNA256991	SRS670968
1A5 x 1E4	C48.2	PRJNA256991	SRS670969
1A5 x 1E4	C49.1	PRJNA256991	SRS670970
1A5 x 1E4	C5.1	PRJNA256991	SRS670972
1A5 x 1E4	C5.2	PRJNA256991	SRS670973
1A5 x 1E4	C51.1	PRJNA256991	SRS670974
1A5 x 1E4	C51.2	PRJNA256991	SRS670975
1A5 x 1E4	C52.1	PRJNA256991	SRS670976
1A5 x 1E4	C53.1	PRJNA256991	SRS670977
1A5 x 1E4	C54.1	PRJNA256991	SRS670979
1A5 x 1E4	C55.1	PRJNA256991	SRS670981
1A5 x 1E4	C55.2	PRJNA256991	SRS670982
1A5 x 1E4	C56.1	PRJNA256991	SRS670983
1A5 x 1E4	C56.2	PRJNA256991	SRS670985
1A5 x 1E4	C57.1	PRJNA256991	SRS670986
1A5 x 1E4	C57.2	PRJNA256991	SRS670987
1A5 x 1E4	C59.2	PRJNA256991	SRS670989
1A5 x 1E4	C6.1	PRJNA256991	SRS670990
1A5 x 1E4	C60.1	PRJNA256991	SRS670992
1A5 x 1E4	C60.2	PRJNA256991	SRS670993
1A5 x 1E4	C61.1	PRJNA256991	SRS670994
1A5 x 1E4	C61.2	PRJNA256991	SRS670995
1A5 x 1E4	C62.1	PRJNA256991	SRS670996
1A5 x 1E4	C62.2	PRJNA256991	SRS670997
1A5 x 1E4	C63.1	PRJNA256991	SRS670998
1A5 x 1E4	C7.1	PRJNA256991	SRS671000
1A5 x 1E4	C7.2	PRJNA256991	SRS671001
1A5 x 1E4	C8.1	PRJNA256991	SRS671002
1A5 x 1E4	CR4_A1.1	PRJNA256991	SRS671004
1A5 x 1E4	CR4_A2.1	PRJNA256991	SRS671006
1A5 x 1E4	CR4_A2.2	PRJNA256991	SRS671007
1A5 x 1E4	CR4_A3.1	PRJNA256991	SRS671010
1A5 x 1E4	CR4_A3.2	PRJNA256991	SRS671012
1A5 x 1E4	CR4_A4.1	PRJNA256991	SRS671015
1A5 x 1E4	D1.1	PRJNA256991	SRS671014
1A5 x 1E4	D1.2	PRJNA256991	SRS671016
1A5 x 1E4	D1.3	PRJNA256991	SRS671018
1A5 x 1E4	D10.1	PRJNA256991	SRS671019
1A5 x 1E4	D11.1	PRJNA256991	SRS671021
1A5 x 1E4	D2.1	PRJNA256991	SRS671023
1A5 x 1E4	D3.1	PRJNA256991	SRS671026
1A5 x 1E4	D3.2	PRJNA256991	SRS671025
1A5 x 1E4	D4.1	PRJNA256991	SRS671027

1A5 x 1E4	D5.1	PRJNA256991	SRS671029
1A5 x 1E4	D7.1	PRJNA256991	SRS671031
1A5 x 1E4	D7.2	PRJNA256991	SRS671032
1A5 x 1E4	D7.3	PRJNA256991	SRS671033
1A5 x 1E4	D9.1	PRJNA256991	SRS671035
1A5 x 1E4	D9.2	PRJNA256991	SRS671038
