# Supplementary material for: Quantitative Trait Locus Mapping of Melanization in the Plant Pathogenic Fungus Zymoseptoria tritici
Source: G3 (Bethesda). 2014 Oct 29;4(12):2519–33. doi: 10.1534/g3.114.015289 (PMC4267946; doi:10.1534/g3.114.015289)
Supplement: Supporting Information [file supp_g3.114.015289_TableS2.pdf]

Table S2 Camera and light setup overview.

Parameter	Specification
Camera height	45 cm
Lights	Two boxes with each two light bulbs (set up as a X). Boxes are setup to the left and right!
Bulbs	PHILIPHS TL 20W/55 DE LUXE
Background	Blue paper (single layered)
Calibration reference	Millimeter grid paper
Focus reference	Petri dish lid with a labeling 'Focus Reference'
