# Supplementary material for: Quantitative Trait Locus Mapping of Melanization in the Plant Pathogenic Fungus Zymoseptoria tritici
Source: G3 (Bethesda). 2014 Oct 29;4(12):2519–33. doi: 10.1534/g3.114.015289 (PMC4267946; doi:10.1534/g3.114.015289)
Supplement: Supporting Information [file supp_g3.114.015289_TableS3.pdf]

Table S3 Detailed information on the camera and lens used during image taking, as well as detailed information regarding the camera settings.

Camera type / Parameter [a]	Specification / Settings
Body	CANON EOS 60D Body
Lens	CANON EF 50mm F/2.5 Compact Macro
ISO-Sensitivity	200
Aperture (Depth of field)	14
Shutter speed 1)	5
Focus point 2)	Set to the most right square
Light exposure measurement	full
Quality	RAW (5184x3456) and JPEG (5184x3456)
Perpheral illumin. Correct.	Enable
Red-eye reduc.	Disable
Flash control (Flash firing)	Disable
Expo.comp./AEB	Set to Zero (in the center)
Auto Lighting Optimizer	Standard
Picture Style	Standard
White Balance	AWB (=automatic)
WB Shift/BKT	0,0/±0
Color space	sRGB
ISO Auto	Max: 3200
Live View shoot.	Enable
AF mode	Live mode
Grid display	OFF
Aspect ratio	3:2
Expo.simulation	Enable
Silent shooting	Mode 1
Metering timer	4 sec.
File numbering	Continuous

[a] 1) The shutter speed was set to 5, because under the specific light settings this value provided optimal illumination conditions. 2) The focus point was not set onto the fungal colonies, but to a Petri dish lid, just beside the plate containing the colonies. This additional lid was labeled with 'Focus Reference'. This labeling was at the same height as the fungal colonies, thus resulting for all colonies in sharp images, even if the height of the medium varied a bit.
