# Supplementary material for: Quantitative Trait Locus Mapping of Melanization in the Plant Pathogenic Fungus Zymoseptoria tritici
Source: G3 (Bethesda). 2014 Oct 29;4(12):2519–33. doi: 10.1534/g3.114.015289 (PMC4267946; doi:10.1534/g3.114.015289)
Supplement: Supporting Information [file supp_g3.114.015289_TableS4.pdf]

Table S4 Melanization phenotypes (grey values) for the quality filter retained progeny used in the QTL analysis for each of the two crosses.

		Environment / Colony age (dpi)								
Cross	Retained progeny for QTL analysis	Cold / 8	Cold / 11	Cold / 14	Control / 8	Control / 11	Control / 14	Fungicide / 8	Fungicide / 11	Fungicide / 14
3D1 x 3D7	1.1	NA	93.04607873	63.78769444	NA	71.23581861	67.52683429	NA	67.02107424	69.7407959
3D1 x 3D7	1.2	138.9211667	122.9319667	97.11144524	96.55215	58.83029167	51.17904167	90.96024888	65.84066857	52.35253429
3D1 x 3D7	10.1	NA	64.8691709	49.19910134	NA	57.37409722	52.89087429	NA	58.08289303	61.88949621
3D1 x 3D7	100.1	126.0365062	96.51860995	84.74405776	84.4686653	54.38001237	41.18947667	97.21626125	61.62162222	43.78903833
3D1 x 3D7	100.2	NA	NA	NA	130.362	111.038	89.081	122.192	103.41	80.703
3D1 x 3D7	101.2	105.2208167	110.885	82.07116667	115.7047548	87.58248333	70.77283333	113.8538611	92.12066667	77.19388889
3D1 x 3D7	102.1	105.6240078	58.91512232	62.00044471	70.04411476	54.14168105	44.92877808	75.14588822	52.81095889	45.30180626
3D1 x 3D7	102.2	130.57	107.3673333	89.673	125.4268	123.7577667	98.14745	128.3555556	110.1217778	84.55083333
3D1 x 3D7	103.1	123.2223212	86.10474875	58.1981572	79.39884322	50.95954548	39.97316381	85.77439636	53.72039736	41.51781945
3D1 x 3D7	104.1	129.5802667	106.52175	100.1136917	84.41975333	68.6814	53.95695	79.29458333	70.58371667	56.54508334
3D1 x 3D7	104.2	128.1313236	97.07834538	77.17254615	120.69925	113.3807896	94.37626944	124.7285833	104.5802222	80.3585
3D1 x 3D7	105.1	136.8757474	120.7077669	105.5548362	125.8309911	70.91158182	59.40977424	114.7506416	68.71309821	58.40494
3D1 x 3D7	105.2	143.5365783	141.1571521	119.8104176	131.9361982	106.5099222	81.25990333	137.0743612	102.7411322	83.67342
3D1 x 3D7	106.1	139.6565128	114.8448463	77.57321724	128.167917	63.6189048	56.07898959	99.45640081	74.7566568	61.05086472
3D1 x 3D7	106.2	115.9161838	79.5166859	62.98536364	72.47211933	60.85494351	53.55747143	95.35758965	67.89552051	58.22254167
3D1 x 3D7	107.1	139.9931532	134.0789152	104.7542548	99.64869838	69.55389269	59.78968333	100.5925153	71.2904198	56.71587476
3D1 x 3D7	108.1	126.0041042	100.9846077	88.60353125	84.49662993	62.95990197	50.02795222	87.86632974	61.15597824	47.16040321
3D1 x 3D7	108.2	129.5529	82.22087905	56.18452	117.9318333	117.24645	97.407375	119.83958	110.3138833	85.44816667
3D1 x 3D7	109.1	135.3257329	120.0563643	97.52829116	82.16497106	65.55824286	61.74073047	82.79800085	67.83865263	58.78931581
3D1 x 3D7	109.2	137.3307708	123.0388448	93.01046042	96.44440355	67.56110829	52.35975952	100.2994008	72.21352232	56.7993125

3D1 x 3D7	11.1	137.6486214	131.1213452	108.9592876	132.0365407	90.66232009	66.06892381	133.1571107	109.75148	78.56545167
3D1 x 3D7	11.2	NA	121.8148423	111.8334222	NA	120.8221206	117.90772	NA	126.1377512	117.5103294
3D1 x 3D7	110.1	126.5874751	95.12559761	78.28639186	100.1105441	65.51498795	47.47087143	115.2536224	67.42445379	43.80073636
3D1 x 3D7	110.2	NA	NA	NA	125.4243611	106.8474572	85.39096667	122.3512603	110.7835679	90.86082619
3D1 x 3D7	111.2	137.30737	136.2373752	97.88299143	117.6417819	80.28050952	76.18885953	118.6178548	90.40874	77.8929
3D1 x 3D7	112.1	135.478825	115.7907415	97.84553665	105.8666501	68.53853043	48.48154286	118.8581524	74.87240171	53.75149107
3D1 x 3D7	113.1	125.3779453	95.21866615	76.047	85.39857165	62.78296942	53.28581778	97.93049	66.821415	54.44839
3D1 x 3D7	113.2	128.519147	133.066299	124.0499274	142.3264405	139.8238529	119.5206	148.5043167	147.0384889	125.2621607
3D1 x 3D7	114.1	138.3819095	124.2893086	105.4281481	96.22480025	66.47385357	54.10065476	99.12644524	68.84467762	54.68510667
3D1 x 3D7	114.2	133.3009271	121.1906575	80.90807242	125.2174758	78.20477222	55.09530286	116.1495555	75.72660556	60.96945833
3D1 x 3D7	115.1	136.0427333	98.37231537	71.32154925	89.0400375	63.88561667	48.7861197	108.78675	76.25120833	53.87179167
3D1 x 3D7	116.1	135.0430083	115.5107914	84.17046389	118.9417083	91.14092897	71.23753333	128.22167	96.84129	66.53856333
3D1 x 3D7	116.2	140.8755833	139.82436	78.72343333	93.04799778	73.22596111	58.11796667	98.49983333	72.07444444	58.56844444
3D1 x 3D7	117.1	132.4399439	122.5927791	106.0175239	119.3944039	81.66589767	67.64874889	112.8362233	76.85737111	57.38383143
3D1 x 3D7	117.2	128.52064	105.45836	79.7792	75.26450762	68.66133143	56.7680875	94.56266667	75.62916667	58.88944444
3D1 x 3D7	118.1	133.3702659	112.158625	83.27358667	119.619208	91.38438881	64.85122679	122.2293167	96.69215	64.49689143
3D1 x 3D7	118.2	126.11014	84.838625	55.55818333	138.3078333	123.9671667	117.181125	142.0044792	127.4886042	108.3779167
3D1 x 3D7	119.1	145.1185318	135.580901	117.24819	134.9084222	119.741765	101.1712333	139.3677143	123.6001867	110.5751833
3D1 x 3D7	119.2	139.7671636	133.0280805	99.67200237	127.5515459	99.94991549	75.25956643	126.7247514	89.38076064	73.23461905
3D1 x 3D7	12.1	139.7715	110.0047283	94.17649128	102.651645	66.20026864	56.78980476	108.3259117	72.55310973	61.16291869
3D1 x 3D7	120.1	134.7883886	105.9334518	79.61856238	133.1658166	95.6161536	67.51032576	135.1733952	93.09351571	65.90646667
3D1 x 3D7	120.2	124.2219805	110.072525	100.2247767	95.65480329	85.03769111	86.65640714	90.14887841	83.99976778	81.37065333
3D1 x 3D7	121.2	123.9392351	98.98418546	79.66360635	92.39195763	65.91036984	54.76798907	89.79158722	70.27571382	58.98644444
3D1 x 3D7	122.1	126.5892667	109.40377	80.8714	101.6230529	57.814995	48.83373333	98.04725	59.8783	46.02056667
3D1 x 3D7	123.1	NA	69.50102659	49.82799148	NA	58.8571529	46.36049889	NA	68.11835801	54.66151539

3D1 x 3D7	125.1	126.4556433	100.3504289	77.63635833	85.64143535	59.08528523	53.43250051	90.51655429	69.37335	61.18938809
3D1 x 3D7	126.1	139.5133892	118.4039468	94.91439377	76.11003998	66.28649052	64.25514187	75.358708	65.68804542	67.48054593
3D1 x 3D7	127.1	131.725375	117.4720167	104.2255	101.6777198	81.4786	68.09599333	124.9721167	90.83951667	75.69251667
3D1 x 3D7	127.2	143.4491733	122.447816	81.54710834	131.3147957	111.5763038	92.14050667	123.9024349	91.32499	63.9832
3D1 x 3D7	128.1	NA	105.0129791	76.99662611	NA	82.70266069	74.0846381	NA	113.1437882	99.79397253
3D1 x 3D7	128.2	127.0527224	78.39297549	59.571295	78.78503135	58.73308359	52.63618222	90.26637411	61.753475	50.01614445
3D1 x 3D7	129.2	140.1191589	114.5787879	93.51255278	134.9651768	119.3261459	93.968775	126.6757781	106.5582031	82.00331905
3D1 x 3D7	13.1	134.0823333	121.9055201	104.598197	116.7126463	79.55738214	61.99740833	117.3200201	87.98913532	73.77595577
3D1 x 3D7	130.1	136.1447291	122.906545	103.4695933	78.05342548	54.76757206	49.95019	97.02448333	63.06407	50.06703333
3D1 x 3D7	131.1	141.5348463	124.7765354	84.07406762	109.4931242	105.9982066	91.02191667	125.7177935	121.0286218	99.46412
3D1 x 3D7	133.1	131.9478242	106.4417907	67.59122038	97.33066485	73.30647811	58.20252214	113.4148019	95.20031684	69.50066061
3D1 x 3D7	134.1	143.5851279	134.3886446	102.1055175	124.3813	111.7503562	92.38336143	136.938979	126.3972179	110.295
3D1 x 3D7	135.2	130.4660128	114.7943829	97.77874911	127.2511994	99.90496112	77.859375	123.9033679	93.88124672	72.15154167
3D1 x 3D7	136.1	140.7493921	129.8525634	82.80071678	127.8523795	94.4159059	70.69312222	134.8109516	108.0835791	81.27556254
3D1 x 3D7	137.1	119.4746667	110.7685583	85.52414583	102.61695	73.00778333	49.91662	91.11275333	66.73592667	56.01036667
3D1 x 3D7	137.2	129.5483633	112.82077	92.908915	128.7930011	121.4635848	109.73014	131.7700625	119.1038375	107.63625
3D1 x 3D7	138.1	127.0638667	83.92144286	53.52492238	84.11145338	58.71760143	49.02142889	85.82568	63.03203	48.49969
3D1 x 3D7	139.2	125.9147446	108.3824429	107.074289	87.53323333	58.30098714	49.5000881	112.3985143	78.85935714	59.55893571
3D1 x 3D7	14.1	NA	83.32912603	53.33956	NA	49.97735758	41.21580381	NA	64.96018294	50.04632222
3D1 x 3D7	141.1	115.4414806	73.28415486	59.03199092	89.82217616	79.61292417	60.25002833	109.025493	86.46800278	70.04466888
3D1 x 3D7	142.1	125.3967	93.19292	61.25091	80.61012	64.16727	58.29271	83.57514333	82.07171	63.61969667
3D1 x 3D7	142.2	142.21608	123.4780691	103.7644203	98.01887413	62.77557698	50.43148333	92.194025	63.94450625	50.59447917
3D1 x 3D7	143.1	130.7703195	103.581716	73.65817778	119.7673708	92.76618333	69.39238032	137.1490394	114.8420785	83.41273809
3D1 x 3D7	144.1	138.1035984	133.8091562	70.73748056	106.6117286	80.84769157	65.92154254	82.92068762	65.62776071	59.47129333
3D1 x 3D7	145.1	135.3504	133.8312	86.7972	113.604875	69.32104167	51.56841667	95.634	69.729625	49.977875

3D1 x 3D7	145.2	129.2534468	73.03755654	56.01757783	79.4303338	59.82851984	54.35139405	88.94466165	62.80936379	53.76453056
3D1 x 3D7	146.1	118.8807422	81.40470303	62.60609658	74.08911486	58.54237652	47.38810556	92.77377802	69.4047919	60.45700377
3D1 x 3D7	146.2	132.6046987	111.6485549	74.14845667	122.1992651	101.986996	81.48734048	135.4102204	106.7135515	78.93662286
3D1 x 3D7	147.1	136.4732993	128.67055	108.254875	134.6993644	120.7140088	99.95066	139.6552089	124.4744369	106.8345488
3D1 x 3D7	148.1	136.286	114.7665762	82.628	125.8477333	117.1805	98.0468	137.6229	117.5911333	103.2995333
3D1 x 3D7	149.1	131.1865095	128.3453467	116.5734	112.1270984	73.51390333	49.46273333	94.54061667	63.88535	51.77802
3D1 x 3D7	149.2	120.8365625	95.95891667	75.98576667	98.12266667	69.509	54.48325	120.652875	104.4980625	83.12341667
3D1 x 3D7	15.1	NA	111.2883258	83.38954571	NA	79.41166167	60.071305	NA	72.57174698	56.74346091
3D1 x 3D7	15.2	NA	85.192125	53.33523667	NA	67.56582	44.65534583	NA	61.62569524	53.80952
3D1 x 3D7	150.1	125.2006901	96.7623936	62.65496821	114.4581669	91.90862167	70.32053929	126.4477333	102.5120111	80.92061
3D1 x 3D7	150.2	127.8079833	94.45016667	71.11658333	117.9136214	75.18476429	59.65614048	107.148	78.792	70.349
3D1 x 3D7	151.1	122.6230759	98.57915474	78.37785353	130.1197491	97.49294555	69.00558333	128.9237327	104.1192422	80.02399091
3D1 x 3D7	152.1	141.7708667	128.1424171	94.66597238	124.0634471	104.0821583	79.34974306	121.41195	96.74219	78.05893333
3D1 x 3D7	152.2	139.6088605	127.4561053	86.96286372	93.76765471	70.264686	60.49020409	90.42330261	75.80675778	64.27734476
3D1 x 3D7	154.1	138.0291878	120.4358417	100.9598698	115.1169251	86.2850923	66.31999048	124.7431726	94.19792538	68.14131399
3D1 x 3D7	154.2	133.12278	132.6397492	119.433145	116.4124	101.28245	72.76950333	113.42433	87.48600333	58.78476667
3D1 x 3D7	155.1	128.35642	91.59913333	62.19364444	91.89161623	74.82485119	54.38807778	104.7476958	75.24792262	58.17046072
3D1 x 3D7	156.1	120.3050869	73.37593952	63.11625915	90.66820417	71.42360812	56.7617875	88.0975903	70.99817429	56.71396571
3D1 x 3D7	157.1	145.3160951	108.641799	94.49760608	111.9562691	73.80657301	50.79755	106.2519157	75.28544077	55.66672061
3D1 x 3D7	158.1	144.9867352	99.01515128	63.72135032	85.61962574	60.51236923	52.34413	89.64194496	63.89058974	52.76340741
3D1 x 3D7	158.2	140.6205324	134.9761876	118.6019507	107.4968587	72.74571847	57.73827746	112.6076579	73.71987929	59.36658241
3D1 x 3D7	159.1	129.3485369	93.50156855	85.11393056	82.90811275	66.96638244	58.19407802	85.6230771	63.60724387	52.54960675
3D1 x 3D7	16.1	136.2823619	106.9365986	70.32432833	105.1598383	92.32704722	86.28127083	129.1850959	106.6898065	85.71427762
3D1 x 3D7	160.1	124.8917081	91.80438838	87.26488643	131.9349647	114.8857203	89.0324873	130.1053634	110.7333865	87.97914643
3D1 x 3D7	160.2	132.7578886	107.5048927	82.40952286	97.73144359	76.27823433	61.50911905	124.2041796	102.113184	76.92891429

3D1 x 3D7	161.1	114.9409848	82.71843261	79.32695781	83.57319937	67.72782061	58.15893	99.9556764	75.35892381	60.68038889
3D1 x 3D7	161.2	126.16632	88.48092736	60.78921611	115.965403	100.6948312	86.29	113.9808894	97.87364215	81.15535357
3D1 x 3D7	162.1	112.5389223	61.18032419	66.87168131	85.64414134	66.32368606	54.04496299	101.0176697	81.77553891	58.71008232
3D1 x 3D7	162.2	142.0265508	101.1079996	83.996875	120.0816425	87.86108921	76.23789286	115.9747656	78.46690183	66.89146578
3D1 x 3D7	163.1	116.2694698	79.69892045	62.5408549	89.94441995	62.28876657	46.71306806	85.43330952	69.03779977	52.04257833
3D1 x 3D7	164.1	141.3336511	139.7041262	133.1069132	138.5022579	122.944054	86.19689039	134.1091958	128.660429	96.45068538
3D1 x 3D7	164.2	130.4233825	77.79271731	63.12385139	85.29031857	66.18034675	56.76234921	111.9178215	74.39504266	59.81449167
3D1 x 3D7	165.1	141.7799278	120.2202652	85.1389498	130.8062332	86.82499984	64.84592292	119.3387083	85.5438497	68.66865833
3D1 x 3D7	165.2	140.5679498	126.6398297	94.29226089	103.5160775	77.99954265	65.83169047	95.78463277	74.20973939	58.44691587
3D1 x 3D7	166.1	140.6971626	122.0042207	114.9363918	88.64881645	64.79402064	56.74132484	106.7851129	84.66343045	63.83962937
3D1 x 3D7	167.1	139.3141667	135.0375986	124.2252	136.7217143	142.5445778	139.22575	146.5065333	147.9730857	135.04325
3D1 x 3D7	167.2	140.1945519	135.0509203	100.6235077	103.7432064	61.63875268	56.9507875	104.0934101	61.93783445	52.77663632
3D1 x 3D7	168.1	137.4919675	131.4617516	122.27475	126.7435308	112.2218591	72.28571191	109.4642303	100.3596607	70.47102143
3D1 x 3D7	169.1	121.9518131	91.7737569	69.72953667	106.9001767	91.21401799	80.64860278	135.9567168	109.0450082	86.70945873
3D1 x 3D7	169.2	133.4081591	104.632679	89.26455794	92.91455357	86.19270667	72.29893333	95.70178745	86.33760273	62.88875682
3D1 x 3D7	17.2	NA	78.02109722	54.53772727	NA	85.84417613	72.71295175	NA	96.9145714	81.59784473
3D1 x 3D7	170.1	133.4990222	112.38848	94.14356667	104.4950397	75.96337042	57.4673	91.141675	62.02766806	51.631275
3D1 x 3D7	170.2	130.4647887	122.1167192	99.50609158	96.46677897	73.62106374	53.98268816	81.29295325	64.64216982	51.46869637
3D1 x 3D7	171.1	136.9493321	101.8828103	77.79513857	113.2749764	80.69472718	63.04177083	113.3054646	83.92688542	67.251925
3D1 x 3D7	172.1	131.3999605	97.87915555	73.07184722	94.15807478	79.93741238	61.98920635	103.2158988	87.81111458	68.5316625
3D1 x 3D7	173.1	128.2914354	101.0742199	69.81236667	120.2945938	92.97012777	69.64448611	132.6136389	93.72208333	73.5067619
3D1 x 3D7	174.1	NA	133.973075	103.3649967	81.41447786	57.23125476	49.63572308	92.17682792	63.07896575	50.92446667
3D1 x 3D7	174.2	134.6040208	121.4943916	99.81489299	129.7922309	114.1100626	101.15172	137.0385239	112.3471531	98.18325556
3D1 x 3D7	175.1	136.9860548	127.7213121	98.38204366	98.94851281	71.53172436	56.63314048	101.5353816	76.76785069	61.03854524
3D1 x 3D7	176.2	135.6852333	134.4379333	94.76	88.8825381	64.45169047	56.53269667	89.58376111	61.75283492	54.26904762

3D1 x 3D7	177.2	133.1597735	103.0041777	71.98844051	85.96196895	80.39028286	78.73309167	94.20947329	91.685755	76.73945833
3D1 x 3D7	178.1	NA	NA	NA	NA	NA	NA	126.9862143	92.74939286	82.50085715
3D1 x 3D7	179.1	136.8075833	128.5307389	91.42731667	122.78785	88.36983306	77.86247222	128.5165	100.612375	84.09683333
3D1 x 3D7	179.2	132.0871729	102.2969964	84.54231337	89.65980145	64.50878737	55.3489295	93.37581436	66.74216143	59.45812667
3D1 x 3D7	18.1	NA	74.11730128	63.12752571	NA	70.05034295	73.37750333	NA	71.63110883	61.39760389
3D1 x 3D7	18.2	NA	79.52240393	51.92528031	NA	64.17108769	47.83613551	NA	96.81743163	78.69287397
3D1 x 3D7	180.1	130.928	125.6056111	110.2960417	102.5074846	61.14320089	48.77499762	101.8163208	67.18779167	59.23447917
3D1 x 3D7	180.2	123.4830833	91.92473651	82.19987143	93.62233333	69.86728571	53.272	122.2260441	96.3794127	70.87319444
3D1 x 3D7	181.1	138.5844167	118.37634	94.73722921	127.1647764	117.1652574	104.1304444	129.1146393	121.4279271	110.6032292
3D1 x 3D7	181.2	133.9570464	100.5583562	73.34261857	92.76504167	89.20709333	76.90155	90.9479006	77.88104091	61.97859281
3D1 x 3D7	182.1	138.8383854	132.047874	101.2305104	106.9959371	64.53352258	57.63130667	90.86887583	64.45460702	57.52971167
3D1 x 3D7	183.1	NA	NA	NA	NA	NA	NA	NA	NA	NA
3D1 x 3D7	184.1	NA	NA	NA	NA	NA	NA	NA	NA	NA
3D1 x 3D7	185.1	NA	NA	NA	NA	NA	NA	NA	NA	NA
3D1 x 3D7	19.1	138.7258714	107.6435993	82.88130148	103.3610352	78.20949838	61.11105111	117.9348998	94.24286286	73.7936619
3D1 x 3D7	19.2	NA	107.6364694	72.39309874	NA	85.75249216	56.85602167	NA	76.3162016	62.8778537
3D1 x 3D7	2.1	120.7387152	73.80284558	53.2984424	71.45976675	59.86445624	53.14225512	100.9353505	73.39745619	59.47159333
3D1 x 3D7	20.1	147.066	115.4841667	69.31626667	95.77294643	70.54279464	61.3963125	122.2411944	97.62733334	83.31825
3D1 x 3D7	20.2	123.4709953	97.40425237	63.45829606	NA	NA	NA	113.3631644	90.21282463	67.59146515
3D1 x 3D7	21.1	119.0331243	79.74781381	50.06561714	101.3145369	70.13502747	51.53922047	107.9206684	72.78840476	53.30451872
3D1 x 3D7	21.2	138.2668083	133.4046289	90.352745	100.6563	63.72740222	53.8289927	103.1368583	69.40558399	60.48088413
3D1 x 3D7	22.1	127.368559	72.98749167	51.847525	93.68857333	59.97400333	49.10106667	102.3140991	69.31290667	50.18473333
3D1 x 3D7	22.2	132.1485744	122.2830277	96.73257455	90.00063577	88.60251508	77.730375	114.7322311	93.48751581	81.75186905
3D1 x 3D7	23.1	140.7580371	112.4338281	87.59477607	96.36800265	63.78457273	56.1898875	110.0397162	71.49468462	56.49143778
3D1 x 3D7	23.2	111.2859571	72.89072999	69.66789171	72.92616186	60.61715912	51.36600897	80.51897992	64.78053619	57.30784974

3D1 x 3D7	24.1	125.5408957	91.2279461	73.49852576	97.42646881	85.0940594	82.83992333	135.9855603	98.26310206	92.04419333
3D1 x 3D7	24.2	122.6635302	74.30309643	57.48431317	92.277975	59.74193222	50.36335667	95.16678889	66.39898667	53.14889667
3D1 x 3D7	25.1	123.7440512	76.32463467	61.97325604	81.67686039	64.00770968	65.80936667	91.84036791	72.28121398	66.28256528
3D1 x 3D7	26.1	140.2089124	134.5376982	113.3056728	113.6351578	76.04276089	61.45908294	112.2144822	76.8064081	68.85675286
3D1 x 3D7	26.2	122.0893114	90.12150899	66.15026381	74.09280354	54.49137167	43.91788333	85.62801631	67.28101238	52.85703506
3D1 x 3D7	27.1	137.9265	97.268225	68.1778125	121.5021	100.93985	85.25397917	131.6998	96.7159	82.45925
3D1 x 3D7	28.1	140.1991462	135.1912308	119.8964695	115.6988642	79.92737254	79.81264948	113.6345348	83.9633832	72.56067293
3D1 x 3D7	29.1	136.9227365	94.89935517	66.83721091	110.1512011	73.20999159	67.25682222	117.3635191	76.75948	67.31321191
3D1 x 3D7	3.1	135.5114162	132.5341077	124.030381	130.4991552	99.91538214	85.47613524	144.4285324	113.2363191	94.00156667
3D1 x 3D7	3.2	NA	107.2703559	64.23136177	NA	67.22586355	52.98679189	NA	64.34867429	51.22074476
3D1 x 3D7	30.1	136.137124	100.6566617	80.20256547	83.55256528	61.18095386	53.02263854	88.22787638	59.62700433	46.67800013
3D1 x 3D7	31.1	140.299	131.90167	115.1181386	130.8839768	104.23676	81.53833	132.2737457	95.49844333	74.93745
3D1 x 3D7	32.1	139.6322857	133.0101176	92.56205882	105.6008301	84.40129808	60.34463636	122.6354031	103.2017243	80.88757446
3D1 x 3D7	33.1	137.7362917	85.02304286	50.20698809	129.2478433	84.59907667	53.65629	131.2358743	77.19615333	54.93131667
3D1 x 3D7	34.1	128.8797411	80.97609762	66.22621944	105.5443994	74.50585111	61.6895031	111.4458092	83.48721312	67.43619141
3D1 x 3D7	34.2	135.6100941	133.8769663	97.29378749	130.6120754	109.3446677	81.57846212	124.8864203	101.5228742	78.99150222
3D1 x 3D7	35.1	151.3156667	135.808625	107.8965833	118.443	80.05411333	64.34767778	103.7530875	90.82901667	80.89579167
3D1 x 3D7	35.2	129.0207497	106.7253499	78.1549258	105.7457097	77.39811111	66.00772917	136.0357192	99.6603437	78.43361119
3D1 x 3D7	36.1	129.9945	105.3896333	69.2704	99.90005	71.04260833	57.93026667	108.9648571	82.60601429	69.52265714
3D1 x 3D7	37.1	138.7162725	126.4002913	93.65840051	71.75059888	62.1588506	60.5575518	82.24823684	68.06613503	61.01281925
3D1 x 3D7	37.2	128.931	99.48766667	79.28011111	110.0875	76.6515	57.3565	97.5155	66.30625	48.82704167
3D1 x 3D7	38.1	NA	133.1666807	127.3755176	NA	116.1471719	70.44852164	NA	104.3004748	88.90872074
3D1 x 3D7	39.1	NA	131.3461301	93.78400151	NA	94.60087308	74.96788155	NA	89.10517679	66.84571429
3D1 x 3D7	4.1	NA	90.05499416	73.03173041	NA	52.72491286	46.65054381	NA	64.51305291	56.62683689
3D1 x 3D7	4.2	NA	124.1556813	73.6604533	NA	64.56066044	48.38919464	NA	69.79101837	56.30538159

3D1 x 3D7	40.1	141.70531	107.8079846	82.61208714	100.6079834	84.92258603	75.18412286	122.9637647	99.98711556	85.53970786
3D1 x 3D7	40.2	121.3964834	78.51984117	61.78659825	78.12761823	61.414025	49.45535	101.8484005	75.96920917	55.26850249
3D1 x 3D7	41.1	130.7753503	97.19234806	73.98085974	80.533316	57.7114879	53.42984048	97.68310368	78.7261676	62.48762976
3D1 x 3D7	41.2	117.689935	95.46783108	72.33974667	120.7719914	100.0805483	79.40846226	131.0665609	105.8263777	78.498605
3D1 x 3D7	42.1	142.536875	113.4659381	81.20591	131.0386572	125.636	109.2982334	131.7406417	129.0642292	121.8810209
3D1 x 3D7	42.2	139.9364979	128.3161324	115.6285059	137.440968	114.3296267	93.23377083	139.1647589	122.5143214	89.06352571
3D1 x 3D7	43.1	125.9104712	76.26142996	59.77047929	76.55469454	60.85553348	50.81685893	85.7241218	60.48627	50.08355026
3D1 x 3D7	44.1	135.1108	110.750666	76.83469026	104.6690036	73.41648379	57.1443697	122.8837675	98.8061257	75.23764611
3D1 x 3D7	45.1	133.2493889	109.9353779	87.20926458	113.0734917	84.49419515	76.42185	122.0764024	88.34963524	73.34068333
3D1 x 3D7	46.1	142.40395	131.890406	115.3515167	121.0491152	79.19920595	64.28520524	120.4966191	86.00835635	77.68723691
3D1 x 3D7	46.2	103.9169082	71.74250997	59.35801967	84.64489352	70.5434067	56.17210909	106.3485062	78.6240503	62.95410857
3D1 x 3D7	47.1	NA	148.15	137.5893667	98.138	78.86825	64.451	100.2993333	91.31076667	73.99536667
3D1 x 3D7	48.1	142.6662994	140.8874869	112.6236155	123.0763619	76.02267111	60.51474286	113.9076738	74.77128333	56.8428
3D1 x 3D7	48.2	132.7349751	89.82751422	72.52160694	101.9810254	72.28780165	58.44153997	119.3008538	74.49031914	56.38209286
3D1 x 3D7	49.1	104.866192	78.25491788	62.8686925	81.2022176	58.65756659	43.28791212	120.2614794	92.3313479	64.8911899
3D1 x 3D7	49.2	140.9522	118.044747	83.390575	124.3443333	103.61325	91.84045833	127.3345452	107.1449267	81.60776809
3D1 x 3D7	5.1	NA	117.4724124	81.57235068	NA	88.41194371	68.96772462	NA	110.6734782	89.4195342
3D1 x 3D7	5.2	136.4810257	95.6565312	70.62825176	118.5702153	96.29915757	73.24583905	116.0068305	82.71073195	67.58075296
3D1 x 3D7	50.1	138.1254355	118.2649729	96.53738788	121.2600262	98.56399498	81.08327	130.294369	98.5071941	83.51318857
3D1 x 3D7	51.1	130.0670719	88.1732157	50.34719958	116.4704617	100.4002129	86.17416667	138.0303553	118.9777818	97.89570001
3D1 x 3D7	51.2	145.73354	126.3671257	102.5568233	90.03249889	71.14110791	54.30990857	76.13278778	63.09383397	47.22846278
3D1 x 3D7	52.1	132.1225505	111.8326229	83.38598294	98.35742868	77.84063683	62.95922949	130.1691795	101.9171043	82.38034524
3D1 x 3D7	52.2	145.1371458	138.333125	105.03245	102.8240625	83.50347917	64.64826042	112.9603583	75.57325	59.4755
3D1 x 3D7	53.2	144.7885	134.3973867	121.74444	136.7690417	104.0823125	79.99897917	134.1033438	112.0042042	97.91391667
3D1 x 3D7	54.1	125.7245038	107.8008033	96.61177667	95.91757393	77.67038	62.11016667	115.3815275	90.43367347	65.33060674

3D1 x 3D7	55.1	141.002075	133.5922761	116.6712278	91.99541047	70.9634	60.9066373	100.0572597	76.32626667	64.16302286
3D1 x 3D7	55.2	148.8433191	118.5140814	93.24240555	122.0431391	87.61767833	61.74316667	128.2704688	92.63234524	64.5694
3D1 x 3D7	56.1	126.2931533	99.61779999	90.84006667	72.43056786	59.49861429	51.64427119	107.7752924	80.46331472	62.45433939
3D1 x 3D7	56.2	143.7479312	117.6152838	84.11179417	112.3781365	89.13699818	67.38544667	103.633403	83.66261667	65.72513333
3D1 x 3D7	57.1	131.310289	108.6622561	79.3535651	132.1836299	106.4823254	70.67171929	145.8548178	108.3602286	80.40545333
3D1 x 3D7	57.2	136.3089227	101.7280267	68.54899584	120.1614797	102.9056305	86.48826857	132.9383929	108.1800977	86.24935
3D1 x 3D7	58.1	141.2475465	122.043441	103.3169964	121.9814364	77.82913603	61.93803531	115.7518433	78.52296357	62.99389238
3D1 x 3D7	59.1	137.9244676	120.4946433	93.36255651	109.0218792	66.16379343	56.00400833	100.3726743	67.16583788	53.87725555
3D1 x 3D7	59.2	132.60078	81.15803048	50.39966	82.54733766	56.68342857	46.95882857	89.71904167	60.40077976	49.0646625
3D1 x 3D7	6.1	NA	93.41434254	68.84771718	NA	57.95341286	45.03879365	NA	69.62722917	66.09039881
3D1 x 3D7	60.2	145.262375	130.9526875	98.77077778	114.6155667	61.89401428	50.57148667	102.9869	60.81056667	50.03586667
3D1 x 3D7	61.1	124.452478	87.75384171	65.20402111	75.40455953	63.22999915	55.97930339	91.75474965	64.07253333	55.5072739
3D1 x 3D7	62.1	125.9933989	79.39557802	59.69427473	106.5580009	74.18817016	51.14752198	128.5982939	100.96777	63.98341
3D1 x 3D7	62.2	124.4408159	76.57061762	65.62779167	78.01873371	58.81243131	53.98816381	93.67488833	66.53611309	54.87406667
3D1 x 3D7	63.1	133.1737375	118.0319444	88.19184653	112.8804551	88.40741286	60.84107429	126.1854829	82.33631333	61.41224286
3D1 x 3D7	64.1	NA	106.5487444	76.49073111	NA	95.31878207	71.59089	NA	66.42441441	56.28366806
3D1 x 3D7	65.1	129.4267262	121.809479	91.16494127	117.4588334	94.79097143	70.55424405	124.1708791	101.3354929	76.91656818
3D1 x 3D7	66.1	129.6917941	104.0463241	73.37228949	94.90311463	78.34021628	64.29825394	113.3980429	83.80335357	67.29928333
3D1 x 3D7	67.1	134.8926073	111.6200654	87.11802254	112.5281602	82.475125	59.93570571	137.7336072	85.81034226	63.99475824
3D1 x 3D7	68.1	124.1402381	92.11848571	70.13685357	82.35368809	72.62845714	63.716	118.4946875	91.217375	75.532125
3D1 x 3D7	68.2	144.06475	128.6390417	89.34975	91.018875	48.432375	39.62333333	83.05716667	56.93666667	43.477
3D1 x 3D7	69.1	NA	120.5982618	57.93401032	NA	76.29766893	59.30156048	NA	94.96980357	67.89861753
3D1 x 3D7	69.2	145.4181667	126.857975	70.13020357	91.71168841	58.1824377	46.55726159	87.02538254	60.70047619	51.30366667
3D1 x 3D7	7.1	132.5015709	91.46150114	76.02591778	96.82423341	87.68825182	75.63258667	127.2132401	106.1091593	82.56958333
3D1 x 3D7	70.1	138.9225604	116.7144049	84.30784231	113.9619414	76.95461298	55.08614556	113.8873173	78.59654936	54.82436239

3D1 x 3D7	71.2	119.4642417	74.62974097	81.52620833	79.34175128	69.86559573	63.92911905	92.60511042	72.98635417	64.59934375
3D1 x 3D7	72.1	135.1607585	114.8528589	90.19066742	118.8364471	106.132657	85.20056581	131.5317916	108.2671471	86.20108167
3D1 x 3D7	72.2	128.0205253	101.0333415	78.08121603	118.2341122	80.19439233	65.79632216	114.8081555	77.62225144	60.66963095
3D1 x 3D7	73.1	129.7474636	101.8440771	68.32869333	96.87696081	71.21999157	59.56131524	104.5816117	69.05347936	58.26187524
3D1 x 3D7	74.1	134.6715337	121.0559872	101.7737357	111.16535	76.40378692	64.89848382	114.5054034	80.10963308	75.49383833
3D1 x 3D7	75.1	130.3196065	104.7133066	81.85850476	109.3800674	94.57603175	81.46999905	112.1699701	94.66611691	77.97759524
3D1 x 3D7	75.2	129.1837541	85.17277272	77.54531758	90.40055411	77.68543802	66.83718475	98.68646601	76.41030705	65.0068375
3D1 x 3D7	76.1	133.5391	119.0110048	100.08689	108.7695333	86.52351619	73.31793333	125.5714333	84.69796	73.69570667
3D1 x 3D7	76.2	131.9574773	100.369903	79.43331976	131.2627192	97.31352948	76.97154722	138.3672406	100.5760292	79.03825615
3D1 x 3D7	77.1	112.2192307	77.31251155	83.31881957	82.85405324	79.43143167	63.31244961	92.77239034	74.17171094	62.79830923
3D1 x 3D7	78.1	116.7932006	87.36882299	61.67377469	83.61560857	68.0388633	50.16998321	104.2035277	76.88833658	60.39997778
3D1 x 3D7	79.1	133.17	112.7085	72.31582222	104.7686333	74.36303333	60.6129	111.141625	84.8306875	63.855875
3D1 x 3D7	8.1	121.8492468	77.59015334	78.16812475	85.07275989	64.00210096	53.89652172	85.06553007	68.07385397	59.06951238
3D1 x 3D7	8.2	139.2425217	129.8859946	93.23705232	104.8151985	71.12934389	59.12174048	95.33764016	75.46994696	59.01196341
3D1 x 3D7	80.1	116.1126154	65.84912121	57.30694944	96.54146415	66.83216952	58.0993	123.5218227	87.43271222	64.62689278
3D1 x 3D7	80.2	115.7311444	86.55267833	73.24845952	95.45435464	91.27937045	78.71675	94.78494762	74.23724643	75.01395
3D1 x 3D7	82.1	127.1834967	82.67500658	50.17296659	83.42189909	54.64328762	45.9118	97.6490682	61.74469299	51.14766667
3D1 x 3D7	82.2	130.8845429	78.89452788	61.55035276	117.0183546	93.47223095	83.01379028	126.496826	98.93255507	76.715925
3D1 x 3D7	83.1	116.5425241	76.03682893	69.55818864	84.18277488	63.14270772	52.25750877	121.3914298	87.54078062	67.9946998
3D1 x 3D7	83.2	NA	NA	NA	71.722	51.673	41.856	NA	NA	NA
3D1 x 3D7	84.1	122.5390833	113.2460833	114.0093125	138.799919	118.12185	98.2186	142.0643533	126.87885	108.80015
3D1 x 3D7	84.2	139.9358867	129.704392	114.2944071	91.39070024	65.82530643	57.37835773	80.60586943	63.94896263	56.81418595
3D1 x 3D7	86.2	130.90975	83.08857917	66.333125	86.9205	59.06973667	50.79038	NA	NA	NA
3D1 x 3D7	87.1	119.4662	72.2639092	53.68916104	69.94410834	55.68082692	49.76068006	82.79014444	54.46703643	49.11392333
3D1 x 3D7	88.1	128.5371605	121.11342	97.80528911	84.91981648	69.77668316	61.44299154	86.95841158	68.23619879	60.30950404

3D1 x 3D7	89.1	130.0942326	124.4101288	113.1728922	117.874396	82.51538977	70.30835556	137.4089416	109.1990627	72.33131667
3D1 x 3D7	89.2	123.2666667	132.5490833	123.0581667	117.9218691	91.89802858	69.80549524	126.73456	101.3191	83.42284333
3D1 x 3D7	9.1	127.5061178	96.33129728	74.34117143	99.29095116	74.66140208	59.44418651	107.4584133	81.72285143	60.24218333
3D1 x 3D7	90.1	124.1059409	78.02744416	59.96323818	85.30534091	60.62567111	54.19336667	103.7316753	75.44853333	63.92218
3D1 x 3D7	91.2	131.7397859	114.3827143	102.2866889	114.106	97.85499893	83.90320833	127.8512286	94.42201667	77.04988333
3D1 x 3D7	92.1	134.1069473	122.2893976	82.2742621	103.2878176	80.5254809	56.65332667	93.93645363	73.42889835	56.39866539
3D1 x 3D7	93.1	126.0612453	106.4070198	73.60460364	112.6845277	98.69777475	83.41178572	126.2278936	106.2318507	94.98958333
3D1 x 3D7	93.2	140.4458789	118.9459466	87.0450144	111.8445034	79.66245576	64.34473691	106.6398897	89.55186246	78.64313333
3D1 x 3D7	94.1	116.3552507	68.77585889	58.79182093	85.2101035	71.15724824	54.09736947	106.9119375	76.44265947	65.98349275
3D1 x 3D7	94.2	134.3618664	123.5504928	100.7884533	128.7292414	108.15678	89.42358889	138.1719361	109.5675905	91.57035
3D1 x 3D7	95.1	134.8024131	129.9026638	122.6036508	135.5167899	125.2594775	110.4964441	123.9543039	111.56085	93.16291818
3D1 x 3D7	95.2	141.1246189	124.4959078	100.0641096	94.06768127	59.70294939	53.56043918	82.77282723	58.54581293	53.7420254
3D1 x 3D7	96.1	121.5177962	63.45879472	49.96683043	68.57126972	55.61269659	49.64093087	95.12954237	64.49154222	56.6136747
3D1 x 3D7	96.2	124.296238	86.67959407	70.29780167	80.44291685	61.8958596	54.94391905	84.71772647	59.71470746	53.61638809
3D1 x 3D7	97.1	138.1796607	100.3891116	68.43869268	119.1502393	78.36920063	58.97555714	136.962099	92.29469121	68.41900897
3D1 x 3D7	97.2	128.5034806	102.2765582	64.6266859	115.3046029	91.96235268	77.66428572	104.7856744	76.8605727	65.12964667
3D1 x 3D7	98.1	141.210134	114.906873	92.998	89.02942909	57.17405951	44.84483266	83.10620476	55.00385238	47.99129667
3D1 x 3D7	99.1	NA	110.6977885	84.6508166	NA	74.19974585	54.27046907	NA	93.19490873	70.52173047
1A5 x 1E4	A1.1	132.5790795	63.51353985	56.04975215	117.6858372	99.76006338	87.54909508	106.8396329	65.5010146	73.15050697
1A5 x 1E4	A10.1	142.9830019	134.5393723	100.0858318	106.6643368	63.99398022	51.21225476	108.0828536	86.66337922	93.15221478
1A5 x 1E4	A11.1	127.6758367	90.22278341	98.99613553	100.1444955	73.21620833	59.40346667	128.8109878	86.88855994	72.76998893
1A5 x 1E4	A11.2	133.1738894	86.42442276	87.7914685	76.62979025	60.10945913	52.29096156	117.7366153	97.33199177	85.27899593
1A5 x 1E4	A12.2	144.8502917	137.824848	118.5203617	135.7655886	92.82833099	86.64696114	146.766013	143.6718542	127.8917814
1A5 x 1E4	A13.1	140.6154299	114.7669524	113.7456037	92.6217908	99.09597842	70.50643029	109.3177058	101.9236541	77.0169961
1A5 x 1E4	A13.2	134.5581563	121.4889278	116.2314639	74.44483159	65.60741548	72.87964286	122.1446957	89.13732001	75.79855

1A5 x 1E4	A14.1	138.6986332	133.9625169	124.2767459	135.2878282	91.4913371	68.21450981	66.29180588	108.4765724	101.2765516
1A5 x 1E4	A14.2	138.37409	116.2342862	109.2732059	94.84636191	64.25854329	47.00154242	144.1510614	116.6752539	98.92374286
1A5 x 1E4	A16.1	135.5075833	129.0794425	129.0404991	100.9680229	79.89036855	56.38064881	147.2335933	134.7606533	117.8988333
1A5 x 1E4	A16.2	138.1434167	91.82944448	85.69151786	125.6431238	98.42976667	74.06099714	154.02225	144.7092222	121.06525
1A5 x 1E4	A17.1	133.1909728	128.9427935	110.7885259	83.72118406	73.47524402	44.71963413	132.7753152	115.5283863	108.2860229
1A5 x 1E4	A18.1	127.3506712	115.7412573	120.2684069	74.74213354	72.7874175	46.99657182	104.4959783	73.88110567	66.34136313
1A5 x 1E4	A18.2	129.1399439	74.41897701	82.52629193	81.07931155	54.47141143	54.61966667	117.473407	68.28897142	69.31737708
1A5 x 1E4	A19.1	137.3291517	129.1016152	122.1713675	114.8750199	65.84285945	55.52193956	119.6722083	100.0062796	92.99184349
1A5 x 1E4	A2.2	133.7945333	96.60595476	64.67851905	90.66408667	65.67700333	49.69129333	129.5042	111.9315	87.016
1A5 x 1E4	A21.1	131.0761357	87.36033167	99.8336711	77.3623032	77.1727873	59.84112778	82.71865221	82.28084667	67.12995879
1A5 x 1E4	A21.2	139.89555	121.0532072	118.8668214	85.39833333	85.99943333	55.02743333	97.68191667	104.1765834	68.83288889
1A5 x 1E4	A22.1	124.4123333	104.7658333	82.51233333	111.5723611	67.17516667	53.61016667	133.24	118.28	107.0805
1A5 x 1E4	A22.2	143.0424025	133.3678388	118.4917771	113.454642	69.51728251	53.39574066	142.5509417	99.49903252	87.42445675
1A5 x 1E4	A23.1	138.7694893	120.1341026	103.3632918	103.015939	77.35675812	66.11889222	125.6242972	91.72923656	89.07978099
1A5 x 1E4	A23.2	137.7155392	90.11965742	67.08916712	104.5964524	100.1020111	100.7403542	148.0114006	151.3291591	149.3876882
1A5 x 1E4	A24.1	139.1906869	82.51778271	72.20053347	121.4889598	105.8941424	97.72690001	125.1300802	108.1280397	99.58680903
1A5 x 1E4	A24.2	145.1315	111.6883334	97.05383332	116.8624583	102.83025	73.87679167	145.5495	146.94375	133.596375
1A5 x 1E4	A25.1	145.9211531	122.0976939	95.87569646	101.2892737	89.65724907	78.92249136	130.3232446	103.489721	110.4403998
1A5 x 1E4	A25.2	133.3692643	99.25903834	105.4285083	90.33938416	76.71826667	66.0684	143.825	117.5645933	99.17278
1A5 x 1E4	A26.1	135.0252967	94.7338896	104.8950762	78.05405359	73.46566768	55.37960905	129.09845	86.65133091	107.3535159
1A5 x 1E4	A26.2	142.7656382	107.981773	98.26837192	109.4252095	79.87659524	62.435	158.6042611	142.8583504	125.19635
1A5 x 1E4	A28.1	127.4782045	109.6338012	109.1889528	66.3117875	57.32428614	45.95409984	108.8615133	53.98129444	68.94106667
1A5 x 1E4	A29.1	127.9265542	113.7188785	103.2456775	78.40115632	63.75651326	47.65048667	106.6025515	96.70824022	65.70786497
1A5 x 1E4	A3.1	132.6597167	97.88726001	80.17256667	101.0009467	64.45957333	51.65788	128.2988981	56.36165333	84.67344524
1A5 x 1E4	A3.2	150.6098832	140.2116994	118.1365329	138.9273489	101.1325535	71.10404692	138.5841527	131.8159045	93.44056161

1A5 x 1E4	A30.1	151.6194667	135.9128616	117.2946396	87.00642338	79.69179206	50.802785	135.2840417	120.7787544	77.97255556
1A5 x 1E4	A30.2	137.0505126	115.8981977	108.0665547	98.05828578	64.91121667	53.51093333	135.9968012	86.10851366	69.28510392
1A5 x 1E4	A31.1	143.6752758	106.7417029	96.71028477	89.94646667	75.48881191	59.91456667	160.9419334	134.2334167	103.78175
1A5 x 1E4	A32.1	142.0272354	131.6329133	117.8287005	90.59721154	83.59696667	67.82681	127.2505748	126.5577364	100.3617333
1A5 x 1E4	A33.1	145.7235333	113.524955	90.12016667	109.6034647	99.29941506	86.96640095	134.4067333	127.51079	102.10654
1A5 x 1E4	A33.2	138.4803667	116.583269	104.6886898	101.2511357	67.15950955	59.63854788	119.7407047	95.43281282	81.59951056
1A5 x 1E4	A35.2	130.7345987	112.9385192	112.8132933	90.26866116	86.43454351	68.38337394	146.7870439	134.38783	109.7301907
1A5 x 1E4	A36.1	143.1481689	140.9159657	126.3872468	132.3760106	79.05037815	56.64825178	107.2926933	105.2822248	88.01124857
1A5 x 1E4	A37.1	132.4328367	106.1139162	101.5665065	88.22397422	83.11985341	46.52782727	109.0477078	111.4371116	74.36529524
1A5 x 1E4	A38.1	151.6616333	143.3072286	129.0137833	116.8726844	85.70714539	47.74022143	97.70244	111.5696067	75.79465417
1A5 x 1E4	A39.1	142.3779717	100.6595933	83.25163722	126.7278746	127.627911	105.0282276	165.6963009	155.8281622	131.7053457
1A5 x 1E4	A39.2	138.3859	119.5689	93.11612667	101.123225	74.46750833	60.41398333	108.95705	116.69864	105.4121667
1A5 x 1E4	A4.1	110.5555249	69.87343665	69.66528988	96.31039826	121.9455877	119.0412329	147.9205894	133.2705481	140.1868579
1A5 x 1E4	A40.1	147.2704205	141.3243064	122.5631598	130.0601929	106.3686648	61.93760833	144.4268333	129.10238	85.54543333
1A5 x 1E4	A40.2	129.1255778	84.85927951	67.89256682	125.1018135	123.6736914	109.3732343	149.5326674	138.7090651	109.08101
1A5 x 1E4	A41.1	139.7002614	117.7233675	104.15073	74.18158386	64.11071678	51.12194667	124.5683955	109.8487445	78.1695987
1A5 x 1E4	A42.1	NA	NA	NA	NA	NA	NA	NA	NA	NA
1A5 x 1E4	A42.2	132.6881369	87.73847854	69.81695714	90.6378026	74.69791511	65.12042619	140.2226088	137.1479287	124.123999
1A5 x 1E4	A43.1	140.265125	137.0129792	124.8567917	135.7705	124.1853333	77.39696667	153.792	129.3958	101.4582
1A5 x 1E4	A43.2	143.5481167	131.0605181	122.2229895	122.0099324	99.95770666	80.58088524	122.5364829	87.29904273	91.42494152
1A5 x 1E4	A44.1	148.6290934	146.0935148	122.0924748	101.6941934	83.35170286	75.08639524	130.1571198	109.3873122	95.5128357
1A5 x 1E4	A44.2	134.4996991	87.59516346	71.93589033	114.5356663	119.348498	115.9900301	144.4155095	142.3938027	138.7617523
1A5 x 1E4	A45.1	144.9642917	113.28578	82.64723333	125.8014576	112.8108019	95.06084667	162.3703786	163.50136	148.1152
1A5 x 1E4	A45.2	141.3185667	131.8298144	117.4909179	96.25123021	68.70987293	57.23838738	146.4312421	132.4308826	108.2757133
1A5 x 1E4	A46.1	135.5093449	100.4376277	100.0153823	80.11675762	75.78519049	49.04425626	137.8661671	125.9194243	98.19198129

1A5 x 1E4	A46.2	137.7198833	109.3041105	93.9163638	86.72292121	64.60196162	58.24560952	126.7294738	105.1249029	87.81838667
1A5 x 1E4	A47.1	137.7872733	116.3291876	100.0947738	104.8185105	76.61460778	57.92884068	142.5591921	129.3636956	114.5495083
1A5 x 1E4	A47.2	142.0822783	131.5754548	114.0051174	106.2476641	88.69771389	65.16922857	137.117671	136.4285155	122.9240967
1A5 x 1E4	A48.1	131.8528889	129.8186	111.2033833	82.30006667	72.7424	47.5742	117.44325	109.1277867	70.44095
1A5 x 1E4	A49.1	144.0360333	105.3717533	98.88207936	119.5317333	92.00655	70.26708333	139.8451	128.6395667	102.08793
1A5 x 1E4	A5.1	127.6277718	73.14858054	89.52311273	101.7392523	72.24244167	60.50827857	139.5365542	100.8558421	83.23721442
1A5 x 1E4	A50.2	138.3679194	117.7755948	108.19339	105.3818867	81.13446667	69.46616667	148.6895625	136.74825	130.0129667
1A5 x 1E4	A51.2	134.9260333	132.9756857	124.3790071	128.274603	79.17764286	44.30346667	92.30891667	100.493725	60.62627333
1A5 x 1E4	A53.2	137.8274533	127.4843683	124.565385	101.324893	63.30901714	50.21866667	115.1636467	101.11245	86.37755
1A5 x 1E4	A54.1	132.415	122.4978333	107.9678333	125.1972084	88.47916665	71.74841667	124.8423333	112.5528333	93.0335
1A5 x 1E4	A54.2	122.9869589	117.3784516	111.5344056	80.88426667	75.64437619	60.18197143	129.93235	119.0513889	81.52066667
1A5 x 1E4	A55.1	142.418575	90.408695	88.52519278	89.47980556	83.7371	64.88553333	114.7867333	98.26295	76.46991667
1A5 x 1E4	A55.2	135.1555	107.0047	111.5239	84.33645833	82.42841667	44.81225	106.942625	56.35675	83.65645833
1A5 x 1E4	A57.1	131.9742191	85.32011667	58.15598333	103.9768238	80.77416429	66.37371	117.5860333	117.7001	90.777875
1A5 x 1E4	A59.1	127.96256	114.0855933	96.89270665	79.91454167	81.53592857	61.7538	139.5225917	119.3076167	79.76961667
1A5 x 1E4	A59.2	132.5115	144.2213334	125.201	121.147875	100.214375	68.043375	163.802	142.084	120.7175
1A5 x 1E4	A6.1	132.7965971	130.4159867	119.3923019	74.84744893	69.4330004	60.62271333	126.506097	141.3996621	143.7859211
1A5 x 1E4	A60.1	145.78355	119.3120317	99.76969842	107.4881528	87.20307333	72.61015	153.5468724	143.1628584	107.382445
1A5 x 1E4	A60.2	131.0936047	84.02562233	77.72023658	111.9049391	99.26510683	84.26126	135.8522742	126.2155284	110.2238298
1A5 x 1E4	A62.1	NA	NA	NA	NA	NA	NA	NA	NA	NA
1A5 x 1E4	A62.2	139.6797801	124.14506	115.8640572	80.90529689	83.80021234	59.68796889	128.8021144	128.9199584	94.39715491
1A5 x 1E4	A63.1	128.9855294	114.1023754	110.6916741	96.86473332	89.50750335	60.19971524	126.8765	101.96475	82.215625
1A5 x 1E4	A63.2	143.1964801	131.1013224	121.0491986	126.481857	95.21814883	64.3777141	120.996454	107.6633833	86.29578408
1A5 x 1E4	A64.1	129.211285	92.07845761	90.28865065	106.2831761	76.93611966	69.79676278	131.5425586	106.0642833	83.11237
1A5 x 1E4	A66.1	133.4675407	116.9485872	110.0947785	95.81794348	62.53737672	53.71243071	129.2341556	108.2874429	85.72849524

1A5 x 1E4	A66.2	134.7839333	90.15729001	66.9965	128.4970965	95.47460714	73.06184643	148.285	143.60954	130.4062
1A5 x 1E4	A8.1	125.4681583	120.930332	104.5378498	89.98524407	56.86299865	45.65538413	129.5496293	112.0432488	89.11155095
1A5 x 1E4	A9.1	119.4421308	78.12743333	78.86890385	62.04392204	57.30012535	71.60754842	125.0787143	117.6950194	89.0731049
1A5 x 1E4	A9.2	130.35401	75.25609841	67.92827957	119.3472766	94.03320782	71.80053214	157.7818671	144.7443084	123.0305747
1A5 x 1E4	B1.1	128.5080333	109.614325	100.0280667	99.58846	86.51501333	61.303	109.0359375	105.55452	62.229
1A5 x 1E4	B1.3	128.0616947	96.35146621	94.00783356	81.64581055	70.25627641	63.28457603	144.9114646	128.7444737	93.80936044
1A5 x 1E4	B10.1	129.7614788	111.3268313	100.7267792	76.29773133	60.95323164	44.08152987	117.9937711	86.2285683	70.93620545
1A5 x 1E4	B10.2	135.2359228	106.7759484	102.1226727	101.368487	80.73537521	66.79540805	125.1993446	103.3987878	98.62729286
1A5 x 1E4	B11.1	141.7384965	115.6457832	98.3688306	82.5369656	67.69005643	59.29933333	119.9751621	103.9971897	88.38618017
1A5 x 1E4	B12.1	141.5255035	132.42609	121.9116829	139.7456425	95.81670814	62.2581548	108.3949184	111.6018044	85.49447619
1A5 x 1E4	B13.2	130.0131	120.1087667	110.36835	84.93360571	67.01679333	57.68346667	118.7979	122.4579	89.53116667
1A5 x 1E4	B14.1	125.1453322	86.09613634	97.7787486	115.6244924	105.1143541	89.08824666	114.9433221	128.2443436	118.5328107
1A5 x 1E4	B15.1	NA	NA	NA	NA	NA	NA	NA	NA	NA
1A5 x 1E4	B16.1	129.7618295	134.448456	103.3734042	65.03256726	60.93242853	52.00648409	73.53937857	94.49350167	56.07600714
1A5 x 1E4	B16.2	133.5910267	74.03923035	69.45972476	122.0592316	114.44024	106.36536	151.0714667	147.8694422	143.1907333
1A5 x 1E4	B17.1	134.194713	86.2012914	78.49137286	78.77827026	66.37804532	62.76420778	134.2188009	110.7325069	92.63421056
1A5 x 1E4	B17.2	137.9435833	101.0213756	91.02327143	98.70439334	67.32458667	54.1888	144.2077778	130.3613533	108.3912667
1A5 x 1E4	B18.1	144.6246333	141.1399	97.1924	134.7702	103.47165	100.9975833	158.1511	159.7602222	143.9955
1A5 x 1E4	B18.2	134.8159594	110.8981636	108.906787	101.8989477	81.14125481	63.77520404	126.5698357	135.5055404	105.9267589
1A5 x 1E4	B19.1	136.1340571	125.9591277	114.6866234	103.1804196	80.34716845	50.12216901	130.2656447	106.6616476	88.86485843
1A5 x 1E4	B19.2	141.0155083	113.1531694	108.5638875	107.0559149	95.47190223	85.45019024	146.2291833	141.7172598	139.7296997
1A5 x 1E4	B20.1	143.55828	113.5642566	67.96673286	118.3602919	103.6283588	84.93420309	145.6275634	135.632825	123.7297079
1A5 x 1E4	B20.2	139.1774103	95.37115429	78.74335848	119.1122956	84.16932738	75.4789	148.5363373	138.4223522	126.9171271
1A5 x 1E4	B21.1	161.281	NA	NA	112.739	96.318	94.502	147.4275	152.4523333	142.8315
1A5 x 1E4	B22.1	132.7680006	83.11662486	75.07489622	112.2363388	117.160774	105.0957764	125.4875486	122.7314557	113.0768567

1A5 x 1E4	B22.2	139.1952627	123.481887	115.0993309	111.0737396	91.70052183	81.72054181	148.61088	144.0961076	138.16405
1A5 x 1E4	B23.1	137.1044352	130.9171317	117.246215	100.7991559	89.16327714	76.867305	135.9359895	137.8761842	140.965961
1A5 x 1E4	B24.1	132.4782599	130.605809	117.1873413	85.14666827	68.91424936	49.44348434	110.4923662	91.05316673	80.97807053
1A5 x 1E4	B24.2	137.2404799	134.8213389	124.7277361	95.47497071	99.256425	61.31818333	117.2651516	87.30823556	96.51404857
1A5 x 1E4	B25.1	130.7955455	90.37751187	92.60034262	82.10492569	61.00173175	54.13857283	110.164525	65.89980064	71.12189611
1A5 x 1E4	B26.1	132.1237159	122.4718224	119.1080457	98.58230511	86.70305286	64.60113429	151.1460755	141.3617091	107.3603071
1A5 x 1E4	B27.1	138.085921	102.9919718	100.6205989	108.3601161	85.31249987	77.30130892	142.9945053	116.1073948	76.95035833
1A5 x 1E4	B28.1	141.1243435	116.7684836	91.97350862	100.0655125	88.89748809	80.6754	144.090426	137.5373225	130.2682758
1A5 x 1E4	B28.2	143.23725	89.97300333	74.10408	95.73631429	65.6586	60.23148	143.35475	121.8797333	119.3861
1A5 x 1E4	B29.1	138.4255	131.5315	115.6945	109.6045	75.2175	58.4075	140.9045	133.4095	101.734
1A5 x 1E4	B3.1	132.6958875	112.90655	98.81697	89.40645167	64.63610667	50.94798667	115.2772333	125.364625	76.91194
1A5 x 1E4	B30.1	137.9884309	96.2201925	77.0611681	100.7175396	105.8821973	84.97631809	132.713852	134.526945	114.8883433
1A5 x 1E4	B31.1	138.1798667	117.766325	111.62	102.32775	91.720125	60.218	127.274	123.789	103.3935
1A5 x 1E4	B31.2	131.6718945	120.8216799	113.6070354	113.2249022	104.7102111	89.53248	157.8152	128.3234738	108.36704
1A5 x 1E4	B32.1	138.7214013	75.69165808	71.08225076	94.63540622	67.40480401	55.67591111	122.7344765	107.4379494	88.13497974
1A5 x 1E4	B32.2	135.5626167	134.5339833	117.1776733	95.25105833	70.01573333	59.57763	151.197	153.7325	136.5776667
1A5 x 1E4	B33.1	145.205288	137.2626367	122.3737256	96.48070661	72.52905247	50.95451154	137.75206	126.8075433	93.34935953
1A5 x 1E4	B33.2	129.4767367	84.21919384	81.64606525	118.055901	67.63995833	53.07055556	133.6182843	65.72365585	61.25066667
1A5 x 1E4	B34.2	129.5802086	131.3973956	116.418814	106.0849396	66.90673429	55.45728333	86.79510417	103.5976976	73.20078333
1A5 x 1E4	B35.1	138.1842385	74.32456511	69.73116667	111.6035597	75.78205455	62.16727667	141.8544567	143.3892196	122.3254108
1A5 x 1E4	B35.2	135.4471357	128.4571441	115.5542085	69.391025	64.1969	60.39947714	127.42976	112.3707067	91.62565238
1A5 x 1E4	B37.1	130.8659178	99.83010384	68.32395179	87.25136667	65.12884889	57.67602857	147.279525	119.3171667	117.7047958
1A5 x 1E4	B37.3	137.8295732	126.0171412	97.15215625	109.0527674	112.7640119	94.7834375	155.66806	154.8043276	145.4118033
1A5 x 1E4	B38.1	139.4165383	90.644215	95.60740714	113.5009139	79.06933333	67.25332667	144.1964667	77.4182	79.48383333
1A5 x 1E4	B39.1	130.5622126	112.0812618	105.8642321	111.6439901	97.40146021	95.1614724	136.5257681	141.2983541	136.4894674

1A5 x 1E4	B39.2	139.6081707	133.3632509	112.6192921	124.8674568	104.4782996	98.44952666	145.1739729	140.84618	132.1983088
1A5 x 1E4	B4.1	143.7639931	116.029286	94.53149554	103.4939681	91.75732202	73.96416714	144.9810334	132.5640737	111.5671869
1A5 x 1E4	B4.2	131.2955	117.5425	105.1417333	96.475405	91.82979166	66.51614167	129.89125	101.298625	101.735375
1A5 x 1E4	B40.2	140.842375	111.5995	77.0483	82.96198333	70.7223	57.70006667	156.3395	155.563	131.889
1A5 x 1E4	B41.1	134.516229	86.44480663	NA	112.5749542	92.64829143	80.1234005	140.2502783	127.1528203	120.3244413
1A5 x 1E4	B42.1	138.3251411	108.6528	82.67801667	122.85721	129.11522	126.6996667	164.1462029	163.6769167	135.62272
1A5 x 1E4	B42.2	124.0168373	82.61479515	78.6627503	72.72879522	68.23400212	66.48560664	128.7297522	109.9260841	97.19888542
1A5 x 1E4	B42.3	139.0953334	108.9238333	69.73733333	114.30975	93.47453	88.59615	157.1938333	155.2893333	126.9715
1A5 x 1E4	B43.1	133.9527683	109.2556533	104.7310197	82.57884483	65.07837936	50.98952084	150.2178182	122.6348206	114.1586252
1A5 x 1E4	B44.1	138.6275192	94.18028024	NA	103.9329205	94.62070348	80.38115673	140.5590853	113.6569681	102.418449
1A5 x 1E4	B45.1	141.4474554	134.1595908	123.0438228	132.423774	82.70684485	56.22325095	138.007628	122.306676	89.36502035
1A5 x 1E4	B45.2	145.98095	99.9948	73.1593	101.7535133	67.3481	58.01701667	151.1516667	118.6985	105.15125
1A5 x 1E4	B46.1	137.536422	118.4371207	95.99732728	96.34851139	67.18737949	61.8608419	134.4066159	83.1526283	82.89940476
1A5 x 1E4	B46.2	129.8602201	126.1781683	113.1956278	103.2035306	73.75895549	55.15816306	123.8968548	122.8148559	109.6998867
1A5 x 1E4	B48.1	139.5879353	106.4159279	100.5809852	84.82881424	72.57909064	51.74898064	141.7865768	138.7888194	110.5240635
1A5 x 1E4	B48.2	125.8614879	99.08073353	85.89214108	113.1137093	91.55285228	75.291	116.7033886	95.15253005	96.09366195
1A5 x 1E4	B49.1	144.1564905	128.2497613	107.9341162	102.2783771	64.61570659	55.92975	151.9002381	125.6348067	109.9867951
1A5 x 1E4	B50.1	123.5875	117.2452467	109.7721067	87.98448667	65.15776666	46.90643333	124.8872833	80.4757	63.03908667
1A5 x 1E4	B50.2	125.7868333	115.5642397	112.0851997	82.99202222	81.74185985	60.19221778	125.9008964	106.0321041	84.75310131
1A5 x 1E4	B51.1	133.1131879	105.2627535	101.976049	104.1805617	68.71855487	62.54118571	133.9954808	137.3796131	121.9296723
1A5 x 1E4	B7.1	141.9079791	137.258303	126.6419826	106.7360542	105.71044	65.07901571	133.7761673	123.5348667	90.19001334
1A5 x 1E4	B9.1	138.2268579	131.4043573	116.7382508	106.0870073	76.30081809	61.07464762	115.75994	115.2124667	67.37472
1A5 x 1E4	B9.2	138.9647485	106.3165457	76.89690721	75.47834298	57.71783262	44.56008222	134.5449572	97.43468388	79.94560434
1A5 x 1E4	C1.1	127.5245389	73.64792524	59.50207372	102.8008739	71.10956455	54.34925	142.7621974	118.399576	108.3362155
1A5 x 1E4	C1.2	137.13525	124.0585	119.613	102.7106667	88.32183333	62.7545	125.93325	117.778	97.4685

1A5 x 1E4	C10.1	135.7489835	102.8437168	104.2433061	123.0122465	74.51611475	41.02894311	155.2625376	106.4701828	83.57872073
1A5 x 1E4	C11.1	145.2241476	119.57814	96.4371875	109.1291333	104.2816667	97.54918	166.7965556	163.2025167	155.0664444
1A5 x 1E4	C12.1	141.5438286	116.2443784	111.7239715	120.0890667	90.20245	60.10815889	135.2768423	105.329946	94.72405238
1A5 x 1E4	C12.2	NA	NA	NA	NA	NA	NA	NA	NA	NA
1A5 x 1E4	C13.1	127.5553165	89.29952628	94.7013492	80.26816015	68.84060641	55.70192794	159.1511333	115.75638	107.5439222
1A5 x 1E4	C14.1	140.0680891	94.98631877	95.71696463	109.4511264	80.23738481	62.09112641	147.3266742	114.4920824	115.2885183
1A5 x 1E4	C16.1	138.7354529	84.7587192	72.29394432	95.850793	62.87359295	53.86069818	132.718665	101.5904968	86.95619487
1A5 x 1E4	C16.2	123.876625	101.60025	105.815	103.3781	70.4133	74.5273	128.853	98.4185	96.89175
1A5 x 1E4	C16.3	138.3346675	96.76493328	93.44512333	90.91069442	66.09414475	62.871395	126.9855567	74.22889206	88.79056627
1A5 x 1E4	C17.1	142.7215644	132.9026265	120.3982217	91.45934417	77.86443056	59.03718127	152.5392766	139.4103952	131.6338833
1A5 x 1E4	C19.1	146.3476967	127.4464732	109.5807119	88.94753714	75.80795821	58.79238827	144.7334518	139.387173	133.4559239
1A5 x 1E4	C2.1	131.7234601	95.27911065	101.0985851	105.8110037	71.64244635	63.26905714	153.0798486	115.353605	93.98769714
1A5 x 1E4	C2.2	130.1316827	91.51264895	73.99062026	114.6030851	89.83661606	62.46781857	121.0379009	99.52963007	81.57816282
1A5 x 1E4	C20.1	129.6544431	97.80093213	93.48593444	81.9401527	54.928691	41.28602424	NA	NA	98.216
1A5 x 1E4	C20.2	137.724285	107.2917967	99.59888166	102.029189	79.40390357	60.38620857	154.0035341	125.6119964	102.2982833
1A5 x 1E4	C21.1	119.3093315	81.55554787	80.78540189	89.29023655	49.10955738	43.15654314	137.2280196	108.9856075	73.88662927
1A5 x 1E4	C21.2	126.0507262	108.9028131	108.8525581	86.17175569	70.07317212	54.06693333	156.7422	135.6315667	114.9175625
1A5 x 1E4	C22.1	142.8353514	117.1007588	107.536309	78.11042204	69.69702157	60.36667167	156.3499396	133.8794199	142.9835
1A5 x 1E4	C22.2	144.4536667	130.71475	87.423375	135.0869333	117.45825	102.525875	148.87225	156.6655	137.7085
1A5 x 1E4	C23.1	136.2338078	117.4659515	103.4335346	91.27405506	53.80671236	52.37190333	153.576576	114.5450588	101.9189507
1A5 x 1E4	C23.2	128.4643228	95.25976012	102.7280243	126.1464875	90.05688529	80.27681131	124.903625	110.5636667	78.76466667
1A5 x 1E4	C24.1	134.0582105	69.64222597	51.00982212	100.8950475	76.25730486	66.52643405	180.047	106.9842071	100.3839756
1A5 x 1E4	C24.2	132.1045	105.756875	73.69875	137.6296458	128.115375	115.4746042	159.4175	162.1505	163.062
1A5 x 1E4	C25.1	136.0160391	70.41153283	60.80436321	79.59059306	57.87156142	48.97869889	115.7216251	85.76035522	70.39166526
1A5 x 1E4	C26.1	133.9156975	118.9206149	82.16869582	65.68936975	67.49848356	77.6047823	163.5146433	150.9274457	144.0472689

1A5 x 1E4	C27.1	132.8423309	80.06154592	72.90514286	94.55936737	72.61621597	59.69446429	168.8231659	151.2485923	131.9449827
1A5 x 1E4	C27.2	136.3252721	131.1158003	118.3307716	83.82705264	64.8126165	53.34550718	139.4995522	120.2966596	92.59595913
1A5 x 1E4	C28.1	130.479069	116.6854858	113.4424294	76.85163926	65.87534538	51.76089143	158.69825	91.24401118	84.80298818
1A5 x 1E4	C28.2	130.1669992	73.85098107	64.27348104	117.9577885	110.2962957	96.22513	130.6200046	101.4368999	89.23253304
1A5 x 1E4	C29.2	139.6823971	115.5535913	116.9444255	116.1837258	101.136391	69.34035233	138.7755714	71.40249167	94.63641666
1A5 x 1E4	C3.1	140.3301186	103.9566103	85.83152333	100.8894152	79.92450564	71.98861778	160.2839909	152.0471128	134.8799714
1A5 x 1E4	C3.2	134.5841893	83.11963697	64.99290286	134.9947094	121.7876562	119.1931122	140.8284822	132.8992663	140.1654556
1A5 x 1E4	C30.1	151.5715786	136.1510761	125.9742967	130.5114987	71.17414216	64.29031753	152.0555	104.8751432	87.51754444
1A5 x 1E4	C30.2	134.1023547	101.4425202	104.2191254	103.6012222	77.6689329	69.2841875	105.0896244	108.153301	100.4925355
1A5 x 1E4	C31.1	147.838	132.5845	127.7885	104.2463333	63.38866667	52.29133333	NA	NA	NA
1A5 x 1E4	C32.1	132.71918	123.9616664	117.9231526	111.9334998	73.90874889	58.08241587	NA	105.920547	79.74651167
1A5 x 1E4	C32.2	139.7737333	108.1751167	101.287285	90.73287333	83.4193246	69.37380476	155.6486189	146.256621	133.6010238
1A5 x 1E4	C33.1	137.1705296	108.964665	89.44336111	105.4674609	72.76378988	71.56895119	139.1918049	99.99494887	74.45681429
1A5 x 1E4	C33.2	139.369881	102.1718115	87.10281092	120.0569589	113.02144	116.1991933	140.0506445	112.9342417	125.7985167
1A5 x 1E4	C34.1	147.5775536	136.6016341	125.677171	84.93427605	74.52785824	63.50498952	149.1029315	137.8857736	133.915305
1A5 x 1E4	C34.2	130.321625	123.4813333	120.4628667	86.26383333	88.465625	56.985	144.7725556	87.6595	50.91175
1A5 x 1E4	C35.1	142.3662227	128.8896975	109.3473719	113.666039	60.78977044	47.9867019	108.5758444	84.48499945	85.3406602
1A5 x 1E4	C36.1	127.0308333	86.16389583	86.6175625	72.6855	81.30425	96.0484	162.3046667	150.5595833	154.282875
1A5 x 1E4	C38.1	147.0651333	116.3787813	83.46219444	120.3935724	101.3347627	94.37235556	151.9736667	141.5345226	144.3460167
1A5 x 1E4	C38.2	118.9603417	80.41088214	92.51217917	76.26142218	67.45820698	57.08816964	133.4716438	103.8560554	89.45447619
1A5 x 1E4	C4.1	125.6319663	77.58713649	95.82068889	105.4003852	69.62644459	59.70382333	110.3769072	79.09003818	73.12279333
1A5 x 1E4	C4.2	121.7002894	77.88447504	77.51602175	99.36923833	77.58271452	66.40835857	146.0494667	100.08159	98.63253999
1A5 x 1E4	C41.1	147.7500086	109.7750465	89.49132975	102.2181195	93.64220574	76.24140111	157.3657333	108.3418756	94.90216883
1A5 x 1E4	C41.2	139.0121882	133.2154852	115.915477	120.1366951	91.64463783	74.86228604	143.0756151	145.9642432	128.0395209
1A5 x 1E4	C42.1	144.6483969	98.84638462	69.41314795	106.0900022	93.79166952	92.66941667	139.81129	130.8678591	131.2997667

1A5 x 1E4	C42.2	142.6855625	131.8754042	122.3299678	106.7901857	77.17219881	54.20860571	141.3477667	124.2586	102.3181
1A5 x 1E4	C44.1	141.4718384	106.8179153	103.8437758	86.13929075	75.16571905	53.4784	107.8652784	83.54049013	87.41918385
1A5 x 1E4	C44.2	138.0350445	117.0016017	103.2994714	107.3702259	93.90524666	82.03859048	154.0073542	147.6823111	148.3282667
1A5 x 1E4	C45.1	137.5851776	94.36936982	90.00061948	88.56857445	87.04234333	65.62448286	125.5999722	104.5545349	83.58703481
1A5 x 1E4	C45.2	136.0938333	92.10665909	71.24163691	139.43201	128.9586983	120.74786	156.2096506	158.758505	145.57049
1A5 x 1E4	C48.1	132.4121205	127.8426862	116.1254657	85.69619206	80.82021031	56.40029	100.51829	126.6741792	75.9943
1A5 x 1E4	C48.2	137.5296833	99.63488952	91.5923883	99.78220204	78.31131814	60.73988556	141.469541	144.0531725	121.1839548
1A5 x 1E4	C49.1	147.9555156	130.9669089	114.1252422	NA	NA	NA	155.6216762	146.5110324	143.8070431
1A5 x 1E4	C5.1	134.74533	91.19825855	67.53456722	112.1167485	67.3717248	54.50375333	144.5631696	109.6402337	97.07734103
1A5 x 1E4	C5.2	122.4868448	98.56379539	94.16805925	108.9229595	64.77106176	50.61289167	128.56437	115.9043164	81.41373214
1A5 x 1E4	C51.1	126.8299393	80.95133587	62.85174085	94.018625	69.96709889	48.91384866	134.4387948	92.10839897	82.27423162
1A5 x 1E4	C51.2	NA	129.402	90.195	125.5095	103.489	91.9435	136.676	120.114	108.526
1A5 x 1E4	C52.1	149.9309667	139.4417	121.3661	102.1914667	91.1401	81.41772083	144.6253333	151.4207333	147.6338333
1A5 x 1E4	C53.1	144.8260667	105.0682333	73.24316667	132.9782122	123.8044111	108.7698917	149.735875	128.65875	114.4807833
1A5 x 1E4	C54.1	135.5046667	90.06941667	78.36625	99.638875	62.7065	45.8876	110.648125	92.74	81.227
1A5 x 1E4	C55.1	109.167344	107.2654064	100.8906894	57.26538036	61.84735752	51.71754559	106.18113	73.07108507	64.8809356
1A5 x 1E4	C55.2	135.1879165	115.0362036	107.6181829	87.86478712	80.1432212	62.87777342	125.08156	91.30203841	74.81903333
1A5 x 1E4	C56.1	145.842725	86.43894667	65.59886	115.9547068	109.7606541	98.63940602	145.4812875	114.9390147	108.0668241
1A5 x 1E4	C56.2	122.2490264	121.5312937	103.0517146	77.84124999	70.86354445	58.7452503	99.59983334	90.83019429	62.575715
1A5 x 1E4	C57.1	144.5168427	91.01843401	69.39815666	90.03664024	81.25058572	62.37663338	133.6969909	114.0097167	100.2970748
1A5 x 1E4	C57.2	139.5225179	129.0397701	118.6768624	136.1172544	82.07330555	79.93935533	147.9636867	144.962802	110.863324
1A5 x 1E4	C59.2	138.3616443	123.1176089	108.8007682	112.8022248	95.06695825	84.43780667	152.3203889	157.2451458	150.3229919
1A5 x 1E4	C6.1	140.369809	76.11906	61.99127151	85.73941124	62.67687485	44.5367	114.6468019	83.5593218	72.51994193
1A5 x 1E4	C60.1	143.2763818	133.1723465	113.3340635	116.2300829	91.46114155	71.00761305	139.8293823	130.9212085	120.1724615
1A5 x 1E4	C60.2	133.4199495	78.84219596	66.76915498	98.95501287	62.58574033	56.96450476	110.4542643	97.89470158	70.73642667

1A5 x 1E4	C61.1	140.4823907	129.3644404	123.0604814	106.8338879	116.219205	72.27605	123.9249949	114.8183606	105.1328956
1A5 x 1E4	C61.2	151.352	129.3785	119.10325	NA	NA	NA	NA	NA	NA
1A5 x 1E4	C62.1	136.1648143	78.11690191	57.21065016	112.6051667	93.17224262	85.99448833	134.4011389	92.88533651	87.48232
1A5 x 1E4	C62.2	136.1580048	125.5261103	113.8715245	100.5129568	58.69548398	46.13089015	119.7205857	105.9849997	84.67107476
1A5 x 1E4	C63.1	142.5026762	130.5333909	121.1775795	91.47786315	67.20438607	47.365845	127.3047442	122.857718	122.6557621
1A5 x 1E4	C7.1	141.5204583	131.4315708	121.2324941	107.0507563	85.77393214	64.21690625	133.6570833	127.1418	75.51886667
1A5 x 1E4	C7.2	136.0962522	92.10649795	69.3716777	120.2493719	116.8331467	103.4156257	156.0892984	141.4524407	146.0501393
1A5 x 1E4	C8.1	129.047	146.4905939	140.9315711	137.959353	86.46366084	59.71768571	119.5967432	107.5144365	74.37524199
1A5 x 1E4	CR4_A1.1	139.9556049	138.0073314	122.8030192	120.6225381	47.92726329	47.05137667	115.2707845	91.49091524	76.67543944
1A5 x 1E4	CR4_A2.1	144.2044739	128.8940845	115.4417262	122.1748049	124.0750834	95.23080757	119.2809543	78.84947969	85.91690825
1A5 x 1E4	CR4_A2.2	132.4851678	94.4412177	71.75834965	114.9885022	99.15919555	82.27587525	140.0621167	136.48	127.7393418
1A5 x 1E4	CR4_A3.1	133.4120786	92.26691548	83.46434429	92.48945	71.28244444	63.00256667	117.039125	98.18359823	76.27530556
1A5 x 1E4	CR4_A3.2	132.4076667	115.7426567	93.26704953	98.72075	67.68665	60.30465	146.1632333	155.88444	145.7870667
1A5 x 1E4	CR4_A4.1	139.0089542	130.8030266	121.9852163	95.89821771	69.33165951	54.53366667	113.5860222	101.7604929	75.25519253
1A5 x 1E4	D1.1	128.2198073	99.31057183	94.87723454	68.54464988	62.15237103	52.74219065	110.2411657	64.06267241	65.10296911
1A5 x 1E4	D1.2	130.8457906	90.43009944	72.0139829	128.3467526	115.8741778	103.55543	143.0750406	151.1918897	130.1428483
1A5 x 1E4	D1.3	136.9333446	127.3751092	115.4084881	68.09081038	64.30700227	57.02493827	127.8084872	103.2388425	85.59412857
1A5 x 1E4	D10.1	137.13191	97.16337923	98.19009126	93.13030816	55.46310278	47.96614167	125.5779811	81.95287525	81.72203053
1A5 x 1E4	D11.1	137.4227722	128.4090567	116.0124194	104.4621952	85.031795	64.62406	152.0947143	148.1162556	140.3647691
1A5 x 1E4	D2.1	147.0093667	111.6559	91.41493636	105.253095	97.73143112	73.492095	109.4359262	110.30165	93.83195279
1A5 x 1E4	D3.1	140.3704502	120.6201148	105.9397132	102.1309036	66.12438462	55.45510715	137.2680826	108.4175022	94.5778123
1A5 x 1E4	D3.2	127.2232359	123.9091457	109.0111575	106.4264574	87.55285172	76.70290848	144.8806259	126.8359511	103.25651
1A5 x 1E4	D4.1	142.9247362	99.85726419	98.16665112	86.86838947	77.3569095	51.355195	126.8347138	112.9523967	86.03669192
1A5 x 1E4	D5.1	139.6082102	123.9581736	101.9029697	129.9543736	120.8089585	91.11336095	155.3814996	158.4681185	155.3631985
1A5 x 1E4	D7.1	141.4650625	90.737	68.1256	99.07035	72.71023833	55.702675	146.61775	145.754375	131.328

1A5 x 1E4	D7.2	142.8895	139.5806627	130.2345897	132.3254059	77.77676049	49.79751746	117.9996648	110.61985	64.02539464
1A5 x 1E4	D7.3	131.353625	117.7358583	118.582375	80.81006667	79.23994167	58.67323333	131.358	145.0743333	134.449
1A5 x 1E4	D9.1	141.3127633	102.2868183	75.38129524	155.4523	146.25109	131.66258	162.43075	164.8569633	155.3209733
1A5 x 1E4	D9.2	138.49125	99.77906587	100.5998273	95.95926784	86.737175	71.68969167	139.1101325	141.2166408	112.2440933
