# Supplementary material for: Quantitative Trait Locus Mapping of Melanization in the Plant Pathogenic Fungus Zymoseptoria tritici
Source: G3 (Bethesda). 2014 Oct 29;4(12):2519–33. doi: 10.1534/g3.114.015289 (PMC4267946; doi:10.1534/g3.114.015289)
Supplement: Supporting Information [file supp_g3.114.015289_TableS5.pdf]

**Table S5 Orthologs in *Z. tritici* to genes involved in melanin biosynthesis**

| Protein ID | Name [a] | Chromo-some | Start (bp) | Stop (bp) | Melanin pathway function | Melanin pathway | Mutant effect | Tested in *Z. tritici* | Highest RPKM mean [b] | RPKM Stdv | Reference [c] |
|---|---|---|---|---|---|---|---|---|---|---|---|
| 96592 | *PKS1*[1)] | 11 | 586456 | 593034 | Polyketide synthase encoding gene | DHN | Full loss of function | No | 7.8 (8wpi) ✖ | 0.6 | (Takano *et al.* 1995; Butler and Day 1998; Langfelder *et al.* 2003[2)]; Hu *et al.* 2012[1)]) |
| 68710 | *THR2*[2)] | 2 | 2514972 | 2515981 | 1;3;6;8-tetrahydroxynaht halene reductase encoding gene | DHN | Full loss of function | No | 132.5 (13dpi) | 41.8 | (Butler and Day 1998; Langfelder *et al.* 2003[2)]; Ipcho *et al.* 2012) |
| 18775 | *MgSCY1*[1)] | 1 | 963695 | 964159 | Syctalone dehydratase encoding gene | DHN | Full loss of function | No | 133.1 (8wpi) | 15 | (Butler and Day 1998; Langfelder *et al.* 2003[2)]; Ipcho *et al.* 2012) |
| 90337 | *SCY2*[3)] | 2 | 133146 | 133741 | Syctalone dehydratase encoding gene | DHN | Full loss of function | No | 13.6 (7dpi) ✖ | 9.4 | (Butler and Day 1998; Langfelder *et al.* 2003[2)]; Ipcho *et al.* 2012) |
| 87994 | *THR1*[4)] | 11 | 604612 | 605471 | 1;3;8-tetrahydroxynaht halene reductase encoding gene | DHN | Full loss of function | No | 231.2 (8wpi) ✖ | 43.3 | (Butler and Day 1998; Langfelder *et al.* 2003[2)]; Ipcho *et al.* 2012) |
| 35451 | *LAC1*[5)] | 2 | 140750 | 142914 | Laccase encoding gene | DHN/ DOPA ? | Reduced | No | 24.3 (7dpi) ✖ | 12.4 | (Lin *et al.* 2012; Upadhyay *et al.* 2013) |
| 36350 | *LAC2*[5)] | 2 | 144093 | 146209 | Laccase encoding gene | DHN/ DOPA ? | Reduced | No | 40.8 (7dpi) ✖ | 24.2 | (Lin *et al.* 2012; Upadhyay *et al.* 2013) |
| 42222 | *LAC3*[5)] | 5 | 2279071 | 2281252 | Laccase encoding gene | DHN/ DOPA ? | Reduced | No | 38.0 (7dpi) ✖ | 22 | (Lin *et al.* 2012; Upadhyay *et al.* 2013) |

| | | | | | | | | | | | |
|---|---|---|---|---|---|---|---|---|---|---|---|
| 42793 | *LAC4*[5)] | 5 | 2282533 | 2284432 | Laccase encoding gene | DHN/ DOPA ? | Reduced | No | 32.6 (7dpi) | 20.8 | (LIN *et al.* 2012; UPADHYAY *et al.* 2013) |
| 32322 | *LAC5*[5)] | 1 | 5292579 | 5294444 | Laccase encoding gene | DHN/ DOPA ? | Reduced | No | 56.7 (7dpi) ✖ | 37.8 | (LIN *et al.* 2012; UPADHYAY *et al.* 2013) |
| 108040 | *LAC6*[5)] | 2 | 1650426 | 1652574 | Laccase encoding gene | DHN/ DOPA ? | Reduced | No | 193.8 (7dpi) ✖ | 49.5 | (LIN *et al.* 2012; UPADHYAY *et al.* 2013) |
| 38161 | *LAC7*[5)] | 3 | 2746686 | 2748706 | Laccase encoding gene | DHN/ DOPA ? | Reduced | No | 7.9 (8wpi) | 0.6 | (LIN *et al.* 2012; UPADHYAY *et al.* 2013) |
| 109666 | *LAC8*[5)] | 5 | 2492745 | 2495149 | Laccase encoding gene | DHN/ DOPA ? | Reduced | No | 451.7 (8wpi) ✖ | 74.3 | (LIN *et al.* 2012; UPADHYAY *et al.* 2013) |
| 52873 | *MgGpa1*[1)] | 1 | 2514902 | 2516229 | G alpha protein encoding gene | ? | Hampered | Yes | 132.5 (13dpi) | 17.6 | (SOLOMON *et al.* 2004; MEHRABI *et al.* 2009) |
| 68892 | *MgGpb1*[1)] | 2 | 2862220 | 2863783 | G beta protein encoding gene | ? | Hampered | Yes | 118.2 (13dpi) ✖ | 7.3 | (MEHRABI *et al.* 2009) |
| 76503 | *MgGpa3*[1)] | 10 | 1111808 | 1113544 | G alpha protein encoding gene | ? | Reduced | Yes | 33.9 (13dpi) | 4.9 | (MEHRABI *et al.* 2009) |
| 76502 | *MgHog1*[1)] | 10 | 1108132 | 1110166 | Mitogen-activated protein kinase encoding gene | ? | Full loss of function | Yes | 228.6 (7dpi) | 64.8 | (MEHRABI *et al.* 2006b) |
| 102121 | *MgSlt2*[1)] | 1 | 1331331 | 1332992 | Mitogen-activated protein kinase encoding gene | ? | Full loss of function | Yes | 205.4 (13dpi) ✖ | 14.8 | (MEHRABI *et al.* 2006a) |
| 73071 | *MgFus3*[1)] | 6 | 1182095 | 1184167 | Mitogen-activated protein kinase encoding gene | ? | Full loss of function | Yes | 163.1 (13dpi) ✖ | 3.9 | (COUSIN *et al.* 2006) |
| 37276 | *MVE1*[1)] | 2 | 2995014 | 2995823 | Velvet gene | ? | Full loss of function | Yes | 123.8 (7dpi) | 38.1 | (CHOI and GOODWIN 2011b) |
| 47503 | *MCC1*[1)] | 9 | 1594202 | 1595191 | C-type cyclin | ? | Increased | Yes | 35.5 (13dpi) | 7 | (CHOI and GOODWIN 2011a) |

| | | | | | | | | | | |
|---|---|---|---|---|---|---|---|---|---|---|
| | | | | | encoding gene | | | | | |
| 96591 | *PRF1*[5)] | 11 | 584834 | 585795 | Prefoldin chaperone encoding gene | DHN ? | ? | No | 91.4 (8wpi) | 11 | No Reference, but this is a gene just downstream from 96592 (*PKS1*), thus in the region of the expected DHN melanin pathway cluster. It shows conservation over other fungi and thus is believed to belong to the DHN melanin pathway. |
| 87993 | *CMR1*[4)] | 11 | 600309 | 603354 | Transcription factor encoding gene | DHN ? | Full loss of function | No | 12.7 (8wpi) ✖ | 1.6 | (Eliahu *et al.* 2007) |
| 99683 | *AYG1*[6)] | 3 | 2737599 | 2739046 | Unknown | DHN | Reduced | No | 34.0 (8wpi) ✖ | 3.4 | (Tsai *et al.* 1999[3)]) |
| 100584 | *AYG2*[7)] | 6 | 2057066 | 2058412 | Unknown | DHN | Reduced | No | 15.7 (13dpi) ✖ | 0.4 | (Tsai *et al.* 1999[3)]) |
| 72443 | *PEX13*[8)] | 5 | 1942248 | 1943680 | Peroxisome biogenesis factor encoding gene | ? | Full loss of function | No | 101.9 (7dpi) ✖ | 40.6 | (Fujihara *et al.* 2010) |
| 98537 | *PEX6*[4)] | 1 | 3057033 | 3061525 | Peroxisome assembly factor encoding gene | ? | Reduced | No | 21.1 (13dpi) | 2.2 | (Kimura *et al.* 2001) |
| 65403 | *CLAP1*[9)] | 1 | 1543207 | 1547078 | Copper-transporting ATPase encoding gene | ? | Reduced | No | 41.4 (7dpi) | 12.5 | (Parisot *et al.* 2002) |
| 57911 | *MAC1*[10)] | 4 | 1213598 | 1215218 | Transcription factor encoding gene | ? | Reduced | No | 201.6 (7dpi) ✖ | 48.5 | (Lin *et al.* 2006) |

[a] [1)] Name as found in the reference literature or on the joint genome institute (JGI) website for *Z. tritici* (http://genomeportal.jgi-psf.org/Mycgr3/Mycgr3.home.html). [2)] Name given by us. Similar name given as found in ortholog of *Colletotrichum lagenarium*. [3)] Two syctalone dehydratases (Protein ID: 18775 / 90337) were found when blasting the *SNOG_11564.3* (Ipcho *et al.* 2012) gene against *Z. tritici*. As 18775 had the name '*MgSCY1*' (JGI), we gave 90337 the name '*SCY2*'. [4)] Same name given as found in ortholog of *Colletotrichum lagenarium*. [5)] Name given by us by abbreviating the enzyme encoded by the gene. [6)] Same name

given as found in ortholog of *Aspergillus fumigatus*. [7)] Name given by us. Similar name given as found in ortholog of *Aspergillus fumigatus*. [8)] Same name given as found in ortholog of *Colletotrichum orbiculare*. [9)] Same name given as found in ortholog of *Colletotrichum lindemuthianum*. [10)] Same name given as found in ortholog of *Cryptococcus neoformans*.

[b] We had chosen a RKPM value of 2 as an expression threshold. All the genes associated with melanization showed ≥ 2 RPKM values. Hence all of the orthologs could affect melanization in *Z. tritici.* ✖ Indicates significant changes in transcript abundances over time.

[c] [1)] Hu, Y., X. Hao, J. Lou, P. Zhang, J. Pan et al., 2012 A PKS gene, pks-1, is involved in chaetoglobosin biosynthesis, pigmentation and sporulation in *Chaetomium globosum*. Science China-Life Sciences 55: 1100–1108.

[2)] Langfelder, K., M. Streibel, B. Jahn, G. Haase, and A. A. Brakhage, 2003 Biosynthesis of fungal melanins and their importance for human 11 pathogenic fungi. Fungal Genet. Biol. 38: 143–158.

[3)] Tsai, H. F., M. H. Wheeler, Y. C. Chang, and K. J. Kwon-Chung, 1999 A developmentally regulated gene cluster involved in conidial pigment biosynthesis in *Aspergillus fumigatus*. J. Bacteriol. 181: 6469–6477.
