# Supplementary material for: Quantitative Trait Locus Mapping of Melanization in the Plant Pathogenic Fungus Zymoseptoria tritici
Source: G3 (Bethesda). 2014 Oct 29;4(12):2519–33. doi: 10.1534/g3.114.015289 (PMC4267946; doi:10.1534/g3.114.015289)
Supplement: Supporting Information [file supp_g3.114.015289_TableS6.pdf]

Table S6 Summary of QTL positions and effects for cross 3D1 x 3D7.

Environment	Colony age (dpi)	QTL peak marker [a]	Chromosome	Estimated position of peaking marker (cM)	Estimated position of peaking marker (kb)	LOD score at peak	P-value	Mean 1A5 allele (grey values)	Mean 1E4 allele (grey values)	Mean difference	Allele effect [b]	Percentage of variance explained by QTL (%)	Estimated position of proximal marker (kb) [c]	Estimated position of distal marker (kb) [c]	Bayes confidence interval length (kb)
Cold	8	c10.loc107 (p)	10	107.13	646	8.29	< 0.001	135.78	129.09	6.69	3D1	13.6	634	673	39
Cold	8	12_143193	12	5.74	143	4.85	0.002	134.56	129.28	5.27	3D1	9.2	29	305	276
Cold	11	8_1250811	8	140.32	1251	4.07	0.01	101.72	112.39	10.66	3D7	7.1	897	2228	1332
Cold	11	c10.loc108 (p)	10	108.13	649	9.55	< 0.001	116.28	100.17	16.11	3D1	15.6	634	673	39
Cold	11	11_535446	11	77.83	535	4.32	0.006	113.31	102.18	11.13	3D1	7.4	447	1332	885
Control	8	c11.loc82 (p)	11	82.06	581	32.2	< 0.001	118	93.73	24.27	3D1	39.2	560	603	43
Control	11	5_871061	5	157.26	871	4.38	0.003	74.99	85.47	10.48	3D7	7.5	449	2799	2350
Control	11	c11.loc81 (p)	11	81.06	571	30.79	< 0.001	93.62	69.23	24.4	3D1	38.2	549	603	54
Control	14	5_958098	5	160.33	958	3.78	0.016	61.52	69.96	8.43	3D7	6.5	449	2799	2350
Control	14	c11.loc80 (p)	11	80.06	560	21.17	< 0.001	75.79	57.28	18.51	3D1	30	512	603	91
Fungicide	8	c11.loc81 (p)	11	81.06	571	34	< 0.001	124.55	100.17	24.38	3D1	45.8	549	592	43
Fungicide	11	c11.loc82 (p)	11	82.06	581	23.8	< 0.001	96.79	75.05	21.74	3D1	30.4	549	625	76
Fungicide	14	1_1740191	1	193.5	1740	3.64	0.014	64.87	73.03	8.16	3D7	6.3	1063	4267	3203
Fungicide	14	c11.loc81 (p)	11	81.06	571	19.34	< 0.001	78.24	60.96	17.28	3D1	27.2	535	614	79

[a] A (p) indicates that a pseudomarker provided the highest LOD score. In all other cases a true marker provided the highest LOD score.

[b] 3D7 indicates that the parental 3D7 allele provided the higher phenotypic mean then the parental 3D1 allele, while 3D1 indicates that the parental 3D1 allele provided the higher phenotypic mean then the parental 3D7 allele.

[c] Markers flanking Bayes confidence interval.
