# Supplementary material for: Quantitative Trait Locus Mapping of Melanization in the Plant Pathogenic Fungus Zymoseptoria tritici
Source: G3 (Bethesda). 2014 Oct 29;4(12):2519–33. doi: 10.1534/g3.114.015289 (PMC4267946; doi:10.1534/g3.114.015289)
Supplement: Supporting Information [file supp_g3.114.015289_TableS7.pdf]

Table S7 Summary of QTL positions and effects for cross 1A5 x 1E4.

Environment	Colony age (dpi)	QTL peak marker [a]	Chromosome	Estimated position of peaking marker (cM)	Estimated position of peaking marker (kb)	LOD score at peak	P-value	Mean 1A5 allele (grey values)	Mean 1E4 allele (grey values)	Mean difference	Allele effect [b]	Percentage of variance explained by QTL (%)	Estimated position of proximal marker (kb) [c]	Estimated position of distal marker (kb) [c]	Bayes confidence interval length (kb)
Cold	8	1_2840762	1	445.26	2841	3.6	0.029	134.52	138.11	3.58	1E4	6.3	2604	3275	671
Cold	8	c2.loc226 (p)	2	226.07	1699	10	< 0.001	139.11	133.43	5.69	1A5	17	1646	1819	173
Cold	8	c3.loc208 (p)	3	208.04	1301	8.52	< 0.001	133.71	138.79	5.08	1E4	12.1	869	1749	880
Cold	11	1_1553785	1	219.31	1554	8.59	< 0.001	103.25	117.72	14.47	1E4	14.3	1020	1627	607
Cold	11	2_1814469	2	243.95	1814	3.68	0.018	114.88	105.17	9.71	1A5	6.4	1428	2270	842
Cold	11	5_1699464	5	224.63	1699	5.9	< 0.001	116.26	104.11	12.15	1A5	10.1	773	1911	1138
Cold	11	8_429575	8	58.42	430	7.65	< 0.001	117.28	103.6	13.68	1A5	12.8	348	446	99
Cold	14	1_1028414	1	157.63	1028	7.65	< 0.001	91.91	105.6	13.69	1E4	13	1014	1589	575
Cold	14	c3.loc249 (p)	3	249.04	1780	3.44	0.046	103.29	94.17	9.12	1A5	5.7	1621	1968	347
Cold	14	5_1593047	5	215.71	1593	5.13	< 0.001	104.07	92.71	11.36	1A5	8.9	1230	1913	683
Cold	14	7_841106	7	181.47	841	4.01	0.014	103.42	93.3	10.13	1A5	7	428	1893	1464
Cold	14	8_397588	8	55.79	398	6.62	< 0.001	105.18	92.37	12.81	1A5	11.3	348	446	99
Control	8	c1.loc106 (p)	1	106.14	771	3.4	0.037	106.6	97.98	8.62	1A5	5.8	441	5371	4930
Control	8	2_1526778	2	205.66	1527	7.34	< 0.001	108.31	95.86	12.45	1A5	12.3	1455	1646	191
Control	11	1_3069693	1	492.19	3070	6.24	< 0.001	76.24	88.1	11.86	1E4	10.9	2919	3249	331
Control	14	1_3006378	1	480.86	3006	5.32	< 0.001	61.69	72.61	10.92	1E4	9.2	2854	5652	2798
Control	14	3_1739730	3	245.64	1740	4.29	0.005	62.41	72.25	9.84	1E4	7.5	1494	1968	474
Control	14	4_421356	4	55.21	421	5.61	< 0.001	61.71	72.91	11.2	1E4	9.5	161	476	315
Control	14	5_1919504	5	249.23	1920	4.64	0.001	62.31	72.55	10.24	1E4	8	1385	2105	721

Control	14	11_523597	11	66.72	524	4.71	< 0.001	72.97	62.64	10.33	1A5	8.2	329	581	252
Fungicide	8	2_1470426	2	201.05	1470	7.55	< 0.001	140.56	127.73	12.83	1A5	12.9	1464	1814	350
Fungicide	8	c4.loc245 (p)	4	245.04	1471	7.17	< 0.001	127.64	140.17	12.53	1E4	12.3	415	1802	1386
Fungicide	11	1_3249298	1	511.19	3249	6.55	< 0.001	108.84	124.9	16.05	1E4	11.2	2974	3275	301
Fungicide	11	2_1632971	2	211.37	1633	4.01	0.011	123.66	111.06	12.6	1A5	7	1428	2375	947
Fungicide	11	3_1371178	3	221.45	1371	4.34	0.007	111.23	124.29	13.06	1E4	7.6	1308	2180	872
Fungicide	11	c4.loc52 (p)	4	52.04	418	9.15	< 0.001	108.37	126.55	18.18	1E4	14.8	415	1094	679
Fungicide	14	1_3249289	1	511.19	3249	6.24	< 0.001	93.34	109.6	16.26	1E4	10.7	2840	5735	2894
Fungicide	14	2_1632971	2	211.37	1633	4.14	0.01	108.58	95.31	13.27	1A5	7.2	1428	2084	657
Fungicide	14	3_1739730	3	245.64	1740	4.22	0.01	95.5	108.91	13.41	1E4	7.4	1315	2206	891
Fungicide	14	c4.loc54 (p)	4	54.04	420	9.47	< 0.001	92.37	111.73	19.36	1E4	16.2	417	426	9
Fungicide	14	6_2299166	6	289.62	2299	3.56	0.027	108.33	96	12.33	1A5	6.2	1166	2438	1272
Fungicide	14	c11.loc70 (p)	11	70.07	533	4.48	0.007	109.68	96.1	13.58	1A5	7.8	271	1160	889

[a] A (p) indicates that a pseudomarker provided the highest LOD score. In all other cases a true marker provided the highest LOD score.
[b] 1E4 indicates that the parental 1E4 allele provided the higher phenotypic mean then the parental 1A5 allele, while 1A5 indicates that the parental 1A5 allele provided the higher phenotypic mean then the parental 1E4 allele.
[c] Markers flanking Bayes confidence interval.
