# Supplementary material for: Quantitative Trait Locus Mapping of Melanization in the Plant Pathogenic Fungus Zymoseptoria tritici
Source: G3 (Bethesda). 2014 Oct 29;4(12):2519–33. doi: 10.1534/g3.114.015289 (PMC4267946; doi:10.1534/g3.114.015289)
Supplement: Supporting Information [file supp_g3.114.015289_TableS8.pdf]

Table S8 Summary of genes affected by sequence variation within each Bayes confidence interval for cross 3D1 x 3D7, excluding all genes containing no sequence variation or with synonymous SNPs only.

Environment	Colony age (dpi)	Chromosome	Estimated position of peaking marker (kb)	LOD score at peak	P-value	Estimated position of proximal marker (kb) [a]	Estimated position of distal marker flanking (kb) [a]	Bayes confidence interval length (kb)	Number of sequence variations [b]	Number of genes [b]	Number of genes affected by sequence variations [b]	Percentage of total genes affected by sequence variations (%) [b]	Number of sequence variation affected genes with unknown function [b]	Percentage of total sequence variation affected genes with unknown function (%) [b]	Number of sequence variation affected genes with significant changes in transcript abundances [b]	Percentage of total sequence variation affected genes with significant changes in transcript abundances (%) [b]
Fungicide	14	1	1740	3.64	0.014	1063	4267	3203	4342	1130	858	76	296	34	349	41
Control	11	5	871	4.38	0.003	449	2799	2350	2551	679	504	74	170	34	220	44
Control	14	5	958	3.78	0.016	449	2799	2350	2550	679	504	74	170	34	220	44
Cold	11	8	1251	4.07	0.01	897	2228	1332	1661	433	304	70	112	37	114	38
Cold	8	10	646	8.29	< 0.001	634	673	39	59	15	12	80	2	17	7	58
Cold	11	10	649	9.55	< 0.001	634	673	39	59	15	12	80	2	17	7	58
Control	8	11	581	32.2	< 0.001	560	603	43	8	14	4	29	0	0	1	25
Control	11	11	571	30.79	< 0.001	549	603	54	13	16	6	38	1	17	3	50
Fungicide	8	11	571	34	< 0.001	549	592	43	12	14	6	43	1	17	3	50
Fungicide	11	11	581	23.8	< 0.001	549	625	76	28	24	12	50	4	33	5	42
Fungicide	14	11	571	19.34	< 0.001	535	614	79	50	26	13	50	5	38	6	46
Control	14	11	560	21.17	< 0.001	512	603	91	67	29	16	55	5	31	6	38
Cold	11	11	535	4.32	0.006	447	1332	885	1025	277	190	69	78	41	86	45
Cold	8	12	143	4.85	0.002	29	305	276	441	78	56	72	22	39	23	41

[a] Markers flanking Bayes confidence interval.
[b] Numbers refer to within Bayes confidence interval.
