# Supplementary material for: Quantitative Trait Locus Mapping of Melanization in the Plant Pathogenic Fungus Zymoseptoria tritici
Source: G3 (Bethesda). 2014 Oct 29;4(12):2519–33. doi: 10.1534/g3.114.015289 (PMC4267946; doi:10.1534/g3.114.015289)
Supplement: Supporting Information [file supp_g3.114.015289_TableS9.pdf]

Table S9 Summary of genes affected by sequence variation within each Bayes confidence interval for cross 1A5 x 1E4 excluding all genes containing no sequence variation or with synonymous SNPs only.

Environment	Colony age (dpi)	Chromosome	Estimated position of peaking marker (kb)	LOD score at peak	P-value	Estimated position of proximal marker (kb) [a]	Estimated position of distal marker flanking (kb) [a]	Bayes confidence interval length (kb)	Number of sequence variations [b]	Number of genes [b]	Number of genes affected by sequence variations [b]	Percentage of total genes affected by sequence variations (%) [b]	Number of sequence variation affected genes with unknown function [b]	Percentage of total sequence variation affected genes with unknown function (%) [b]	Number of sequence variation affected genes with significant changes in transcript abundances [b]	Percentage of total sequence variation affected genes with significant changes in transcript abundances (%) [b]
Fungicide	11	1	3249	6.55	< 0.001	2974	3275	301	677	113	84	74	25	30	30	36
Control	11	1	3070	6.24	< 0.001	2919	3249	331	725	121	90	74	27	30	33	37
Cold	14	1	1028	7.65	< 0.001	1014	1589	575	784	182	145	80	49	34	72	50
Cold	11	1	1554	8.59	< 0.001	1020	1627	607	853	195	154	79	54	35	74	48
Cold	8	1	2841	3.6	0.029	2604	3275	671	1257	243	184	76	60	33	72	39
Control	14	1	3006	5.32	< 0.001	2854	5652	2798	3855	963	704	73	237	34	278	39
Fungicide	14	1	3249	6.24	< 0.001	2840	5735	2894	3956	997	732	73	244	33	294	40
Control	8	1	771	3.4	0.037	441	5371	4930	6888	1673	1245	74	436	35	511	41
Cold	8	2	1699	10	< 0.001	1646	1819	173	225	48	38	79	18	47	28	74
Control	8	2	1527	7.34	< 0.001	1455	1646	191	240	59	44	75	15	34	15	34
Fungicide	8	2	1470	7.55	< 0.001	1464	1814	350	437	101	78	77	31	40	40	51
Fungicide	14	2	1633	4.14	0.01	1428	2084	657	803	188	139	74	59	42	60	43

Cold	11	2	1814	3.68	0.018	1428	2270	842	1075	262	195	74	88	45	85	44
Fungicide	11	2	1633	4.01	0.011	1428	2375	947	1193	301	222	74	95	43	95	43
Cold	14	3	1780	3.44	0.046	1621	1968	347	366	116	81	70	20	25	25	31
Control	14	3	1740	4.29	0.005	1494	1968	474	465	150	103	69	28	27	34	33
Cold	8	3	1301	8.52	< 0.001	869	1749	880	933	266	186	70	69	37	68	37
Fungicide	11	3	1371	4.34	0.007	1308	2180	872	949	282	197	70	62	31	71	36
Fungicide	14	3	1740	4.22	0.01	1315	2206	891	967	290	202	70	64	32	76	38
Fungicide	14	4	420	9.47	< 0.001	417	426	9	7	2	1	50	0	0	0	0
Control	14	4	421	5.61	< 0.001	161	476	315	274	80	59	74	19	32	21	36
Fungicide	11	4	418	9.15	< 0.001	415	1094	679	905	213	153	72	73	48	67	44
Fungicide	8	4	1471	7.17	< 0.001	415	1802	1386	1676	417	297	71	131	44	125	42
Control	14	5	1920	4.64	0.001	1385	2105	721	744	215	156	73	56	36	68	44
Cold	14	5	1593	5.13	< 0.001	1230	1913	683	764	219	162	74	54	33	65	40
Cold	11	5	1699	5.9	< 0.001	773	1911	1138	1272	352	259	74	86	33	102	39
Fungicide	14	6	2299	3.56	0.027	1166	2438	1272	1099	342	244	71	82	34	93	38
Cold	14	7	841	4.01	0.014	428	1893	1464	1194	440	224	51	90	40	103	46
Cold	11	8	430	7.65	< 0.001	348	446	99	132	27	22	81	9	41	7	32
Cold	14	8	398	6.62	< 0.001	348	446	99	132	27	22	81	9	41	7	32
Control	14	11	524	4.71	< 0.001	329	581	252	324	84	67	80	24	36	37	55
Fungicide	14	11	533	4.48	0.007	271	1160	889	1064	275	203	74	76	37	100	49

[a] Markers flanking Bayes confidence interval.
[b] Numbers refer to within Bayes confidence interval.
