# Supplementary material for: Quantitative Trait Locus Mapping of Melanization in the Plant Pathogenic Fungus Zymoseptoria tritici
Source: G3 (Bethesda). 2014 Oct 29;4(12):2519–33. doi: 10.1534/g3.114.015289 (PMC4267946; doi:10.1534/g3.114.015289)
Supplement: Supporting Information [file supp_g3.114.015289_TableS10.pdf]

Table S10 Genes within large-effect QTL regions (confidence intervals containing ≤ 30 candidate genes) for cross 3D1 x 3D7, excluding genes with no sequence variation or with only synonymous SNPs.

Protein ID [a]	Gene ontology Name	Gene ontology biological process	Gene ontology cellular component	Gene ontology molecular function	Number of Non-Syn SNPs [b]	Number of other sequence variations [b c]	Additional information	Highest RPKM mean [d]	RPKM Stdv
Chromosome 10 (Phenotype: 11 dpi cold)									
101405	catalytic activity	acetyl-CoA metabolic process; acetate metabolic process; pyruvate metabolic process;	cytosol; mitochondrion; acetate CoA-transferase complex;	acetate CoA-transferase activity; acetyl-CoA hydrolase activity;	2 (M)	0	/	71.4 (13dpi)	17.4
105917 °	5-methyltetrahydropteroyltriglutamate-homocysteine S-methyltransferase activity	methionine biosynthetic process; methylation;	Not described	5-methyltetrahydropteroyltriglutamate-homocysteine S-methyltransferase activity;	3 (M)	2 upstream (m), 1 UTR5 Prime (m)	/	148.0 (7dpi)	24.0
111014	proteolysis	proteolysis;	Not described	serine-type peptidase activity;	7 (M)	2 intron (m), 1 UTR5 Prime (m)	/	49.8 (7dpi)	43.3
96183	Not described	Not described	Not described	Not described	5 (M)	2 intron (m)	/	0 (No	0.0

								Specific Day)	
76249 °	protein kinase activity	protein phosphorylation; serine family amino acid metabolic process;	Not described	ATP binding; protein serine/threonine kinase activity;	3 (M)	0	Name: MgPbs2 (Hypothetical MAP kinase kinase (MAPKK) involved in the osmosensing signal-transduction pathway, activated under severe osmotic)	139.2 (7dpi)[illegible]	31.8
101408 # °	integral to membrane	transmembrane transport;	integral to membrane;	Not described	2 (M)	0	Transporter - auxin efflux carrier-like with six predicted transmembrane regions	168.0 (8wpi) [illegible]	24.3
48576 °	amino acid transport	amino acid transmembrane transport;	integral to membrane;	amino acid transmembrane transporter activity;	18 (M)	1 intron (m)	/	15.5 (8wpi)[illegible]	1.4
76256 °	N-acetyltransferase activity	acyl-carrier-protein biosynthetic process;	Not described	N-acetyltransferase activity;	2 (M)	1 frame shift (H)	/	24.9 (13dpi)	8.6

48696	DNA binding	regulation of transcription, DNA-dependent;	transcription factor complex;	sequence-specific DNA binding transcription factor activity; DNA binding;	0	1 upstream (m), 1 intron (m)	Name: MgSwi6 (Hypothetical transcription factor that is related to DNA binding component of the SBF complex (Swi4p-Swi6p) in S. cerevisiae)	12.3 (13dpi)[illegible]	3.1
76259	Not described	oxidation-reduction process; tryptophan metabolic process; peroxidase reaction; response to oxidative stress; methane metabolic process;	Not described	heme binding; catalase activity;	1 (M)	0	/	70.7 (8wpi)[illegible]	12.6
101410	protein binding	Not described	Not described	Not described	1 (M)	1 upstream (m), 1 UTR5 Prime (m)	/	413.6 (7dpi)[illegible]	64.8
105922	Not described	Not described	Not described	Not described	0	1 UTR5 Prime (m)	/	48.9 (13dpi)[illegible]	6.7
Chromosome 11 (Phenotype: 8 dpi control)									
96588 °	Not described	Not described	intracellular;	zinc ion binding; nucleic acid binding;	1 (M)	1 intron (m)	/	2.0 (8wpi)	0.6

50123	ubiquitin thiolesterase activity	ubiquitin-dependent protein catabolic process; protein deubiquitination;	Not described	ubiquitin thiolesterase activity; cysteine-type peptidase activity;	0 (M)	2 intron (m)	/	31.9 (8wpi)	3.9
96591 # ° $	protein folding	protein folding;	prefoldin complex;	unfolded protein binding;	0 (M)	1 upstream (m)	/	91.4 (8wpi)	11.0
96592 ° $	catalytic activity	biosynthetic process;	Not described	hydrolase activity, acting on ester bonds; transferase activity;	3 (M)	0	Name: *PKS1* (polyketide synthase gene highly similar to those involved in fungal melanin biosynthesis; contains conserved domains typically found in PKSs that synthesize fungal pigments)	7.8 (8wpi) [illegible]	0.6

[a] A # Indicates the QTL peak is positioned closest to this gene or within the gene. A ° Indicates candidate genes with a higher priority of contributing to melanization compared to unmarked candidate genes. $ Indicates orthologs in *Z. tritici* to genes involved in melanin biosynthesis (Table S5).

[b] The letter within brackets following the number of a specific sequence polymorphism refers to the likely impact of the sequence polymorphism according to SnpEff. H=high, M=moderate, L=low, m=modifier.

[c] Other sequence variations include codon change plus insertion/deletion, codon insertion/deletions, 100 bp upstream or downstream, frame shift, intron, splice site acceptor/donor, start gained/lost, stop gained/lost, untranslated regions (UTR).

[d] We chose a RKPM value of 2 as an expression threshold. [illegible]A [illegible]Indicates significant changes in transcript abundances over time.
