# Supplementary material for: Quantitative Trait Locus Mapping of Melanization in the Plant Pathogenic Fungus Zymoseptoria tritici
Source: G3 (Bethesda). 2014 Oct 29;4(12):2519–33. doi: 10.1534/g3.114.015289 (PMC4267946; doi:10.1534/g3.114.015289)
Supplement: Supporting Information [file supp_g3.114.015289_TableS11.pdf]

Table S11 Genes within large-effect QTL regions (confidence intervals containing ≤ 30 candidate genes) for cross 1A5 x 1E4, excluding genes with no sequence variation or with only synonymous SNPs.

Protein ID [a]	Gene ontology Name	Gene ontology biological process	Gene ontology cellular component	Gene ontology molecular function	Number of Non-Syn SNPs [b]	Number of other sequence variations [b c]	Additional information	Highest RPKM mean [d]	RPKM Stdv
Chromosome 4 (Phenotype: 14 dpi fungicide)									
92291 # °	transcription factor activity	regulation of transcription from RNA polymerase II promoter;	nucleus;	sequence-specific DNA binding RNA polymerase II transcription factor activity; zinc ion binding;	0 (M)	4 downstream (m), 1 upstream (m), 2 intron (m)	/	80.7 (7 dpi)	21.9
Chromosome 8 (Phenotype: 11 dpi cold)									
94941	Not described	Not described	Not described	Not described	0 (M)	4 intron (m)	/	14.7 (13dpi)	4.8
94942	Not described	Not described	Not described	Not described	16 (M)	0	/	0.2 (8wpi)	0.2
14031	Not described	Not described	Not described	Not described	9 (M)	1 upstream (m)	/	3.2 (13dpi)	1.6
74422	Not described	Not described	Not described	Not described	0 (M)	1 intron (m)	/	33.2 (13dpi)	5.9

94946	Not described	Not described	Not described	Not described	7 (M)	0	/	4.3 (13dpi)	2.9
100879	glucose-6-phosphate dehydrogenase activity	pentose-phosphate shunt; response to hydrogen peroxide; glutathione metabolic process;	cytoplasm;	glucose-6-phosphate dehydrogenase activity; NADP binding;	0 (M)	1 downstream (m), 2 UTR5 Prime	Name: G6PD (EC 1.1.1.49 glucose-6-phosphate dehydrogenase; NADP-glucose-6-phosphate dehydrogenase; Zwischenferment; D-glucose 6-phosphate dehydrogenase; glucose 6-phosphate dehydrogenase (NADP); NADP-dependent glucose 6-phosphate dehydrogenase; 6-phosphoglucose dehydrogenase; Entner-Doudoroff enzyme; glucose-6-phosphate 1-dehydrogenase; G6PDH; GPD)	623.7 (8wpi)	33.8

74429	structural constituent of ribosome	translation; ribosome biogenesis;	ribosome;	structural constituent of ribosome;	0 (M)	2 downstream (m), 1 upstream (m), 2 intron (m), 4 UTR3 Prime	/	2315.0 (13dpi)	194.8
87034	Not described	ribosomal large subunit assembly;	nucleolus; preribosome, large subunit precursor;	5S rRNA binding; rRNA primary transcript binding;	3 (M)	0	/	58.2 (13dpi)	6.8
87035	DNA binding	transcription, DNA-dependent; response to DNA damage stimulus;	membrane;	DNA binding; ATP binding; helicase activity;	1 (M)	1 downstream (m)	Homolog of Saccharomyces cerevisiae INO80, a Swi2/Snf2-related ATPase that forms a large complex, containing actin and several actin-related proteins, that has chromatin remodeling activity and 3' to 5' DNA helicase activity in vitro	36.0 (13dpi)	6.0

46572	Not described	Not described	Not described	Not described	0 (M)	1 upstream (m), 1 intron (m)	/	107.2 (7dpi) ▯	18.9
27012	phospholipid biosynthetic process	phospholipid biosynthetic process;	membrane;	phosphotransferase activity, for other substituted phosphate groups;	0 (M)	2 upstream (m)	Putative CDP-alcohol phosphatidyltransferase. Shows some sequence similarity to cardiolipin synthase. Predicted signal peptide. Predicted membrane localization.	10.0 (7dpi)	5.5
110344 °	Not described	nuclear mRNA cis splicing, via spliceosome; mRNA transport;	nuclear cap binding complex;	RNA cap binding;	4 (M)	1 downstream (m), 2 frame shift (H), 1 intron (m)	/	48.6 (13dpi) ▯	5.2
46635	nucleus	nuclear mRNA splicing, via spliceosome; cellular bud site selection;	U2 snRNP;	Not described	0 (M)	2 intron (m)	/	69.1 (7dpi)	25.6

100881	protein kinase activity	protein phosphorylation; serine family amino acid metabolic process;	Not described	ATP binding; protein serine/threonine kinase activity;	0 (M)	3 intron (m), 1 UTR5 Prime (m)	Name: GSK3 (serine/threonine protein kinase, CMGC family, glycogen synthase kinase subfamily)	180.8 (13dpi) ▯	51.8
87038	Not described	Not described	Not described	nucleotide binding;	2 (M)	0	/	1.1 (7dpi)	1.8
29926 °	Not described	Not described	Not described	Not described	0 (M)	1 upstream (m), 6 intron (m)	/	31.7 (13dpi) ▯	3.2
74444 °	tRNA processing	mitochondrial tRNA wobble uridine modification;	mitochondrion;	flavin adenine dinucleotide binding;	10 (M)	2 intron (m)	/	31.5 (7dpi)	9.5
87039 #	Not described	Not described	membrane;	Not described	3 (M)	0	/	0 (No Specific Day)	0
46302 °	protein kinase activity	protein phosphorylation; serine family amino acid metabolic process;	Not described	ATP binding; protein serine/threonine kinase activity;	4 (M)	4 downstream (m), 2 intron (m)	/	21.6 (13dpi)	2.0

110348 °	nucleic acid binding	DNA catabolic process;	Not described	endonuclease activity; nucleic acid binding;	7 (M)	0	/	4.5 (7dpi)	4.5
94962 °	Not described	Not described	Not described	Not described	3 (M)	0	/	13.0 (7dpi)	7.7
110349 °	Not described	Not described	Not described	Not described	9 (M)	3 frame shift (H), 3 intron (m)	putative secreted protein unknown function, probable unique to M graminicola.	11.8 (7dpi)	8.3

[a] A # Indicates the QTL peak is positioned closest to this gene or within the gene. A ° Indicates candidate genes with a higher priority of contributing to melanization compared to unmarked candidate genes. [$] Indicates orthologs in *Z. tritici* to genes involved in melanin biosynthesis (Table S5).

[b] The letter within brackets following the number of a specific sequence polymorphism refers to the likely impact of the sequence polymorphism according to SnpEff. H=high, M=moderate, L=low, m=modifier.

[c] Other sequence variations include codon change plus insertion/deletion, codon insertion/deletions, 100 bp upstream or downstream, frame shift, intron, splice site acceptor/donor, start gained/lost, stop gained/lost, untranslated regions (UTR).

[d] We chose a RKPM value of 2 as an expression threshold. A Indicates significant changes in transcript abundances over time.
