# Supplementary figures and images for: Quantitative Trait Locus Mapping of Melanization in the Plant Pathogenic Fungus Zymoseptoria tritici
Source: G3 (Bethesda). 2014 Oct 29;4(12):2519–33. doi: 10.1534/g3.114.015289 (PMC4267946; doi:10.1534/g3.114.015289)

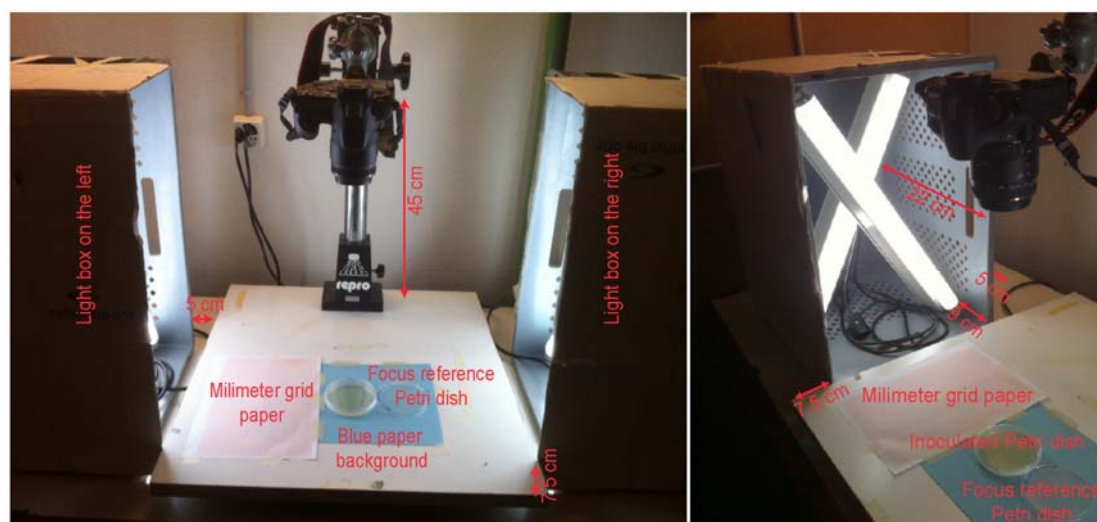

**Figure S1** Camera and light setup overview.

Supplement: Supporting Information [file supp_g3.114.015289_FigureS1.pdf]
