# Supplementary material for: Quantitative Trait Locus Mapping of Melanization in the Plant Pathogenic Fungus Zymoseptoria tritici
Source: G3 (Bethesda). 2014 Oct 29;4(12):2519–33. doi: 10.1534/g3.114.015289 (PMC4267946; doi:10.1534/g3.114.015289)
Supplement: Supporting Information [file supp_g3.114.015289_FigureS3.pdf]

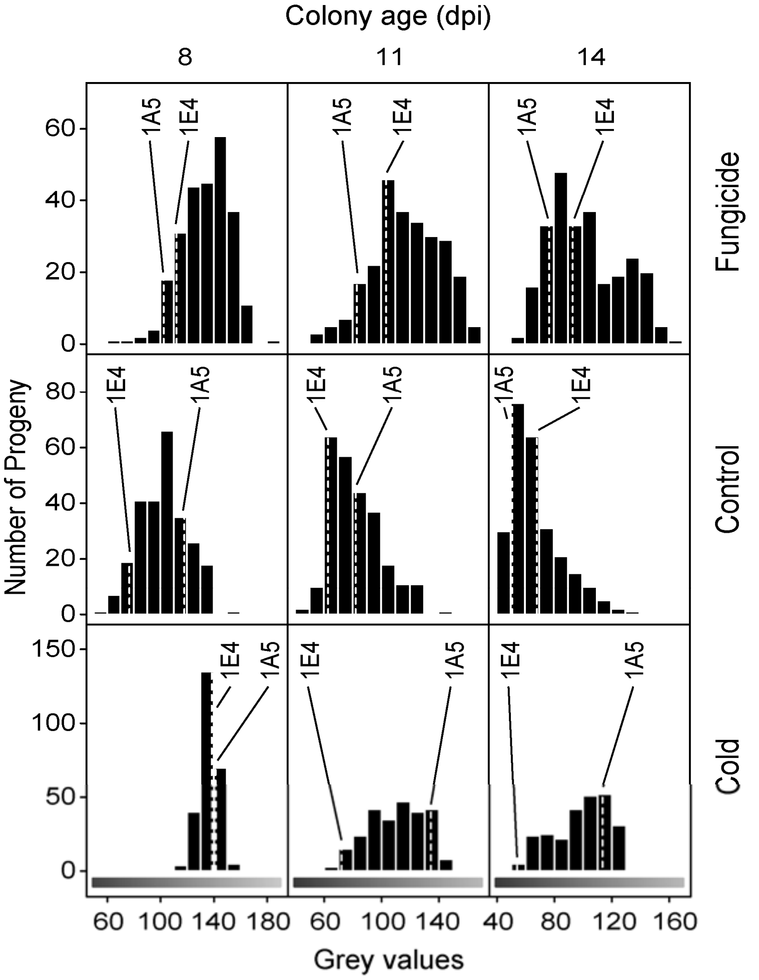

Figure S3 Melanization measured in the cross 1A5 x 1E4 over the three different environments. Each environment is represented by the three colony ages. The rug just above the grey values corresponds to the intensity of grey shading. Melanization increases as time progresses, indicated with lower mean values for older colonies.
