# Supplementary material for: Quantitative Trait Locus Mapping of Melanization in the Plant Pathogenic Fungus Zymoseptoria tritici
Source: G3 (Bethesda). 2014 Oct 29;4(12):2519–33. doi: 10.1534/g3.114.015289 (PMC4267946; doi:10.1534/g3.114.015289)
Supplement: Supporting Information [file supp_g3.114.015289_FigureS5.pdf]

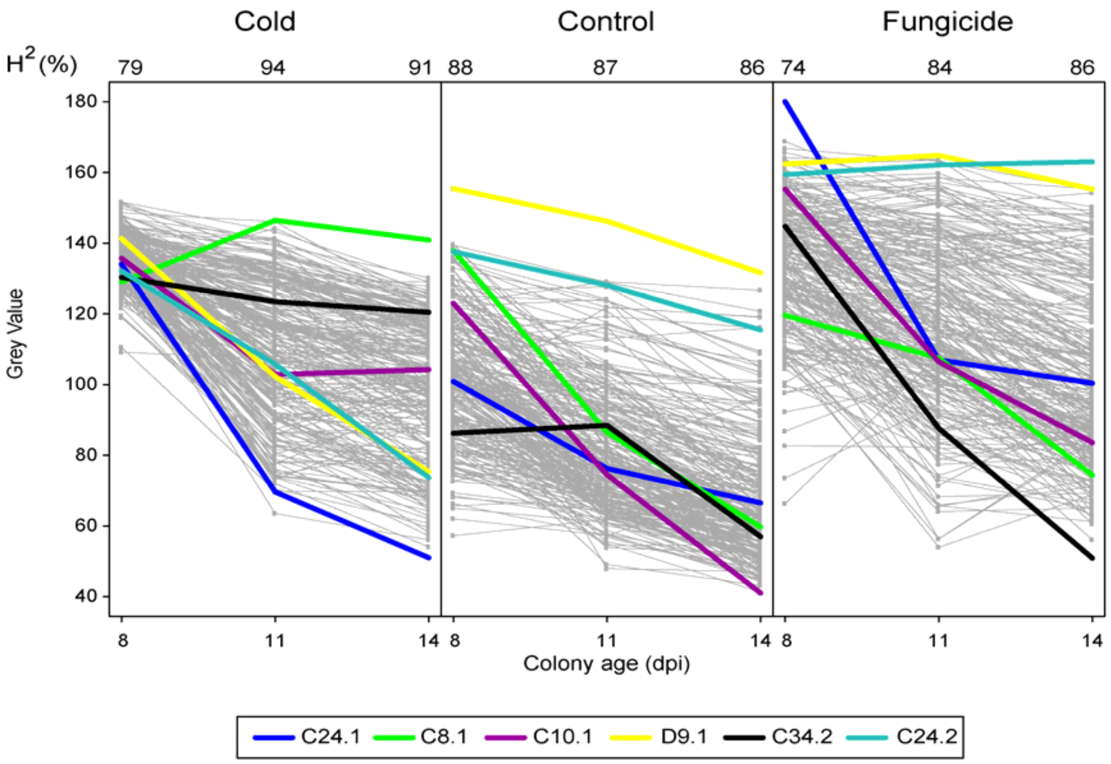

Figure S5 Norms of reaction for the cross 1A5 x1E4 across the three colony ages and environments. The two progeny with the most extreme (highest and lowest), complete phenotypes found at 14 dpi colony age in each environment are highlighted in the two orhter environments. Broad-sense heritability (H^2) values are indicated for each ECAM phenotype.
