# Supplementary material for: Quantitative Trait Locus Mapping of Melanization in the Plant Pathogenic Fungus Zymoseptoria tritici
Source: G3 (Bethesda). 2014 Oct 29;4(12):2519–33. doi: 10.1534/g3.114.015289 (PMC4267946; doi:10.1534/g3.114.015289)
Supplement: Supporting Information [file supp_g3.114.015289_FileS1.pdf]

File S1

Fiji Batch Macro

Important Macro Information:

The following macro can be used in Fiji. To run the macro, do the following:

1. Create a folder (e.g. ‘Main Folder’).
2. Place your JPEG images to be analyzed into the folder.
3. Create the following subfolders within the folder:
    a. FinalSummary
    b. HSVtemp
    c. Labels
    d. Macro Used
    e. Masks
    f. OutlinesAndBackground
    g. Results
4. Save the following macro-text in a normal text file into the folder ‘Macro Used’.
5. Open ImageJ/Fiji and select from the menu ‘Plugins -> Macros -> Run’.
6. Select the txt file, where the macro-text has been saved.
7. Select the ‘Main Folder’ and your macro should run automatically.

Additional notes:

1. The macro requires more or less circular colony growth of the target organism.
2. This macro as shown here is suitable for Fiji version 1.47i running on a mac computer. For a windows computer, singular forward slashes (/) need to be changed into double back slashes (\\), except for the forward slash in the command: (endsWith(list[i], "/").
3. The speed of the batch macro can be optimized be deactivating commands that generate masks (e.g all commands which contain ‘HSVtemp’). Deactivation can be conducted by applying two forward slashes in front of the command.
4. To avoid scoring of noise particles (including the polygon selection created during the macro) and fused colonies, we recommend to apply a subset onto the individual image results tables, by selecting for only particles providing a roundness value above 0.9. We used a loop in R to do so.

5. Settings most likely need to be adjusted if using a different organism then *Z. tritici* and different light and camera settings. Under same light and camera settings, we were able to apply the macro also for other plant pathogenic fungi, such as *Rhynchosporium commune*.

Detailed information regarding camera and light setup can be found in Table S2 and Figure S1. Detailed information regarding camera settings can be found in Table S3.

THE BATCH MACRO:

```
//******************************************************************************************
//Batch macro: “Image Analysis; Zymoseptoria trtici; Size and Grey Value Measure of Single Spore Colony on Petri Dish”

//Author: Mark Lendenmann, Plant Pathology Group, ETH Zurich

//Measures size and grey value of single spore colonies grown on Petri dishes.
//Creates single image results tables, labeled overlay masks on binary image, labeled outline masks on original image, overall summary table

//******************************************************************************************
// 1. SET SCALE: e.g. pixels to square millimeters / SET MEASURMEMENTS – change this to suit images

open("/Users/lmark/Desktop/Fiji Macro on this Mac/Callibration_45cm_9468.JPG");
run("In");
makeLine(1980, 1338, 2250, 1338);
run("Set Scale...", "distance=270 known=10 pixel=1 unit=mm global");
close();

//******************************************************************************************
// 2. BATCH PROCESS: IMAGE THRESHOLDING, LABELS AND MASKS SAVING OF EACH IMAGE, RESULTS WITH PARTICLE AREA AND MEAN GREY VALUE, OUTLINE MASKS SAVED

macro "Image Analysis Colony Sizes" {
```

```
dir = getDirectory("Choose a Directory ");
print ("Chose Directory:" + dir);
list = getFileList(dir);
print("Directory contains "+list.length+" files and subfolders");
setBatchMode(true);
for (i=0; i<list.length; i++) {
if (endsWith(list[i], "/")) {
print("File is a subfolder and is ignored");
}
else {
if (endsWith(list[i], "db")) {
print("File is a Thumbs.db file and is ignored");
}
else {
path = dir+list[i];
IJ.freeMemory();
print("Free memory:", call("ij.IJ.freeMemory"));
open(path);
name = File.getName(path);
nameshort = File.nameWithoutExtension;
print("Path:", path);
print("Name:", name);
print("Directory:", dir);

// run("Threshold...");
// Color Thresholder 1.46b
// Autogenerated macro, single images only!
min=newArray(3);
max=newArray(3);
filter=newArray(3);
a=getTitle();
run("HSB Stack");
```

```
run("Convert Stack to Images");
selectWindow("Hue");
saveAs("Jpeg", dir+"/HSVtemp/"+nameshort+"Hue.jpg");
rename("0");
selectWindow("Saturation");
saveAs("Jpeg", dir+"/HSVtemp/"+nameshort+"Saturation.jpg");
rename("1");
selectWindow("Brightness");
saveAs("Jpeg", dir+"/HSVtemp/"+nameshort+"Brightness.jpg");
rename("2");
min[0]=57;
max[0]=190;
filter[0]="stop";
min[1]=0;
max[1]=255;
filter[1]="pass";
min[2]=0;
max[2]=255;
filter[2]="pass";
for (j=0;j<3;j++){
selectWindow(""+j);
setThreshold(min[j], max[j]);
saveAs("Jpeg", dir+"/HSVtemp/"+nameshort+"HSVRed"+j+".jpg");
rename(j);
run("Convert to Mask");
saveAs("Jpeg", dir+"/HSVtemp/"+nameshort+"HSVRed"+j+".jpg");
rename(j);
if (filter[j]=="stop")  run("Invert");
}
imageCalculator("AND create", "0","1");
imageCalculator("AND create", "Result of 0","2");
for (k=0;k<3;k++){
```

```
selectWindow(""+k);

close();

}

selectWindow("Result of 0");

close();

selectWindow("Result of Result of 0");

rename(a);

// Colour Thresholding-------------


//*******************************************************************************************

              // 2.1 ANALYZE PARTICLES: NOTE: The Selections are saved to the RoiManager.

run("Set Measurements...", "area mean min fit shape redirect=None decimal=3");

run("Analyze Particles...", "size=0.70-Infinity circularity=0.3-1.00 show=[Overlay Masks] display clear include summarize add");


//*******************************************************************************************

                          // 2.1.1 CALL PLUGIN TO SET LABEL BACKGROUND FILL COLOR, LABEL COLOR AND FONT

// !!!!! NOTE: Here you can make changes for the background of the labels, stroke-color (=outline color), width, fill and even for the labels color and font size!!!!!


call("ij.plugin.filter.ParticleAnalyzer.setFontSize", 50);

run("Overlay Options...", "stroke=yellow width=1 fill=cyan apply");

run("Labels...", "color=black font=30 show bold");


//*******************************************************************************************

              // 2.2 SAVE MASKS AND LABELS


saveAs("Jpeg", dir+"/Labels/"+nameshort+"label.jpg");

run("Remove Overlay");

saveAs("Jpeg", dir+"/Masks/"+nameshort+"mask.jpg");


//*******************************************************************************************
```

```
// 2.3 SAVE RESULTS FORMED ON ORIGINAL IMAGE

run("Clear Results");
open(path);
rename("D");
print("Path:", path);
print("Name:", name);
print("Directory:", dir);

//Note: We add a small polygon with a very low roundness, just so the RoiManager isn’t empty!

makePolygon(4102,3206,4099,3207,4106,3206,4104,3204);
roiManager("Add");
roiManager("Measure");
selectWindow("D");
roiManager("Set Color", "yellow");
roiManager("Set Line Width", 2.5);
run("From ROI Manager");
run("Labels...", "color=yellow font=30 show");
saveAs("Jpeg", dir+"/OutlinesAndBackground/"+nameshort+"outline.jpg");
run("Close");
run("Select None");
selectWindow("Results");
saveAs("Text", dir+"/Results/"+nameshort+"results.txt");
selectWindow("Results");
run("Close");
selectWindow("Log");
run("Close");
roiManager("Delete");
} //ends else if statement
} //ends else statement
} // end for loop batch processing complete folder
```

```
//****************************************************************************************************
// 3. SAVE THE FINAL SUMMARY TO A NEW FOLDER NAMED "SUMMARY"


selectWindow("Summary");
saveAs("Text", dir+"/FinalSummary/"+"Final"+"summary.txt");
run("Close");
print("Macro is finished");
beep();


} //end macro
```
